# Towards the Targeted Protein Degradation of PRMT1

**DOI:** 10.1002/cmdc.202400269

**Published:** 2024-06-20

**Authors:** Poppy L. Martin, Francisco Javier Pérez‐Areales, Shalini V. Rao, Stephen J. Walsh, Jason S. Carroll, David R. Spring

**Affiliations:** ^1^ Yusuf Hamied Department of Chemistry University of Cambridge Cambridge CB2 1EW United Kingdom; ^2^ Cancer Research UK Cambridge Institute University of Cambridge Cambridge CH2 ORE United Kingdom

**Keywords:** PRMT1, PROTAC, Targeted Protein Degradation, Protein Arginine Methyltransferase, Heterobifunctional Molecule

## Abstract

Targeting the protein arginine methyltransferase 1 (PRMT1) has emerged as a promising therapeutic strategy in cancer treatment. The phase 1 clinical trial for **GSK3368715**, the first PRMT1 inhibitor to enter the clinic, was terminated early due to a lack of clinical efficacy, extensive treatment‐emergent effects, and dose‐limiting toxicities. The incidence of the latter two events may be associated with inhibition‐driven pharmacology as a high and sustained concentration of inhibitor is required for therapeutic effect. The degradation of PRMT1 using a proteolysis targeting chimera (PROTAC) may be superior to inhibition as proceeds via event‐driven pharmacology where a PROTAC acts catalytically at a low dose. PROTACs containing the same pharmacophore as **GSK3368715**, combined with a motif that recruits the VHL or CRBN E3‐ligase, were synthesised. Suitable cell permeability and target engagement were shown for selected candidates by the detection of downstream effects of PRMT1 inhibition and by a NanoBRET assay for E3‐ligase binding, however the candidates did not induce PRMT1 degradation. This paper is the first reported investigation of PRMT1 for targeted protein degradation and provides hypotheses and insights to assist the design of PROTACs for PRMT1 and other novel target proteins.

## Graphical abstract

A novel approach to reduce the activity of Protein Arginine Methyltransferase 1 (PRMT1). PROTACs for PRMT1, which contain the pharmacophore of GSK3368715, were rationally designed, innovatively synthesised, and evaluated for activity in cellular assays. PRMT1 is assessed to be amenable to PROTAC‐induced degradation and the insights presented are relevant for the development of future PROTACs for PRMT1 and other novel target proteins.

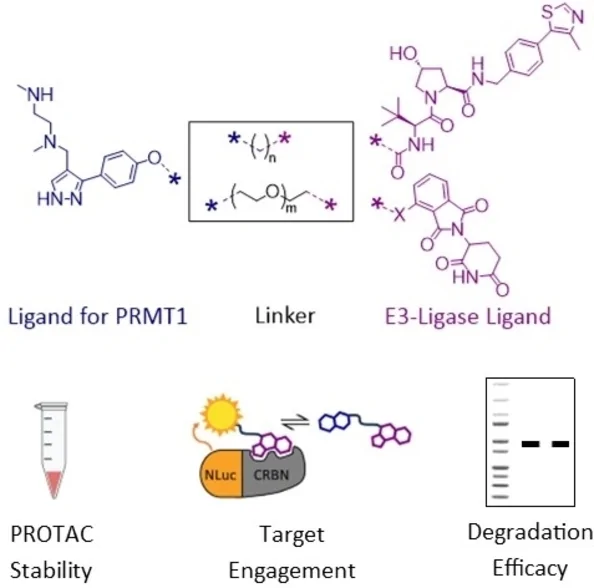

## Introduction

**Figure 1:**
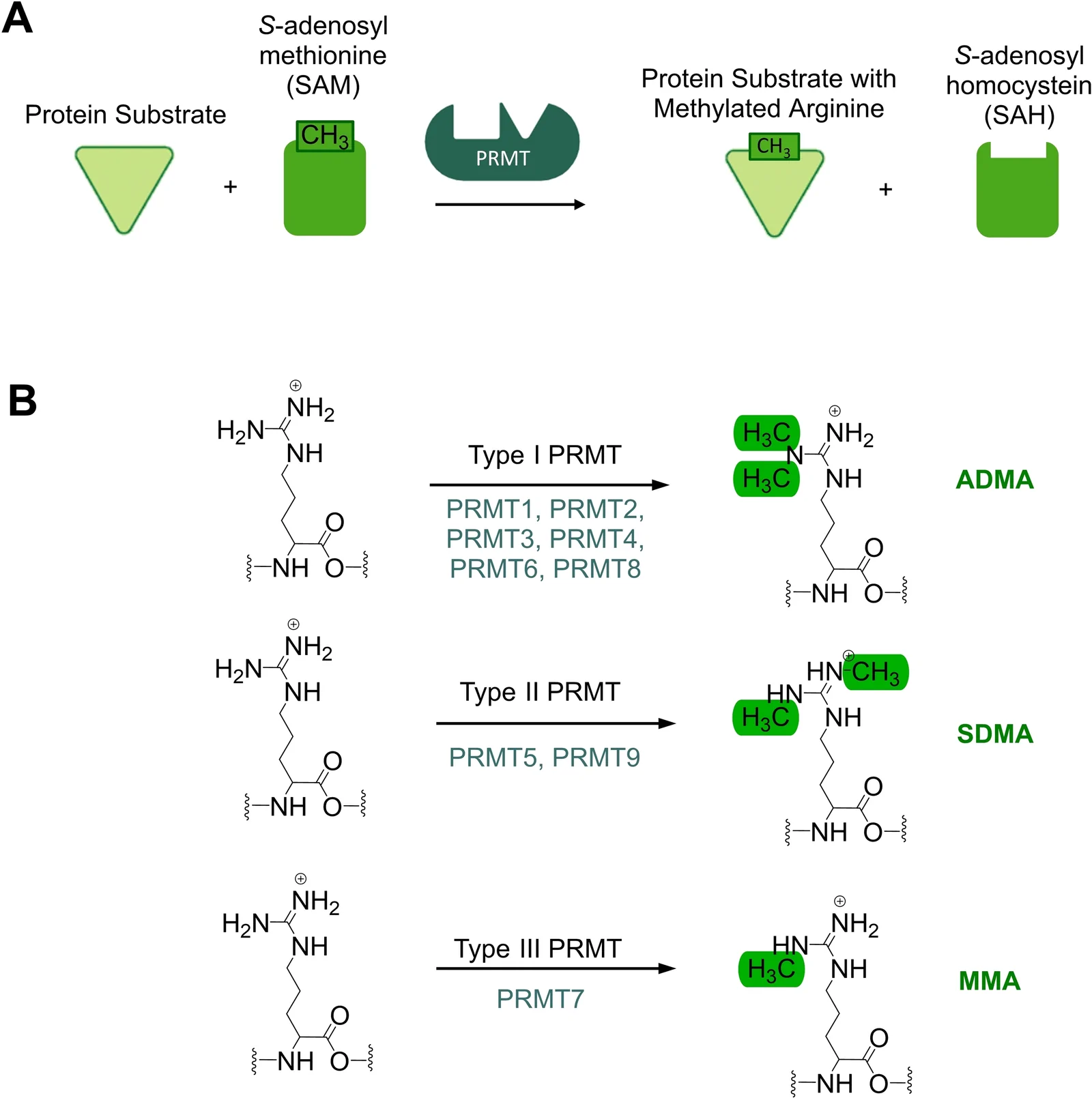
The PRMT catalysed arginine methylation of protein substrates. A) Schematic of the catalytic activity of protein arginine methyltransferases. B) Arginine methylation products. ADMA=asymmetric dimethylation of arginine. SDMA=symmetric dimethylation of arginine. MMA=monomethylation of arginine.

Arginine methylation is a post translational modification (PTM) that regulates many cellular processes including signal transduction, mRNA splicing, transcriptional control, DNA repair and protein translocation.^[1]^ The protein arginine methyltransferases (PRMTs) are a family of proteins responsible for the transfer of a methyl group from *S*‐adenosyl methionine (SAM) to the guanidium group of arginine residues in protein substrates. This PTM alters the protein substrate's structure and hydrophobicity, which can lead to a change in its localisation, enzymatic ability and interactome.^[2]^ There are nine PRMT proteins that are categorised into three types depending on the resulting arginine‐methylated product (Figure 1). Each PRMT protein has a distinct and non‐redundant role in the cell due to high substrate specificity.^[3]^ PRMT1 is the predominant Type I PRMT and accounts for 85 % of cellular asymmetric dimethylation of arginine (ADMA) levels.^[4]^ Specific protein substrates of PRMT1 include histones, for example H3R4,^[5]^ as well as non‐histone proteins, for example the progesterone receptor.^[6]^

In clinical samples of breast cancer and pancreatic ductal adenocarcinoma (PDAC), upregulated PRMT1 expression is associated with poor prognosis.[^6^, ^7^, ^8^, ^9^, ^10^, ^11^] Aberrant PRMT1 expression results in the dysregulation of transcription and promotes tumourigenesis,^[2]^ and PRMT1 knockdown in cell and animal models of breast cancer and PDAC evokes a reduction in tumour cell proliferation.[^5^, ^6^, ^10^, ^11^, ^12^, ^13^] *In vitro* studies have highlighted PRMT1 as a promising target in combination treatments as synergistic interactions have been identified between PRMT1 and various chemotherapeutics,^[12]^ immunotherapeutics,^[14]^ as well as inhibitors of the epidermal growth factor receptor[^15^, ^16^] and the Type II PRMT, PRMT5.[^17^, ^18^] Thus, reduction of PRMT1 activity has emerged as a promising therapeutic strategy.^[19]^

Potent small‐molecule inhibitors of PRMT1 have been published although none exhibit selectivity for PRMT1 over other Type I PRMTs.[^18^, ^20^, ^21^, ^22^] **GSK3368715** is a pan‐Type I PRMT inhibitor and entered a phase I clinical trial for the treatment of solid tumours and diffuse large B‐cell lymphoma [NCT03666988] (Table 1). Early termination of the trial however occurred due to limited efficacy, low target engagement at the tumour level, and severe adverse effects.^[23]^ It is unclear whether the lack of clinical efficacy and poor safety profile of **GSK3368715** are a result of pan‐Type I PRMT inhibition or a result of off‐target effects and poor pharmacokinetic properties specific to **GSK3368715**. Altogether, the clinical utility of targeting PRMT1 remains elusive and an alternative tool for its investigation is needed.

**Table 1:** Structure and selectivity profile of **GSK3368715** for the PRMTs. IC_50_ values from published biochemical assays.^[18]^

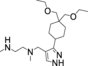		
Protein	Type	IC_50_, nM
PRMT1	I	3
PRMT3	I	162
PRMT4	I	38
PRMT6	I	5
PRMT8	I	4
PRMT5	II	>20,000
PRMT9	II	>20,000
PRMT7	III	>40,000

A PROTAC is a heterobifunctional molecule that hijacks the ubiquitin proteasome system for the degradation of a protein of interest. A PROTAC induces proximity between an E3‐ligase and the target protein, triggering its ubiquitination and subsequent degradation by the proteasome. This ‘event‐driven’ mechanism of action enables a PROTAC to act catalytically and mediate the ubiquitination of multiple copies of a given target protein,^[24]^ and therefore a low dose may elicit a strong biological response without off‐target toxicity. A PROTAC for PRMT1 may have greater efficacy and a better safety profile compared to an inhibitor of PRMT1 which requires a high and sustained concentration for a therapeutic effect.^[25]^

## Results and Discussion

### PROTAC Design for PRMT1

**Figure 2:**
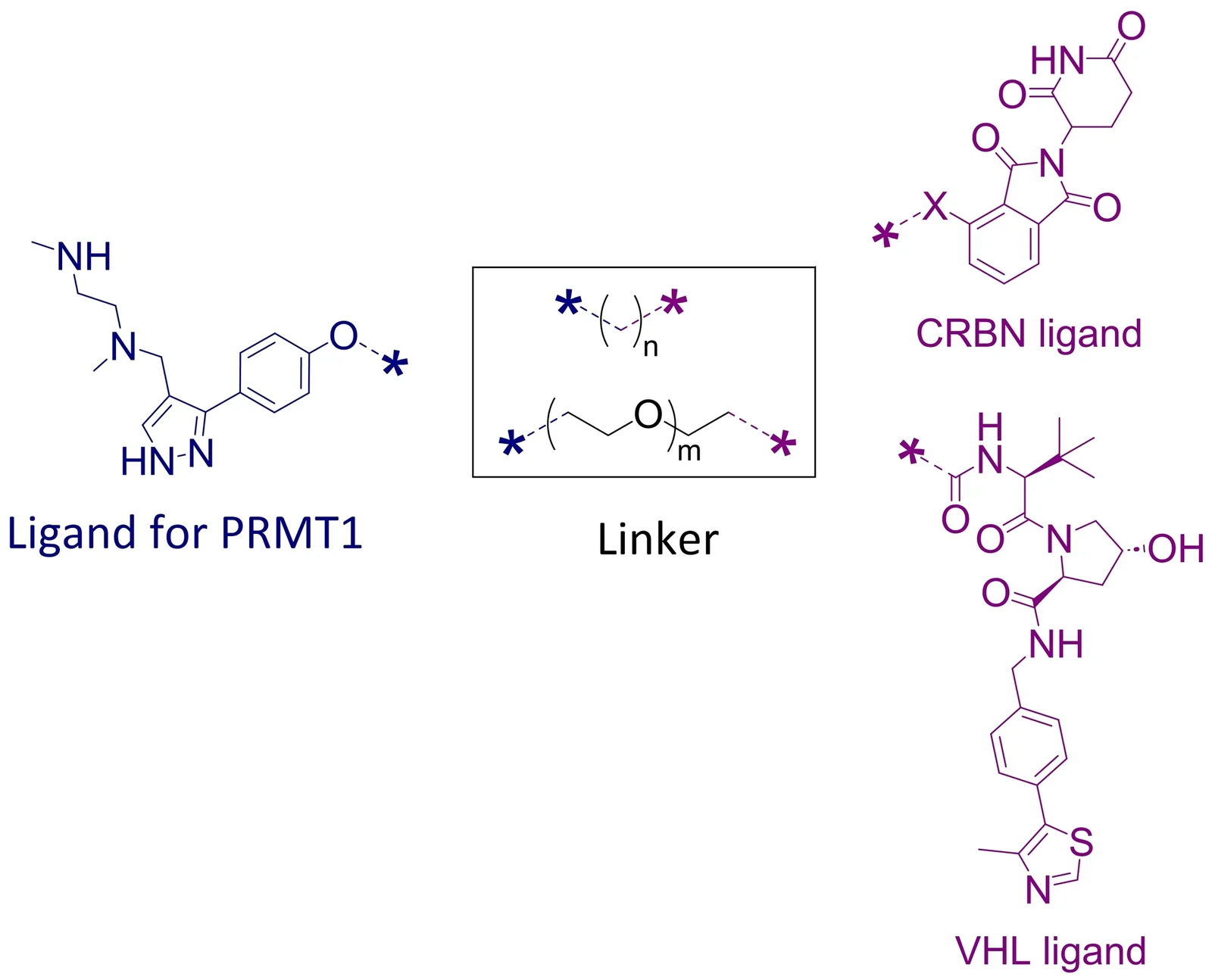
General Design of PRMT1 PROTACs.

PRMT1 is an amenable protein for PROTAC‐induced degradation according to the 4‐point ‘PROTACability’ criteria laid out by Schneider *et al*.^[26]^ First, PRMT1 shuttles between the nucleus and cytoplasm,[^3^, ^7^] and high degradation efficacy with PROTACs has been observed in these subcellular locations.^[27]^ Second, endogenous PRMT1 is degraded by the ubiquitin proteasome system; ubiquitination sites[^28^, ^29^, ^30^] and degron motifs^[31]^ have been reported for PRMT1, and its degradation is both E3‐ligase and proteasome dependent.[^32^, ^33^, ^34^] In the MCF‐7 cell line, it was corroborated that PRMT1 degradation occurs via the proteasome (Figure S1, ESI†). The third criterion requires information on the half‐life of the target protein. The published half‐life of PRMT1 in dividing mammalian cell lines varies between 4–70 h (Table S1, ESI†). This was confirmed in a cycloheximide chase experiment in the MCF‐7 cell line where a half‐life greater than 8 h was observed (Figure S2, ESI†). We however consider that criterion 3 does not have any discriminatory value as, despite the half‐life of a target protein often discussed as a critical parameter for the efficacy of PROTAC‐induced degradation, quantitative values for a suitable half‐life have not been reported.[^26^, ^35^, ^36^, ^37^] It is probable that, rather than the half‐life having a defined value, the PROTAC‐induced rate of degradation must be significantly faster than the resynthesis rate of the endogenous protein,^[38]^ and this will depend on the efficacy and kinetics of the PROTAC. Finally, criterion 4 requires the availability of small‐molecule ligands for the target protein, exemplified for PRMT1 by **GSK3368715**. Thus, a library of PROTAC candidates that comprise an E3‐ligase ligand and a ligand for PRMT1 connected by various linkers was designed (Figure 2).

Ligands for the Cullin‐RING E3‐ligases Von Hippel‐Lindau (VHL) and Cereblon (CRBN) were selected as these E3‐ligases are present in the same cellular locations as PRMT1.^[27]^ The pharmacophore of **GSK3368715** was selected for the PRMT1 ligand as it has high potency for PRMT1 and binds reversibly.[^18^, ^21^] The clinical limitations of **GSK3368715** do not preclude its use in a PROTAC. First, PRMT1 target engagement was observed in peripheral blood mononuclear cells with **GSK3368715** showing that this molecule binds to PRMT1.^[23]^ Second, the poor safety profile of **GSK3368715** may be associated with off‐target effects that are associated with a high and sustained concentration of this inhibitor in the cell. Finally, the low target engagement in the tumour (and subsequent lack of clinical efficacy) may be a result of pharmacokinetic factors.^[39]^ The crystal structure of **GSK3368715** bound to PRMT1^[18]^ and structure activity studies^[21]^ show that the pyrazole and ethylenediamino group are essential for the potent inhibition of PRMT1 activity and thus deemed the pharmacophore for PRMT1 binding. Conversely, substituents are tolerated at the para‐position of the aryl ring attached to this pharmacophore without a loss of potency,^[21]^ and a derivative with a phenol substituent was confirmed to inhibit PRMT1 (Figure S3, ESI†). Thus, this position of the aryl ring was selected for linker attachment.

For linker design, an iterative approach was followed where a library of PROTAC candidates containing linkers of varying lengths and polarities was investigated. Alkyl and polyethylene glycol (PEG) linkers were explored as their high flexibility can allow for multiple spatial orientations between the E3‐ligase and the target protein,^[40]^ and their different hydrophobicity allows for the screening of pharmacokinetic properties.[^41^, ^42^] This maximises the likelihood of forming a ternary complex that enables ubiquitin transfer to occur between the E3‐ligase and target protein.

### VHL‐Recruiting PROTACs

**Scheme 1:**
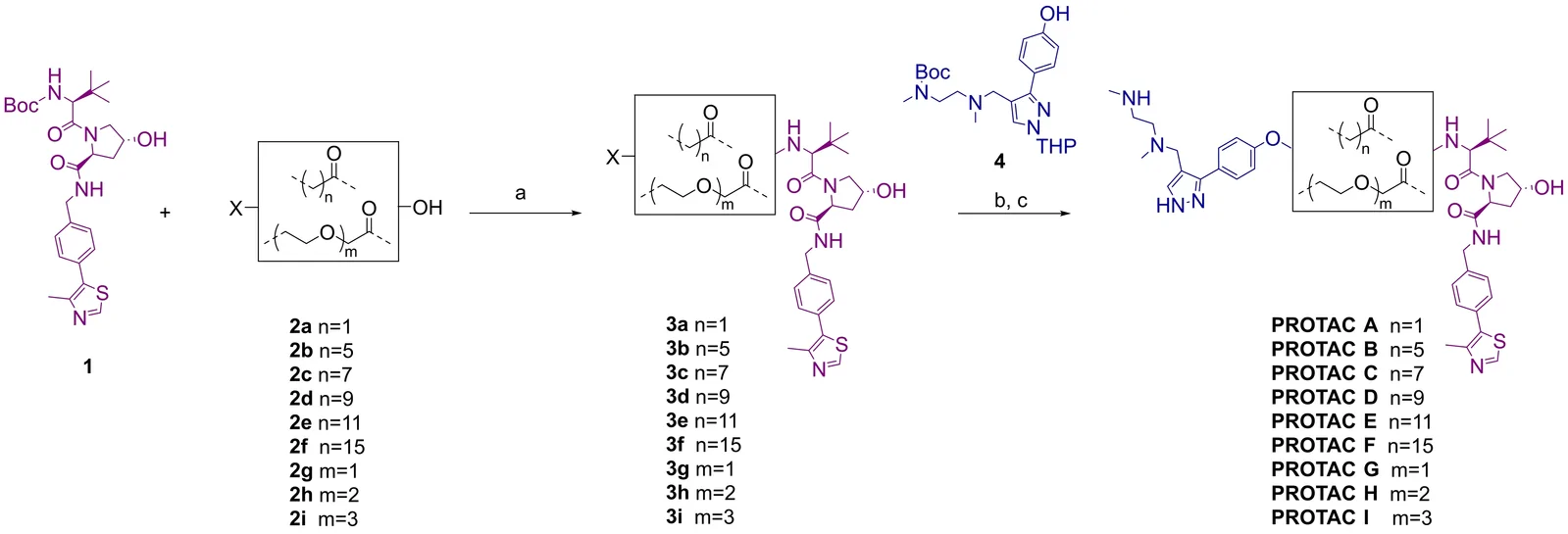
Synthesis of VHL‐recruiting PROTACs. X=Chloride or Bromine. Reagents and conditions: a) 1. TFA, DCM, rt, 1 h. 2. HATU, DIPEA, EtOAc, DMF, rt, 0.5–3 h. b) 4, KI, K_2_CO_3_, MeCN, 80 °C, 1–5 days, c) HCl, MeOH, rt, 1 h.

PROTAC candidates that recruit the VHL E3‐ligase were synthesised from VHL ligand **1** which underwent amide coupling reactions with linkers **2 a**–**i** which contain alkyl and PEG chains of various lengths. PRMT1 ligand **4** was then *O*‐alkylated with halides **3 a**–**i** and the resulting products were deprotected to give **PROTACs A‐I** (Scheme 1).

A sensitive and selective Western blot protocol was validated to investigate the effect of PROTAC treatment on the cellular levels of PRMT1 in the MCF‐7 cell line (Figure S4, ESI†). Derived from a metastatic breast adenocarcinoma, the MCF‐7 cell line has the hormone receptor status that characterises the most frequently diagnosed subtype of breast cancer: ER‐positive, PR‐positive, HER2‐negative.[^43^, ^44^] This breast cancer subtype, and consequently the MCF‐7 cell line, are a suitable model system to predict the clinical efficacy of PRMT1 PROTACs; PRMT1 upregulation correlates with a reduction in relapse‐free survival for patients with this breast cancer subtype[^6^, ^45^] and anti‐proliferative effects have been observed in the MCF‐7 cell line following pan‐Type I PRMT inhibition.[^17^, ^20^]

**Figure 3:**
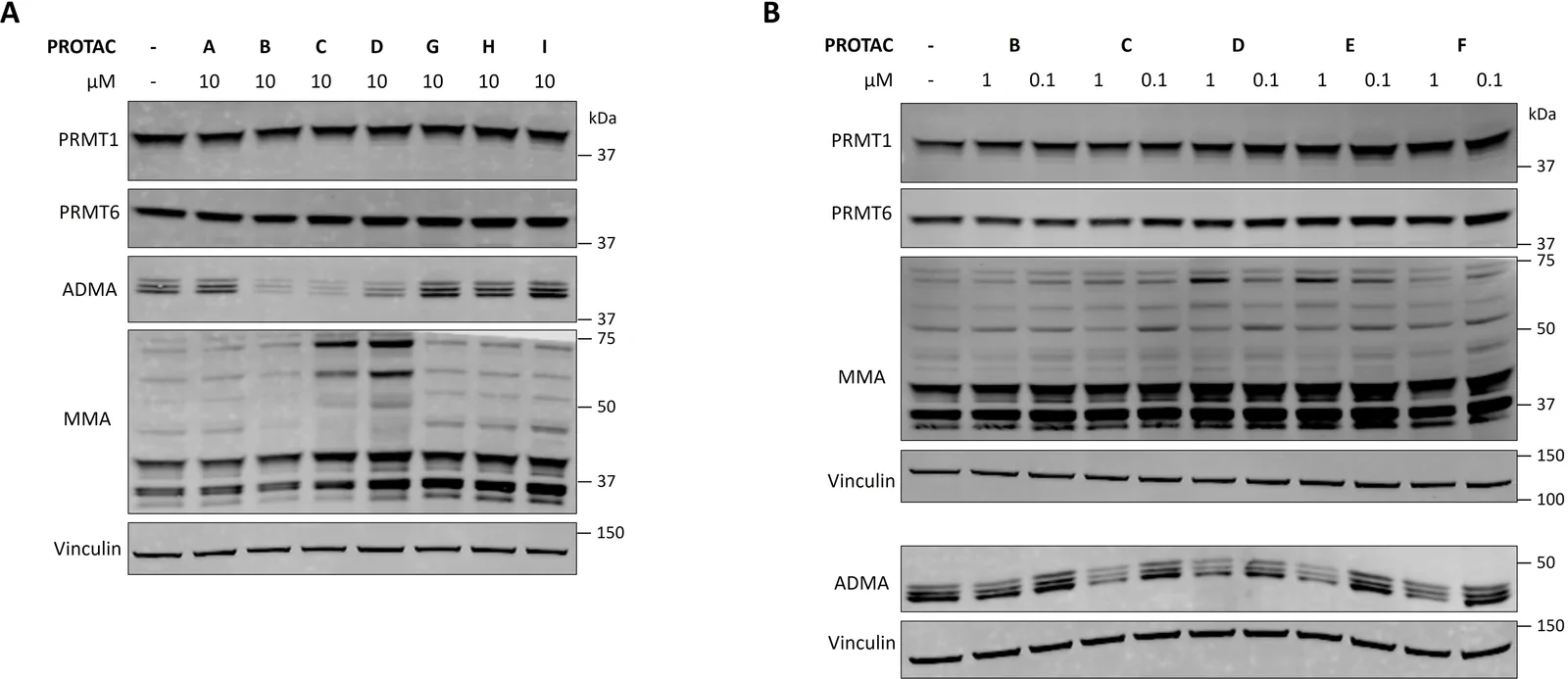
PRMT1 and PRMT6 degradation assays with VHL‐recruiting PROTACs. A) **PROTACs A**–**I** at 10 μM. **PROTACs E**–**F** were cytotoxic and not analysed. MCF‐7 cells were treated with the indicated compound for 24 h and then harvested for analysis by Western blot. B) Same as A but PROTACs tested at 1 and 0.1 μM. Image representative of two independent experiments. Uncropped blots are shown in ESI†.

At 10 μM in the MCF‐7 cell line, PRMT1 degradation was not observed with any of the synthesised PROTACs (Figure 3A). **PROTAC E** and **PROTAC F** were cytotoxic and not analysed. **GSK3368715** has similar IC_50_ values for PRMT1, PRMT6 and PRMT8 (Table 1) and therefore the PROTACs were also investigated for their ability to degrade PRMT6 (degradation not observed). PRMT8 is expressed primarily in the brain and thus not investigated.^[46]^

**PROTACs A‐I** are expected to retain their ability to inhibit PRMT1 and therefore their ability to permeate the cell membrane, and bind to PRMT1, was determined by measuring changes in two post‐translational modifications: asymmetric dimethylation of arginine (ADMA) and monomethylation of arginine (MMA). **PROTACs B‐D** showed a decrease in ADMA which is indicative of Type I PRMT inhibition. The significant increase in MMA observed for **PROTAC C** and **PROTAC D** can be attributed to the selective inhibition of PRMT1.[^13^, ^47^] PROTACs can exhibit a hook effect where increasing the PROTAC concentration above an optimal level reduces their degradation efficacy.[^48^, ^49^] To ensure that the ability of the PROTACs to degrade PRMT1 was not masked by the hook effect, the PROTACs that inhibited PRMT1 or were cytotoxic were further tested at 1 and 0.1 μM. Neither PRMT1 nor PRMT6 degradation was observed (Figure 3 B). The PROTACs were also tested in two PDAC‐derived cell lines (KP‐3 and HPAF‐II) and PRMT1 degradation was not observed (Figure S5, ESI†).

### CRBN‐Recruiting PROTACs

**Scheme 2:**
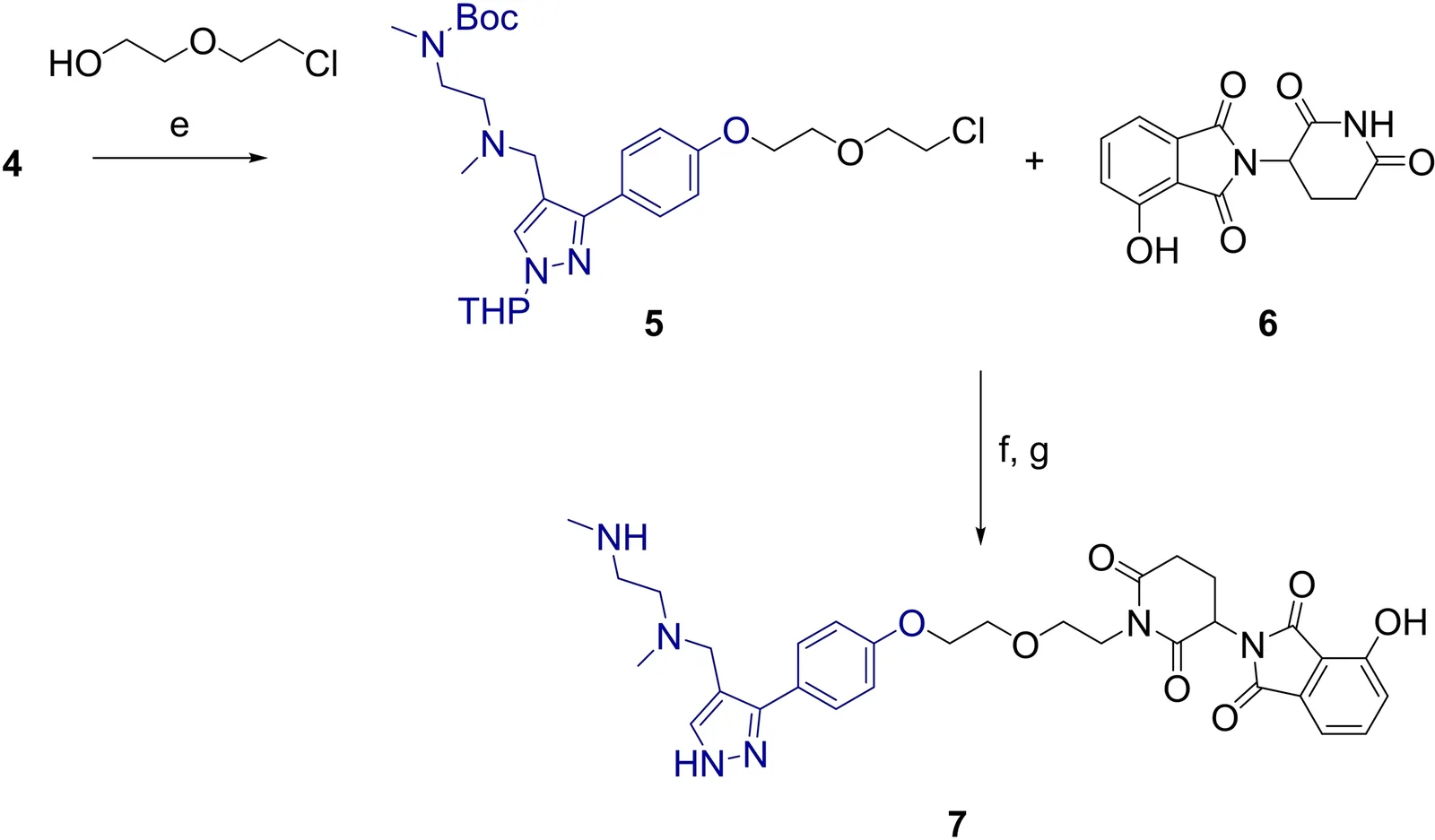
Alkylation of CRBN ligand **6** gave the undesired *N*‐alkylated product **7**. Reagents and conditions: e) 2‐(2‐chloroethoxy)ethanol, PPh_3_, DTBAD, THF, rt, o/n. f) KI, K_2_CO_3_, DMSO, 80 °C, o/n. g) HCl, MeOH, rt, 1 h.

To synthesise PROTACs that recruit the CRBN E3‐ligase, PRMT1 ligand **4** underwent a Mitsunobu reaction to produce **5** which was then used for the alkylation of CRBN ligand **6**. This resulted in the undesired *N*‐alkylation of the glutarimide ring to give heterobifunctional molecule **7** that does not bind CRBN (Scheme 2, Figure S6 ESI†).

**Scheme 3:**
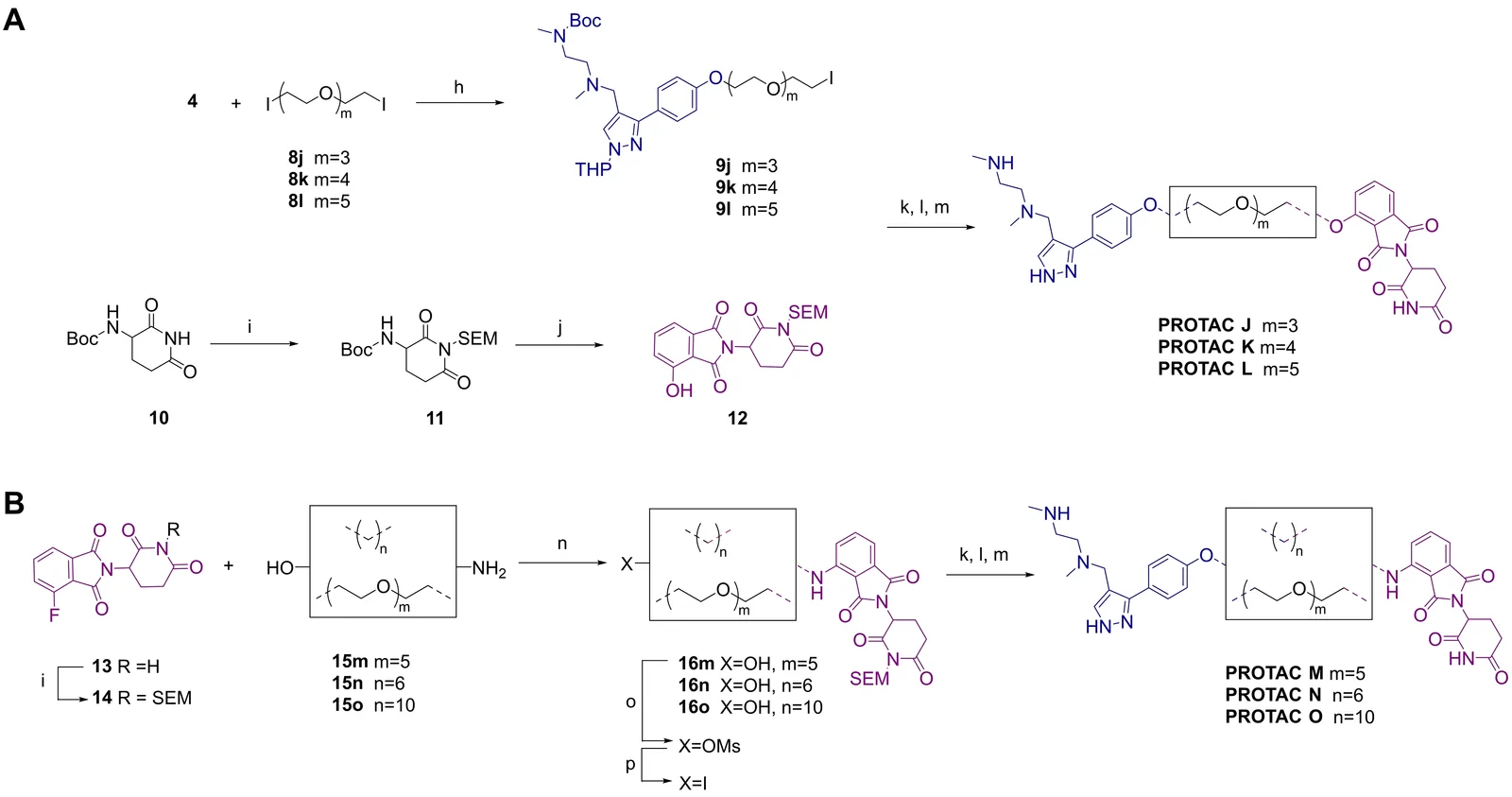
Synthesis of CRBN‐recruiting PROTACs. A) Ether bond between the linker and the CRBN ligand. B) Amino group between the linker and CRBN ligand. Reagents and conditions: h) Cs_2_CO_3_, DMF, rt, o/n. i) SEMCl, DBU, rt, 1 h. j) 4‐hydroxyisobenzofuran‐1,3‐dione, trifluoroethanol, microwave, 150 °C, 2 h. k) 9 or 4, Cs_2_CO_3_, DMF, 80 °C, o/n. l) HCl, MeOH, rt, 1 h. m) NH_4_OH, MeCN, rt, 5 min. n) DIPEA, DMSO, 130 °C, o/n. o) MsCl, Et_3_N, DCM, rt, 3 h. p) NaI, MeCN, 50 °C, o/n.

A SEM protecting group was employed on the glutarimide ring of CRBN ligand **6**. Three CRBN‐recruiting PROTACs were synthesised by the *O*‐alkylation of PRMT1 ligand **4** with the corresponding linker **8 j**–**l** to give **9 j**–**l** which underwent a subsequent *O*‐alkylation with SEM‐protected CRBN ligand **12**. The removal of the protecting groups was then attempted using acidic conditions. The *N*‐Boc and THP protecting groups on the PRMT1 ligand were removed however the SEM group on the CRBN ligand was converted to an *N*‐hydroxymethyl functional group. This group was removed under basic conditions to afford **PROTACs J**–**L** (Scheme 3 A). It is documented that the glutarimide ring and the phthalimide group of the CRBN ligand are susceptible to hydrolysis,[^50^, ^51^] and the stability of **PROTAC L** in cell culture media was investigated and found to be short (t_1/2_=0.9 h). Changing the functional group between the CRBN ligand and linker can affect the susceptibility of the ligand to hydrolysis.^[52]^ **PROTAC M** was synthesised, which comprised a similar structure as **PROTAC L**, but with an amino group between the linker and CRBN ligand (Scheme 3 B). Pleasingly, increased stability in cell culture media was observed with **PROTAC M** (t_1/2_=2.1 h). Two further PROTACs were synthesised (**PROTAC N** and **PROTAC O**) which contain an alkyl linker; it was observed for the VHL‐recruiting PROTACs that only PROTACs with an alkyl linker inhibited PRMT1 activity, suggesting that linker identity affects affinity to PRMT1.

**Figure 4:**
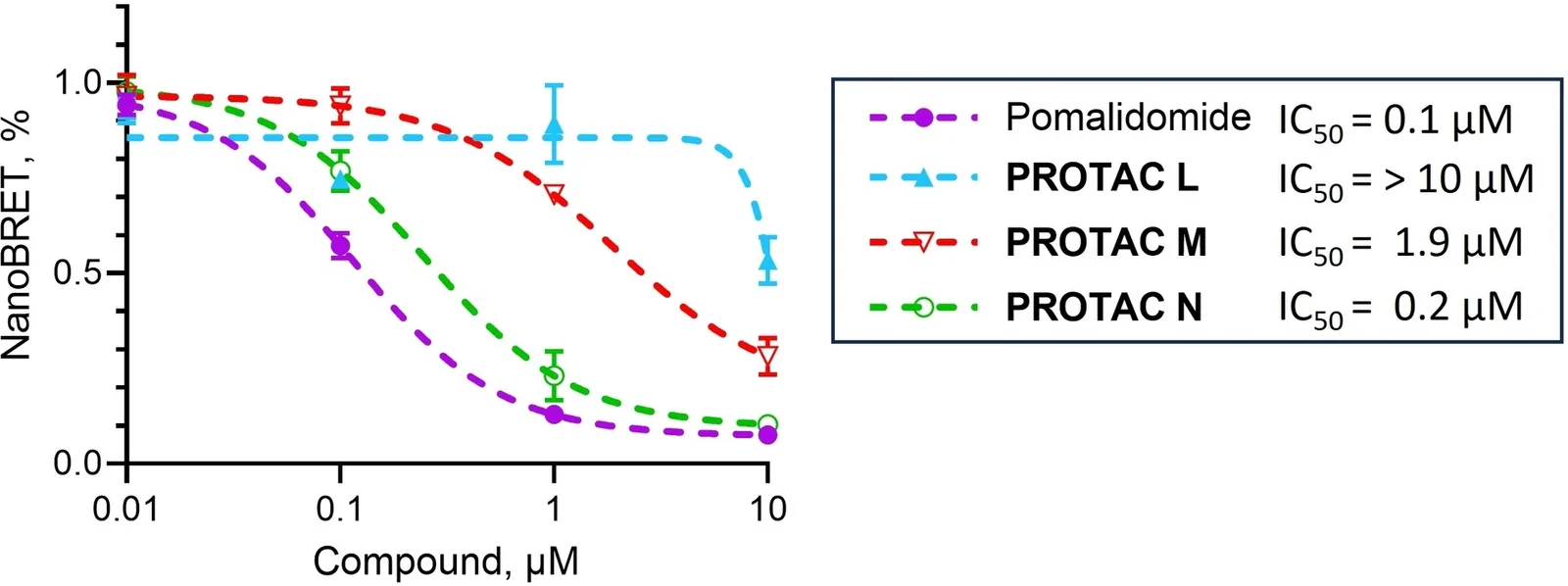
Target engagement of PROTACs to NLuc‐CRBN. Data is normalised to untreated cells and BRET calculated by BRET emission intensity / NLuc emission intensity. IC_50_ is defined as the concentration of the compound that results in half‐maximal inhibition of tracer binding to NLuc‐CRBN fusion protein and was calculated using the [inhibitor] vs. response curve fit with variable slope in GraphPad Prism. The mean and standard error of two independent experiments, with multiple biological replicates, are shown.

To confirm that the PROTACs are able to enter the cell and bind CRBN, the Promega NanoBRET Target Engagement assay was undertaken. In this assay, bioluminescence resonance energy transfer (BRET) is achieved by the transfer of luminescent energy from a fusion protein comprising NanoLuc luciferase and CRBN (NLuc‐CRBN) to a fluorescent tracer. When the tracer is bound to NLuc‐CRBN, BRET occurs. When the tracer is incubated with a compound that also binds to the CRBN‐NLuc fusion protein, there is competition for binding and a decrease in BRET. The decrease in BRET is proportional to ‘target engagement’ which refers to the ability of the PROTAC to bind to the NLuc‐CRBN fusion protein and this will reflect the PROTAC's binding affinity to CRBN as well as cell permeability and PROTAC stability. The PROTACs investigated showed variable target engagement. A comparison of **PROTAC L** and **PROTAC M** shows a large difference (IC_50_=>10 μM and IC_50_=1.9 μM respectively) despite their structures differing only by the functional group between the linker and the CRBN ligand. This suggests that an amino group leads to greater CRBN target engagement, likely due to increased stability (Figure 4). **PROTAC N**, containing an alkyl chain linker, was the most potent PROTAC investigated.

**Figure 5:**
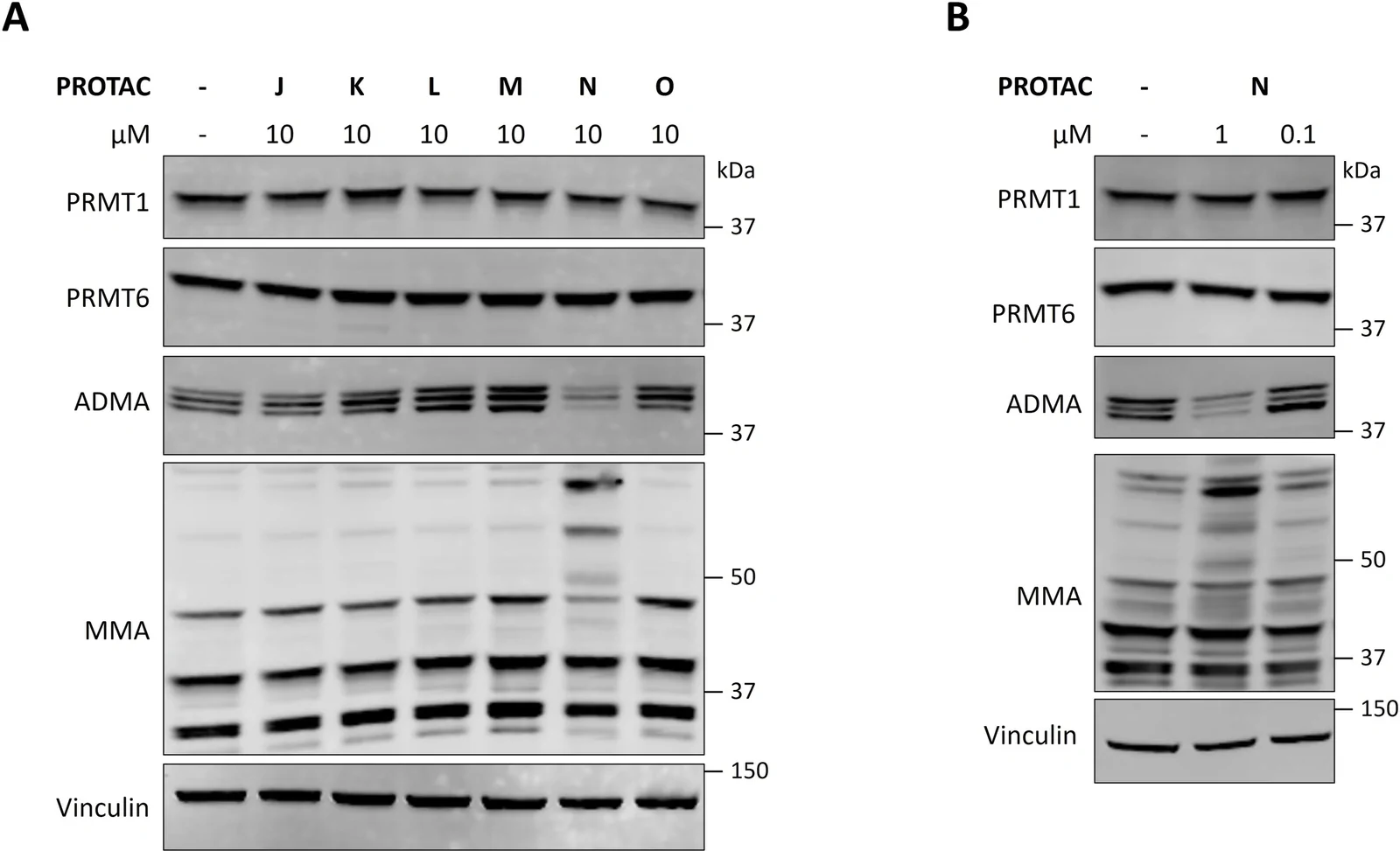
PRMT1 and PRMT6 degradation assays with CRBN‐recruiting PROTACs. A) **PROTACs J**–**O** at 10 μM. MCF‐7 cells were treated with the indicated compound for 24 h and then harvested for analysis by Western blot. B) Same as A but **PROTAC N** tested at 1 and 0.1 μM. Images representative of two independent experiments. Uncropped blots are shown in ESI†.

The CRBN‐recruiting PROTACs, **PROTAC J‐O** were evaluated for PRMT1 degradation efficacy in the MCF‐7 cell line and degradation was not observed with any of the candidates at 10 μM. **PROTAC N** inhibited PRMT1 activity and when tested at lower concentrations to account for the hook effect, neither PRMT1 nor PRMT6 degradation was observed (Figure 5). Degradation was not observed with any of the CRBN‐recruiting PROTACs in the KP‐3 and HPAF‐II cell lines at 10 μM (Figure S7, ESI†), nor following a 6 h incubation in the MCF‐7 cell line (Figure S8, ESI†).

## Conclusions

Fifteen PROTACs that target PRMT1 were investigated. Neither PRMT1 nor PRMT6 degradation was observed with any of the PROTACs synthesised. Cell permeability and PRMT1 binding were shown with selective PROTACs by the increased level of monomethylated arginine upon PROTAC treatment. Also, despite the short half‐life of the CRBN‐recruiting PROTACs in cell culture media, **PROTACs L‐N** were shown to bind CRBN.

Hypotheses are presented for why degradation was not observed. First, the PROTACs synthesised may not induce the formation of a ternary complex that facilitates ubiquitin transfer. This may be due to unsuitable linker length.^[53]^ Alternatively, a PROTAC‐induced ternary complex may not result in PRMT1 degradation due to the distance and relative orientation between the ubiquitin‐charged E2‐enzyme and a lysine that can be ubiquitinated on the surface of PRMT1,[^54^, ^55^] or the incorrect topology of the PROTAC‐induced polyubiquitin chain on the target protein.^[56]^

**Figure 6:**
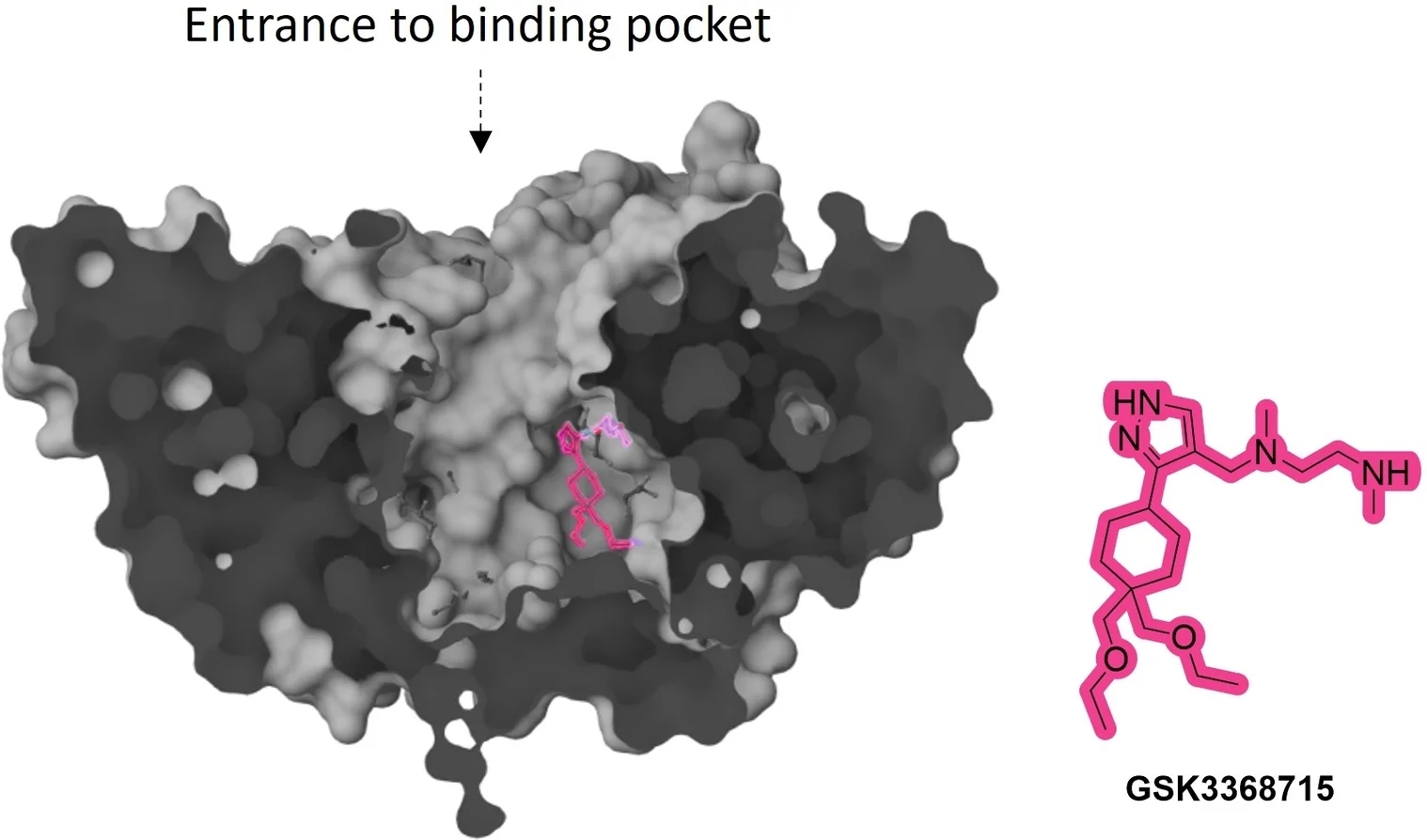
Crystal structure of **GSK3368715** bound to PRMT1 (PDB: 6NT2). PRMT1 is active as a homodimer where a toroidal structure is formed from two PRMT1 protein molecules, and the binding pocket of each PRMT1 molecule faces inwards and into a solvent‐filled channel. A molecular surface representation of the substrate binding pocket of PRMT1 (grey). **GSK3368715** as a ball and stick model (pink) and the chemical structure is shown at the correct orientation. Crystal structure published in Fedoriw *et al*.^[18]^ and visualised using Mol*.^[57]^

Second, properties of the chosen PRMT1 ligand may prevent the PROTAC from simultaneously binding PRMT1 and the E3‐ligase. **GSK3368715** binds at the base of the large deep substrate binding pocket of PRMT1^[18]^ (Figure 6) and the PROTACs synthesised should share a similar binding mode. This may limit the ability of the E3‐ligase ligand to project into the intracellular fluid to recruit the E3‐ligase. When selecting the PRMT1 ligand, focus was on the selection of a potent PRMT1 ligand that could tolerate linker attachment without a loss in PRMT1 binding affinity. The spatial position of the ligand bound to PRMT1, and the predicted trajectory of the linker and E3‐ligase ligand, were not considered and may be unsuitable. Future PROTACs for PRMT1 should use a different PRMT1 ligand that binds at a shallower position on PRMT1. It may also be advantageous to bind at an allosteric site on PRMT1 to improve the selectivity of degradation as there is high sequence homology between the Type I PRMTs in the substrate and SAM binding pockets.^[22]^

A potent PROTAC for PRMT1 has the potential to be a therapeutic strategy for cancer treatment and this work should assist the future design and biological evaluation of PROTACs that target PRMT1.

## Supporting Information

The authors have cited additional references within the Supporting Information.[^58^, ^59^, ^60^, ^61^, ^62^, ^63^, ^64^]

## Author Contributions

P.L.M – investigation and data analysis. F.J.P.A and S.V.R – supervision and data analysis. S.J.W – conceptualisation. J.S.C and D.R.S – conceptualisation and supervision. Writing – original draft, P.L.M; review and editing, all authors.

## Conflict of Interests

The authors declare no conflict of interest.

## Supporting information

As a service to our authors and readers, this journal provides supporting information supplied by the authors. Such materials are peer reviewed and may be re‐organized for online delivery, but are not copy‐edited or typeset. Technical support issues arising from supporting information (other than missing files) should be addressed to the authors.

Supporting Information

## Data Availability

The data that support the findings of this study are available in the supplementary material of this article.
