# Supplementary material for: Towards the Targeted Protein Degradation of PRMT1
Source: ChemMedChem. 2024 Jun 20;19(16):e202400269. doi: 10.1002/cmdc.202400269 (PMC13494812; doi:10.1002/cmdc.202400269)
Supplement: Supplementary file 1 [file CMDC-19-e202400269-s001.pdf]

ChemMedChem

Supporting Information

Towards the Targeted Protein Degradation of PRMT1

Poppy L. Martin, Francisco Javier Pérez-Areales, Shalini V. Rao, Stephen J. Walsh, Jason S. Carroll, and David R. Spring*

Towards the Targeted Protein Degradation of PRMT1

Supporting Information

Poppy L. Martin,[a] Francisco Javier Pérez-Areales,[a] Shalini V. Rao,[b] Stephen J. Walsh,[a] Jason S. Carroll[b] and David R. Spring.[a], *

[a] Yusuf Hamied Department of Chemistry, University of Cambridge, Cambridge, CB2 1EW. E-mail: spring@ch.cam.ac.uk

[b] Cancer Research UK Cambridge Institute, University of Cambridge, Cambridge, CH2 ORE

Contents

1. Supplementary Figures 2
2. Supplementary Tables 7
3. Uncropped Scans of Western Blots shown in Figure 3 and 5 8
4. Experimental: Cell-based Assays 12
5. Experimental: Chemistry 15
  5.1 General Experimental 15
  5.2 PROTAC Stability in Cell Culture Media 16
  5.3 Synthesis 17
    5.3.1 VHL-Recruiting PROTACs 17
    5.3.2 CRBN-Recruiting PROTACs 31
    5.3.3 PRMT1 Ligand 4 44
    5.3.4 VHL ligand 1 48
7. NMR Spectra 49

1. Supplementary Figures

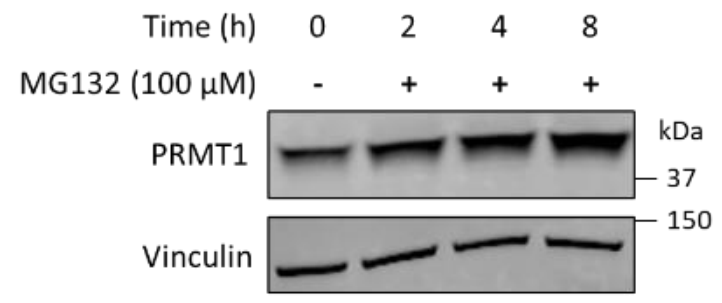

Figure S1. PRMT1 degradation is proteasome dependent in the MCF-7 cell line. MCF-7 cells were treated with the proteasome inhibitor MG132 (100 µM) for the indicated time and PRMT1 levels were analysed by Western blot. Results are representative of two independent experiments.

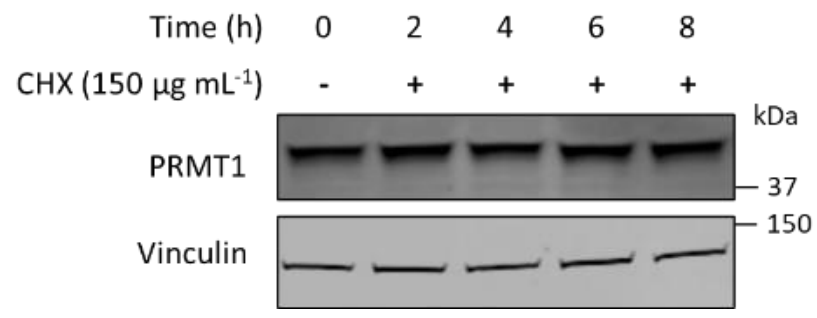

Figure S2. The half-life of PRMT1 in MCF-7 cell line is greater than 8 h. MCF-7 cells were seeded for the experiment in DMEM supplemented with 10% FBS and left for 24 h. The media was then replaced with serum free DMEM media and the cells incubated for a further 24 h. The media was then removed and replaced with serum free DMEM media containing 150 µg mL^{-1} cycloheximide (CHX). The cells were incubated for the indicated time and PRMT1 levels were analysed by Western blot. Results are representative of two independent experiments.

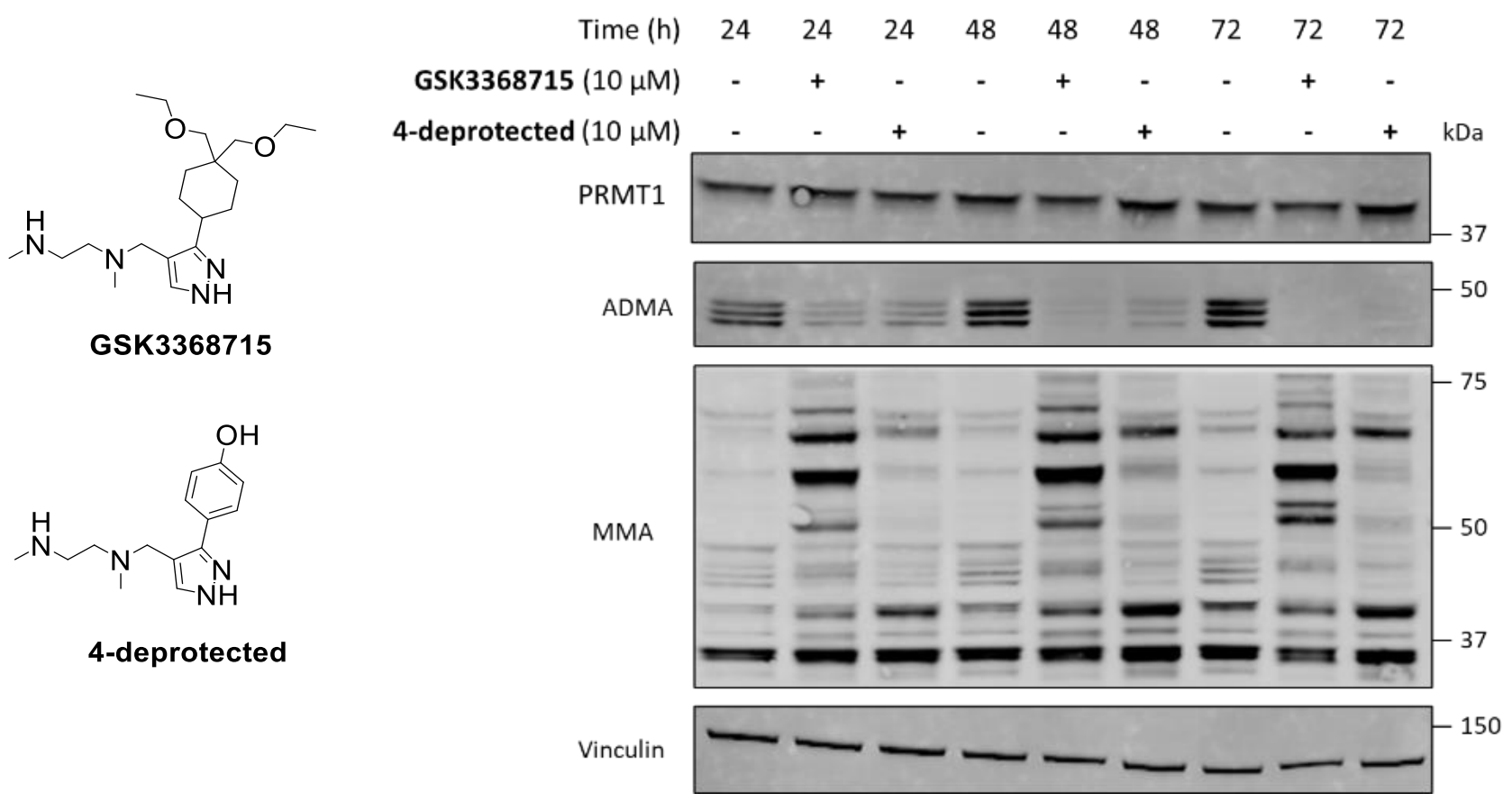

Figure S3. The chosen PRMT1 ligand inhibits PRMT1 activity. **GSK3368715** and **4-deprotected** inhibit the activity of PRMT1 and Type I PRMTs. MCF-7 cells were treated with the indicated compound for 24, 48 or 72 h and then protein levels were analysed by Western blot. The level of the post-translational modifications ADMA and MMA were analysed in addition to the protein levels of PRMT1 and vinculin.

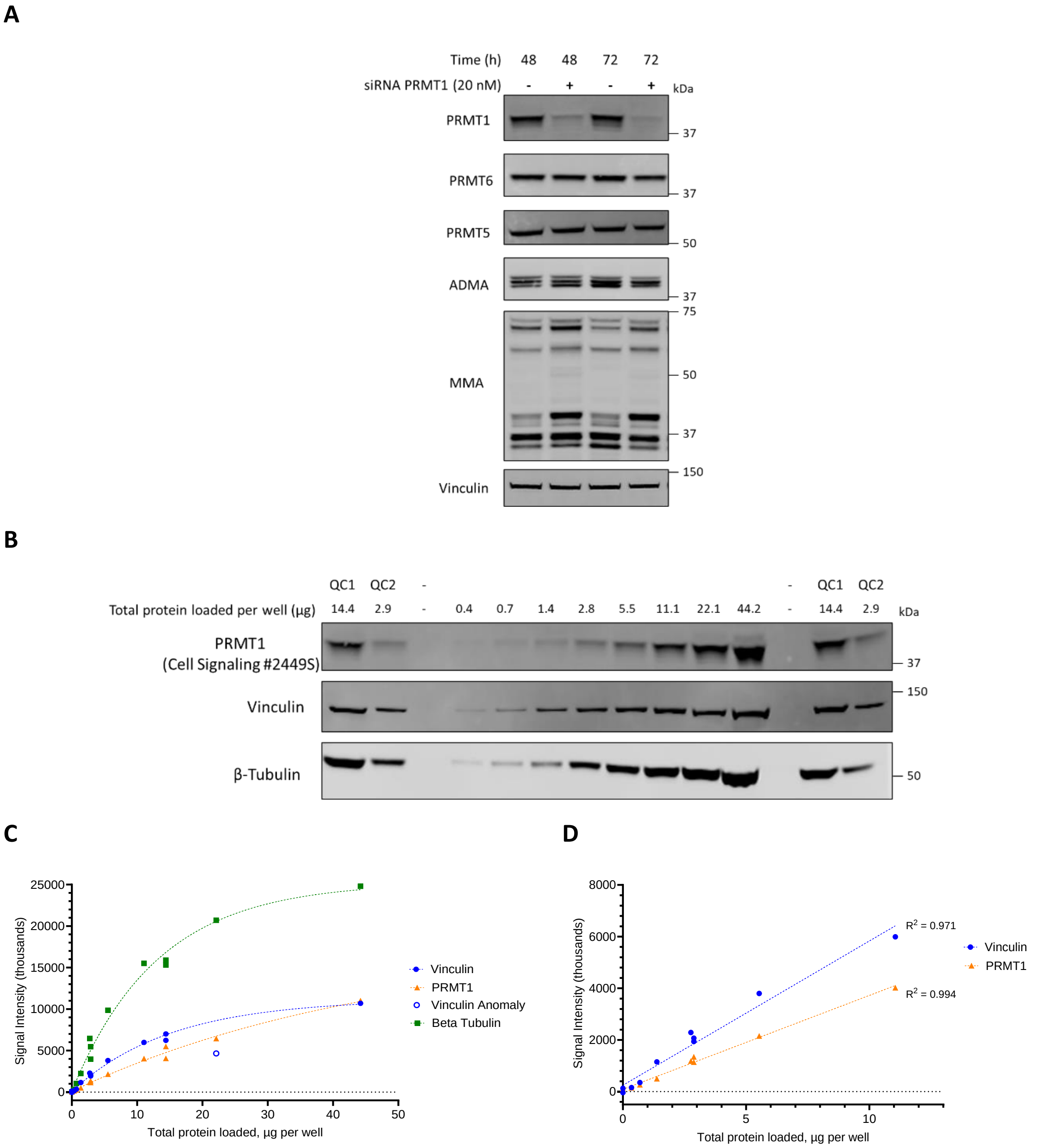

Figure S4. Sensitive and Selective Western blot Method. A) MCF-7 cells were treated with PRMT1 siRNA or non-targeting control siRNA (nt siRNA) for 48 or 72 h and then the cells were harvested for SDS-PAGE. Image representative of two independent experiments. B) A dilution series of the cell-lysate derived from MCF-7 cells was analysed by Western blot. C) The protein bands were quantified with Image Studio analysis software and plotted against the amount of total protein loaded to the well. D) The determined linear range for the semi-quantitative measurement of PRMT1 and vinculin levels by Western blot.

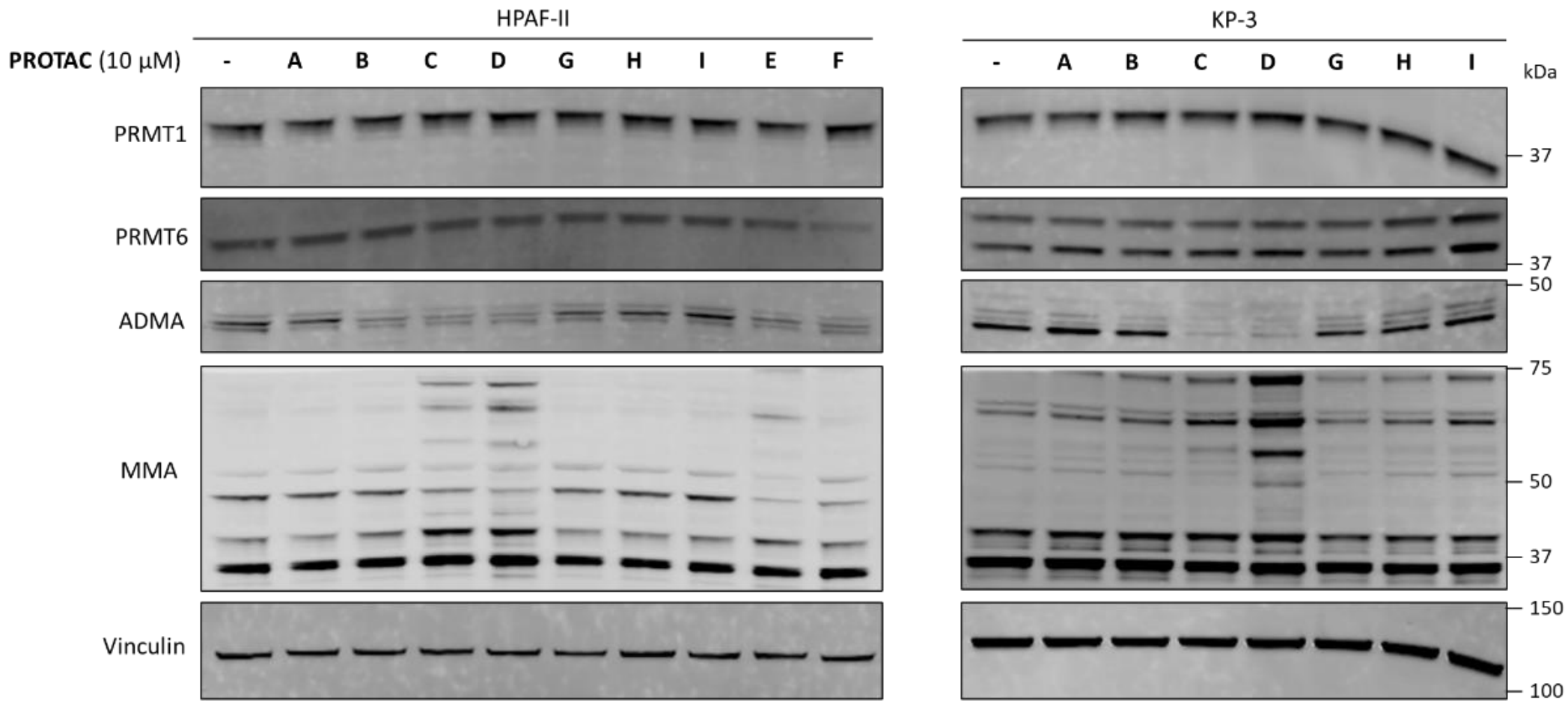

Figure S5. VHL-recruiting PROTACs in two PDAC-derived cell lines. Western blot of **PROTAC A-F** in two PDAC-derived cell lines at 10 µM. HPAF-II and KP-3 cells were treated with the indicated compounds at 10 µM for 24 h and then harvested for analysis by Western blot**. PROTAC E** and **PROTAC F** were cytotoxic in the KP-3 line.

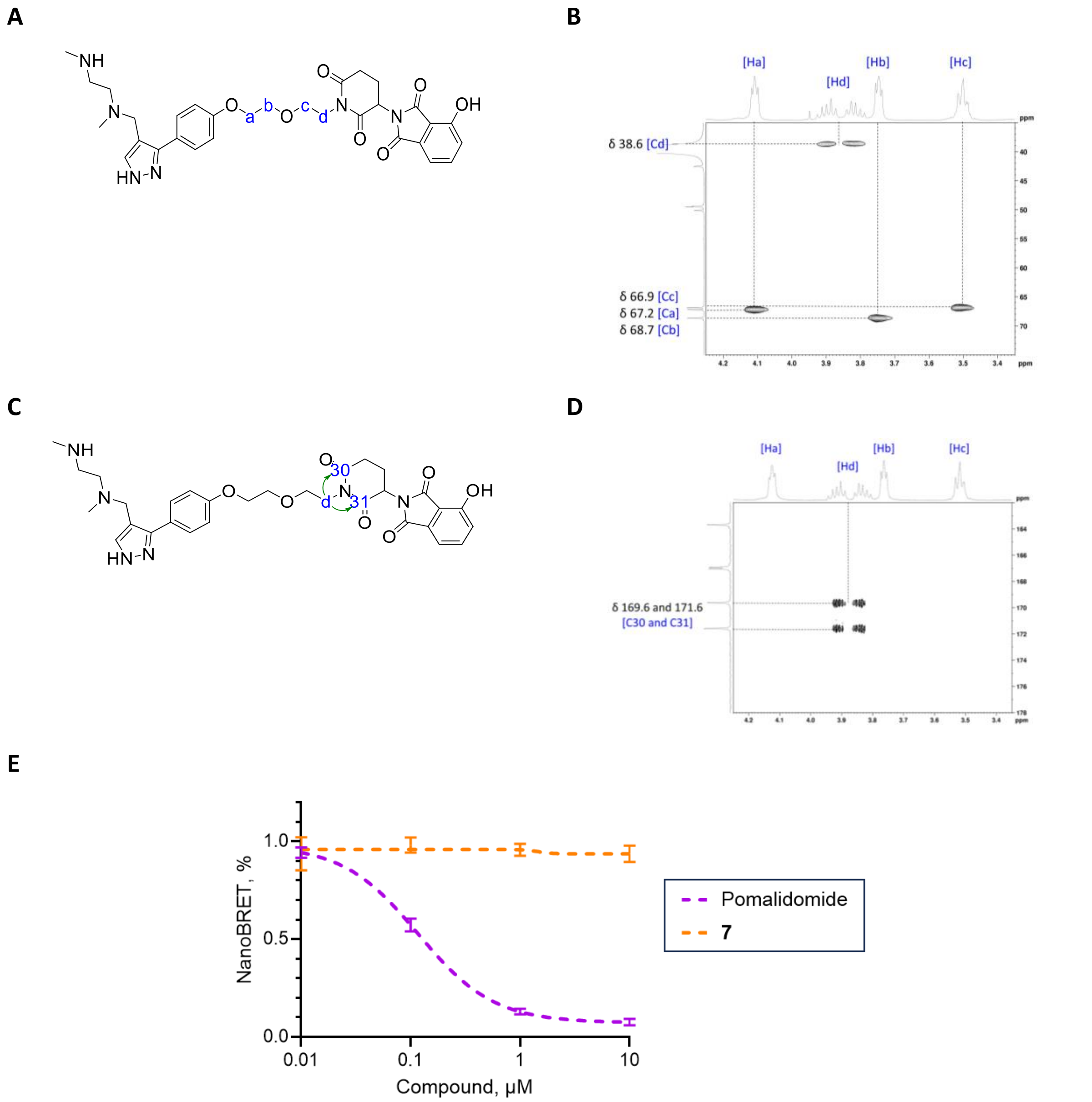

Figure S6. Heterobifunctional molecule **7** does not bind to CRBN**.** A-D) 2D NMR evidence for N-alkylation. A) Structure of **7**. B) ^{1}H-^{13}C HSQC correlations of the PEG linker. The peak for the carbon [Cd] appears at a low chemical shift. C) Structure of **7** with selected ^{3}J couplings shown by green arrow. D) HMBC correlations from protons [Hd] of the PEG linker to the carbonyls of the glutarimide ring [C30 and C31]. E) **7** does not bind to the E3-ligase CRBN. CRBN NanoBRET TE assay for CRBN. Data is normalised to untreated cells and BRET calculated by BRET emission intensity / NLuc emission intensity. The mean and standard error of two independent experiments with multiple biological replicates are shown.

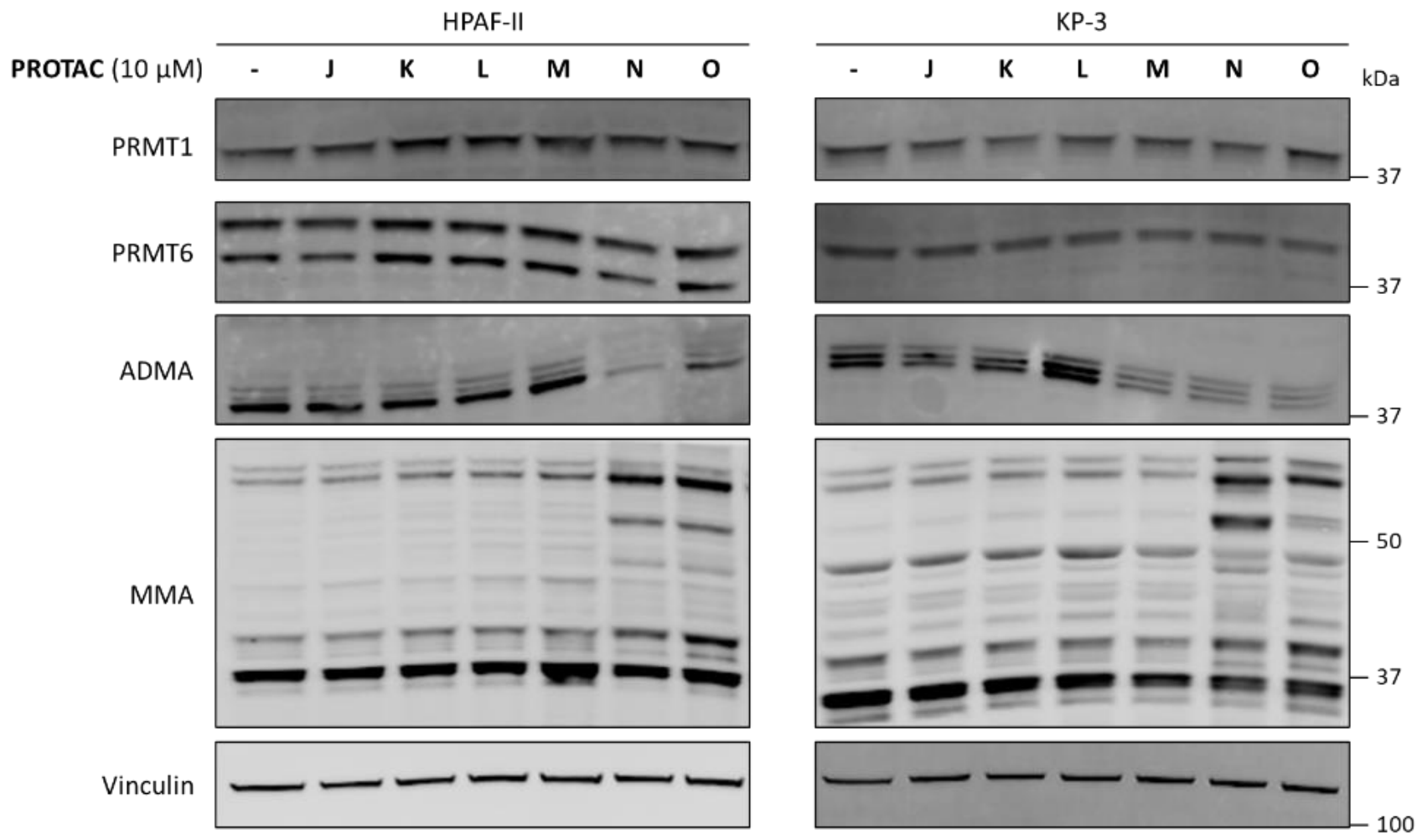

Figure S7. CRBN-recruiting PROTACs in two PDAC-derived cell lines. Western blot of **PROTAC J-O** in two PDAC-derived cell lines at 10 µM. HPAF-II and KP-3 cells were treated with the indicated compounds at 10 µM for 24 h and then harvested for analysis by Western blot.

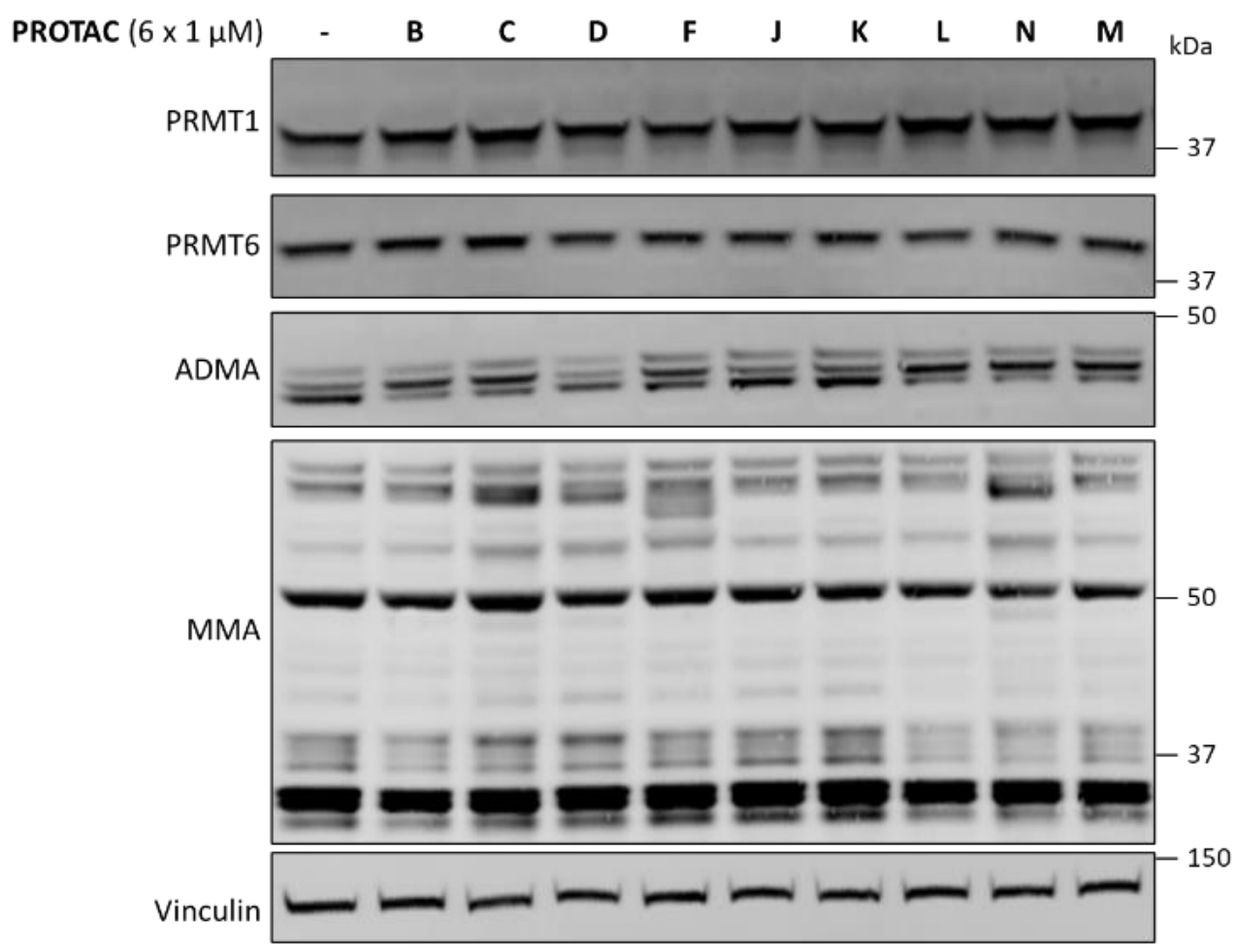

Figure S8. Selected PROTACs in the MCF-7 cell line with 6 h treatment time. MCF-7 cells were treated with 1µM of the indicated PROTAC and one-hour later an additional 1 µM of the indicated compound was added. This was repeated 4 further times (the total concentration added was 6 µM, and the total incubation time was 6 hr). The cells were then harvested for analysis by Western blot.

2. Supplementary Tables

Table S1. Published half-life data for PRMT1 in dividing mammalian cells

Cell line	Origin of cell line	Half-life, h	Method	Ref
MLE12	Mouse lung epithelial	4	Western Blot with **CHX**	[32]
HEK293 transfected with empty pLenti	Human embryonic kidney	5	Western blot with **CHX**	[34]
U2OS	Human osteosarcoma	>8*	Proteomics with **CHX**	[58]
HEK293	Human embryonic kidney	>8*	Proteomics with **CHX**	[58]
HCT116	Human colorectal carcinoma	>8*	Proteomics with **CHX**	[58]
RPE1	Human retinal pigment epithelial	>8*	Proteomics with **CHX**	[58]
HEK293	Human embryonic kidney	>10**	Western blot with **CHX**	[59]
HeLa	Human uterus/cervical epithelial adenocarcinoma	44.7	SILAC-Proteomics	[60]
C2C12	Mouse myoblast	69.7	SILAC-Proteomics	[60]

*No significant change in protein level at 8 h compared to 0 h. ** persistent band, equal intensity at indicated time to the untreated control.

3. Uncropped Scans of Western Blots shown in Figure 3 and 5

Following the transfer of the proteins from the gel onto a PVDF membrane, the membrane was cut at the 75 kDa marker. The top section was analysed by Western blot for vinculin, and the bottom section for the indicated protein/post-translational modification.

Figure 3 A

Lane 2-8 shown in figure.

PRMT1

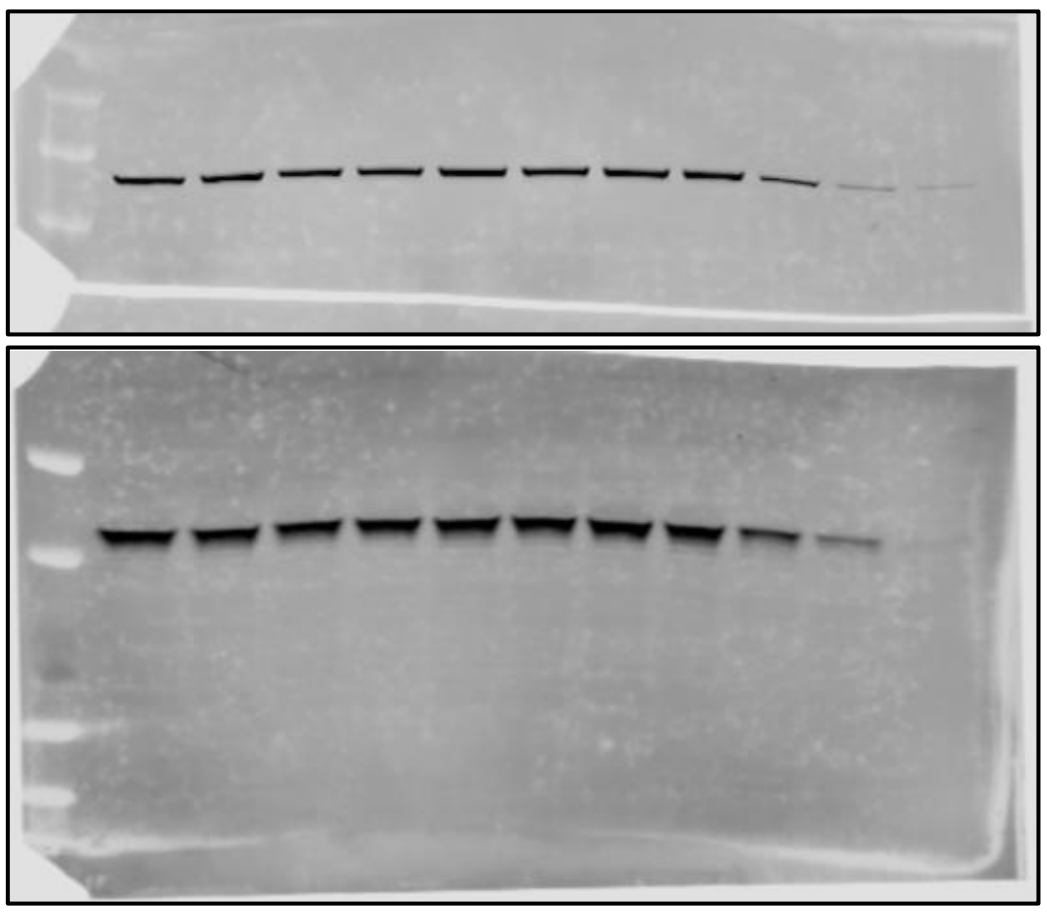

PRMT6

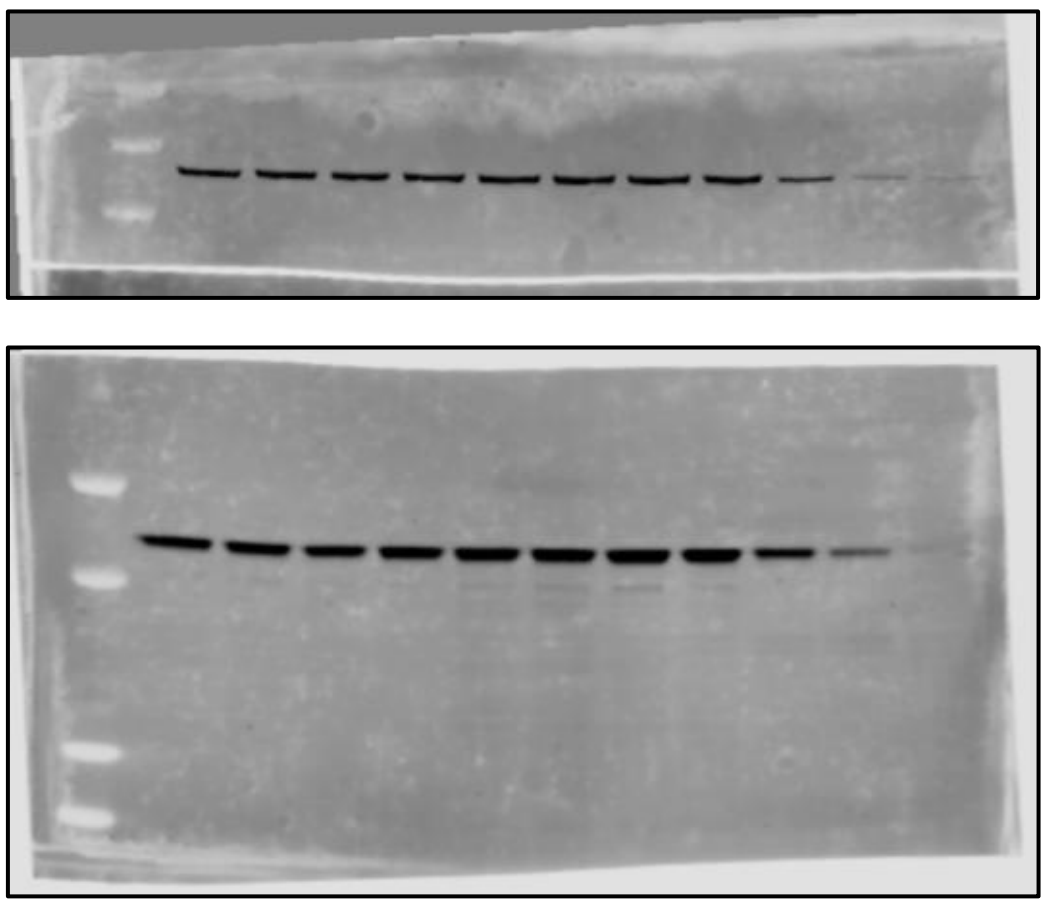

ADMA

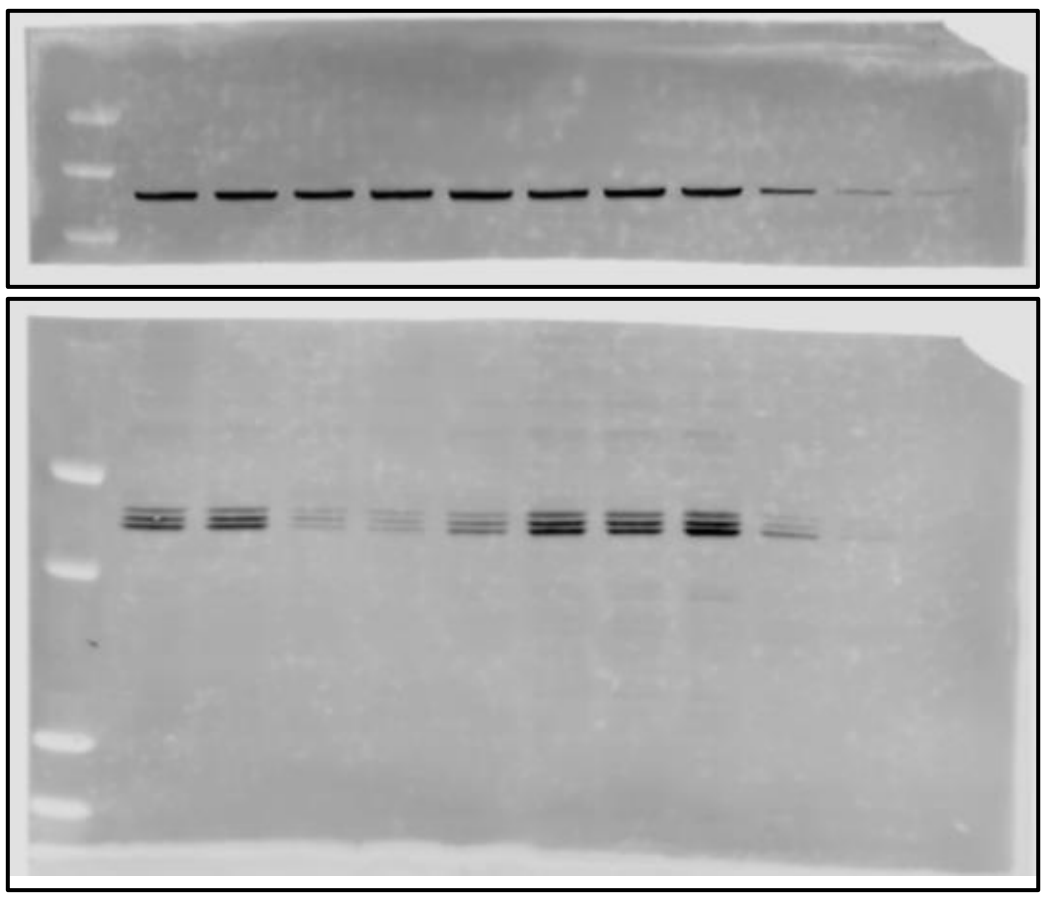

MMA

PRMT1 membrane reprobed for MMA

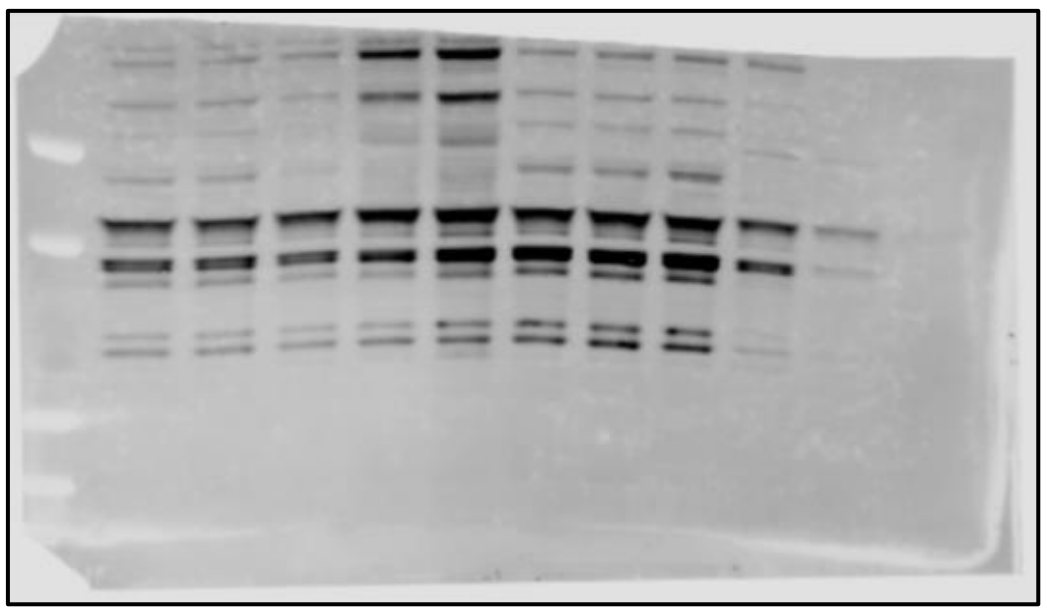

Figure 3 B

Lane 2-12 shown in figure.

PRMT1

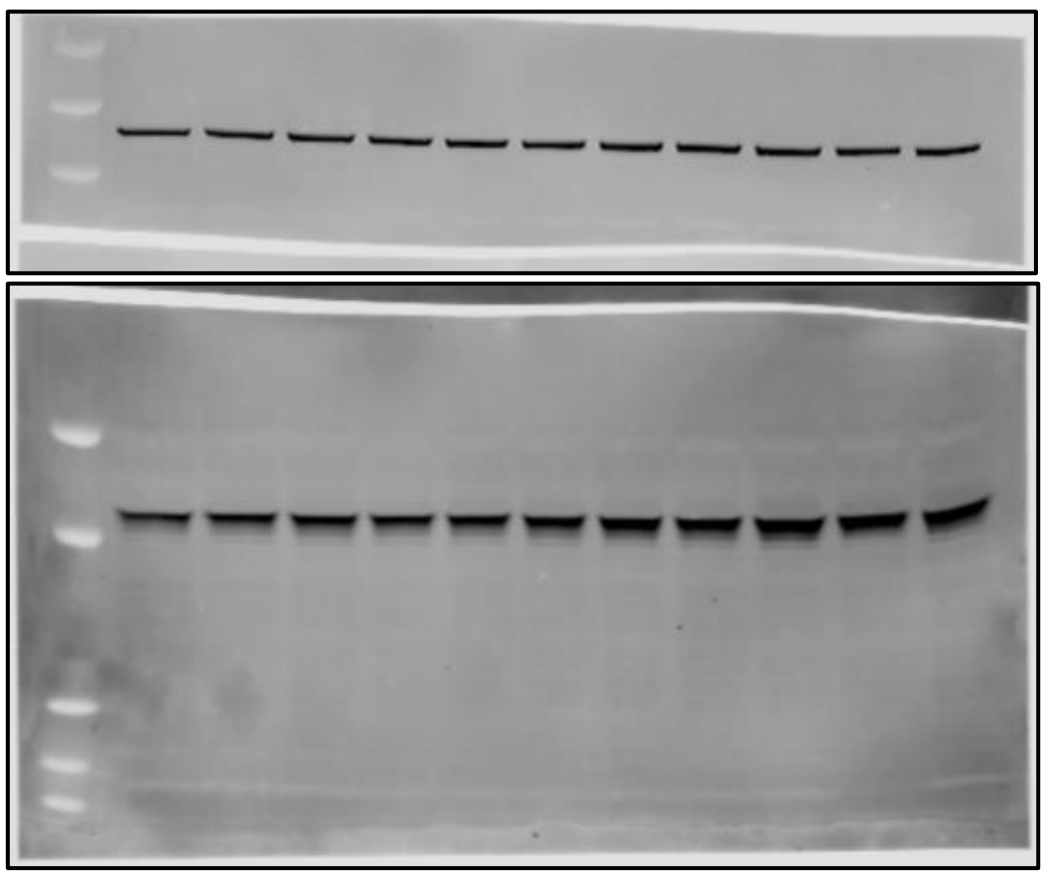

PRMT6

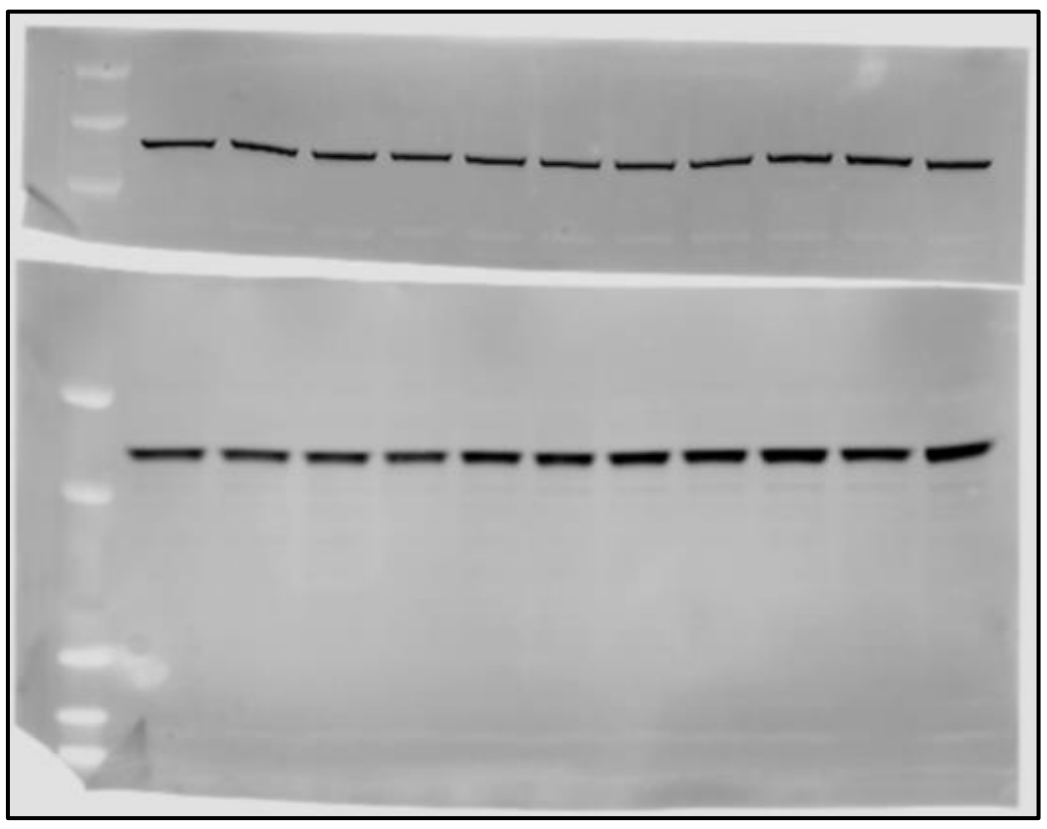

ADMA

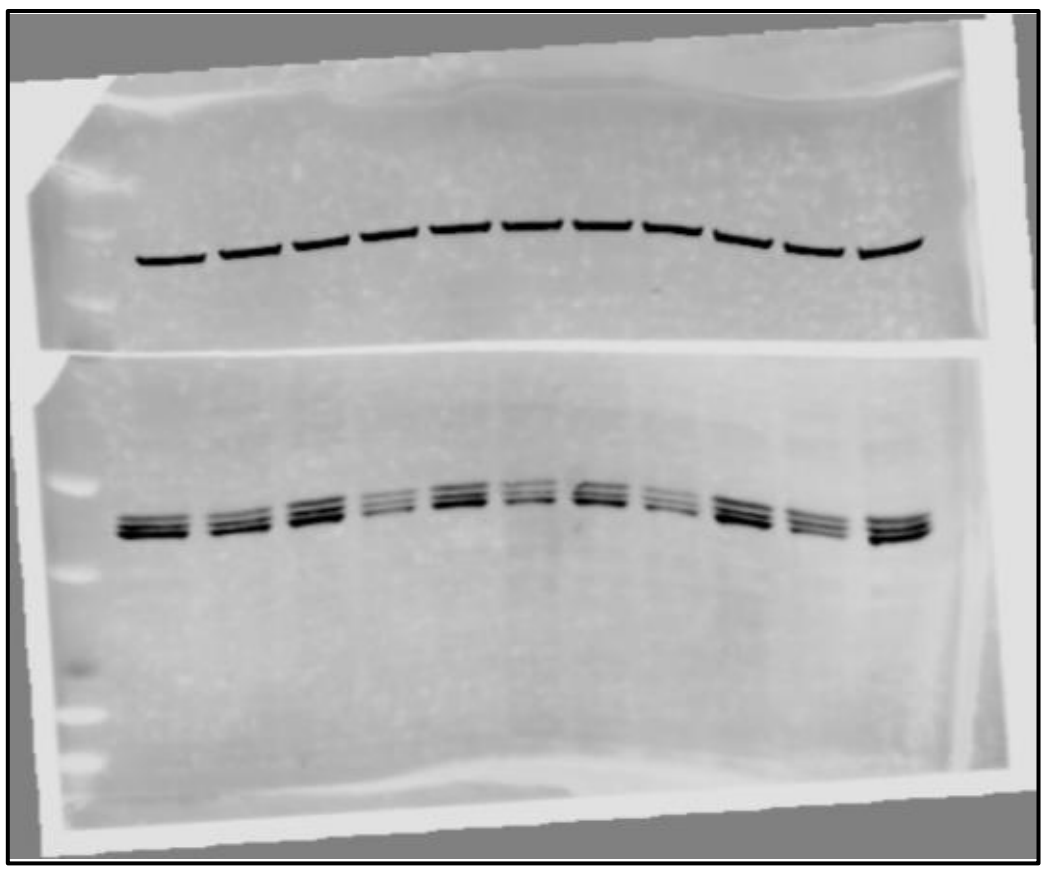

MMA

PRMT1 membrane reprobed for MMA

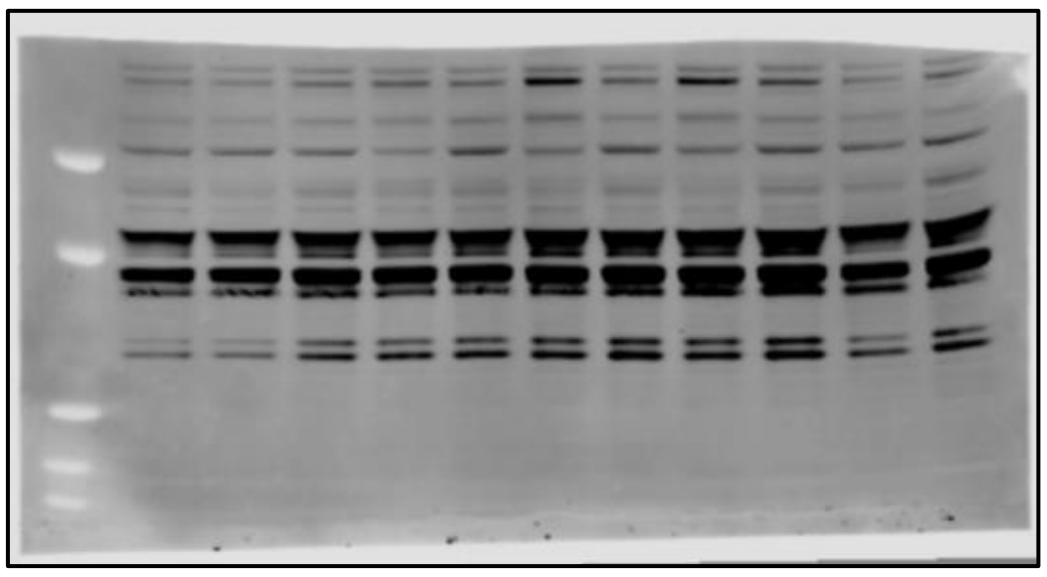

Figure 5 A

Lane 2-8 shown in figure.

PRMT1

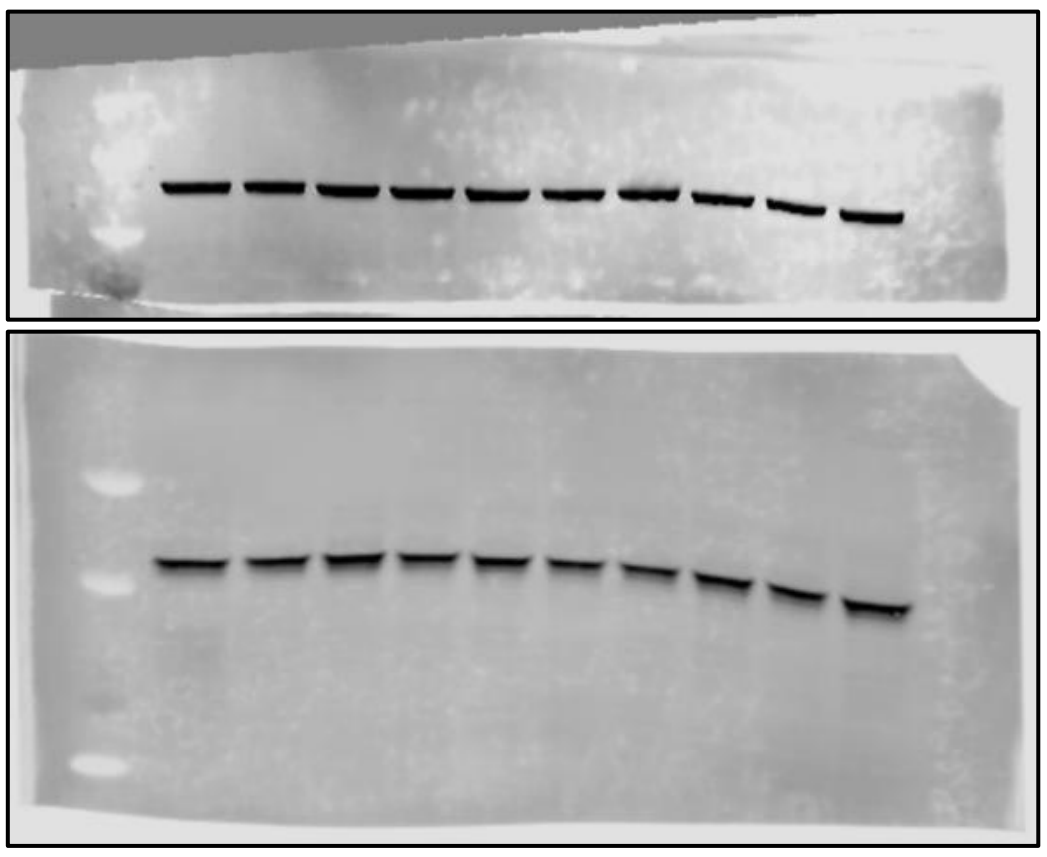

PRMT6

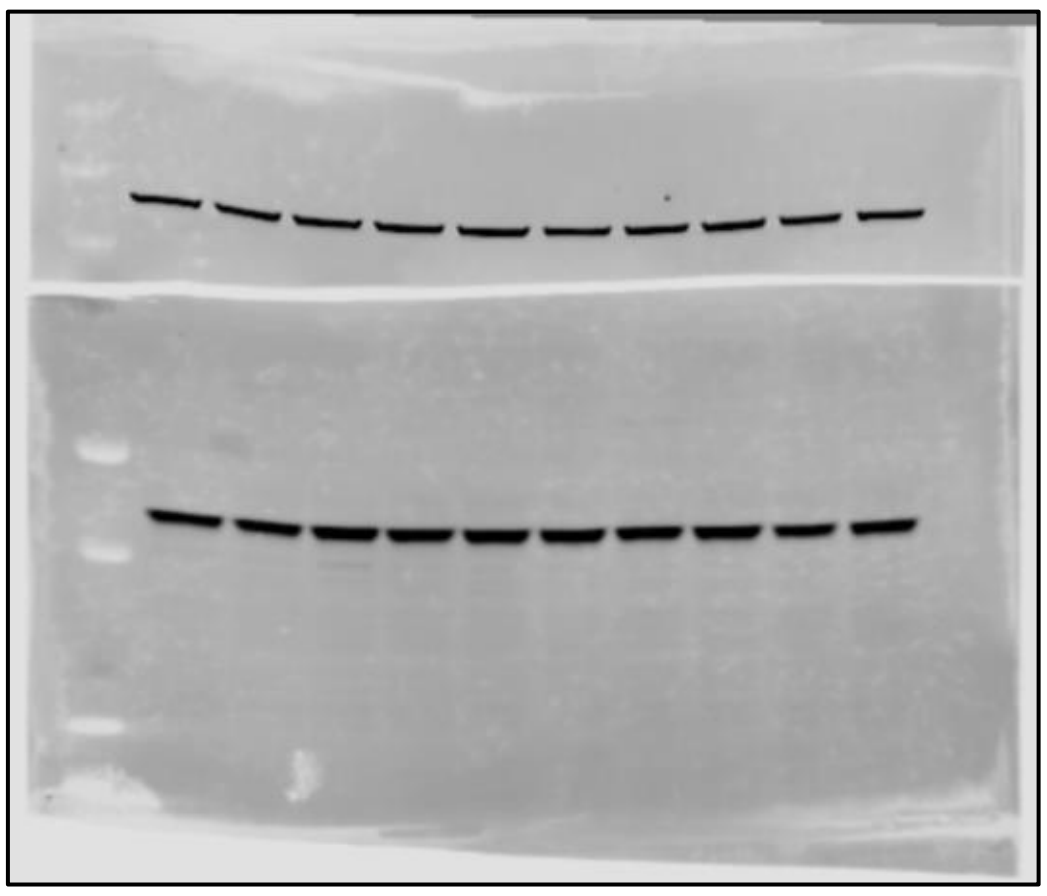

ADMA

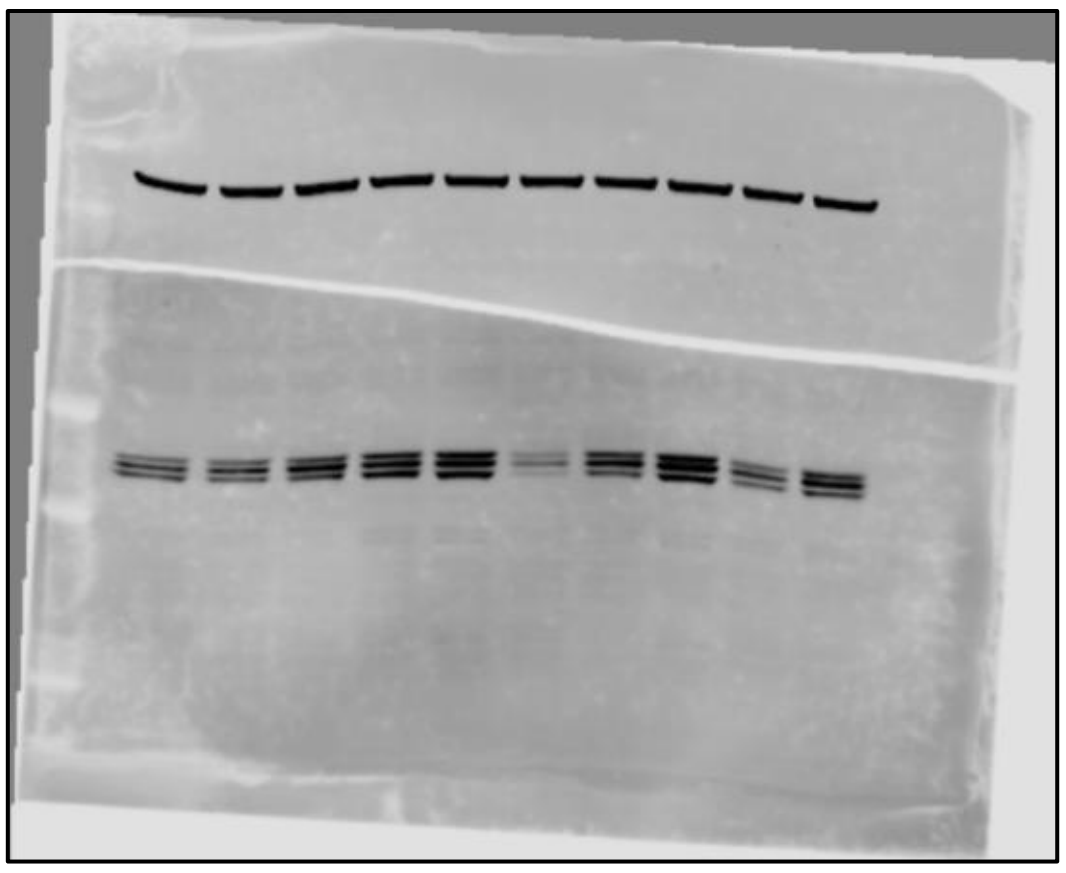

MMA

PRMT1 membrane reprobed for MMA

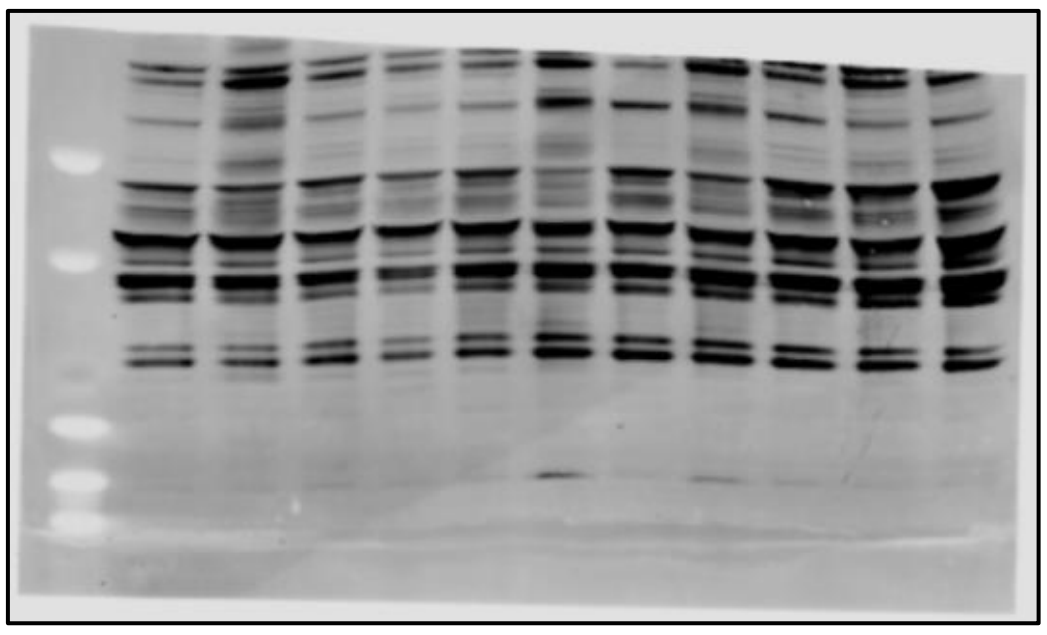

Figure 5 B

Lane 2-4 shown in figure.

PRMT1

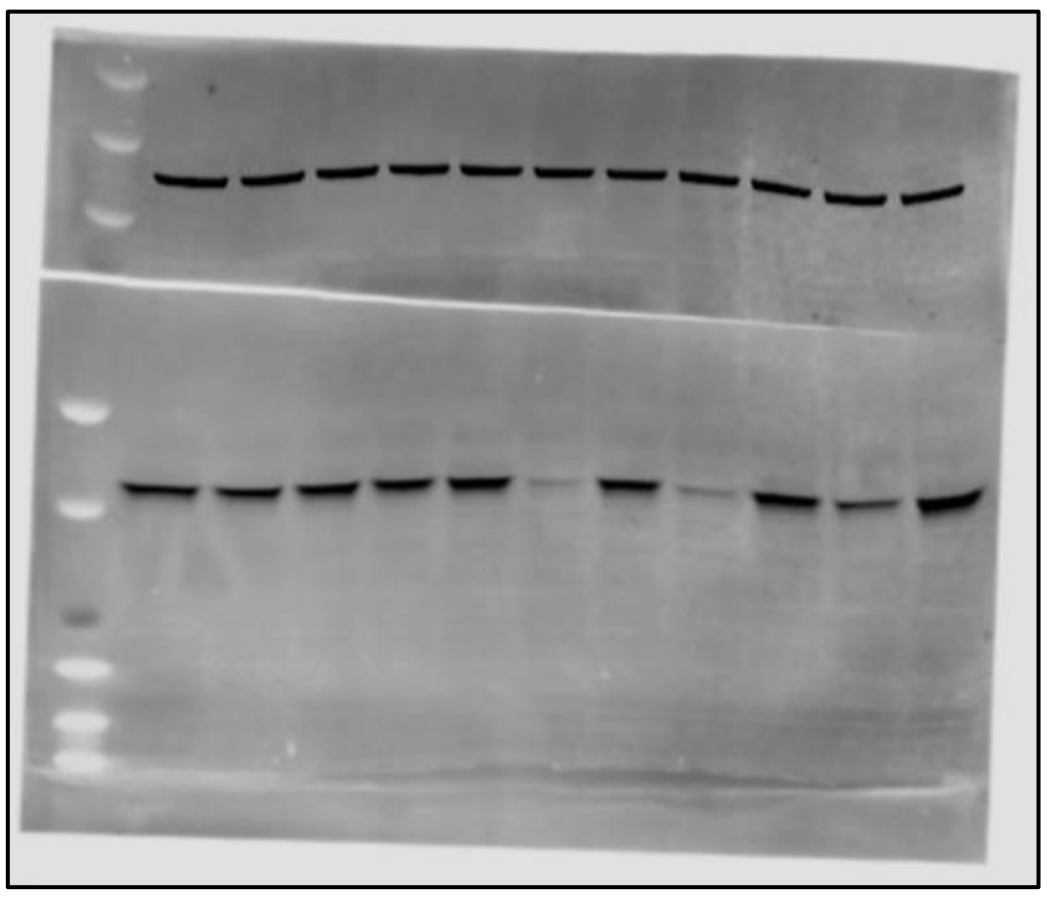

PRMT6

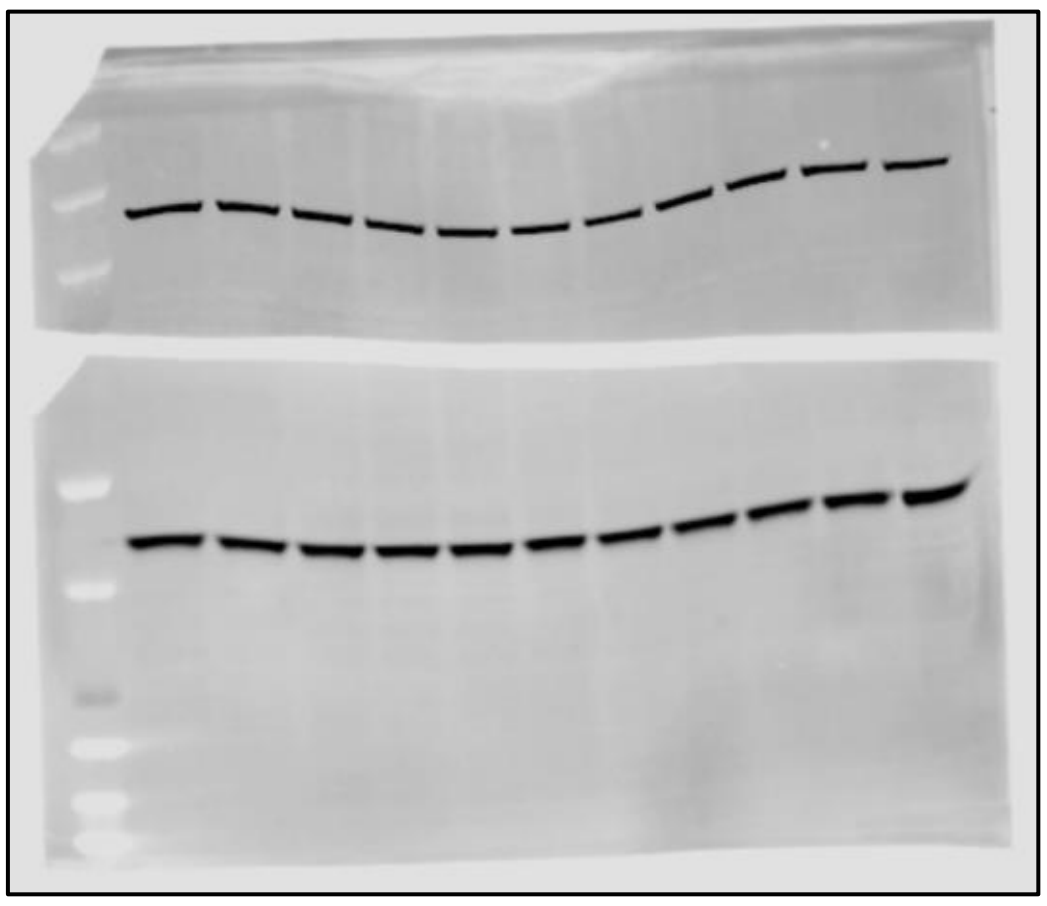

ADMA

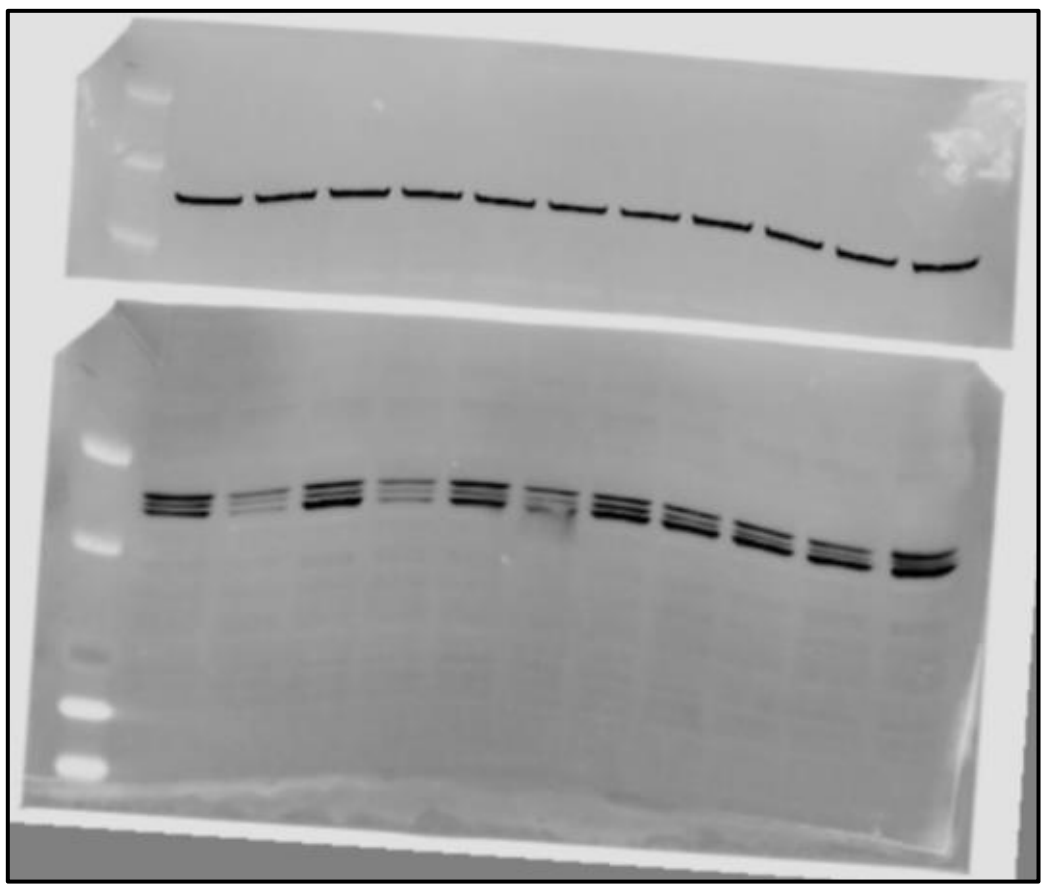

MMA

PRMT1 membrane reprobed for MMA.

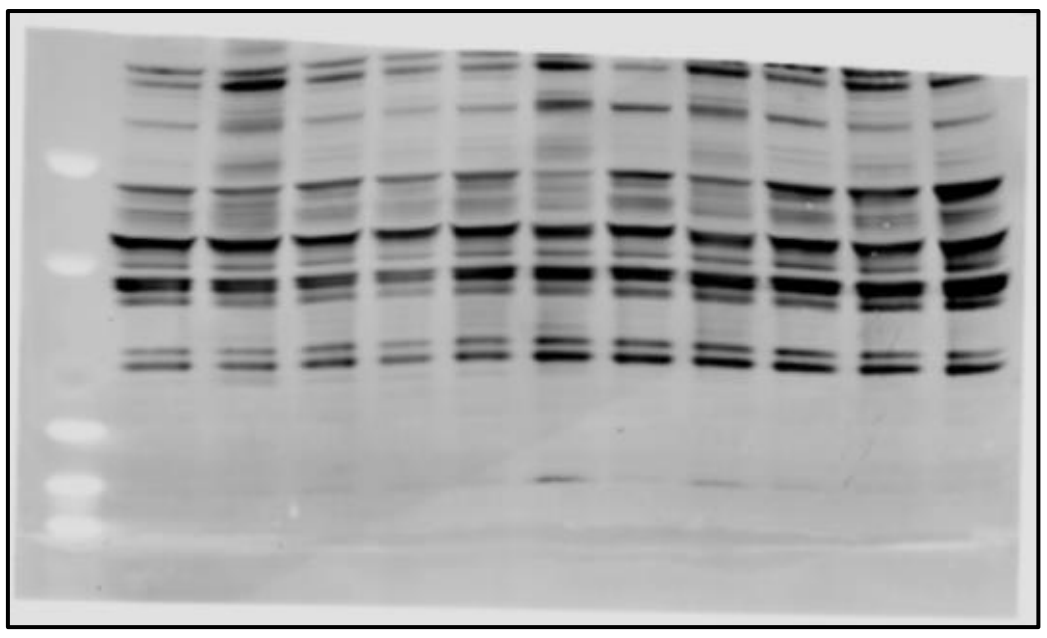

4. Experimental: Cell-based Assays

All *in-vitro* experiments were undertaken at Cancer Research UK Cambridge Institute, University of Cambridge.

Cell Lines The MCF-7 and HPAF-II cell lines were obtained from the American Type Culture Collection (ATCC). The KP-3 cell line was obtained from the Japanese Cell Repository. MCF-7 cells were cultured in Dulbecco's Modified Eagle Medium (DMEM) (gibco, 41966-029) supplemented with 10% Fetal Bovine Serum (FBS) (gibco, A3840402). HPAF-II cells cultured in in Minimum Essential Medium (MEM) (gibco, 11095-080) supplemented with 1% sodium pyruvate (Thermo Scientific, 11360-039) and 10% FBS. KP-3 cells cultured in Roswell Park Memorial Institute (RPMI) 1640 Medium (Gibco, 21875-034) supplemented with 1% sodium pyruvate and 10% FBS. Cells were maintained for no more than 50 passages at 37°C with 5% CO_2.

Western Blot Cells were counted using the Vi-CELL XR cell viability analyser, as per manufactures instructions, and the desired number of cells seeded in full media in individual 10 cm plates (Corning, #430167). The cells were allowed to adhere overnight. The cells were treated with the PROTAC in DMSO and the final DMSO concentration in all experiments was 0.01%. Following treatment, the cells were incubated at 37°C in 5% CO_2 for the indicated time. Cells were washed twice with ice cold PBS. RIPA buffer containing protease and phosphatase inhibitor was added and the cells were detached from the surface with a cell scraper. After centrifugation (8000 rpm for 3 min at 4°C), the supernatant was discarded and the pellet flash frozen and stored at -80°C or taken forward for lysis. The pellet was resuspended in RIPA buffer containing protease and phosphatase inhibitor and incubated at 4°C for 10 minutes with rotation. The sample was sonicated for 3 min (30 seconds on, 30 seconds off) and subjected to centrifugation (21,000 x g for 10 min at 4°C). The supernatant was transferred and the protein concentration quantified by the Direct Detect Spectrometer as per manufacturer's instructions. 30 µg of protein was denatured by heating at 80°C for 10 min in NuPAGE LDS sample buffer, NuPAGE Sample Reducing Agent and made up to 25 µL with RIPA buffer (multiplied up as required). The samples were loaded onto a 4-12% Bis-Tris 1.0 mm pre-cast polyacrylamide gel (12 µL loaded onto 12 well, 10 µL loaded onto 15 well) with a molecular weight ladder. With the MOPS running buffer, voltage was applied with at 60 V for 30 minutes followed by 120 V. The voltage was stopped once the dye front reached the bottom of the gel. The proteins were transferred from the gel to a PVDF membrane using an iBlot2 gel transfer device using the pre-programmed P0 method. Following transfer, the membrane was washed sequentially in methanol, H_2O and TBS-T (0.1% Tween-20 in TBS). The membrane was blocked with 5% milk in TBS for 1 h at room temperature and then incubated with primary antibody in 1% milk in TBS-T overnight at 4 °C.

The membrane was then washed three times for 5 min with TBS-T and incubated with secondary antibody in 1% milk in TBS-T for 1 h at room temperature. The membrane was washed three times for 5 min with TBS-T washed once with TBS for 5 minutes and then imaged using the LiCor CLx or LiCor Odyssey. Protein quantification was performed by densitometry using Image Studio analysis software (LI-COR Biosciences).

**Antibodies**	Source	Catalogue #	Dilution
Rabbit anti-PRMT1	Cell Signaling	2449S	1:1000
Rabit anti-PRMT6	Cell Signaling	14641S	1:1000
Rabbit anti-PRMT5	Active Motif	31001	1:1000
Rabit anti-asymmetric dimethyl arginine (ADMA)	Cell Signaling	13522S	1:1000
Rabit anti-mono-methyl arginine (MMA)	Cell Signaling	8711S	1:1000
Rabbit anti-symmetric dimethyl arginine (SDMA)	Cell Signaling	13222S	1:1000
Rabbit anti-Vinculin	Cell Signaling	E1E9V	1:1000
Mouse anti-β-Tubulin	Cell Signaling	86298S	1:1000
IRDye 800CW donkey anti-rabbit	LiCor	926-32213	1:5000
IRDye 680RD goat anti-mouse	LiCor	926-68070	1:7500
**Reagents**			
RIPA-Buffer (Pierce)	Thermo Scientific	89900	-
Protease Inhibitor (Pierce cOmplete EDTA-free protease inhibitor tablets)	Roche	11873580001	100x
Phosphatase Inhibitor (HALT Phosphatase inhibitor cocktail)	Thermo Scientific	78427	100x
NuPAGE LDS sample buffer	Invitrogen	NP0007	4x
NuPAGE sample reducing agent	Invitrogen		10x
Molecular weight ladder (Precision plus protein dual colour standard)	Bio-Rad	1610374	-
4-12% Bis-Tris 1.0 mm gel (12 well)	NuPAGE	NP0322	-
4-12% Bis-Tris 1.0 mm gel (15 well)	NuPAGE	NP0323	-
MOPS running buffer	NuPAGE	NP0001	20x
iBlot 2 gel transfer device	Invitrogen	NP0004	-
PVDF membrane (iBlot 2 transfer stacks, PVDF)	Invitrogen	IB24001	-
TBS	In-house	-	-
Tween-20 (10% Tween 20)	Bio-Rad	1610781	100x
Milk (skimmed milk powder)	Marvel Original	-	-

CRBN NanoBRET Target Engagement Assay The CRBN NanoBRET TE assay for live-cells was performed according to the manufacture's instruction (Promega, #N2910) with some modifications. MCF-7 cells were transiently transfected with the NanoLuc-CRBN fusion vector (Promega) and Lipofectamine 2000 (Invitrogen, 116680919). 24 h after transfection, the cells were resuspended in DMEM without phenol red (Gibco, 31053-028) at a density of 2 × 10^5 cells per mL and were plated into 96-well plates (Corning, #3474). The cells were incubated with 0.5 µM CRBN NanoBRET tracer (Promega) and the probe compound for 2 h at 37 °C with 5% CO_2. After 2 h incubation, the samples were transferred to a 96 well plate (Corning, #3610) with a white back seal. 3X Nano-Glo substrate (Promega) with NanoLuc extracellular inhibitor (Promega) were added to cells. After 3 minutes at room temperature, BRET was measured using a CLARIOstar PLUS microplate reader equipped with a 450-80 nm band pass filter for donor emission and a 605/30-nm band pass filter for acceptor emission. The BRET ratio was calculated with the equation and the BRET was plotted as a percentage of the DMSO-treated cells:

$$BRET = \left[\frac{Acceptor_{Sample}}{Donor_{Sample}} - \frac{Acceptor_{no\ tracer\ control}}{Donor_{no\ tracer\ control}}\right]$$

Data was processed using GraphPad Prism Version 10.0.3 and the best-fit IC_{50} values of each compound were calculated using the [inhibitor] vs response (variable slope) (four parameters) function.

siRNA MCF-7 cells were plated in a 10 cm and grown to 50-60% confluence. Cells were transfected with siRNA targeting PRMT1 or negative control siRNA (Horizon Discovery) at a final concentration of 20 nM in the presence of Lipofectamine RNAi MAX transfection Reagent (Invitrogen, #13778-150).

5. Experimental: Chemistry

5.1 General Experimental

All chemistry experiments were undertaken at the Yusuf Hamied Department of Chemistry, University of Cambridge. All non-aqueous reactions were conducted under a stream of dry nitrogen using oven-dried glassware. Temperatures of 0°C were maintained using an ice-water bath. All temperatures below -10°C were maintained using an acetone-cardice bath. Room temperature (rt) refers to ambient temperature. Reactions performed under microwave irradiation were performed in sealed vials using a Biotage Initiator+ microwave synthesiser.

Solvents and Reagents Solvent and reagents were used as received from commercial sources unless otherwise stated. Anhydrous dimethylformamide (DMF), dimethyl sulfoxide (DMSO) and 1,4-dioxane were purchased from commercial sources and used without further purification. Ethyl acetate (EtOAc), methanol (MeOH), dichloromethane (DCM) and acetonitrile were distilled from calcium hydride. Tetrahydrofuran was dried using sodium wire and distilled from a mixture of lithium aluminium hydride and calcium hydride with triphenylmethane as an indicator. Petroleum ether (PE) was distilled before use and refers to the fraction between 40–60 °C. All other solvents and reagents were obtained from commercial suppliers and used without further purification.

Purification Flash column chromatography was carried out as detailed in Still *et al.*[61] using Merck 9385 Kieselgel 60 SiO_2 (230–400 mesh) under a positive pressure of air. Automated flash column chromatography was carried out on a Combiflash Rf200 automated chromatography system with Redisep reverse-phase C18-silica flash columns (20–40 µm, 5.5/15/50 g). Preparative HPLC was carried out using an Agilent 1260 Infinity with a reversed-phase Supelcosil™ ABZ+PLUS column (C18, 250 mm × 21.2 mm, 5 µm, PN:54885) or Agilent Prep Agilent Prep 100Å (C18, 250 x 21.2 mm, 10 µm, PN: 410910-702). A linear gradient system was used (solvent A: 0.1% TFA in H_2O, solvent B: 0.05% TFA in MeCN) at a flow rate of 20 mL min^{-1}. HPLC was monitored by UV absorbance at 220 and 254 nm.

Analytical HPLC High pressure Liquid chromatography (HPLC) retention times (R_t) are reported from an Agilent 120 or 12600 Infinity system with a reversed-phase Agilent ZORBAX Eclipse XDB-C18 (150 x 4.6 mm, 5 µm, PN:993967-902) or a Supelcosil™ ABZ+PLUS column (150 x 4.6mm, 3 µm PN:59194) eluting with a linear gradient system (solvent A: 0.05% TFA in H_2O, solvent B: 0.05% TFA in MeCN) over 15 minutes at a flow rate of 1 mL min^{-1}. HPLC was monitored by UV absorbance at 220 and 254 nm.

NMR Nuclear magnetic resonance spectra were recorded at ambient temperature on a Bruker DPX-400 (400 MHz, 101 MHz), Bruker Advance 400 QNP (400 MHz, 101 MHz), Bruker Avance 500 Cryo Ultrashield (500 MHz, 126 MHz) and Bruker Advance 700 TXO Cryo Ultrashield (700 MHz). For proton NMR, ^{1}H NMR chemical shifts (δH) are reported in parts per million (ppm), to the nearest 0.01 ppm, and are referenced to the residual non-deuterated solvent peak ($CDCl_3$: 7.26, DMSO-d_6: 2.50). Coupling constants (J) are reported in Hertz (Hz) to the nearest 0.1 Hz. Data are reported as follows: chemical shift, multiplicity (s = singlet; d = doublet; t = triplet; q = quartet, m = multiplet, br=broad peak) or as a combination of these), coupling constant(s), integration and assignment. For carbon NMR, ^{13}C Chemical shifts (δC) are quoted in ppm, to the nearest 0.1 ppm, and are referenced to the residual non-deuterated solvent peak ($CDCl_3$: 77.16, DMSO-d_6: 39.52). ^{1}H NMR and ^{13}C NMR spectra assignments were supported by DEPT-135, COSY (2D, ^{1}H-^{1}H correlations), HSQC (2D, one bond ^{1}H-^{13}C correlations), HMBC (2D, multiple bond ^{1}H-^{13}C correlations) and NOESY/ROESY (2D, ^{1}H-^{1}H correlations) where appropriate. The numbering of molecules used for ^{13}C and ^{1}H NMR assignments does not conform to IUPAC standards. Spectra were processed using TopSpin v.4.1.3 (Bruker).

HRMS High resolution mass spectrometry (HRMS) measurements were recorded with an Agilent 6230 time-of-flight LC/MS with the Agilent 1260 Infinity II Prime LC system using Electrospray ionisation (ESI) techniques.

5.2 PROTAC Stability in Cell Culture Media

MCF-7 cell media (DMEM supplemented with 10% BFS), Caffeine (400 µM in DMSO) and PROTAC (400 µM in DMSO) was incubated at 37°C in an ambient atmosphere. A 10-50 µL aliquot of the mixture was taken at the indicated time point and an equal volume of ice-cold MeCN was immediately added. The sample was subjected to vortex mixing for 10 seconds and then centrifugation for 3 minutes at 13 G. The supernatants were analysed by analytical HPLC (5-60% or 5-40% MeCN (0.05% TFA) in H_2O (0.05% TFA) over 15 min). The same method was followed for stability studies in human serum (Sigma Aldrich, #H4522) and PBS pH 7.4 (in-house). 'Compound remaining, %' was determined by the integral of the respective peak for caffeine and PROTAC in the UV-HPLC chromatogram (λ = 254 nm). The integral for the PROTAC peak was divided by that of the caffeine peak and the ratio normalised to 0 h incubation. Data was processed using GraphPad Prism Version 10.0.3.

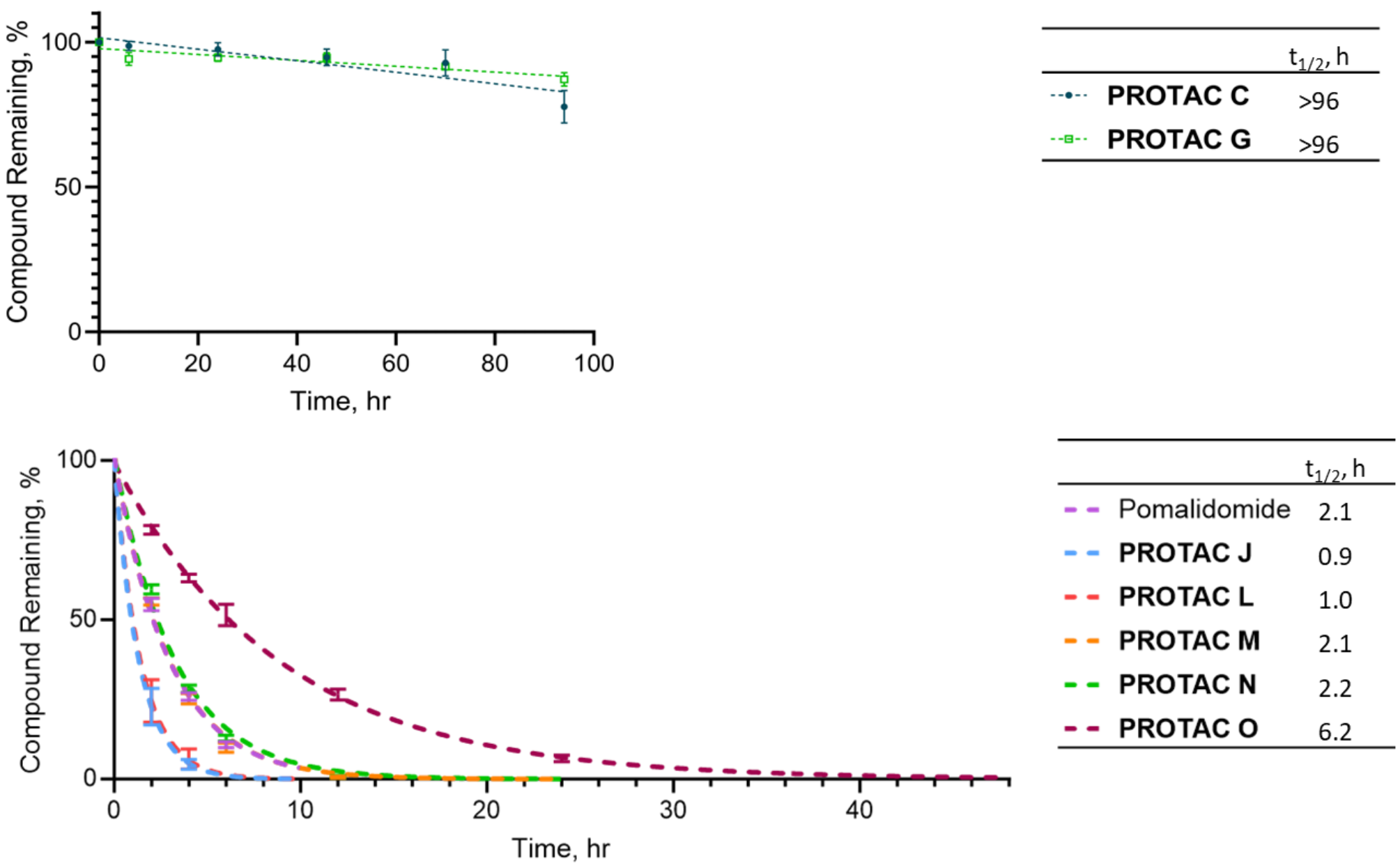

PROTAC Stability in cell culture media. The Y-axis is the percentage of PROTAC remaining compared to the peak area ratio of PROTAC-to-caffeine at 0 h. Data from three independent experiments. Mean and standard error plotted. The data was fitted with an exponential decay function with the plateau constrained to zero.

5.3 Synthesis

5.3.1 VHL-Recruiting PROTACs

General method A VHL ligand **1** (1 eq) was dissolved in DCM (5 mL/mmol) and TFA (5 mL/mmol) was added and the mixture stirred at room temperature for 30 minutes. The solvent was removed *in vacuo*. To remove excess TFA, the residue was coevaporated with MeOH (x2) and toluene (x2). The deprotected amine and the respective linker (1.2 eq) were dissolved in DMF (1 mL/mmol) and EtOAc (5 mL/mmol). DIPEA (3.2 eq) was added dropwise and the mixture was stirred for 10 minutes at room temperature. HATU (1.2 eq) was added and the mixture was stirred at room temperature for 2 min. DMF (1 mL/mmol) was then added and the solution stirred for 2 h (alkyl chloride linker) or 30 min (alkyl bromide linker). The solvent was then removed *in vacuo*.

General Method B Alkyl halide (1 eq), PRMT1 ligand **4** (1 eq), K_2CO_3 (2 eq) and KI (0.2 eq) were dissolved in MeCN (15 mL/mmol) and heated at 80°C. The solvent was removed *in vacuo* and the residue dissolved in MeOH (20 mL/mmol) and $HCl_{(aq, 37\%)}$ (20 mL/mmol) was added dropwise and the mixture stirred at room temperature for 1 hr. The solvent was then removed *in vacuo*.

(2*S*,4*R*)-1-((*S*)-2-(2-chloroacetamido)-3,3-dimethylbutanoyl)-4-hydroxy-*N*-(4-(4-methylthiazol-5-yl)benzyl)pyrrolidine-2-carboxamide, (3a).

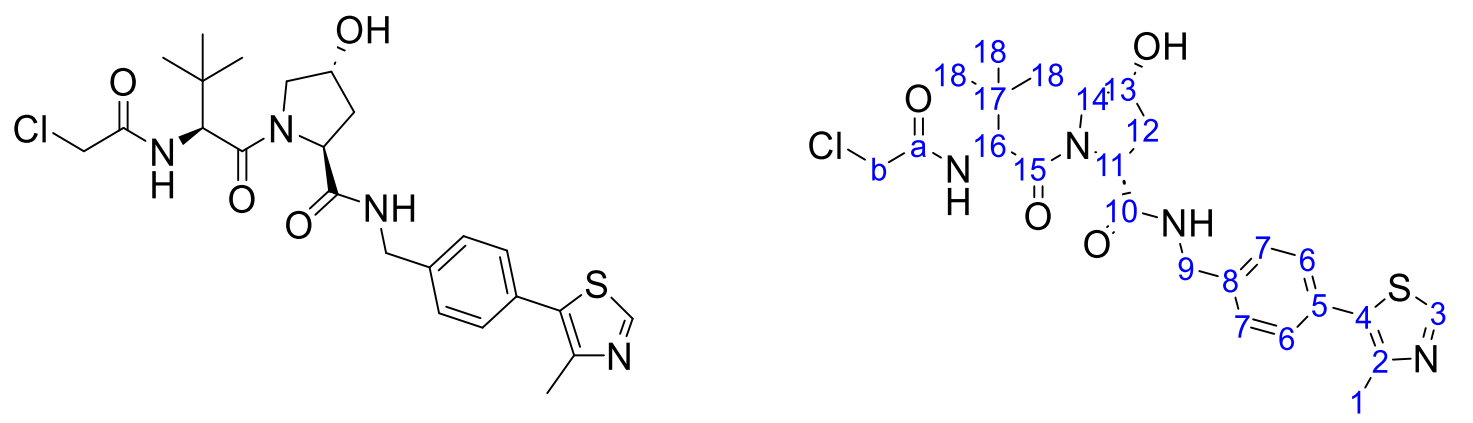

1 (150 mg, 0.28 mmol, 1 eq) was dissolved in DCM (1.4 mL) and TFA (1.4 mL) was added and the resulting mixture stirred at room temperature for 30 minutes. The solvent was removed *in vacuo*. To remove excess TFA, the residue was coevaporated with MeOH (x2) and toluene (x2). The residue was dissolved in DCM (3 mL) and triethyl amine (200 µL, 1.4 mmol, 5 eq) was added. The reaction was cooled to 0°C and chloroacetyl chloride (45 µL, 0.6 mmol, 2 eq) was added and the reaction stirred overnight at room temperature. The reaction was diluted with water and extracted with DCM (x3). The combined organic layers were washed with water, dried with Na_2SO_4 and the solvent removed *in vacuo*. The residue was purified by silica gel chromatography (0-5% MeOH in DCM) to yield the title compound as an orange oil (75 mg, 52%).**^{1}H NMR (500 MHz, d$_6$-DMSO)** δ 8.98 (s, 1H, H3), 8.60 (t, *J*=6.0 Hz, 1H, 9-NH), 8.29 (d, *J*=9.3 Hz, 1H, 16-NH), 7.43-7.38 (m, 4H, H6 and H7), 5.16 (d, *J*=3.5 Hz, 1H, 13-OH), 4.54 (d, *J*=9.4 Hz, 1H, H16), 4.43 (m, 2H, H9), 4.36 (m, 1H, H13), 4.23-4.15 (m, 3H, H11 and Hb), 3.68-3.61 (m, 2H, H14), 2.44 (s, 3H, H1), 2.08-2.01 and 1.92-1.88 (m, 2H, H12). 0.95 (s, 9H, H18). **^{13}C NMR (125 MHz, d$_6$-DMSO)** δ 171.8 (C10), 168.9 (C15), 165.7 (Ca), 151.6 (C3), 147.7 (C2), 139.5 and 131.2 and 129.5 (C4, C5 and C8), 128.6 and 127.4 (C6 and C7), 68.9 and 58.7 (C9 and C13), 56.8 and 56.6 (C16 and C14), 42.5 and 41.7 (C11 and Cb), 37.9 (C17) 35.7 (C12), 26.2 (C18), 15.9 (C1). **HRMS (ESI)** *m/z* calcd for $C_{24}H_{31}ClN_4O_4S$ $[M+H]^+$: 507.1827; found 507.1827.

(2*S*,4*R*)-1-((*S*)-2-(6-chlorohexanamido)-3,3-dimethylbutanoyl)-4-hydroxy-*N*-(4-(4-methylthiazol-5-yl)benzyl)pyrrolidine-2-carboxamide,(3b).

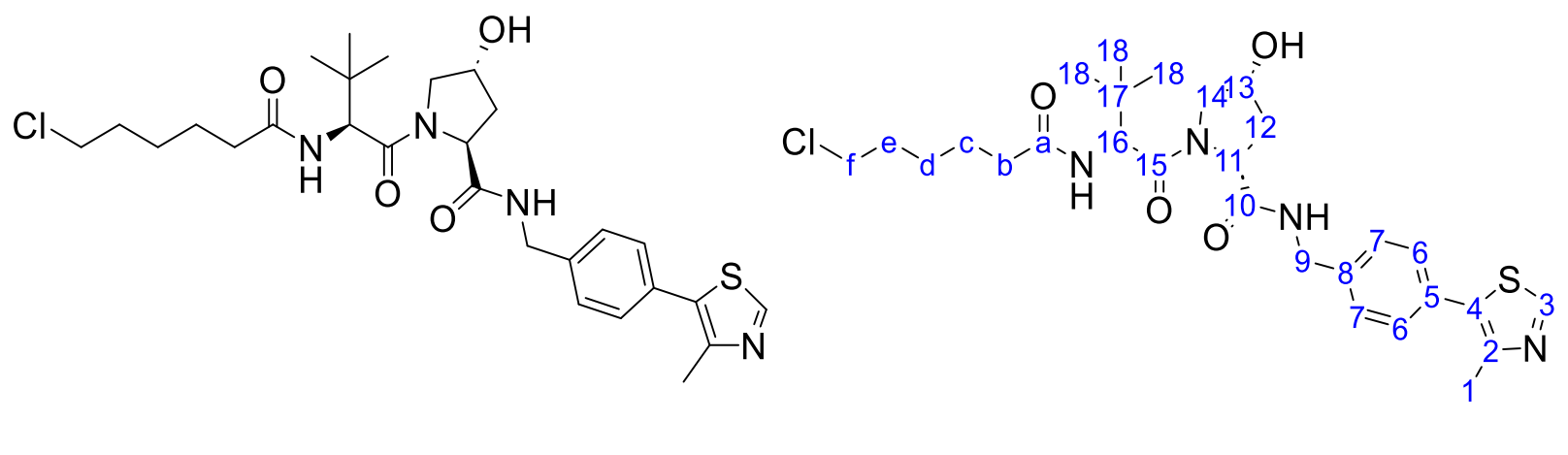

General method A was followed with VHL ligand **1** (150 mg, 0.28 mmol) and the linker 6-chlorohexanoic acid. The residue was then purified by silica gel chromatography (0-5% MeOH in DCM) and the product dissolved in DCM and washed with saturated $Na_2HCO_{3(aq)}$, washed with water, dried with Na_2SO_4 and the solvent removed *in vacuo* to give the title compound as a white solid (161 mg, quantitative). **^{1}H NMR (500 MHz, d$_6$-DMSO)** δ 8.98 (s, 1H, H3), 8.56 (t, *J*=6.0 Hz, 1H, 9-NH), 7.88

(d, *J*=7.9 Hz, 1H, 16-N<u>H</u>), 7.40 (m, 4H, H6 and H7), 5.12 (d, *J*=3.5 Hz, 1H, 13-O<u>H</u>), 4.54 (d, *J*=9.4 Hz, 1H, H16), 4.46-4.34 (m, 3H, H9 and H13), 4.22 (dd, *J*=15.9 and 5.5 Hz, 1H, H11), 3.69-3.64 (m, 2H, H14), 3.61 (m, 2H, Hf), 2.44 (s, 3H, H1), 2.30-2.23 and 2.16-2.09 (m, 2H, Hb), 2.05-2.01 and 1.92-1.87 (m, 2H, H12), 1.74-1.67 (m, 2H, He), 1.56-1.32 (m, 4H, Hc and Hd), 0.93 (s, 9H, H18). **^{13}C NMR (125 MHz, $CDCl_3$)** δ 173.5 (Ca), 172.0 (C15), 170.9 (C10), 150.5 (C3), 148.6 (C2), 138.2 and 131.7 and 131.1 (C4, C5 and C8), 129.7 and 128.2 (C6 and C7), 70.1 (C13 or C16), 58.7 (C9), 57.6 (C13 or C16), 56.8 (C14), 44.9 (Cf), 43.4 (C11), 36.3 and 36.0 (Cb and C12), 35.1 (C17), 32.3 (Ce), 26.5 and 26.5 (C18 and Cd) 24.9 (Cc), 16.2 (C1). **HRMS (ESI)** *m/z* calcd for $C_{28}H_{39}ClN_4O_4S$ $[M+H]^+$: 263.2453; found 263.2453.

(2*S*,4*R*)-1-((*S*)-2-(8-bromooctanamido)-3,3-dimethylbutanoyl)-4-hydroxy-*N*-(4-(4-methylthiazol-5-yl)benzyl)pyrrolidine-2-carboxamide, (3c).

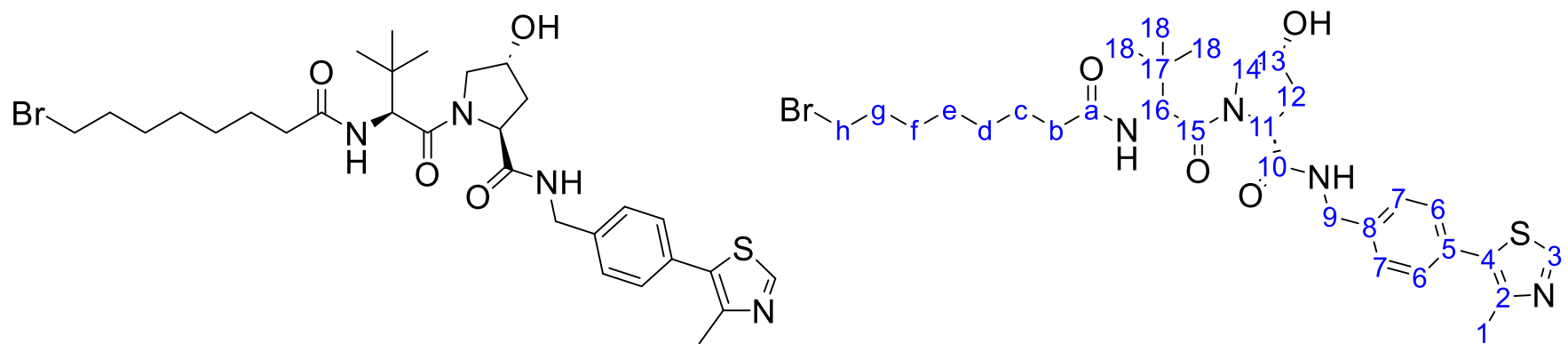

General method A was followed with VHL ligand **1** (150 mg, 0.28 mmol) and the linker 8-bromooctanoic acid. The reaction was stirred for 3 hr. The residue was then purified by silica gel chromatography (0-5% MeOH in DCM). The product was the dissolved in DCM and washed with saturated $Na_2HCO_{3(aq)}$ (x2), washed with water, dried with Na_2SO_4 and the solvent removed *in vacuo* to give a colourless solid (54 mg, 30%). **^{1}H NMR (500 MHz, d_6-DMSO)** δ 8.98 (s, 1H, H3), 8.55 (t, *J*=6.0 Hz, 1H, 9-N<u>H</u>), 7.84 (d, *J*=9.4 Hz, 1H, 16-N<u>H</u>), 7.40 (dd, *J*=9.1 and 9.0 Hz, 4H, H6 and H7), 5.12 (br s, 1H, 13-O<u>H</u>), 4.54 (d, *J*=9.4 Hz, 1H, H16), 4.46-4.34 (m, 3H, H9 and H13), 4.21 (dd, *J*=15.8 and 5.5 Hz, 1H, H11), 3.69-3.62 (m, 2H, H14), 3.51 (t, *J*=6.7, 2H, Hh), 2.44 (s, 3H, H1), 2.29-2.08 (m, 2H, Hb), 2.05-2.01 and 1.92-1.87 (m, 2H, H12), 1.80-1.74 (m, 2H, Hg), 1.54-1.43 (m, 2H, Hc), 1.39-1.23 (m, 6H, Hd-f), 0.93 (s, 9H, H18). **^{13}C NMR (125 MHz, d_6-DMSO)** δ 172.0 (Ca), 171.9 (C15), 169.7 (C10), 151.4 (C3), 147.7 (C2), 139.5 and 131.2 and 129.6 (C4, C5 and C8), 128.6 and 127.4 (C6 and C7), 68.8 (C13 or C16), 58.7 (C9), 56.3 and 53.3 (C13 or C16 and C14), 41.6 (C11), 37.9 (C12), 35.20 and 35.17 and 34.8 (Cb, Ch and C17), 32.2 (Cg), 28.4-27.4 (Cd-f), 26.4 (C18), 25.3 (Cc), 15.9 (C1). **HRMS (ESI)** *m/z* calcd for $C_{30}H_{43}BrN_4O_4S$ $[M+H]^{+:}$ 635.2261 ; found 635.2269.

(2*S*,4*R*)-1-((*S*)-2-(10-bromodecanamido)-3,3-dimethylbutanoyl)-4-hydroxy-*N*-(4-(4-methylthiazol-5-yl)benzyl)pyrrolidine-2-carboxamide, (3d).

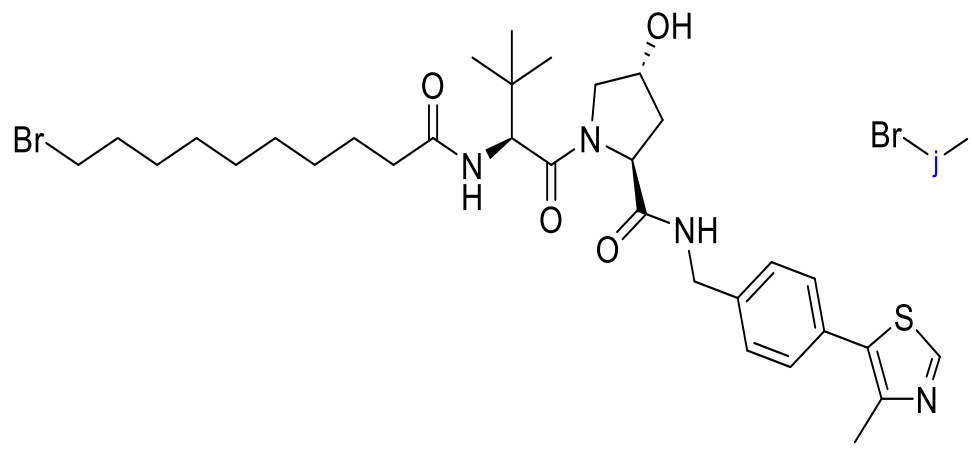

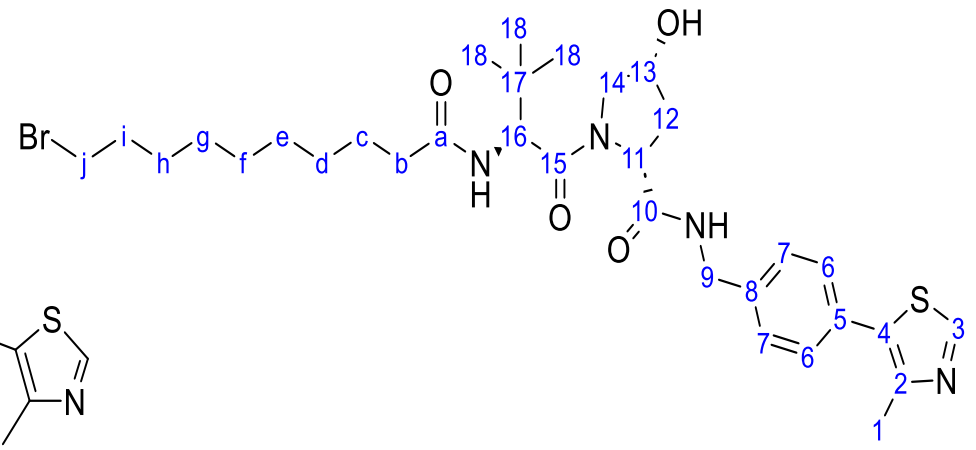

General method A was followed with VHL ligand **1** (100 mg, 0.19 mmol) and the linker 10-bromodecanoic acid. The residue was then purified by flash column chromatography (C18 Gold, 5-95% MeCN in 1% $HCOOH_{(aq)}$ over 40 CV) to give a colourless oil (98 mg, 78%). **^{1}H NMR (500 MHz, d_6-DMSO)** δ 8.98 (s, 1H, H3), 8.55 (t, *J*=6.1 Hz, 1H, 9-NH), 7.83 (d, *J*=9.4 Hz, 1H, 16-NH), 7.40 (m, 4H, H6 and H7), 5.12 (br s, 1H, 13-OH), 4.54 (d, *J*=9.4 Hz, 1H, H16), 4.43 (m, 2H, H9), 4.35 (m, 1H, H13), 4.21 (dd, *J*=15.8 and 5.5 Hz, 1H, H11), 3.66 (m, 2H, H14), 3.50 (m, 2H, Hj), 2.44 (s, 3H, H1), 2.27-2.23 and 2.13-2.21 (m, 2H, Hb), 2.05-2.00 and 1.92-1.88 (m, 2H, H12), 1.78 (m, 2H, Hi), 1.48 (m, 2H, Hc), 1.38 (m 2H, Hh), 1.25 (m, 8H, Hd-Hg), 0.93 (s, 9H, H18). **^{13}C NMR (125 MHz, d_6-DMSO)** δ 172.1 (Ca), 171.9 (C10), 169.7 (C15), 151.4 (C3), 147.7 (C2), 139.5 and 131.2 and 129.6 (C4, C5 and C8), 128.6 and 127.4 (C6 and C7), 68.9 and 58.7 (C9 and C13), 56.3 and 26.3 (C16 and C14), 41.8 (C11), 37.9 (C12), 35.21 and 35.17 (Cb and Cj), 34.9 (C17), 32.2 (Ci), 28.8-28.0 (Cd-Cg), 27.5 (Ch), 26.4 (C18), 25.4 (Cc), 15.9 (C1).**HRMS (ESI)** *m/z* calcd for $C_{32}H_{47}BrN_4O_4S$ $[M+H]^+$: 663.2574; found 223.2564.

(2*S*,4*R*)-1-((*S*)-2-(12-bromododecanamido)-3,3-dimethylbutanoyl)-4-hydroxy-*N*-(4-(4-methylthiazol-5-yl)benzyl)pyrrolidine-2-carboxamide, (3e).

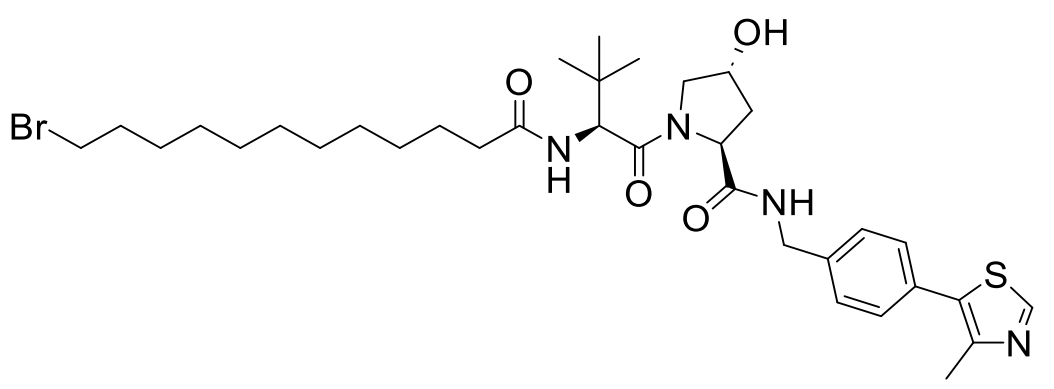

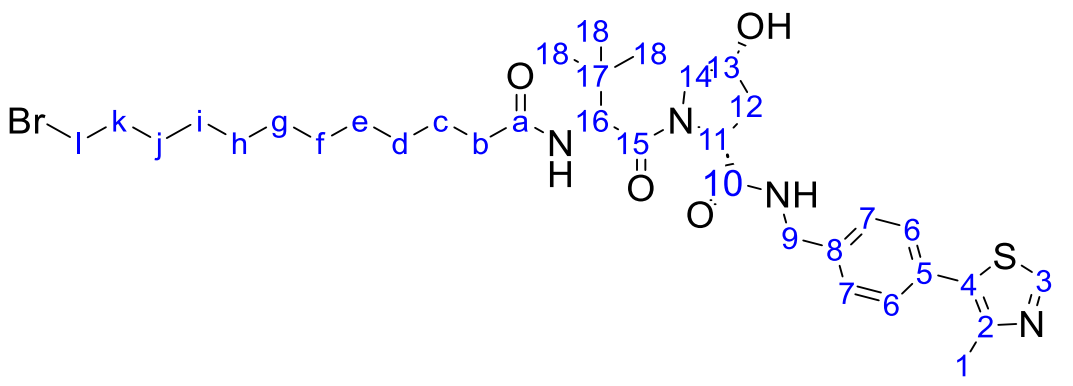

General method A was followed with VHL ligand **1** (100 mg, 0.19 mmol) and the linker 12-bromododecanoic acid. The residue was then purified by flash column chromatography (C18 Gold, 5-95% MeCN in 1% $HCOOH_{(aq)}$ over 40 CV) to give a pale yellow oil (106 mg, 81%). **^{1}H NMR (500 MHz, $CDCl_3$)** δ 8.68 (s, 1H, H3), 7.40 (t, *J*=8.0 Hz, 1H, 9-NH), 7.31 (m, 4H, H6 and H7), 6.56 (d, *J*=9.0 Hz, 1H, 16-NH), 4.63 (t, *J*=8.0 Hz, 1H, H9), 4.56-4.48 (m, 3H, H13 and H16), 4.29 (dd, *J*=15.1 and 5.3 Hz, 1H, H11), 4.02 and 3.63 (m, 2H, H14), 3.36 (m, 2H, Hl), 2.47 (s, 3H, H1), 2.37-2.31 (m, 1H, H12), 2.19-2.08 (m, 3H, H12 and Hb), 1.81 (m, 2H, Hk), 1.56-1.52 (m, 2H, Hc), 1.37 (m, 2H, Hj), 1.23 (m, 12H , Hd-Hi), 0.92 (s, 9H, H18). 13-OH not identified. **^{13}C NMR (125 MHz, $CDCl_3$)** δ 174.2 (Ca), 171.8 (C15). 171.2 (C10), 150.6 (C3), 148.2 (C2), 138.3 and 131.8 and 130.8 (C4, C5 and C8), 129.5 and 128.0 (C6 and

C7), 70.0 (C13 or C16), 59.0 (C9), 57.5 (C13 or C16), 57.0 (C14), 43.2 (C11), 36.5 and 36.4 (C12 and Cb), 35.3 (Cl), 32.8 and 32.8 (Ck and C17), 29.5-28.8 (Cd-Ci), 28.2 (Cj), 26.4 (C18), 25.7 (Cc), 15.9 (C1). **HRMS (ESI)** *m/z* calcd for $C_{34}H_{51}BrN_4O_4S$ $[M+H]^+$: 691.2887; found 691.2894.

(2*S*,4*R*)-1-((*S*)-2-(16-bromohexadecanamido)-3,3-dimethylbutanoyl)-4-hydroxy-*N*-(4-(4-methylthiazol-5-yl)benzyl)pyrrolidine-2-carboxamide, (3f).

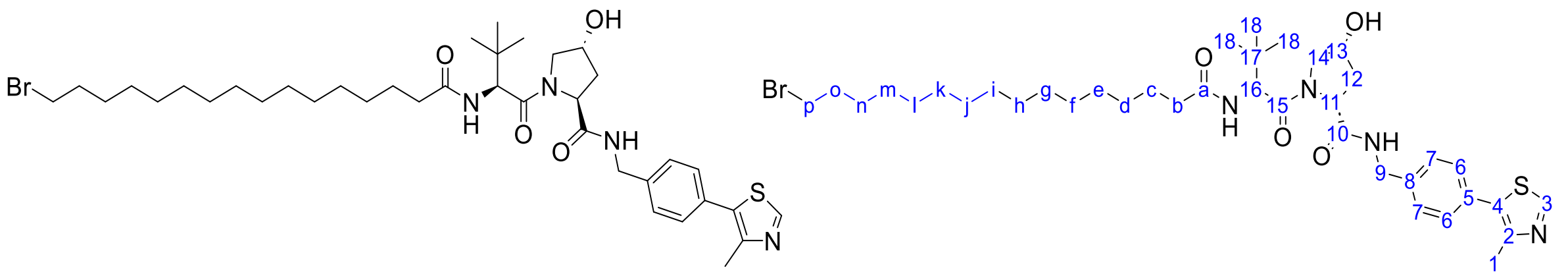

General method A was followed with VHL ligand **1** (100 mg, 0.19 mmol) and the linker 16-bromohexadecanoic acid. The residue was then purified by flash column chromatography (C18 Gold, 5-95% MeCN in 1% $HCOOH_{(aq)}$ over 40 CV) to give a yellow oil (119 mg, 84%). **^{1}H NMR (500 MHz, $CDCl_3$)** δ 8.71 (s, 1H, H3), 7.37 (t, *J*=5.9 Hz, 1H, 9-N<u>H</u>), 7.31 (m, 4H, H6 and H7), 6.56 (d, *J*=8.9 Hz, 1H, 16-N<u>H</u>), 4.68 (t, *J*=7.9 Hz, 1H, H9), 4.56-4.51 (m, 3H, H13 and H16), 4.30 (dd, *J*=15.1 and 5.3 Hz, 1H, H11), 4.04 and 3.63 (m, 2H, H14), 3.37 (m, 2H, Hp), 2.47 (s, 3H, H1),2.41 (m, 1H, H12), 2.17-2.10 (m, 3H, H12 and Hb), 1.82 (m, 2H, Ho), 1.60-1.52 (m, 2H, Hc), 1.39 (m, 2H, Hn), 1.22 (m, 20H, Hd-Hm), 0.92 (s, 9H, H18). 13-O<u>H</u> not identified. **^{13}C NMR (125 MHz, $CDCl_3$)** δ 174.2 (Ca), 171.9 (C15), 171.1 (C10), 150.7 (C3), 148.2 (C2), 138.3 and 131.9 and 130.7 (C4, C5 and C8), 129.5 and 128.1 (C6 and C7), 70.0 (C13 or C16), 59.0 (C9), 57.5 (C13 or C16), 57.0 (C14), 43.2 (C11), 36.5 and 36.4 (Cb and C12), 35.3 (Cp), 34.1 and 32.9 (Co and C17), 29.8-28.8 (Cd-Cm) 28.2 (Cn), 26.5 (C18), 25.8 (Cc), 15.9 (C1). **HRMS (ESI)** *m/z* calcd for $C_{38}H_{59}BrN_4O_4S$ $[M+H]^+$: 747.3513; found 747.3520.

(2*S*,4*R*)-1-((*S*)-2-(2-(2-chloroethoxy)acetamido)-3,3-dimethylbutanoyl)-4-hydroxy-*N*-(4-(4-methylthiazol-5-yl)benzyl)pyrrolidine-2-carboxamide, (3g).

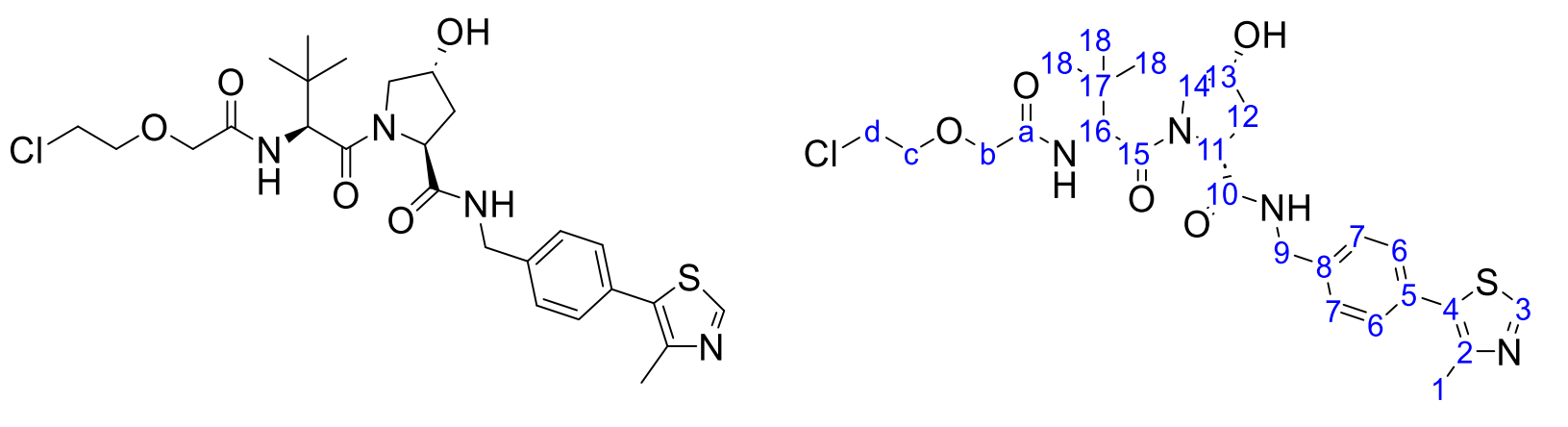

General method A followed with VHL ligand **1** (150 mg, 0.28 mmol) and linker 2-(2-chloroethoxy)ethanol. The residue was then purified by automated column chromatography (C18

Gold, 5-95% MeCN in 1% $CH_3COOH_{(aq)}$ over 40 CV) to give the title compound as a white solid (123 mg, 79%). **^{1}H NMR (500 MHz, d_6-DMSO)** δ 8.98 (s, 1H, H3), 8.61 (t, *J*=6.0 Hz, 1H, 9-N<u>H</u>), 7.50 (d, *J*=9.6 Hz, 1H, 16-N<u>H</u>), 7.40 (m, 4H, H6 and H7), 5.15 (d, *J*=3.5 Hz, 1H, 13-O<u>H</u>), 4.57 (d, *J*=9.6 Hz, 1H, H16), 4.46-4.36 (m, 3H, H9 and H13), 4.24 (dd, *J*=15.8 and 5.5 Hz, 1H, H11), 4.03 (s, 2H, Hb), 3.81-3.74 (m, 4H, Hc and Hd), 3.64 (m, 2H, H14), 2.44 (s, 3H, H1), 2.08-2.04 and 1.93-1.88 (m, 2H, H12), 0.95 (s, 9H, H18). **^{13}C NMR (125 MHz, d_6-DMSO)** δ 171.8 (C10), 169.1 (C15), 168.3 (Ca), 151.5 (C3), 147.7 (C2), 139.4 and 131.2 and 129.7 (C4, C5 and C8), 128.7 and 127.5 (C6 and C7), 70.9 (Cc), 67.3 (Cb), 68.9 and 58.8 (C9 and C13), 56.6 (C16), 43.6 (Cd), 41.6 (C11), 37.8 (C12), 35.8 (C17), 26.2 (C18), 15.9 (C1). **HRMS (ESI)** *m/z* calcd for $C_{26}H_{35}ClN_4O_5S$ $[M+H]^+$: 551.2089; found 501.2088.

(2*S*,4*R*)-1-((*S*)-2-(2-(2-(2-chloroethoxy)ethoxy)acetamido)-3,3-dimethylbutanoyl)-4-hydroxy-*N*-(4-(4-methylthiazol-5-yl)benzyl)pyrrolidine-2-carboxamide, (3h).

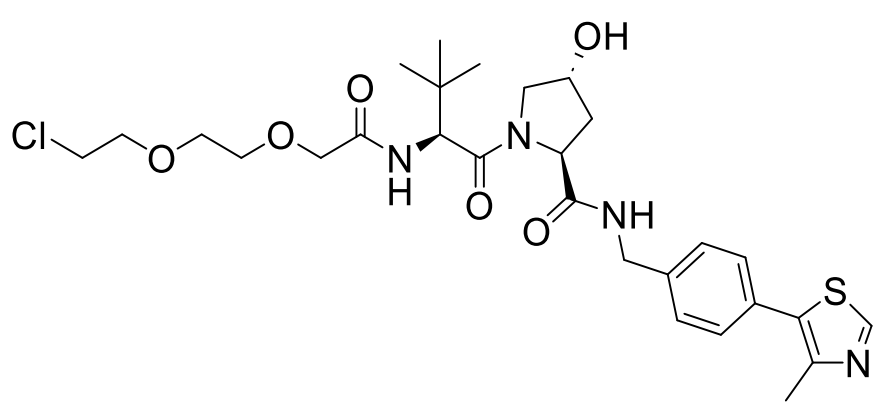

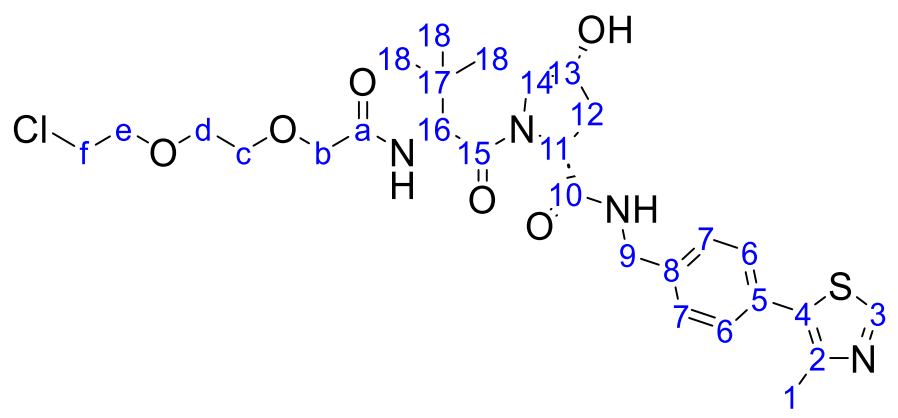

General method A followed with VHL ligand **1** (150 mg, 0.28 mmol) and linker 2-[2-(2-chloroethoxy)ethoxy]ethanol. The residue was then purified by automated column chromatography (C18 Gold, 5-95% MeCN in 1% $CH_3COOH_{(aq)}$ over 40 CV) to give the title compound as a white solid (113 mg, 67%). **^{1}H NMR (400 MHz, d_6-DMSO)** δ 8.98 (s, 1H, H3), 8.59 (t, *J*=5.9 Hz, 1H, 9-N<u>H</u>), 7.42 (m, 1H, 16-N<u>H</u>), 7.40 (m, 4H, H6 and H7), 5.15 (br s, 1H, 13-O<u>H</u>), 4.57 (d, *J*=9.6 Hz, 1H, H16), 4.46-4.36 (m, 3H, H9 and H13), 4.24 (dd, *J*=15.8 and 5.6 Hz, 1H, H11), 3.98 (s, 2H, Hb), 3.79-3.60 (m, 10H, Hc-f and H14), 2.44 (s, 3H, H1), 2.08-2.03 and 1.93-1.87 (m, 2H, H12), 0.95 (s, 9H, H18). **^{13}C NMR (101 MHz, d_6-DMSO)** δ 171.8 (C10), 169.1 (C15), 168.6 (Ca), 151.4 (C3), 147.7 (C2), 139.4 and 131.1 and 129.7 (C4, C5 and C8), 128.7 and 127.4 (C6 and C7), 70.6-69.4 (Cb-e), 68.9 and 58.7 (C9 and C13), 56.6 (C14), 55.7 (C16), 43.4 (Cf), 41.7 (C11), 37.9 (C12), 35.7 (C17), 26.1 (C18), 15.9 (C1). **HRMS (ESI)** *m/z* calcd for $C_{28}H_{39}ClN_4O_5S$ $[M+H]^+$: 595.2352; found 595.2384.

(2*S*,4*R*)-1-((*S*)-2-(*tert*-butyl)-14-chloro-4-oxo-6,9,12-trioxa-3-azatetradecanoyl)-4-hydroxy-*N*-(4-(4-methylthiazol-5-yl)benzyl)pyrrolidine-2-carboxamide, (3i).

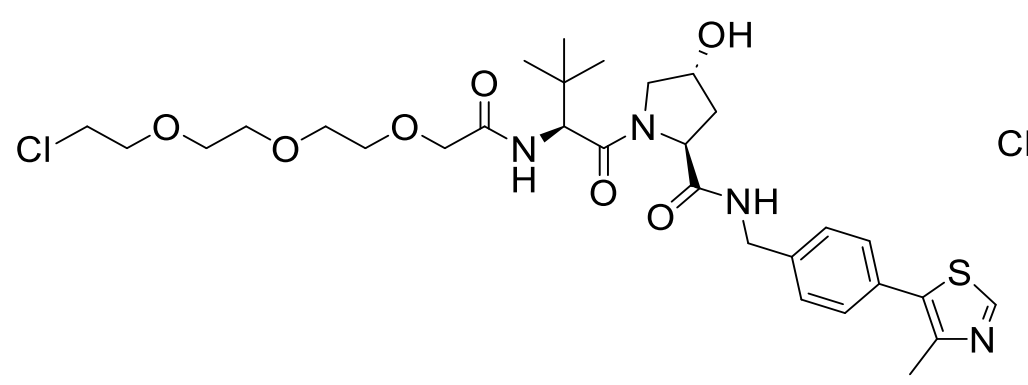

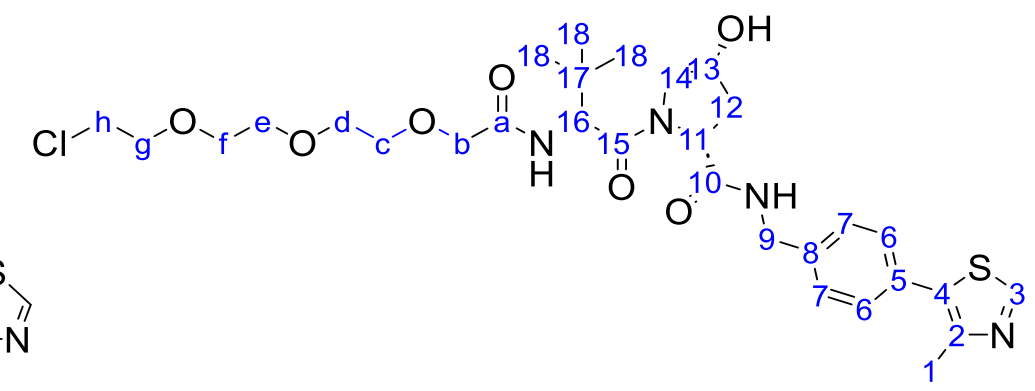

General method A followed with VHL ligand **1** (83 mg, 0.16 mmol) and linker 2-(2-(2-(2-chloroethoxy)ethoxy)ethoxy)ethanol. The residue was then purified by automated column chromatography (C18 Gold, 5-95% MeCN in 1% $CH_3COOH_{(aq)}$ over 40 CV) to give the title compound as a white solid (56 mg, 53%).**^{1}H NMR (500 MHz, d$_6$-DMSO)** δ 8.98 (s, 1H, H3), 8.61 (m, 1H, 9-NH), 7.40 (m, 5H, 16-NH and H6 and H7), 5.16 (br s, 1H, 13-OH), 4.57 (d, *J*=9.6 Hz, 1H, H16), 4.45-4.35 (m, 3H, H9 and H13), 4.24 (m, 1H, H11), 4.03 and 3.97 (m, 2H, Hb), 3.80-3.57 (m, 14H, Hc-Hh and H14), 2.44 (s, 3H, H1), 2.08-2.03 and 1.93-1.88 (m, 2H, H12), 0.94 (s, 9H, H18).**^{13}C NMR (125 MHz, d$_6$-DMSO)** δ 171.5 (C10), 169.1 (C15), 168.6 (Ca), 151.5 (C3), 147.7 (C2), 139.4 and 131.1 and 129.6 (C4, C5 and C8), 128.7 and 127.5 (C6 and C7), 70.9-69.3 (Cb-g), 68.9 and 58.7 (C9 and C13), 56.6 (C14), 55.7 (C16), 43.6 (Ch), 41.7 (C11), 37.9 (C12), 35.7 (C17), 26.2 (C18), 15.9 (C1). **HRMS (ESI)** *m/z* calcd for $C_{30}H_{43}ClN_4O_7S$ $[M+H]^+$: 639.2614; found: 639.2628.

PROTAC A

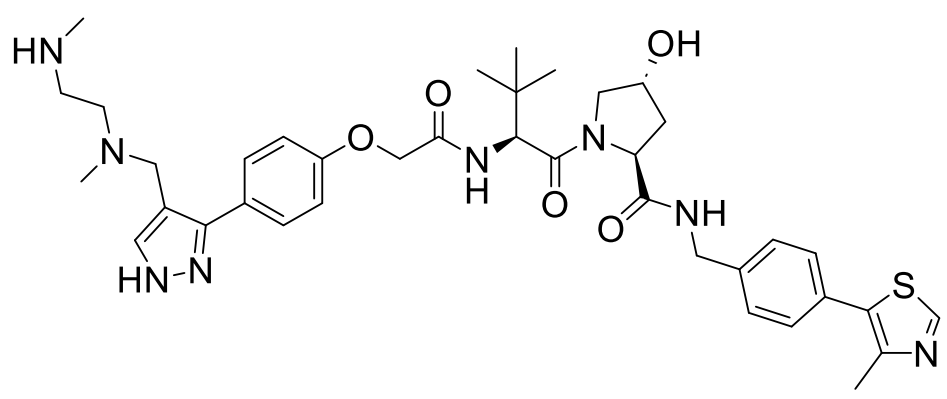

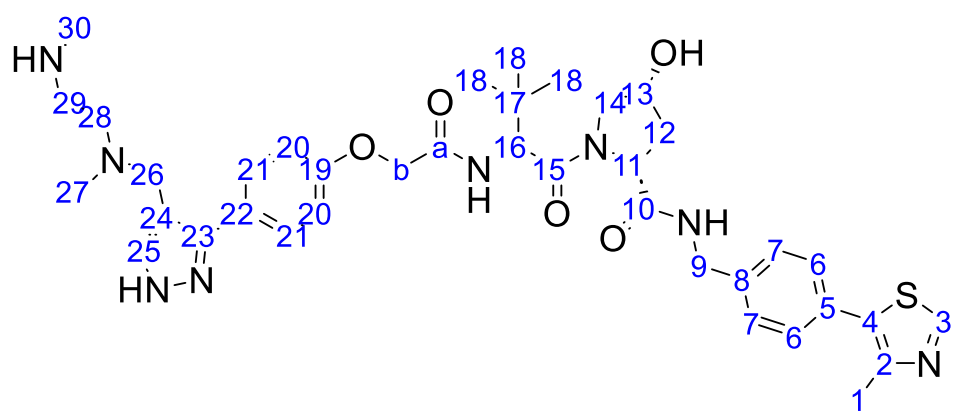

General method B was followed with **3a** (61 mg, 0.12 mmol, 1 eq) and PRMT1 ligand **4** (60 mg, 0.13 mmol, 1.1 eq). The alkylation reaction was heated at 80°C for 4 hr. Following deprotection, the residue was purified using automated column chromatography (C18 Gold, 5-60% MeCN in 0.5% $CH_3COOH_{(aq)}$ over 40 CV), followed by preparative HPLC (5-60% MeCN (0.05% TFA) in H_2O (0.1% TFA) over 20 min) and lyophilised to give the title compound as a white solid (22 mg, 25%). **^{1}H NMR (500 MHz, d$_6$-DMSO)** δ 10.29 (br s, 25-NH), 8.99 (s, 1H, H3), 8.84 (br s, 2H, 30-NH_2^+), 8.61 (t, *J*=6.0 Hz, 1H, 9-NH), 7.95 (d, *J*=9.4 Hz, 1H, 16-NH), 7.88 (s, 1H, H25), 7.52 (d, *J*=8.7 Hz, 2H, H21), 7.41 (m, 4H, H6 and H7), 7.07 (d, *J*=8.8 Hz, 2H, H20), 5.26 (br s, 1H, 13-OH), 4.70 (m, 2H, Hb), 4.60 (d, *J*=9.4 Hz, 1H, H16), 4.47-4.34 (m, 5H, H9, H13 and H26), 4.25 (dd, *J*=15.8 and 5.6 Hz, 1H, H11), 3.64 (m, 2H, H14), 3.25 (m, 4H, H28 and H29), 2.61 (s, 3H, H27), 2.56 (s, 3H, H30), 2.45 (s, 3H, H1), 2.09-2.05 and 1.94-1.88 (m, 2H, H12), 0.95 (m, 9H, H18). **^{13}C NMR (125 MHz, d$_6$-DMSO)** δ 171.8 (C10), 169.1 (C15), 167.1

(Ca), 157.1 (C19), 151.5 (C3), 147.7 (C2), 139.5, 131.1 and 129.7 (C4, C5 and C8), 128.8 (C21), 128.7 and 127.5 (C6 and C7), 114.6 (C20), 68.9 (C9 or C13), 66.5 (Cb), 60.0 (C9 or C13), 56.6 and 56.1 (C14 and C16), 52.7 (C27 or C30), 41.7 (C11), 37.9 (C12), 35.8 (C17), 26.2 (C18), 15.9 (C1). Peaks indicated by HSQC δ 60.0 and 59.0 (C28 and C29), 54.3 (C27 or C30). C22, C23, C24, C25 and C26 not identified. **HPLC** (5-95% MeCN (0.05% TFA) in H_2O (0.0.5% TFA) over 15 min) R_t = 6.63 min. Purity= 97%. **HRMS (ESI)** *m/z* calcd for $C_{38}H_{50}N_8O_5S$ $[M+H]^+$: 731.3698; found 731.3705.

PROTAC B

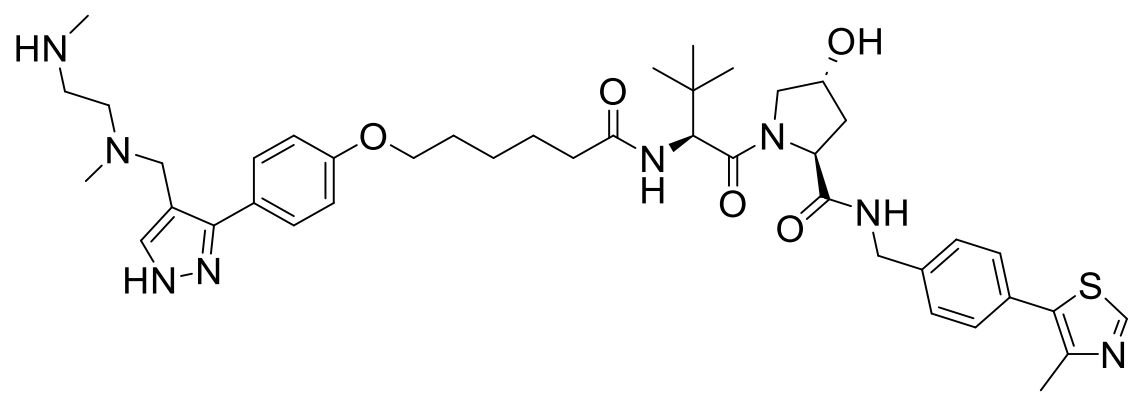

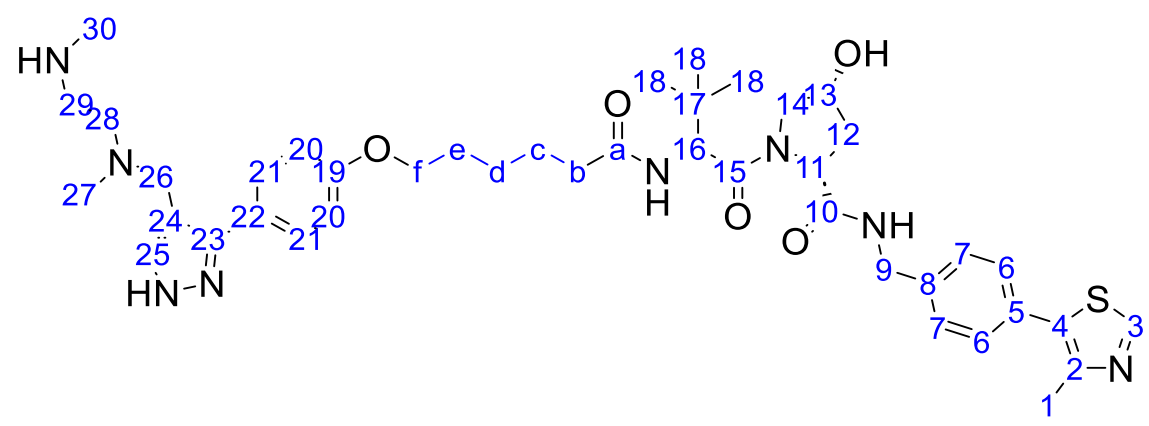

PRMT1 ligand **4** (16 mg, 0.04, 1 eq), **3b** (36 mg, 0.06 mmol, 1.8 eq), K_2CO_3 (17 mg, 0.13 mmol, 3.5 eq) and KI (2 mg, 0.01 mmol, 0.4 eq) were dissolved in MeCN (1 mL) and heated at 80°C for 5 days. The solvent was removed *in vacuo* and the residue purified by automated column chromatography (C18 Gold, 5-95% MeCN in H_2O over 45 CV) to give the title a white solid (14 mg). The solid was dissolved in MeOH (0.5 mL) and $HCl_{(aq,\ 37\%)}$ (0.5 mL) was added dropwise and the mixture stirred at room temperature for 1 hr. The solvent was then removed *in vacuo* and the residue purified by preparative HPLC (5-60% MeCN (0.05% TFA) in H_2O (0.1% TFA) over 20 min) and lyophilised to give the title compound as a white solid (9 mg, 37%). **^{1}H NMR (500 MHz, d_6-DMSO)** δ 9.97 (br s, 1H, 25-N<u>H</u>), 8.98 (s, 1H H3), 8.67 (br s, 2H, 30-N$\underline{H}_2^+$), 8.56 (t, *J*=6.1 Hz, 1H, 9-N<u>H</u>), 7.88 (d, *J*=9.4 Hz, 1H, 16-N<u>H</u>), 7.51 (m, 2H, H21), 7.40 (m, 4H, H6 and H7), 7.02 (d, *J*=8.1 Hz, 2H H20), 5.14 (br s, 1H, 13-O<u>H</u>), 4.56 (d, *J*=9.5 Hz, 1H, H16), 4.42 (m, 2H, H9), 4.35 (m, 1H, H13), 4.21 (dd, *J*=5.5 and 15.9 Hz, 1H, H11), 3.99 (t, *J*=6.4 Hz, 2H, Hf), 3.66 (m, 2H, H14), 3.17 (m, 4H (overlapping with H_2O), H28 and H29), 2.54 (m, 6H, H27 and H30), 2.44 (s, 3H, H1), 2.34-2.28 and 2.19-2.13 (m, 2H, Hb), 2.06-2.00 and 1.93-1.89 (m, 2H, H12), 1.73 (m, 2H, He), 1.60-1.38 (m, 4H, Hc and Hd), 0.94 (s, 9H, H18). H26 likely the broad peak at ~4.2 ppm. H25 not identified. **^{13}C NMR (125 MHz, d_6-DMSO)** δ 172.0 (Ca and C10), 169.7 (C15), 157.8 (q, $^2J_{C\text{-}F}$=31.7 Hz, $CF_3\underline{C}OO^-$), 151.5 (C3), 147.7 (C2), 139.5 and 131.2 and 129.6 (C4, C5 and C8), 128.6 and 127.4 (C6 and C7), 114.7 (C20), 68.9 (C13), 67.4 (Cf), 58.7 (C9), 56.4 and 56.3 (C14 and C16), 41.6 (C11), 38.0 (C12). 35.2 (C17), 34.8 (Cb), 32.7 (C27 or C30), 28.4 (Ce), 26.4 (C18), 25.2 and 25.2 (Cc and Cd), 15.9 (C1). Peaks indicated by HSQC δ 129.3 (C21). C19, C22, C23, C24, C25, C26, C27, C28, C29, C30 not identified. **HPLC** (5-95% MeCN (0.05% TFA) in H_2O (0.0.5% TFA) over 15 min) R_t = 6.97 min. Purity= 99%. **HRMS (ESI)** *m/z* calcd for $C_{42}H_{58}N_8O_5S$ $[M+H]^+$: 787.4324; found 787.4322.

PROTAC C

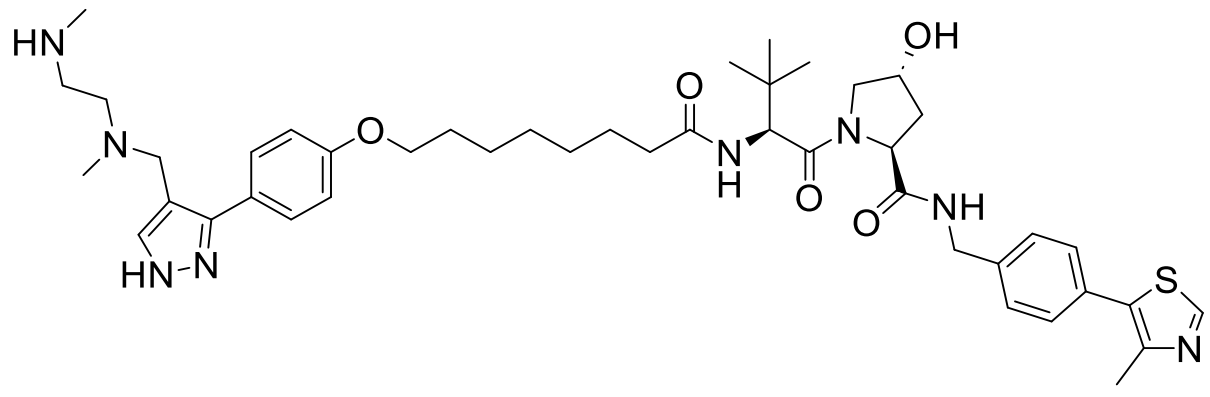

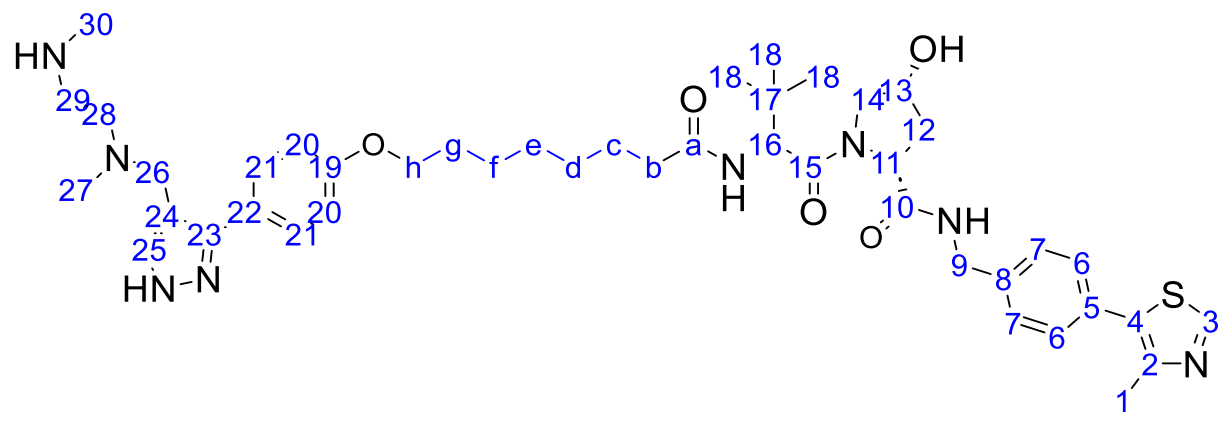

3c (40 mg, 0.06 mmol, 1 eq), PRMT1 ligand **4** (17 mg, 0.13, 2 eq), K_2CO_3 (17 mg, 0.13 mmol, 2 eq) and KI (2 mg, 0.01 mmol, 0.2 eq) were dissolved in MeCN (1 mL) and heated overnight at 80°C. The solvent was removed *in vacuo* and the residue purified by automated column chromatography (C18 Gold, 5-95% MeCN in 0.5% $HCOOH_{(aq)}$ over 45 CV). The product was then diluted with DCM, washed with water, dried with Na_2SO_4 and the solvent removed *in vacuo* to give a pale yellow solid (35 mg). The solid was dissolved in MeOH (0.5 mL) and $HCl_{(aq, 37\%)}$ (0.5 mL) was added dropwise and the mixture stirred at room temperature for 1 hr. The solvent was then removed *in vacuo* and the residue purified by preparative HPLC (5-70% MeCN (0.05% TFA) in H_2O (0.1% TFA) over 20 min) and lyophilised to give the title compound as a white solid (9 mg, 95%). **^{1}H NMR (500 MHz, d$_6$-DMSO)** δ 10.11 (br s, 1H, 25-NH), 8.98 (s, 1H, H3), 8.76 (br s, 2H, 30-NH_2^+), 8.58 (t, *J*=6.0 Hz, 1H, 9-NH), 7.85 (d, *J*=9.4, 1H, 16-NH), 7.83 (br s, 1H, H25), 7.49 (d, *J*=8.2 Hz, 2H, H20), 7.40 (m, 4H, H6 and H7), 7.03 (d, *J*=8.4 Hz, 2H, H21), 4.55 (d, *J*=9.4 Hz, 1H, H16), 4.45-4.41 (m, 2H, H9), 4.35 (m, 1H, H13), 4.21 (dd, *J*=15.9 and 5.4 Hz, 1H, H11), 4.00 (t, *J*=6.4 Hz, 3H, Hh), 3.65 (m, 2H, H14), 3.21 and 3.13 (two br s, 4H, H28 and H29), 2.59 and 2.56 (two br s, 6H, H27 and H30), 2.44 (s, 3H, H1), 2.30-2.25 and 2.15-2.09 (m, 2H, Hb), 2.05-2.01 and 1.92-1.88 (m, 2H, H12), 1.71 (m, 2H, Hg), 1.55-1.38 (m, 4H, Cc and f) 1.36-1.26 (m, 4H, Cd and Ce), 0.94 (s, 9H, H18). H26 likely the broad peak at ~4.3 ppm. 13-OH likely broad peak at ~5.1 ppm. **^{13}C NMR (176 MHz, d$_6$-DMSO)** δ 172.1 (Ca), 171.9 (C10), 169.7 (C15), 158.8 (C19), 158.1 (q, $^2J_{C\text{-}F}$=33.4 Hz, $CF_3\underline{C}OO$), 151.5 (C3), 147.7 (C2), 139.5 and 131.2 and 129.6 (C4, C5 and 8), 129.5 (C21), 128.6 and 127.4 (C6 and C7), 116.5 (q, $^1J_{C\text{-}F}$ =296.2 Hz, $\underline{C}F_3COO^-$), 114.8 (C20), 68.9 (C13), 67.5 (Ch), 58.7 (C9), 56.3 and 56.3 (C14 and C16), 41.6 (C11),~40.3 (C27 or C30), 38.0 (C12), 35.2 (C17), 34.8 (Cb), 32.7 (C27 or C30), 28.6-28.5 (Cd, Ce and Cg), 26.4 (C18), 25.4 and 25.4 (Cc and Cf), 15.9 (C1). C22, C23, C24, C25, C26, C28 and C29 not identified. **HPLC** (5-95% MeCN (0.05% TFA) in H_2O (0.05% TFA) over 15 min) R_t = 7.63 min. Purity= 99%. **HRMS (ESI)** *m/z* calcd for $C_{54}H_{78}N_8O_8S$ $[M+H]^+$: 999.5736; found 999.5729.

PROTAC D

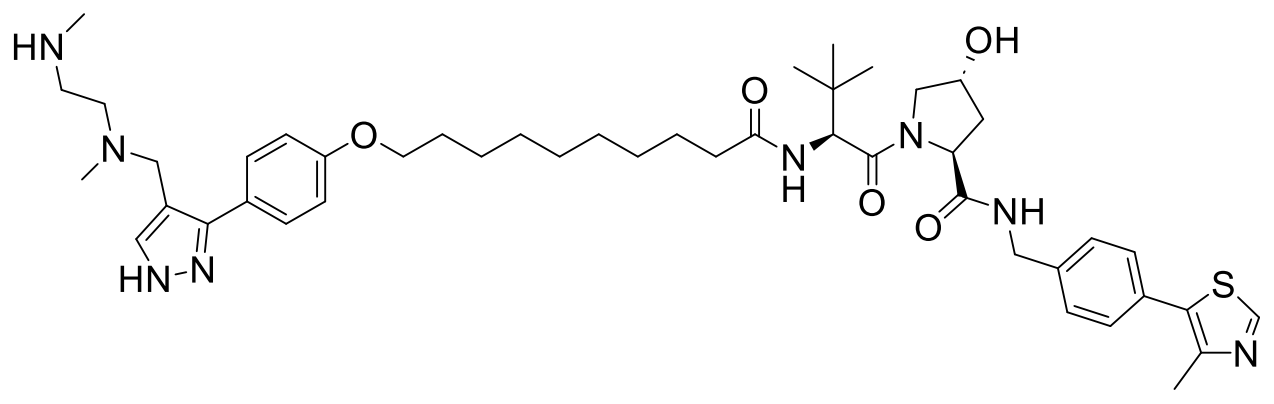

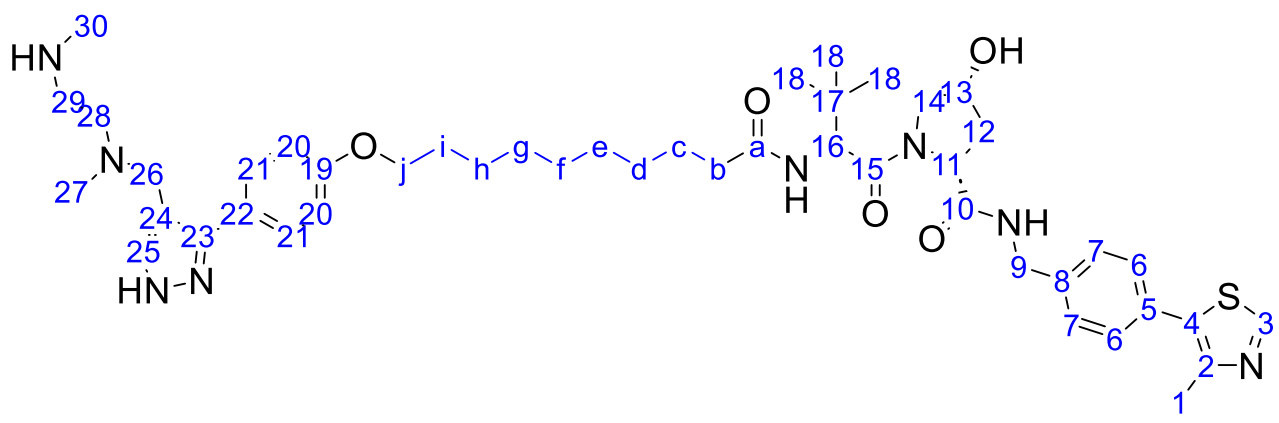

General method B was followed with **3d** (42 mg, 0.06 mmol, 1 eq) and PRMT1 ligand **4** (28 mg, 0.06 mmol, 1 eq). The alkylation reaction was heated overnight at 80°C. Following deprotection, the residue was purified by preparative HPLC (5-60% MeCN (0.05% TFA) in H_2O (0.1% TFA) over 30 min) and lyophilised to give the title compound as a white solid (8 mg, 15%). **^{1}H NMR (700 MHz, d$_6$-DMSO)** δ 10.08 (br s, 25-N<u>H</u>), 8.98 (s, 1H, H3), 8.75 (br s, 2H, 30-N<u>H</u>$_2^+$), 8.56 (t, *J*=6.0 Hz, 1H, 9-N<u>H</u>), 7.84 (m, 2H, H25 and 16-N<u>H</u>), 7.48 (d, *J*=8.3 Hz, 2H, H21), 7.40 (m, 4H, H6 and H7), 7.04 (d, *J*=8.3 Hz, 2H, H20), 4.54 (d, *J*=9.4 Hz, H16), 4.43 (m, 2H, H9), 4.35 (m, 3H, H13 and H26), 4.22 (dd, *J*=15.8 and 5.4 Hz, 1H, H11), 4.00 (t, *J*=6.4 Hz, 2H, Hj), 3.65 (m, 2H, H14), 3.24 and 3.16 (two br s, 4H, H28 and H29), 2.63 and 2.55 (two br s, 6H, H27 and H30), 2.44 (s, 3H, H1), 2.29-2.24 and 2.12-2.08 (m, 2H, Hb), 2.04-2.02 and 1.92-1.88 (m, 2H, H12), 1.72 (m, 2H, Hi), 1.52-1.50 (m, 2H, Hc), 1.43-1.38 (m, 2H, Hh), 1.32-1.24 (m, 8H, Cd-Cg), 0.93 (s, 9H, H18). 13-O<u>H</u> not identified. **^{13}C NMR (176 MHz, d$_6$-DMSO)** δ 172.1 (Ca), 171.9 (C10), 169.7 (C15), 158.6 (C19), 158.3 (q, $^{2}J_{C-F}$=34.6 Hz, CF_3<u>C</u>OO), 151.5 (C3), 147.7 (C2), 139.5 and 131.2 and 129.6 (C4, C5 and C8), 129.5 (C21), 128.6 and 127.4 (C6 and C7), 116.3 (q, $^{1}J_{C-F}$ =288.8 Hz, <u>C</u>F_3COO^-), 114.8 (C20), 68.9 (C13), 67.6 (Cj), 58.7 (C9), 26.3 and 56.2 (C14 and C16), 50.2 (C26), 49.3 and 42.5 (C28 and C29), 41.6 (C11), ~39.7 (C27 or C30), 38.0 (C12), 25.2 (Cb), 33.7 (C17), 32.7 (C27 or C30), 28.9-28.5 (Ci and Cd-Cg), 26.4 (C18), 25.5 and 25.4 (Cc and Ch), 15.9 (C1). Peak indicated by HSQC δ 137.4 (C25). C22, C23 and C24 not identified. **HPLC** (5-95% MeCN (0.05% TFA) in H_2O (0.0.5% TFA) over 15 min) R_t = 8.32 min. Purity= 89%. **HRMS (ESI)** *m/z* calcd for $C_{46}H_{66}N_8O_5S$ $[M+H]^+$: 843.4950; found 843.4943.

PROTAC E

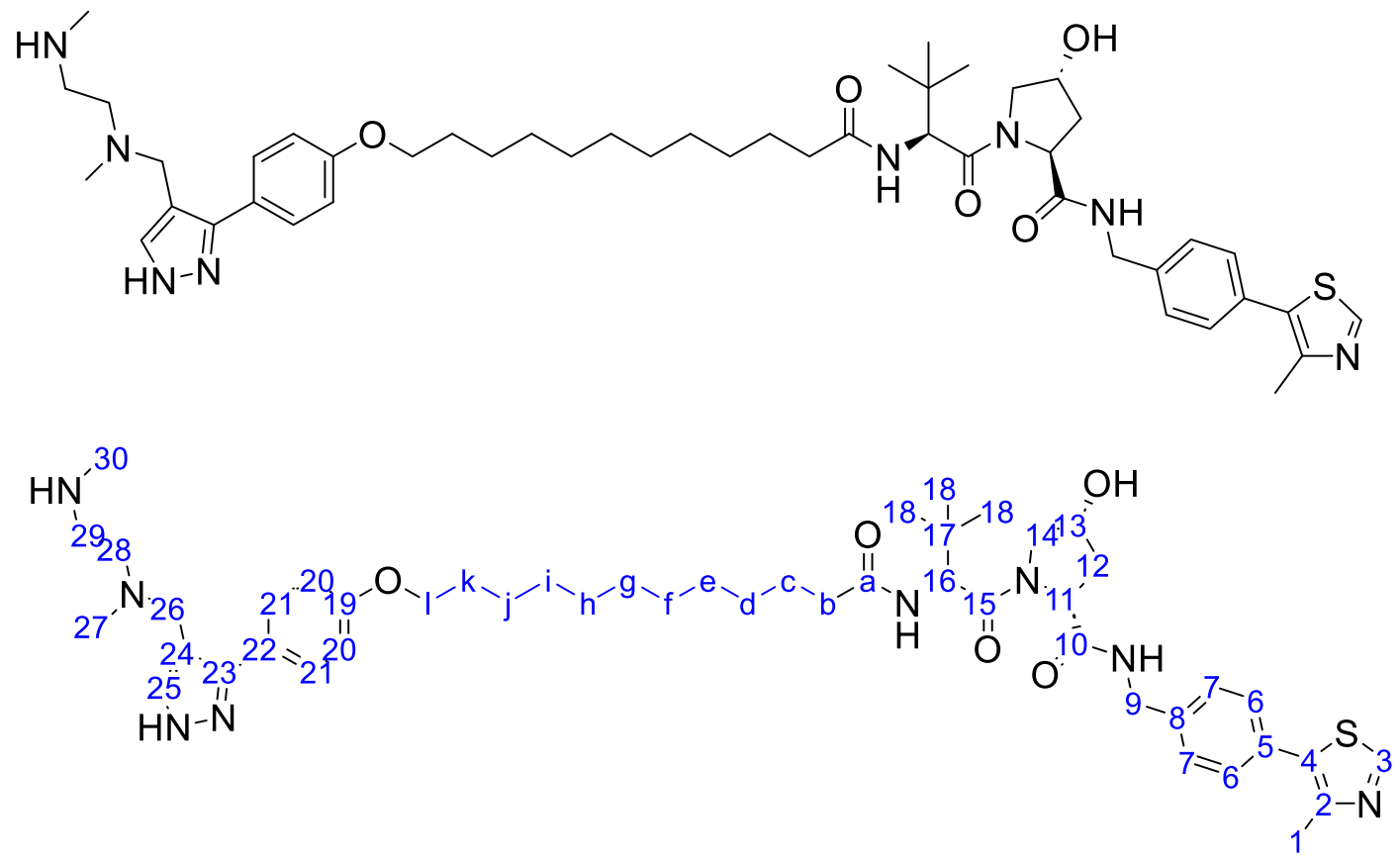

General method B was followed with **3e** (27 mg, 0.04 mmol, 1 eq) and PRMT1 ligand **4** (17 mg, 0.04 mmol, 1 eq). The alkylation reaction was heated overnight at 80°C. Following deprotection, the residue was purified using automated column chromatography (C18 Gold, 5-95% MeCN in 1% $CH_3COOH_{(aq)}$ over 45 CV), followed by preparative HPLC (5-95% MeCN (0.05% TFA) in H_2O (0.1% TFA) over 20 min) and lyophilised to give the title compound as a white solid (8 mg, 24%). **^{1}H NMR (700 MHz, d$_6$-DMSO)** δ 10.11 (br s, 1H, 25-NH), 8.98 (s, 1H, H3), 8.74 (br s, 2H, 30-NH_2^+), 8.56 (t, *J*=6.1 Hz, 1H, 9-NH), 7.84 (m, 2H, H25 and 16-NH), 7.50 (d, *J*=8.2 Hz, 2H, H21), 7.40 (m, 4H, H6 and H7), 7.03 (d, *J*=8.5 Hz, 2H, H20), 5.20 (br s, 1H, 13-OH), 4.54 (d, *J*=9.4 Hz, 1H, H16), 4.42 (m, 2H, H9), 4.35 (m, 1H, H13), 4.21 (dd, *J*=15.9 and 6.0 Hz, 1H, H11), 4.00 (t, *J*=6.5 Hz, 2H, Hl), 3.65 (m, 2H, H14), 3.21 and 3.11 (two br s, 4H, H28 and H29), 2.58-2.54 (m, 6H, H27 and H30), 2.44 (s, 3H, H1), 2.28-2.34 and 2.17-2.08 (m, 2H, Hb), 2.04-2.02 and 1.92-1.89 (m, 2H, H12), 1.72 (m, 2H, Hk), 1.53-1.40 (m, 4H, Hc and Hj), 1.32-1.24 (m, 12H, Hd-Hi), 0.93 (s, 9H, H18). H26 likely the broad peak at ~4.2 ppm. **^{13}C NMR (176 MHz, d$_6$-DMSO)** δ 172.1 (Ca), 172.0 (C10), 169.7 (C15), 158.7 (q, $^2J_{C-F}$=34.7 Hz, CF_3COO), 158.2 (C19), 151.9 (C3), 147.7 (C2), 139.5 and 131.2 and 129.6 (C4, C5 and C8), 129.4 (C21), 128.7 and 127.4 (C6 and C7), 114.7 (C20), 68.9 (C13), 67.6 (Cl), 58.7 (C9), 56.3 and 56.3 (C14 and C16), 41.7 (C11), 38.0 (C12), 35.2 (C17), 34.9 (Cb), 32.7 (C27 or C30), 29.0-28.7 (Cd-i and Ck), 26.4 (C18), 25.6 and 25.5 (Cc and Cj), 16.0 (C1). C22, C23, C24, C25, C26, C28, C29 and C27 or C30 not identified. **HPLC** (5-95% MeCN (0.05% TFA) in H_2O (0.0.5% TFA) over 15 min) R_t = 9.01 min. Purity= 96%. **HRMS (ESI)** *m/z* calcd for $C_{48}H_{70}N_8O_5S[M+H]^+$: 871.5263; found 871.5320.

PROTAC F

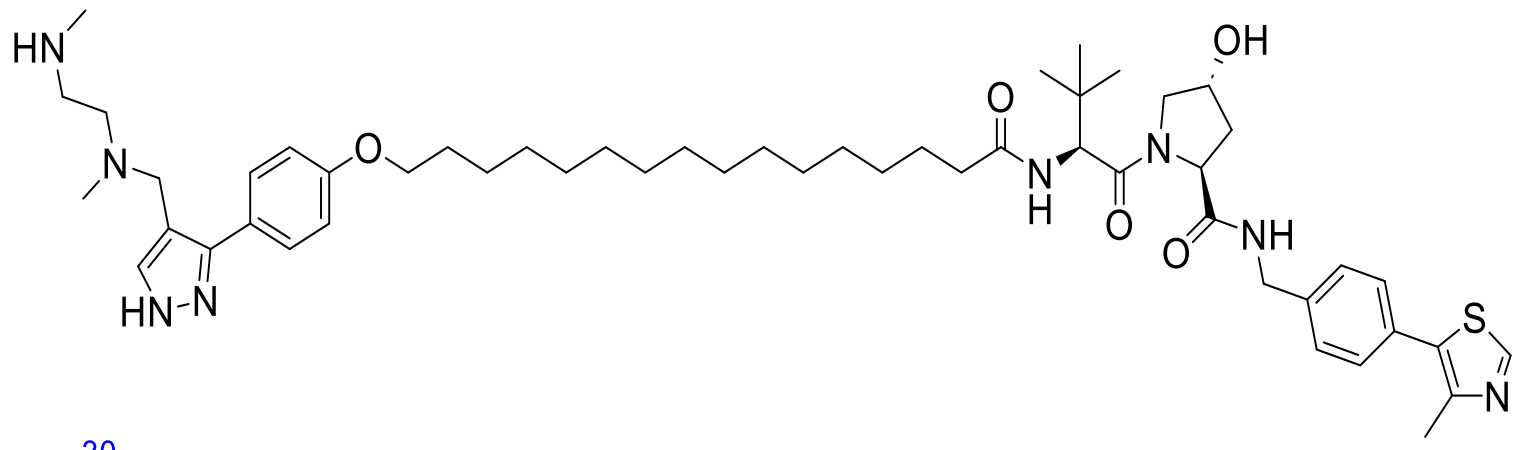

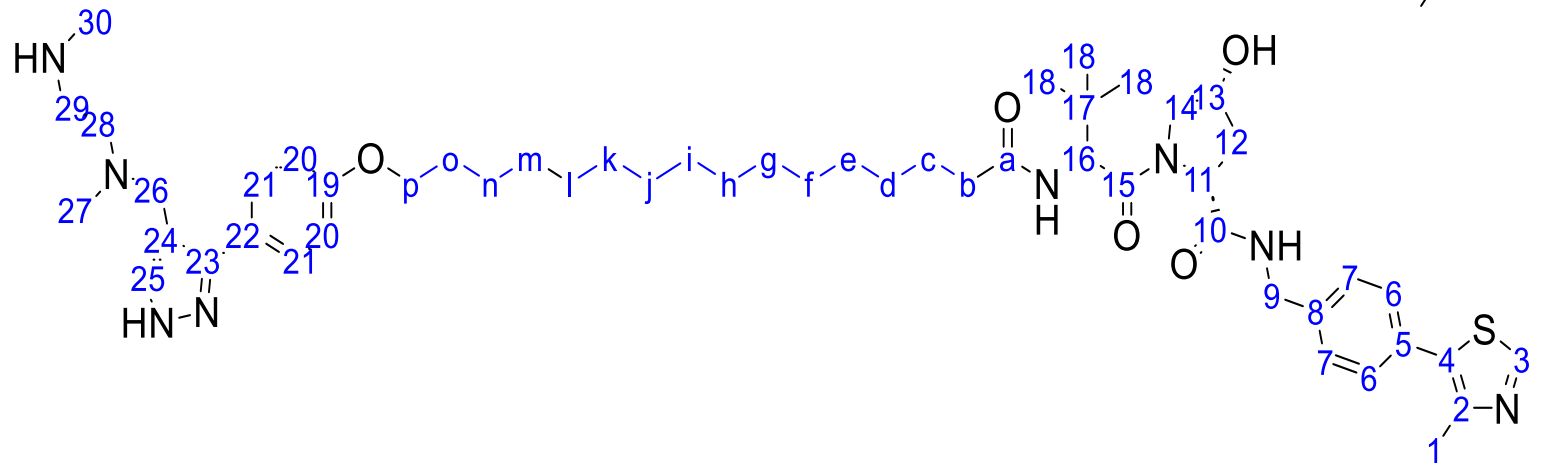

General method B was followed with **3f** (53 mg, 0.07 mmol, 1 eq) and PRMT1 ligand **4** (31 mg, 0.07 mmol, 1 eq). The alkylation reaction was heated overnight at 80°C. Following deprotection, the residue was purified by preparative HPLC (5-95% MeCN (0.05% TFA) in H_2O (0.1% TFA) over 20 min) and lyophilised to give the title compound as a white solid (7 mg, 10%). **^{1}H NMR (700 MHz, d$_6$-DMSO)** δ 10.17 (br s, 1H, 25-NH), 8.98 (s, 1H, H3), 8.76 (br s, 2H, 30-NH_2^+), 8.56 (t, *J*=6.1 Hz, 1H, 9-NH), 7.88 (s, 1H, H25), 7.83 (d, *J*=9.4 Hz, 1H, 16-NH), 7.48 (d, *J*=8.5 Hz, 2H, H21), 7.40 (m, 4H, H6 and H7), 7.063 (d, *J*=8.5 Hz, 2H, H20), 4.54 (d, *J*=9.4 Hz, 1H, H16), 4.43 (m, 2H, H9), 4.33 (m, 3H, H13 and H26), 4.21 (dd, *J*=15.8 and 5.5 Hz, 1H, H11), 4.00 (t, *J*=6.4 Hz, 2H, Hp), 4.65 (m, 2H, H14), 3.24 and 3.16 (two br s, 4H, H28 and H29), 2.62 and 2.55 (two br s, 6H, H27 and H30), 2.44 (s, 3H, H1), 2.28-2.24 and 2.11-2.07 (m, 2H, Hb), 2.07-2.01 and 1.92-1.88 (m, 2H, H12), 1.72 (m, 2H, Ho), 1.53-1.39 (m, 4H, Hc and Hn), 1.32-1.23 (m, 20H, Hd-Hm), 0.93 (s, 9H, H18). 13-OH likely broad peak at ~5.2 ppm. **^{13}C NMR (176 MHz, d$_6$-DMSO)** δ 172.1 (Ca), 172.0 (C10), 170.0 (C15), 158.8 (C19), 158.3 (q, $^2J_{C\text{-}F}$=34.7 Hz, CF_3COO$^-$), 151.5 (C3), 147.7 (C2), 139.5 (C4, C5 or C8), 131.2 (C4, C5 or C8), 129.6 and 129.5 (C21 and C4, C5, or C8), 128.6 and 127.4 (C6 and C7), 116.1 (q, $^1J_{C\text{-}F}$ =294.1 Hz, CF_3COO$^-$), 114.8 (C20), 68.9 (C13), 67.6 (Cp), 58.7 (C9), 56.3 and 56.3 (C14 and C16), 50.1 and 49.4 (C26 and C28 or C29), 42.5 (C28 or 29), 41.6 (C11), ~39.4 (C27 or C30), 38.0 (C12), 35.2 and 34.9 (Cb and C17), 32.6 (C27 or C30), 29.1-28.6 (Cd-m and Co), 26.4 (C18), 25.5 and 25.4 (Cc and Cn), 15.9 (C1). Peak indicated by HSQC δ 137.7 (C25). C22, C23 and C24 not identified. **HPLC** (5-95% MeCN (0.05% TFA) in H_2O (0.0.5% TFA) over 15 min) R_t = 10.61 min. Purity= 93%. **HRMS (ESI)** *m/z* calcd for $C_{52}H_{78}N_8O_5S$ [M+H]$^+$: 927.5889; found 927.5893.

PROTAC G

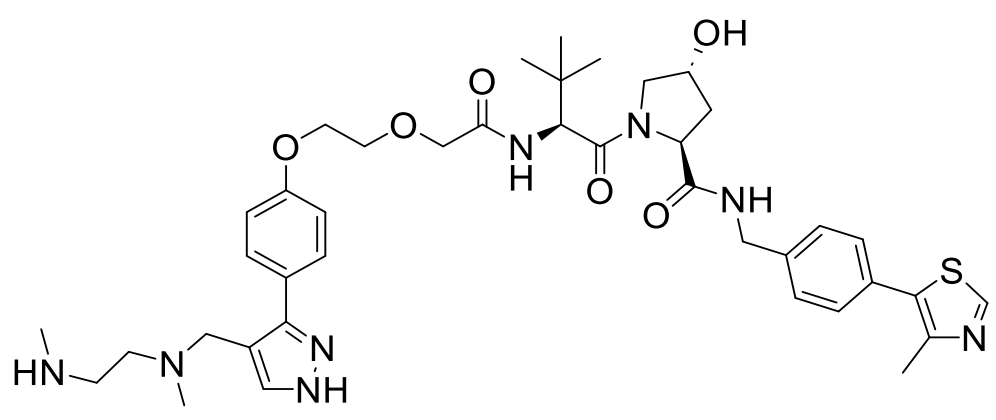 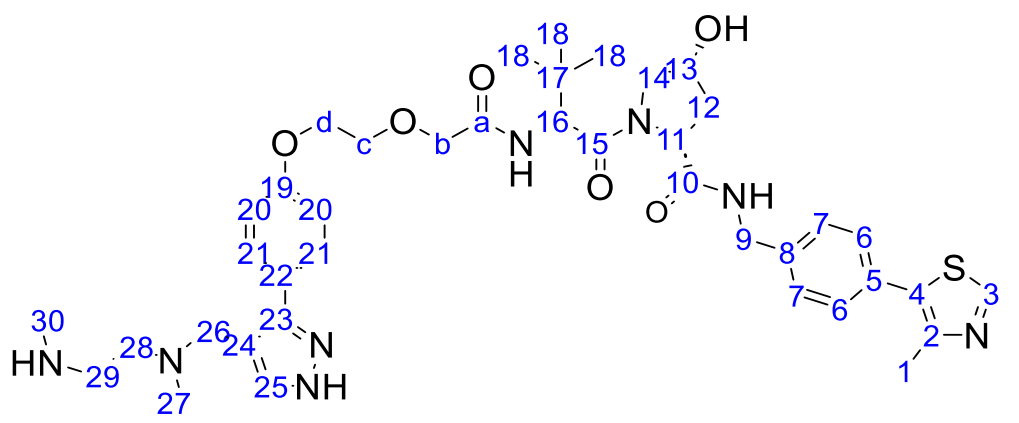

General method B was followed with **3g** (33 mg, 0.06 mmol, 1 eq) and PRMT1 ligand **4** (29 mg, 0.06 mmol, 1.1 eq). The alkylation reaction was heated at 80°C for 4 days. Following deprotection, the residue was purified by automated column chromatography (C18 Gold, 5-60% MeCN in 1% $HCOOH_{(aq)}$ over 45 CV), followed by preparative HPLC (5-60% MeCN (0.05% TFA) in H_2O (0.1% TFA) over 20 min) and lyophilised to give the title compound as a white solid (10 mg, 19%). **^{1}H NMR (500 MHz, d$_6$-DMSO)** δ 10.08 (br s, 1H, 25-NH), 8.96 (s, 1H, H3), 8.72 (m, 2H, 30-NH_2^+), 5.58 (t, *J*=6.0 Hz, 1H, 9-NH), 7.85 (s, 1H, 16-NH), 7.50 (m, 3H, H21 and H25), 7.39 (m, 4H, H6 and H7), 7.10 (d, *J*=8.8 Hz, 2H, H20), 4.60 (d, *J*=9.6 Hz, 1H, H16), 4.43 (m, 1H, H13), 4.34 (m, 2H, H9), 4.28 (dd, *J*=15.7 and 5.8 Hz, 1H, H11), 4.20 (s, 2H, Hd), 4.07 (m, 2H, Hb), 3.87 (m, 2H, Hc), 3.65 (m, 2H, H14), 3.22 and 3.15 (two br s, 4H, H28 and H29), 4.26 and 4.55 (two br s, 6H, H27 and H30), 2.42 (s, 3H, H1), 2.09-2.05 and 1.93-1.88 (m, 2H, H12), 0.96 (s, 9H, H18). H26 likely the broad peak at ~4.3 ppm. 13-OH likely broad peak at ~5.2 ppm. **^{13}C NMR (125 MHz, d$_6$-DMSO)** δ 171.7 (C10), 169.2 (C15) 168.5 (Ca), 158.3 (C19), 158.1 (q, $^2J_{C\text{-}F}$=34.6 Hz, CF_3COO^-), 151.5 (C3), 147.7 (C2), 139.4 and 131.1 and 129.7 (C4, C5 and C8), 129.5 (C21), 128.7 and 127.6 (C6 and C7), 116.2 (q, $^1J_{C\text{-}F}$= 294.5 Hz, CF_3COO^-), 114.9 (C20), 69.6 and 69.5 (Cb and Cc), 68.8 (C13), 67.1 (Cd), 58.7 (C9), 56.6 (C14), 55.7 (C16), 41.7 (C11), ~39.6 (C27 or C30), 38.0 (C12), 35.7 (C17), 32.7 (C27 or C30), 26.2 (C18), 15.9 (C1). C22, C23, C24, C25, C26, C28 and C29 not identified. **HPLC** (5-95% MeCN (0.05% TFA) in H_2O (0.0.5% TFA) over 15 min) R_t = 6.66 min. Purity= 97%. **HRMS (ESI)** *m/z* calcd for $C_{40}H_{54}N_8O_6S$ $[M+H]^+$: 775.3960; found 775.3969.

PROTAC H

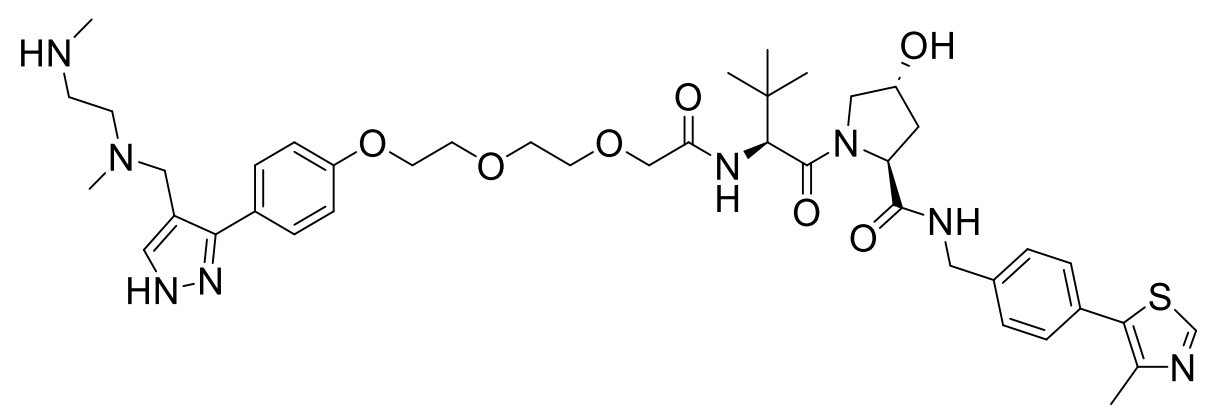

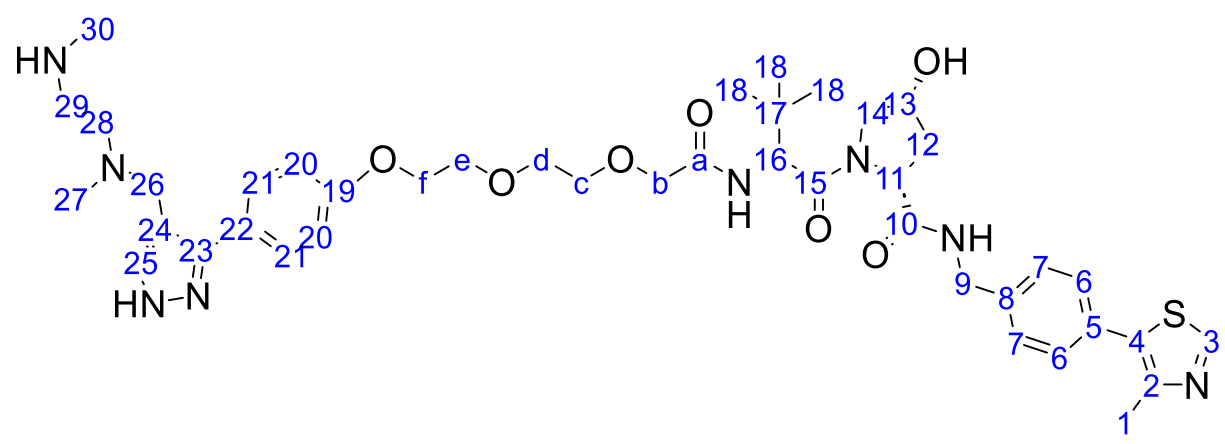

General method B was followed with **3h** (50 mg, 0.08 mmol, 1 eq) and PRMT1 ligand **4** (41 mg, 0.09 mmol, 1.1 eq) The alkylation reaction was heated at 80°C for 3 days. Following deprotection, the residue was purified by automated column chromatography (C18 Gold, 5-60% MeCN in 1% $HCOOH_{(aq)}$ over 40 CV), followed by preparative HPLC (5-40% MeCN (0.05% TFA) in H_2O (0.1% TFA) over 20 min) and lyophilised to give the title compound as a white solid (3 mg, 3%). **^{1}H NMR (500 MHz, d$_6$-DMSO)** δ 10.09 (br s, 1H, 25-N<u>H</u>), 8.97 (s, 1H, H3), 8.67 (m, 2H, 30-N<u>H</u>$_2^+$), 8.59 (t, *J*=6.0 Hz, 1H, 9-N<u>H</u>), 7.84 (br s, 1H, 16-N<u>H</u>), 7.49 (d, *J*=8.1 Hz, 2H, H21), 7.39 (m, 4H, H6 and H7), 7.04 (d, *J*=8.7 Hz, 2H, H20), 5.13 (br s, 1H, 13-O<u>H</u>), 5.46 (d, *J*=9.6 Hz, 1H, H16), 4.43 (t, *J*=8.2 Hz, 1H, H13), 4.37 (m, 2H, H9), 4.26 (dd, *J*=15.5 and 5.8 Hz, 1H, H11), 4.15 (t, *J*=4.6 Hz, 2H, Hf), 3.99 (m, 2H, Hb), 3.81 (m, 2H, Hc), 3.68 (m, 2H, H14), 3.64 (m, 4H, Hd and He), 3.20 and 3.10 (two br s, 4H, H28 and H29), 2.59 and 2.55 (two br s, 6H, H27 and H30), 2.43 (s, 3H, H1), 2.09-2.20 and 1.93-1.88 (m, 2H, H12), 0.94 (s, 9H, H18). H26 likely the broad peak at ~4.3 ppm. H25 not identified. **^{13}C NMR (125 MHz, d$_6$-DMSO)** δ 171.8 (C10), 169.2 (C15) 168.6 (Ca), 158.0 (C19), 151.5 (C3), 147.7 (C2), 139.4 and 131.1 and 129.7 (C4, C5 and C8), 129.5 (C21), 128.9 and 128.7 (C6 and C7), 114.8 (C20), 70.5-69.0 (Cb-Ce), 68.9 (C13), 67.2 (Cf), 58.8 (C9), 56.6 (C14), 55.7 (C16), 41.7 (C11), ~40.3 (C27 or C30), 38.0 (C12), 35.7 (C17), 32.7 (C27 or C30), 26.2 (C18), 15.9 (C1). C22, C23, C24, C25, C26, 28 and 29 not identified. **HPLC** (5-95% MeCN (0.05% TFA) in H_2O (0.0.5% TFA) over 15 min) R_t = 6.58 min. Purity= 99%. **HRMS (ESI)** *m/z* calcd for $C_{42}H_{58}N_8O_7S$ $[M+H]^+$: 819.4222 ; found 819.4218.

PROTAC I

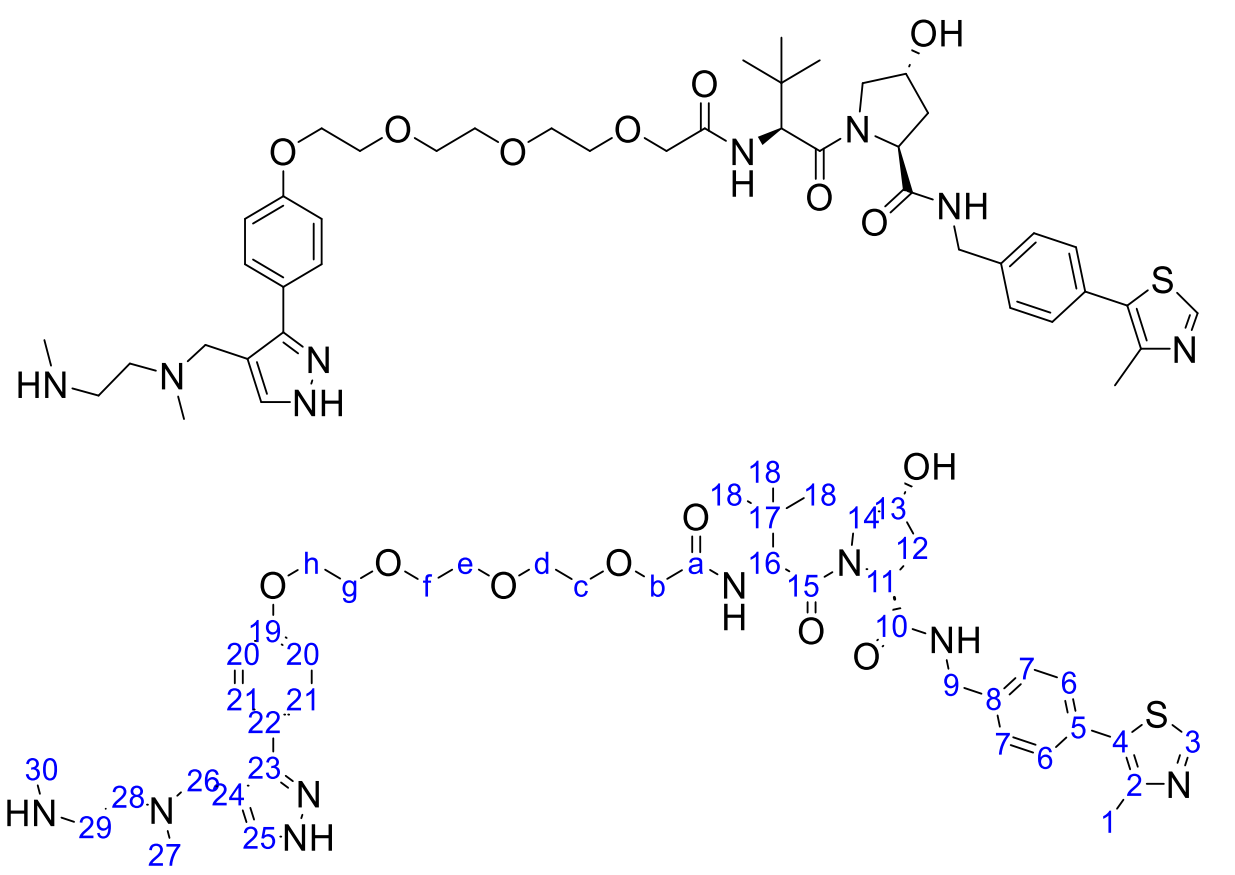

General method B was followed with **3i** (45 mg, 0.07 mmol, 1 eq) and PRMT1 ligand **4** (44 mg, 0.08 mmol, 1.1 eq). The alkylation reaction was heated at 80°C for 4 days. Following deprotection, the residue was purified by automated column chromatography (C18 Gold, 5-60% MeCN in 1% $HCOOH_{(aq)}$ over 45 CV), followed by preparative HPLC (5-60% MeCN (0.05% TFA) in H_2O (0.1% TFA) over 20 min) and lyophilised to give the title compound as a white solid (6 mg, 10%). **^{1}H NMR (700**

MHz, d$_6$-DMSO) δ 9.98 (br, s, 1H, 25-NH), 8.98 (s, 1H, H3), 8.67 (m, 2H, 30-NH_2^+), 8.59 (t, *J*=6.0 Hz, 1H, 9-NH), 7.85 (br s, 1H, 16-NH), 7.50 (d, *J*=8.1 Hz, 2H, H21), 7.40 (m, 4H, H6 and H7), 7.05 (d, *J*=8.4 Hz, 2H, H20), 5.12 (br s, 1H, 13-OH), 4.57 (d, *J*=9.6 Hz, 1H, H16), 4.39 (dd, *J*=15.7 and 6.4 Hz, 1H, H13), 4.40 (m, 4H , H9 and H26), 4.25 (dd, *J*=15.7 and 5.6 Hz, 1H, H11), 4.11 (m, 2H, Hh), 3.97 (m, 2H, Hb), 3.74 (m, 2H, Hg), 3.67 (dd, *J*=10.7 and 4.0 Hz, 2H, H14), 3.60 (m, 8H, Hc-f), 3.21 and 3.14 (two br s, 4H, H28 and H29), 2.61 and 2.55 (two br s, 6H, H27 and H30), 2.44 (s, 3H, H1), 2.08-2.05 and 1.92-1.88 (m, 2H, H12), 0.95 (s, 9H, H18). H25 not identified. **^{13}C NMR (176 MHz, d$_6$-DMSO)** δ 171.8 (C10), 169.1 (C15) 168.6 (Ca), 158.6 (C19), 158.3 (q, $^2J_{C-F}$=33.5 Hz, $CF_3\underline{C}OO^-$), 151.5 (C3), 147.7 (C2), 139.5 and 131.1 and 129.7 (C4, C5 and C8), 129.5 (C21), 128.7 and 127.5 (C6 and C7), 116.5 (q, $^1J_{C-F}$= 296.6 Hz, $\underline{C}F_3COO^-$), 114.8 (C20), 70.4-69.6 (Cb-g), 68.9 (C13), 67.2 (Ch), 58.8 (C9), 56.6 (C14), 55.7 (C16), 49.8 and 42.4 (C28 and C29), 41.7 (C11), ~39.7 (C27 or C30), 37.9 (C12), 35.7 (C17), 32.7 (C27 or C30), 26.2 (C18), 16.0 (C1). C22, C23, C24, C25 and C26 not identified. **HPLC** (5-95% MeCN (0.05% TFA) in H_2O (0.0.5% TFA) over 15 min) R_t = 6.61 min. Purity= 86%. The major impurity at 6.51 min is **PROTAC H** (12%). **HRMS (ESI)** *m/z* calcd for $C_{44}H_{62}N_8O_6S$ [M+H]$^+$: 863.4490 ; found 863.4476.

5.3.2 CRBN-Recruiting PROTACs

General method C PRMT1 ligand **4** (1 eq) and Cs_2CO_3 (2 eq) were dissolved in DMF (10 mL/mmol). The respective linker **8** (4 eq) in DMF (5 mL/mmol) was added dropwise at 0°C and the rection stirred overnight at room temperature. The reaction was then purified by automated column chromatography (C18 Gold, 5-60% MeCN in 1% $HCOOH_{(aq)}$ over 40 CV).

General method D CRBN ligand **14** (1 eq) and linker **15** (1.1 eq) were dissolved in DMSO (10 mL/mmol). DIPEA (2 eq) was added and the mixture heated at 130°C for 2 hr. The mixture was then diluted with water and extracted with EtOAc. The organic layer was washed with water (x3), washed with brine, dried with Na_2SO_4 and the solvent removed *in vacuo*. The residue was then purified by automated column chromatography (C18 Gold, 5-95% MeCN in 1% $HCOOH_{(aq)}$ over 40 CV).

General method E The alcohol **16**(1 eq) was dissolved in DCM (5 mL/mmol). At 0°C, Et_3N (1.5 eq) and methanesulfonyl chloride (1.5 eq) were added in succession and the mixture stirred at room temperature for 3 hr. The reaction was quenched by the careful addition of MeOH and the solvent removed *in vacuo*. The residue was dissolved in water and extracted with EtOAc. The organic layer was washed with brine, dried with Na_2SO_4 and the solvent removed *in vacuo*. The residue and NaI (2 eq) were dissolved in MeCN (5 mL/ml) and heated at 50°C overnight. The solvent was removed *in vacuo* and the residue diluted with $Na_2S_2O_{3(aq,\ 10\%)}$ and extracted with DCM (x3). The combine organic layers were washed with water, dried with Na_2SO_4 and the solvent removed *in vacuo*.

tert-butyl (2-(((3-(4-(2-(2-chloroethoxy)ethoxy)phenyl)-1-(tetrahydro-2_H_-pyran-2-yl)-1_H_-pyrazol-4-yl)methyl)(methyl)amino)ethyl)(methyl)carbamate, (5).

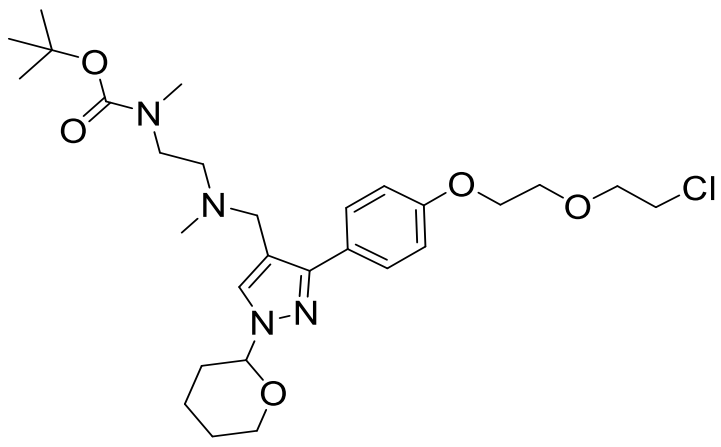
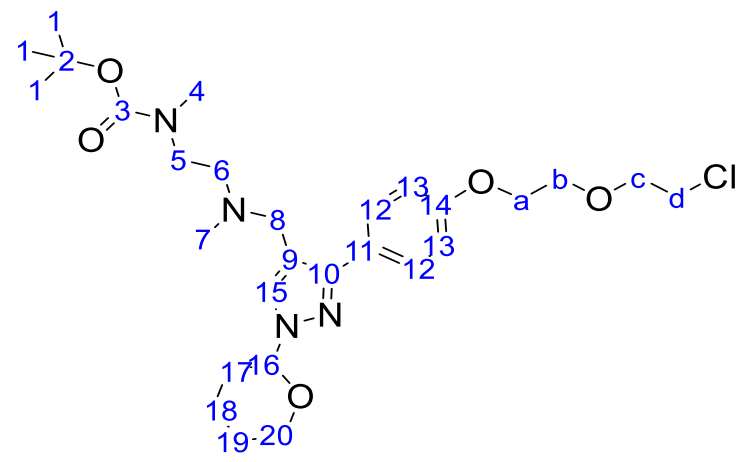

To a solution of triphenylphosphine (76 mg, 0.46 mmol, 2 eq) and 2-(2-chloroethoxy)ethanol (24 µL, 0.46 mmol, 2 eq) dissolved in THF (200 µL) was added DTBAD (68 mg, 0.46 mmol) in THF (200 µL) at 0°C. Finally, a solution of PRMT1 ligand 4 (100 mg, 0.23 mmol, 1 eq) in THF (200 µL) was added dropwise at 0°C. The mixture was stirred for 1 h at 0 °C then allowed to warm to room temperature and stirred overnight. The solvent was removed *in vacuo* and the residue purified by automated column chromatography (C18 Gold, 5-60% MeCN in 1% $HCOOH_{(aq)}$ over 45 CV) to give the title compound as an white solid (67 mg, 54%). **^{1}H NMR (500 MHz, d_6-DMSO)** δ 7.81 (s, 1H, H15), 7.75 (d, *J*=8.1 Hz, 2H, H12), 6.98 (d, *J*=8.8 Hz, 2H, H13), 5.36 (dd, *J*=9.8 and 2.3 Hz, 1H, H16), 4.12 (m, 2H, Ha), 3.93 (m, 1H, H20), 3.81 (m, 2H, Hb), 3.75 (s, 4H, Hc and Hd), 3.63 (m, 1H, H20), 3.42 and 3.38 (s, 2H, H8), 3.29 (m, 2H, H5), 2.75 and 2.73 (s, 3H, H4), 2.51 (m, 2H, H6), 2.19 (s, 3H, H7), 2.12-2.05 (m, 1H, H17), 1.96-1.90 (m, 2H, H17 and H18), 1.66-1.63 (m, 1H, H18), 1.55-1.51 (m, 2H, H19), 1.37 and 1.34 (s, 9H, H1). Rotamers present. **^{13}C NMR (125 MHz, d_6-DMSO)** δ 157.9 (C14), 154.8 (C3), 149.3 (C10), 130.7 (C15), 128.9 (C12), 126.3 (C11), 114.3 (C13), 86.7 (C16), 78.3 (C2), 70.7 (Cc), 68.8 (Cb), 67.1 (Ca), 66.8 (C20) 43.6 (Cd), 40.9 (C7), 34.1 (C4), 29.8 (C17), 28.0 (C1), 24.6 (C19), 22.1 (C18). Peaks indicated by HSQC δ 54.6 (C6), 52.2 (C8), 45.9 (C5). Peak indicated by HMBC δ 115.1 (C9). **HRMS (ESI)** *m/z* calcd for $C_{28}H_{43}ClN_4O_5$ $[M+H]^+$: 551.2995; found 551.3007.

4-hydroxy-2-(1-(2-(2-(4-(4-((methyl(2-(methylamino)ethyl)amino)methyl)-1_H_-pyrazol-3-yl)phenoxy)ethoxy)ethyl)-2,6-dioxopiperidin-3-yl)isoindoline-1,3-dione, (7)

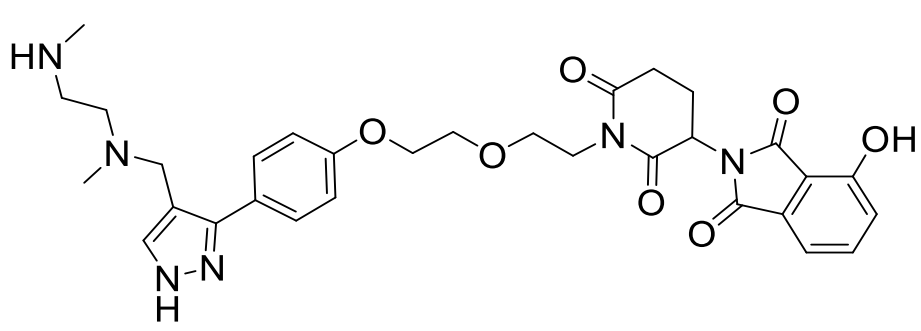
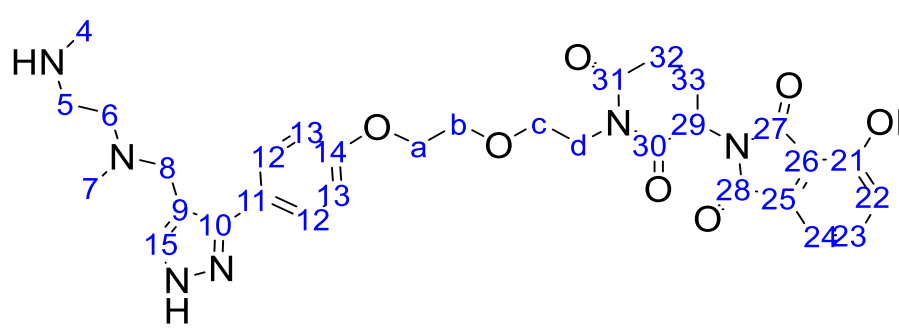

5 (27 mg, 0.05 mmol, 1 eq) in DMSO (1.4 mL) was added to 2-(2,6-Dioxopiperidin-3-yl)-4-hydroxyisoindoline-1,3-dione {CAS: 5054-59-1} (16 mg, 0.06 mmol, 1.2 eq), K_2CO_3 (13 mg, 0.10 mmol,

2 eq) and KI (8 mg, 0.05 mmol, 1 eq) and heated overnight at 80°C. The mixture was then diluted with water and extracted with DCM (x3). The combined organic layers were washed with water, dried with Na_2SO_4 and the solvent removed *in vacuo*. The residue was dissolved in MeOH (500 µL) and $HCl_{(aq,\ 37\%)}$ (500 µL) was added dropwise and the mixture stirred at room temperature for 1 hr. The solvent was then removed *in vacuo* and the residue was purified by preparative HPLC (5-40% MeCN (0.05% TFA) in H_2O (0.1% TFA) over 20 min) and lyophilised to give the title compound a pale yellow solid (11 mg, 34%).**^{1}H NMR (500 MHz, d$_6$-DMSO)** δ 11.35 (br s, 1H, 21-OH), 10.22 (br s, 1H, 15-NH), 8.80 (br s, 2H, 4-NH_2^+), 7.86 (s, 1H, H15), 7.66 (dd, *J*=8.4 and 7.2 Hz, 1H, H23), 7.49 (d, *J*=8.7 Hz, 2H, H12), 7.32 (d, *J*=7.2 Hz, 1H, H24), 7.28 (d, *J*=8.4 Hz, 1H, H22), 7.07 (d, *J*=8.8 Hz, 2H, H13), 5.17 (dd, *J*=13.0 and 5.4 Hz, H29), 4.33 (m, 2H, H8), 4.12 (m, 2H, Ha), 3.94-3.81 (m, 2H, Hd), 3.76 (m, 2H, Hb), 3.52 (m, 2H, Hc), 3.23 and 3.17 (two br s, 4H, H5 and H6), 3.01-2.94 and 2.78-2.73 (m, 2H, H32), 2.61 and 2.55 (two br s, 6H, H4 and H7), 2.54 and 2.07-2.03 (m, 2H, H33). **^{13}C NMR (125 MHz, d$_6$-DMSO)** δ 171.6 and 169.6 (C30 and C31), 167.0 and 165.8 (C27 and C28), 158.4 (q, $^2J_{C\text{-}F}$=33.5 Hz, CF_3COO^-), 155.6 (C21), 136.4 (C23), 133.1 (C15), 129.6 (C12), 123.6 (C22), 114.9 (C13), 114.3 (C24), 68.7 (Cb), 67.2 (Ca), 66.9 (Cc), 49.3 (C29), 38.6 (Cd), 31.2 (C32), 21.3 (C33). Peaks indicated by HSQC δ 50.5 (C8), 49.7 and 42.9 (C5 and C6), 39.3 and 33.1 (C4 and C7). C9, C10, C11, C14, C25 and C26 not detected. **HPLC** (5-95% MeCN (0.05% TFA) in H_2O (0.0.5% TFA) over 15 min) R_t = 6.18 min. Purity= 99%. **HRMS (ESI)** *m/z* calcd for $C_{31}H_{36}N_6O_7$ $[M+H]^+$: 605.2718; found 605.2719.

tert-butyl (2-(((3-(4-(2-(2-(2-(2-iodoethoxy)ethoxy)ethoxy)ethoxy)phenyl)-1-(tetrahydro-2*H*-pyran-2-yl)-1*H*-pyrazol-4-yl)methyl)(methyl)amino)ethyl)(methyl)carbamate, (9j).

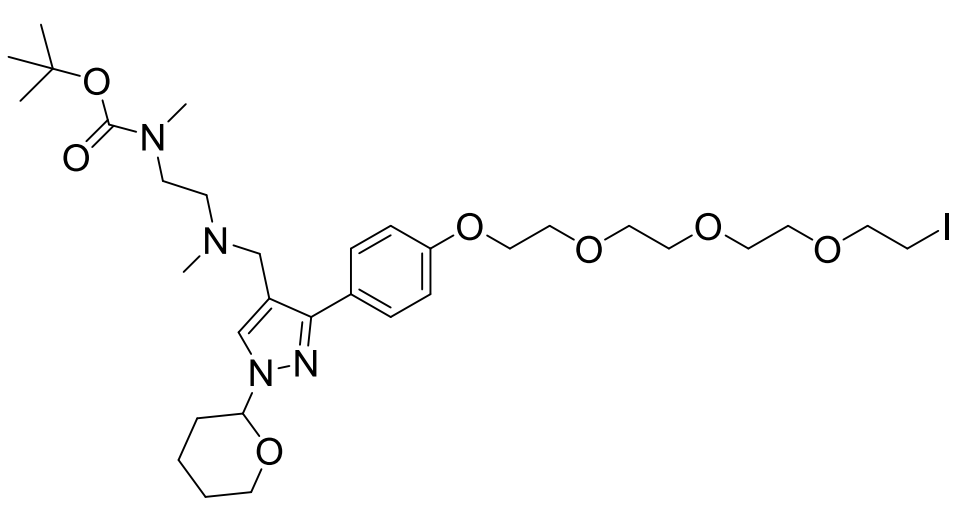

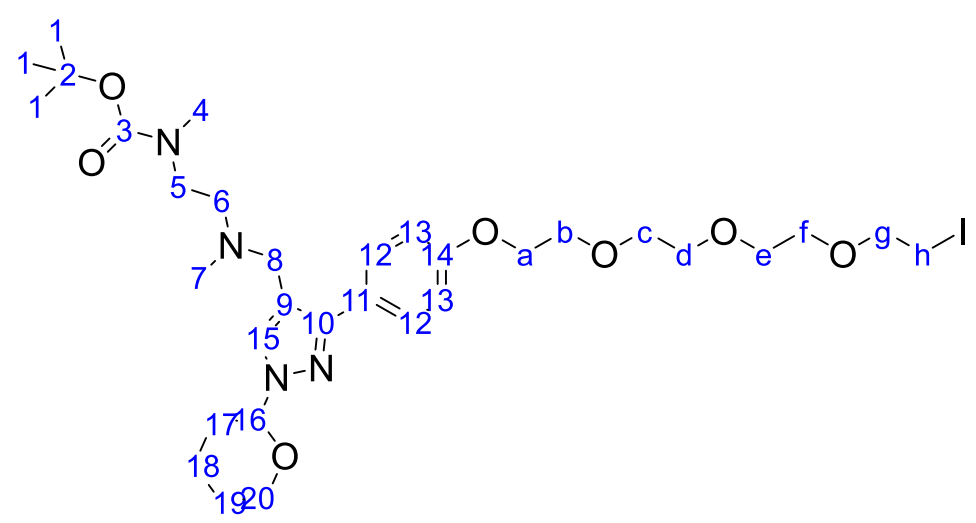

General method C was followed with PRMT1 ligand **4** (39 mg, 0.09 mmol) and the linker **8j.** The title compound was isolated as a yellow oil (41 mg, 65%). **^{1}H NMR (500 MHz, $CDCl_3$)** δ 7.72-7.51 (m, 3H, H12 and H15), 6.97 (d, *J*=8.7 Hz, 2H, H13), 5.39 (dd, *J*=9.7 and 2.4 Hz, 1H, H16), 4.17 (m, 2H, Ha), 4.10 (m, 1H, H20), 3.98 (m, 1H, H8), 3.89 (m, 2H, Hb), 3.78-3.68 (m, 12H, H20 and H8 and Hc-g), 3.50 and 3.36 (m, 2H, H5), 3.27 (m, 2H, Hh), 2.82 (s, 3H, H4), 2.80 and 2.62 (m, 2H, H6), 2.33 (s, 3H, H7), 2.15-2.05 (m, 3H, H17 and H18), 1.73-1.68 (m, 2H, H18 and H19), 1.64-1.58 (m, 1H, H19), 1.45 (s, 9H, H1). Rotamers present. **^{13}C NMR (125 MHz, $CDCl_3$)** δ 167.2 (C3), 158.7 (C14), 130.8 (C15), 129.7 (C12),

114.8 (C13), 87.9 (C16), 79.8 (d, C2), 72.0-70.3 (Cc-Cg), 69.8 (Cb), 68.0 (C20), 67.5 (Ca), 54.1 and 52.2 (C6), 51.8 and 49.8 (C8), 46.1 and 44.7 (C5), 40.5 (d, C7), 34.8 (d, C4), 30.6 (C17), 28.5 (C1), 25.0 (C19), 22.6 (C18), 3.1 (Ch). Rotamers present. C9, C10, C11 not identified. **HRMS (ESI)** *m/z* calcd for $C_{32}H_{51}IN_4O_7$ $[M+H]^+$: 731.2875; found 731.2878.

tert-butyl (2-(((3-(4-((14-iodo-3,6,9,12-tetraoxatetradecyl)oxy)phenyl)-1-(tetrahydro-2*H*-pyran-2-yl)-1*H*-pyrazol-4-yl)methyl)(methyl)amino)ethyl)(methyl)carbamate, (9k).

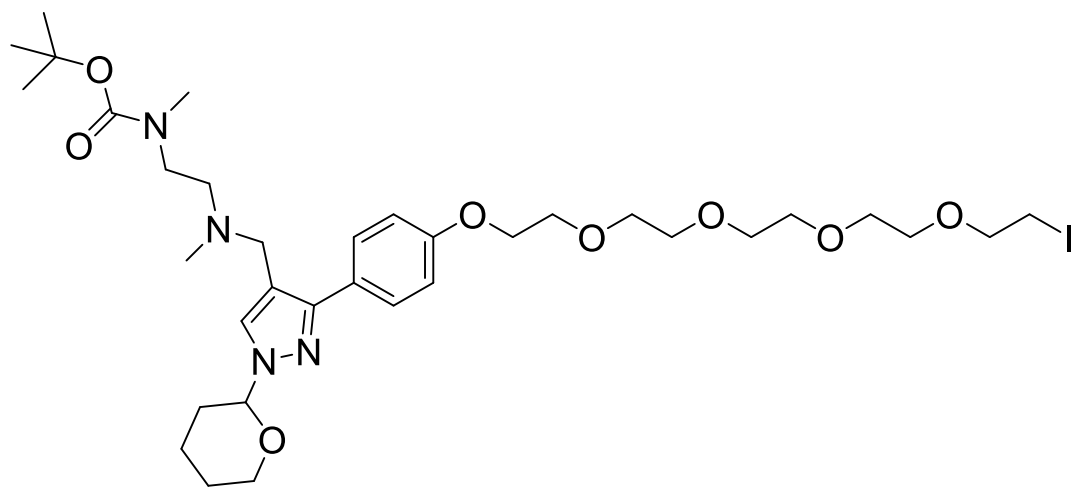

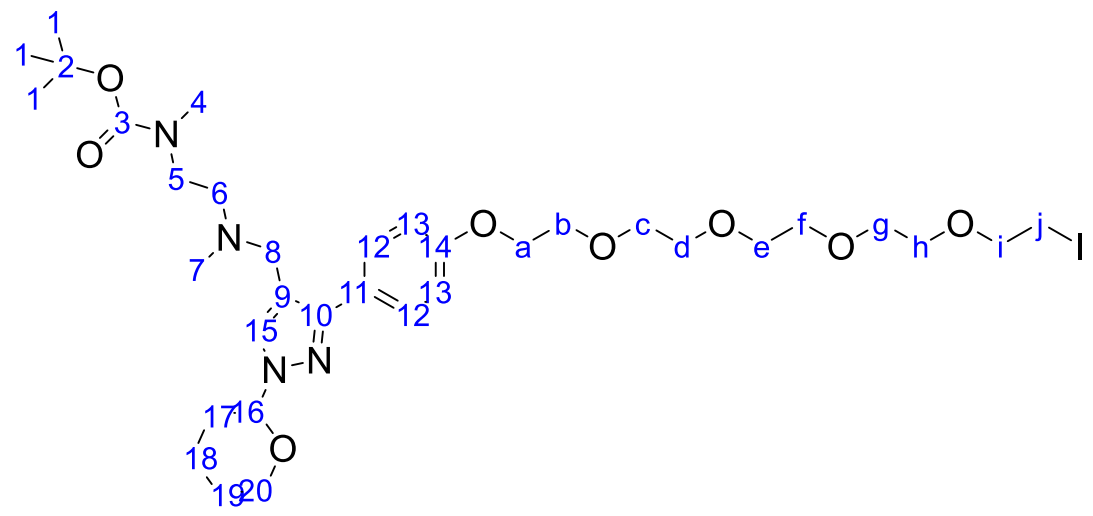

General method C was followed with PRMT1 ligand **4** (38 mg, 0.08 mmol) and linker **8k** (1,14-diiodo-3,6,9,12-tetraoxatetradecane). The title compound was isolated as an orange oil (38 mg, 58%). **^{1}H NMR (500 MHz, $CDCl_3$)** δ 7.87-7.56 (m, 3H, H12 and H15), 6.95 (d, *J*=8.8 Hz, 2H, H13), 5.37 (dd, *J*=9.6 and 2.7 Hz, 1H, H16), 4.15 (m, 2H, Ha), 4.07 (m, 1H, H20), 3.86 (m, 2H, Hb), 3.83 (m, 1H, H8), 3.75-3.65 (m, 15H, Hc-Hi and H20), 3.57 (m, 1H, H8), 3.45 and 3.33 (m, 2H, H5), 3.25 (m, 2H, Hj), 2.81 (s, 3H, H4), 2.75 and 2.55 (m, 2H, H6), 2.29 (m, 3H, H7), 2.16-2.03 (m, 3H, H17 and H18), 1.73-1.64 (m, 2H, H18 and H19), 1.62-1.58 (m, 1H, H19), 1.43 (s, 9H, H1). Rotamers present. **^{13}C NMR (125 MHz, $CDCl_3$)** δ 158.7 (C14), 130.6 (C15), 129.7 (C12), 114.8 (C13), 88.0 (C16), 79.8 (d, C2), 72.1-70.3 (Cc-Ci), 69.9 (Cb), 68.1 (C20), 67.6 (Ca), 54.5 and 52.9 (C6), 52.3 and 50.5 (C8), 46.4 and 45.1 (C5), 40.9 (d, C7), 34.8 (d, C4), 30.6 (C17), 28.5 (C1), 25.1 (C19), 22.7 (C18), 3.1 (Cj). Rotamers present. C3, C9, C10, C11 not identified. **HRMS (ESI)** *m/z* calcd for $C_{34}H_{55}IN_4O_8$ $[M+H]^+$: 775.3137; found 775.3169.

tert-butyl (2-(((3-(4-((17-iodo-3,6,9,12,15-pentaoxaheptadecyl)oxy)phenyl)-1-(tetrahydro-2*H*-pyran-2-yl)-1*H*-pyrazol-4-yl)methyl)(methyl)amino)ethyl)(methyl)carbamate, (9l).

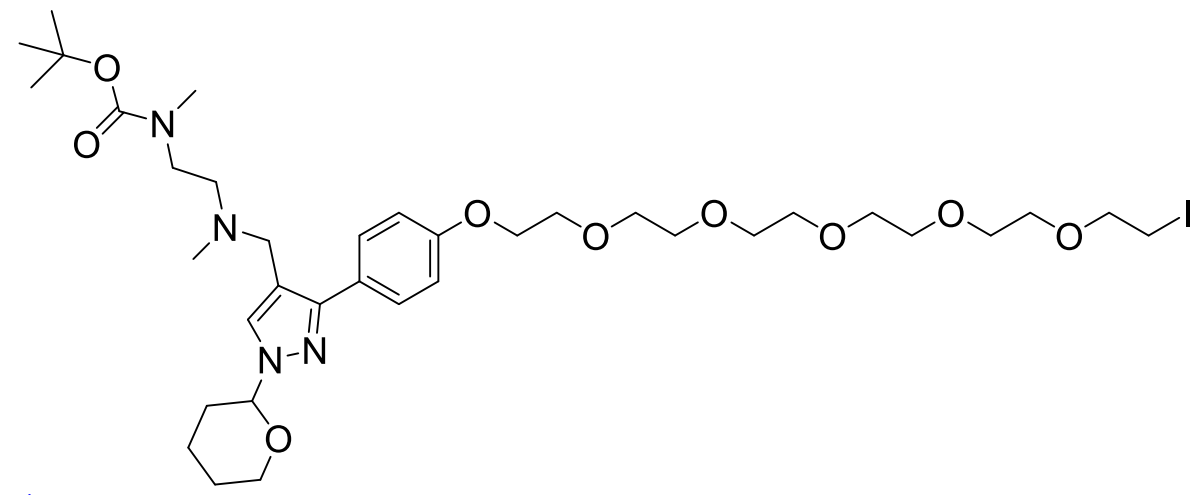

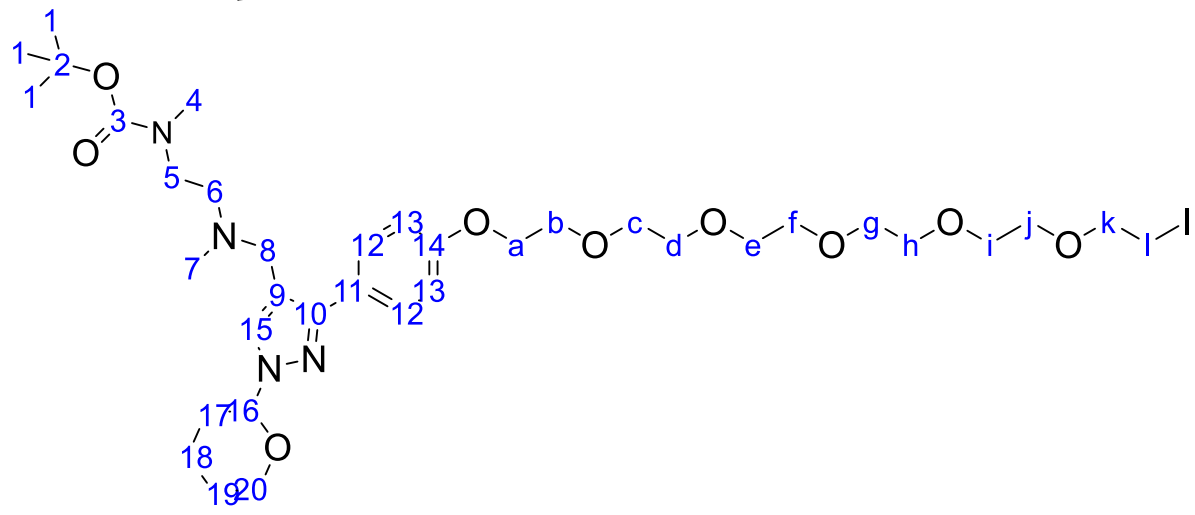

General method C was followed with PRMT1 ligand **4** (40 mg, 0.09 mmol) and linker **8l** (1,17-diiodo-3,6,9,12,15-pentaoxaheptadecane). The title compound was isolated as a pale yellow oil (28 mg, 38%). **^{1}H NMR (700 MHz, $CDCl_3$)** δ 7.72-7.57 (m, 3H, H12 and H15), 6.93 (d, *J*=8.7 Hz, 2H, H13), 5.36 (dd, *J*=9.9 and 2.5 Hz, 1H, H16), 4.14 (m, 2H, Ha), 4.07 (m, 1H, H20), 3.86 (m, 2H, Hb), 3.74-3.64 (m, 20H, Hc-k and H20 and H8), 3.47 (m, 1H, H8), 3.40 and 3.29 (m, 2H, H5), 3.24 (m, 2H, Hl), 2.80 (m, 3H, H4), 2.62 and 2.51 (m, 2H, H6), 2.26 and 2.24 (m, 3H, H7), 2.15-2.03 (m, 3H, H17 and H18), 1.71-1.65 (m, 2H, H18 and H19), 1.61-1.56 (m, 1H, H19), 1.42 and 1.41 (m, 9H, H1). Rotamers present. **^{13}C NMR (176 MHz, $CDCl_3$)** δ 158.5 (C14), 151.1 (d, C10), 129.6 (C12), 129.2 (C15), 114.6 (C13), 87.9 (C16), 79.5 (d, C2), 72.1-70.3 (Cc-Ck), 69.9 (Cb), 68.0 (C20), 67.5 (Ca), 55.0 and 53.8 (C6), 52.7 and 51.7 (C8), 46.8 and 45.8 (C5), 41.6 (d, C7), 34.8 (d, C4), 30.7 (C17), 28.5 (C1), 25.1 (C19), 22.7 (C18), 3.1 (Cl). Rotamers present. C3, C9, and C11 not identified. **HRMS (ESI)** *m/z* calcd for $C_{36}H_{59}IN_4O_9$ $[M+H]^+$: 819.3399; found 819.3420.

tert-butyl (2,6-dioxo-1-((2-(trimethylsilyl)ethoxy)methyl)piperidin-3-yl)carbamate, (11).

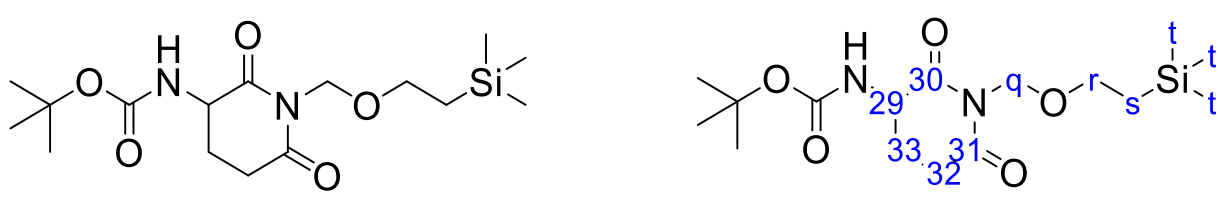

tert-butyl(2,6-dioxopiperidin-3-yl)carbamate (500 mg, 2.2 mmol, 1 eq) and DBU (490 µL, 3.3 mmol, 1.5 eq) were dissolved in DMF (10 mL). SEMCl (460 µL, 2.6 mmol, 1.2 eq) was added dropwise at 0°C and the reaction stirred at room temperature for 2 hr. The reaction was quenched with $NH_4Cl_{(aq, saturated)}$ and extracted with EtOAc (x3). The combined organic layers were washed with water, dried with Na_2SO_4 and the solvent removed *in vacuo* to give the title compound as a yellow oil (820 mg) which was used without further purification. **^{1}H NMR (500 MHz, d_6-DMSO)** δ 7.27 (d, *J*=8.4 Hz, 1H, 29-N<u>H</u>), 5.02 (m, 2H, Hq), 4.32 (m, 1H, H29), 3.51 (m, 2H, Hr), 2.84 (m, 1H, H32 or H33), 2.67 (m, 1H,

H32 or 33), 1.92 (m, 2H, H32 and 33), 1.40 (s, 9H, C(C<u>H</u>$_3$)$_3$), 0.82 (t, *J*=8.1 Hz, 2H, Hs), -0.02 (s, 9H, Ht). Data are in accordance with those previously reported in the literature[62].

2-(2,6-dioxo-1-((2-(trimethylsilyl)ethoxy)methyl)piperidin-3-yl)-4-hydroxyisoindoline-1,3-dione, (12).

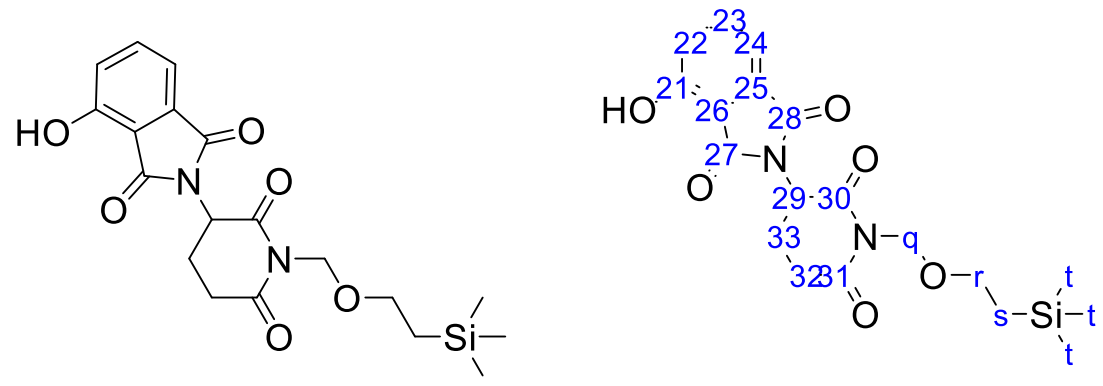

A suspension of **11** (47 mg, 0.13 mmol, 1 eq) and 4-hydroxyisobenzofuran-1,3-dione (21 mg, 0.13 mmol, 1 eq) in trifluoroethanol (2 mL) was heated under microwave conditions for 2 h at 150 °C. The solvent was removed *in vacuo* and the purified by automated column chromatography (C18 Gold, 5-95% MeCN in 0.1% $HCOOH_{(aq)}$ over 45 CV) to give a purple solid (30 mg). Considering residual EtOAc in ^{1}H NMR spectra, the yield is 56%. **^{1}H NMR (500 MHz, CDCl$_3$)** δ 11.22 (br s, 1H, 21-O<u>H</u>), 7.66 (dd, *J*=8.3 and 7.2 Hz, 1H, H23), 7.39 (d, *J*=7.1 Hz, 1H, H24), 7.25 (d, *J*=8.4 Hz, 1H, H22), 5.20 (dd, *J*=13.1 and 5.0 Hz, 1H, H29), 5.07 (s, 2H, Hq), 3.52 (m, ddd, *J*=17.1, 9.6 and 6.9 Hz, 2H, Hr), 3.02 and 2.78 (m, 2H, H32), 2.55 and 2.05 (m, 2H, H33), 0.84 (m, 2H, Hs), -0.02 (s, 9H, Ht). **^{13}C NMR (125 MHz, CDCl$_3$)** δ 171.7 and 170.0 (C30 and C31), 167.0 and 165.7 (C27 and 28), 155.6 (C21), 136.4 (C23), 133.1 (C26), 123.6 (C22), 114.3 (C25), 114.2 (C24), 68.3 (Cq), 66.0 (Cr), 49.2 (C29), 31.2 (C32), 21.0 (C33), 17.4 (Cs), -1.3 (Ct). **HRMS (ESI)** *m/z* calcd for $C_{19}H_{24}N_2O_6Si$ [M+Na]$^+$: 427.1296; found 427.1295.

PROTAC J

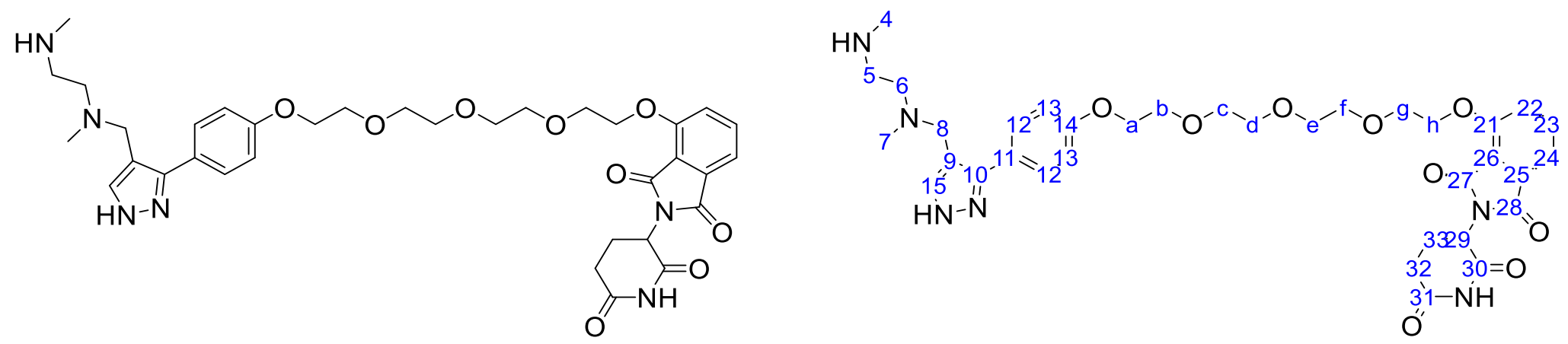

9j (31 mg, 0.04 mmol, 1 eq), CRBN ligand **12** (26 mg, 0.06 mmol, 1.5 eq) and Cs_2CO_3 (28 mg, 0.09 mmol, 2 eq) were dissolved in MeCN (1 mL) and heated at 60ºC overnight. The solvent was removed *in vacuo* and the residue dissolved in DCM (500 µL) and TFA (500 µL) was added dropwise and the mixture stirred at room temperature for 1 hr. The solvent was removed *in vacuo* and the residue purified by automated column chromatography (C18 Gold, 5-40% MeCN in 1% $HCOOH_{(aq)}$ over 45

CV). The product was then dissolved in MeCN (200 µL) and $NH_4OH_{(aq, 35\%)}$ (50 µL) was added and the reaction stirred for 5 min. The mixture was re-acidified by the dropwise addition of $HCl_{(aq, 37\%)}$ until pH 1 was reached. The solvent was the removed *in vacuo* and the residue purified by preparative HPLC (5-40% MeCN (0.05% TFA) in H_2O (0.1% TFA) over 20 min) and lyophilised to give the title compound as a colourless oil (2 mg, 7%).**^{1}H NMR (700 MHz, d_6-DMSO)** δ 11.10 (s, 1H, 30-N<u>H</u>), 10.0 (br s, 15-N<u>H</u>), 8.69 (br s, 2H, 4-N<u>H</u>$_2^+$), 7.84 (br s, 1H, H15), 7.80 (dd, *J*=8.4 and 7.3 Hz, 1H, H23), 7.53 (d, *J*=8.6 Hz, 1H, H22) 7.50 (d, *J*=8.0 Hz, 2H, H12), 7.46 (d, *J*=7.2 Hz, 1H, H24), 7.05 (d, *J*=8.5 Hz, 2H, H13), 5.08 (dd, *J*=12.9 and 5.5 Hz, 1H, H29), 4.38 (br s, 2H, H8), 4.34 (m, 2H, Hh), 4.12 (m, 2H, Ha), 3.80 (m, 2H, Hg), 3.75 (m, 2H, Hb), 3.65 (m, 2H, Hf), 3.58 (m, 2H, Hc), 3.55 (m, 4H, Hd and He), 3.21 and 3.13 (two br s, 4H, H5 and H6), 2.90-2.85 (m, 1H, H32), 2.67-2.52 (m, 8H, H32 and H33 and H4 and H7), 2.04-2.00 (m, 1H, H33). **^{13}C NMR (176 MHz, d_6-DMSO)** δ 172.8 and 169.9 (C30 and C31), 166.8 and 165.3 (C27 and C28), 158.6 (C14), 158.2 (q, $^2J_{C-F}$=33.4 Hz, CF_3<u>C</u>OO^-), 155.8 (C21), 137.0 (C23), 133.2 (C26), 129.5 (C12), 120.0 (C22), 116.3 (C25), 115.4 (C24), 114.8 (C13), 70.2 (Cf), 69.9-69.8 (Cc-Ce), 68.9 (Cb and Ch), 68.7 (Cg), 67.2 (Ca), 48.7 (C29), ~40.0 and 32.7 (C4 and C7), 30.9 (C32), 22.0 (C33). C5, C6, C8, C9, C10, C11 and C15 not identified. **HPLC** (5-95% MeCN (0.05% TFA) in H_2O (0.0.5% TFA) over 15 min) R_t= 6.25 min. Purity= 99%. **HRMS (ESI)** *m/z* calcd for $C_{35}H_{44}N_6O_9$ $[M+H]^+$: 693.3243; found 693.2247.

PROTAC K

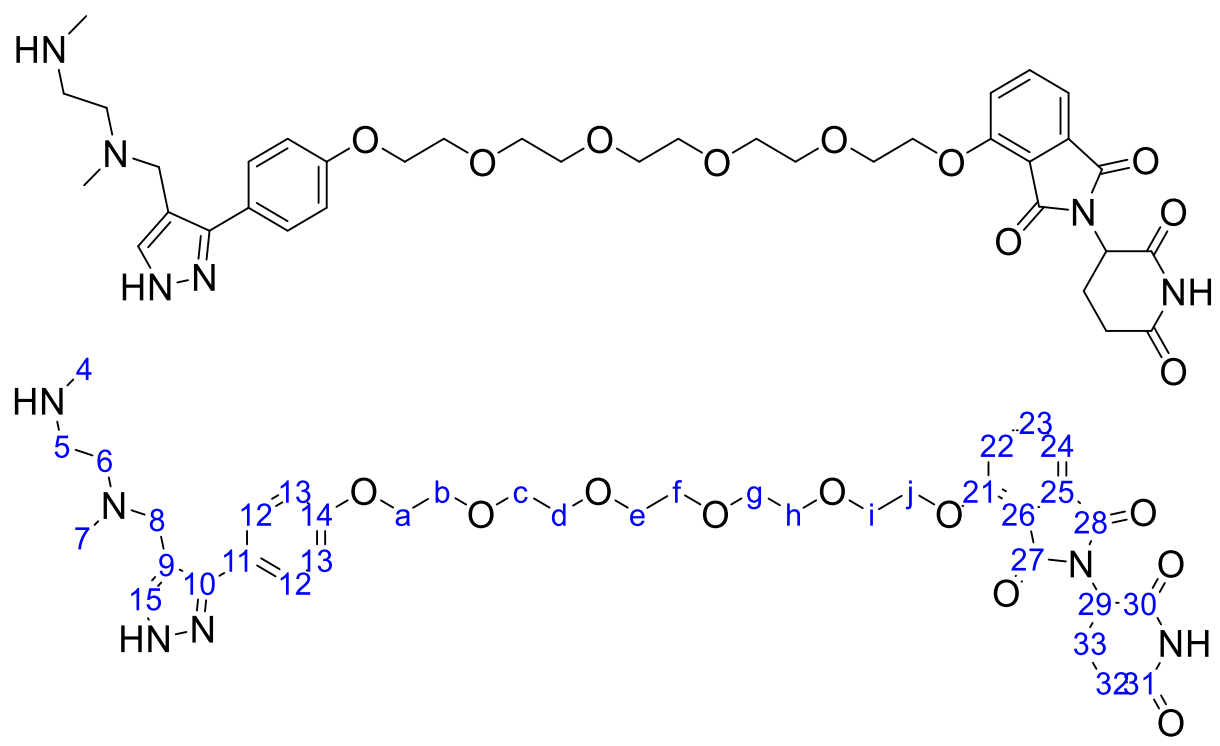

9k (30 mg, 0.04 mmol, 1 eq), CRBN ligand **12** (23 mg, 0.06 mmol, 1.5 eq) and Cs_2CO_3 (25 mg, 0.08 mmol, 2 eq) were dissolved in MeCN (1 mL) and heated at 60°C overnight. The solvent was removed *in vacuo* and the residue dissolved in DCM (500 µL) and TFA (500 µL) was added dropwise and the mixture stirred at room temperature for 1 hr. The solvent was removed *in vacuo* and the residue purified by automated column chromatography (C18 Gold, 5-60% MeCN in H_2O over 60 CV). The product was then dissolved in MeCN (200 µL) and $NH_4OH_{(aq, 35\%)}$ (50 µL) was added and the reaction stirred for 5 min. The mixture was re-acidified by the dropwise addition of $HCl_{(aq, 37\%)}$ until pH 1 was

reached. The solvent was the removed *in vacuo* and the residue purified by preparative HPLC (5-35% MeCN (0.05% TFA) in H_2O (0.1% TFA) over 30 min) and lyophilised to give the title compound as a colourless oil (1 mg, 4%). **^{1}H NMR (700 MHz, d$_6$-DMSO)** δ 11.10 (s, 1H, 30-N<u>H</u>), 10.12 (br s, 15-N<u>H</u>), 8.70 (br s, 2H, 4-N<u>H</u>$_2^+$), 7.80 (dd, *J*=8.5 and 7.2 Hz, 1H, H23), 7.52 (m, 3H, H12 and H22), 7.46 (d, *J*=7.2 Hz, 1H, H24), 7.04 (m, 2H, H13), 5.08 (dd, *J*=12.8 and 5.4 Hz, 1H, H29), 4.38 (br s, 2H, H8), 4.34 (m, 2H, Hj), 4.12 (m, 2H, Ha), 3.80 (m, 2H, Hi), 3.75 (m, 2H, Hb), 3.64 (m, 2H, Hh), 3.58 (m, 2H, Hc), 3.53 (m, 4H, Hd and Hg), 3.51 (m, 4H, He and Hf), 3.20 (m, 4H, H5 and H6), 2.91-2.85 (m, 1H, H32), 2.67-2.52 (m, 8H, H32 and H33 and H4 and H7), 2.04-2.01 (m, 1H, H33). H15 not identified. **^{13}C NMR (176 MHz, d$_6$-DMSO)** δ 172.8 and 169.9 (C30 and C31), 166.8 and 165.3 (C27 and C28), 158.0 (q, $^2J_{C-F}$=31.2 Hz, CF_3<u>C</u>OO^-), 155.8 (C21), 137.0 (C23), 133.2 (C26), 129.4 (C12), 120.0 (C22), 116.3 (C25), 115.4 (C24), 114.7 (C13), 70.2 (Ch), 69.9 (Cc), 69.8 (Cd-Cg), 68.9-68.7 (Cb, Ci, Cj), 67.2 (Ca), 48.7 (C29), ~40.4 and 32.7 (C4 and C7), 30.9 (C32), 22.0 (C33). C5, C6, C8, C9, C10, C11, C14 and C15 not identified. **HPLC** (5-95% MeCN (0.05% TFA) in H_2O (0.0.5% TFA) over 15 min) R_t= 6.41 min. Purity= 94%. **HRMS (ESI)** *m/z* calcd for $C_{37}H_{48}N_6O_{10}$ $[M+H]^+$:737.3505; found 737.3455.

PROTAC L

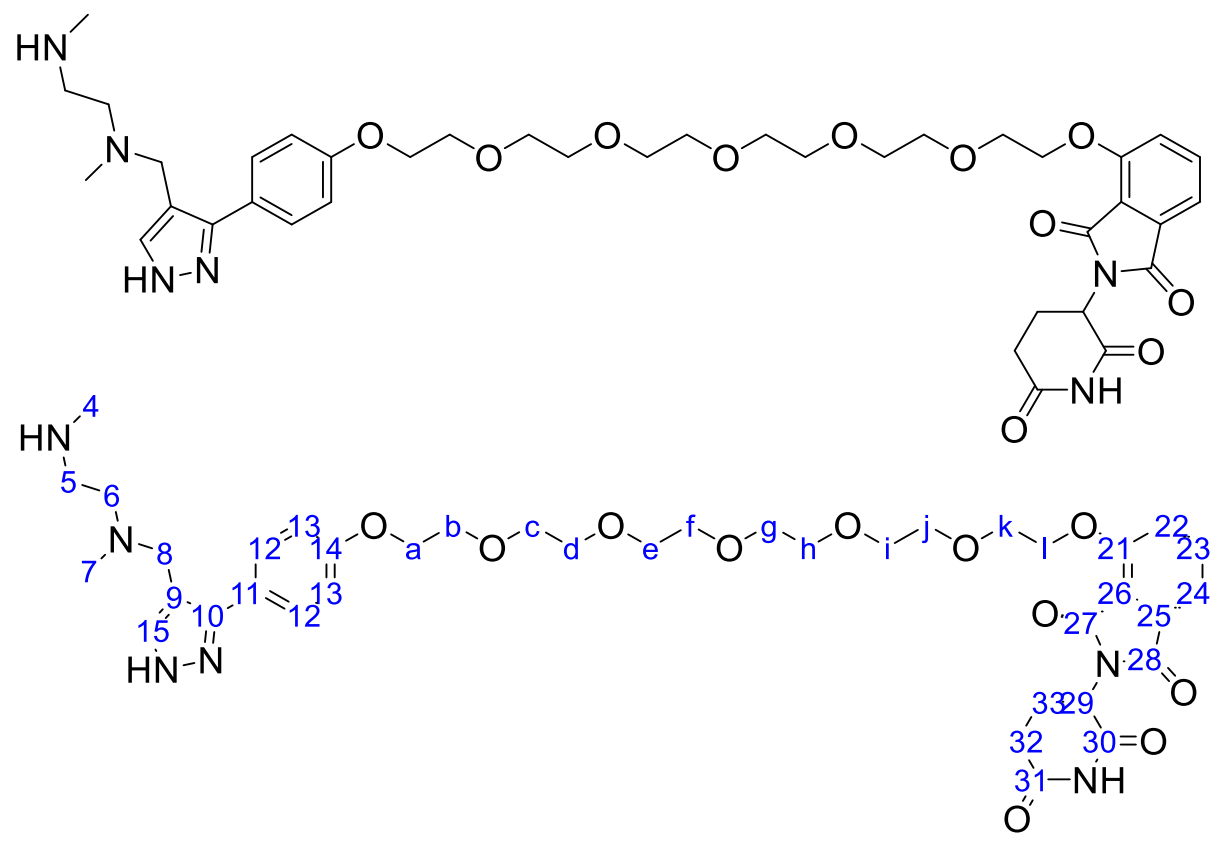

9l (9 mg, 0.01 mmol, 1 eq), CRBN ligand **12** (5 mg, 0.01 mmol, 1 eq) and Cs_2CO_3 (7 mg, 0.02 mmol, 2 eq) were dissolved in MeCN (1 mL) and heated at 80°C overnight. The solvent was removed *in vacuo* and the residue dissolved in DCM (1 mL) and TFA (1 mL) was added dropwise and the mixture stirred at room temperature for 1 hr. The solvent was removed *in vacuo* and the residue purified by preparative HPLC (5-40% MeCN (0.05% TFA) in H_2O (0.1% TFA) over 20 min) and lyophilised to give a white solid. The solid was then dissolved in MeCN (100 µL) and $NH_4OH_{(aq, 35\%)}$ (20 µL) was added and the reaction stirred for 5 min. The mixture was re-acidified by the dropwise addition of $HCl_{(aq, 37\%)}$ until pH 1 was reached. The solvent was the removed *in vacuo* and the residue purified by preparative HPLC (5-40% MeCN (0.05% TFA) in H_2O (0.1% TFA) over 20 min) and lyophilised to give

the title compound as a colourless oil (1 mg, 43%). **^{1}H NMR (700 MHz, d$_6$-DMSO)** δ 11.10 (s, 1H, 30-NH), 10.02 (br s, 15-NH), 8.67 (br s, 2H, 4-NH_2^+), 7.80 (dd, *J*=8.3 and 7.4 Hz, 1H, H23), 7.53 (d, *J*=8.5 Hz, 1H, H12), 7.51 (m, 1H, H22), 7.46 (d, *J*=7.2 Hz, 1H, H24), 7.05 (d, *J*=8.1 Hz, 2H, H13), 5.08 (dd, *J*=13.0 and 5.6 Hz, 1H, H29), 4.38 (br s, 2H, H8), 4.34 (m, 2H, Hl), 4.13 (m, 2H, Ha), 3.80 (m, 2H, Hk), 3.76 (m, 2H, Hb), 3.64 (m, 2H, Hj), 3.59 (m, 2H, Hc), 3.53 (m, 4H, Hd and Hi), 3.50 (m, 8H, He-Hh), 3.20 (m, 4H, H5 and H6), 2.91-2.85 (m, 1H, H32), 2.63-2.52 (m, 8H, H32 and H33 and H4 and H7), 2.03-2.01 (m, 1H, H33). H15 not identified. **^{13}C NMR (176 MHz, d$_6$-DMSO)** δ 172.8 and 169.9 (C30 and C31), 166.8 and 165.3 (C27 and C28), 155.8 (C21), 137.0 (C23), 133.2 (C26), 120.0 (C22), 116.3 (C25), 115.4 (C24), 114.7 (C13), 70.2 (Cj), 69.9 (Cc), 69.8-69.7 (Cd-Ci), 68.9 (Cb), 68.8 (Cl), 68.7 (Ck), 67.2 (Ca), 48.7 (C29), 40.4 (C4), 32.7 (C7), 30.9 (C32), 22.0 (C33). Peak indicated by HSQC δ 128.1 (C12), 50.6 (C8), 49.6 and 42.7 (C5 and C6). C9, C10, C11, C14 and C15 not identified. **HPLC** (5-95% MeCN (0.05% TFA) in H_2O (0.0.5% TFA) over 15 min) R_t= 6.52 min. Purity= 99%. **HRMS (ESI)** *m/z* calcd for $C_{39}H_{52}N_6O_{11}$ [M+H]$^+$: 781.3767; found 781.3748.

2-(2,6-dioxo-1-((2-(trimethylsilyl)ethoxy)methyl)piperidin-3-yl)-4-fluoroisoindoline-1,3-dione, (14).

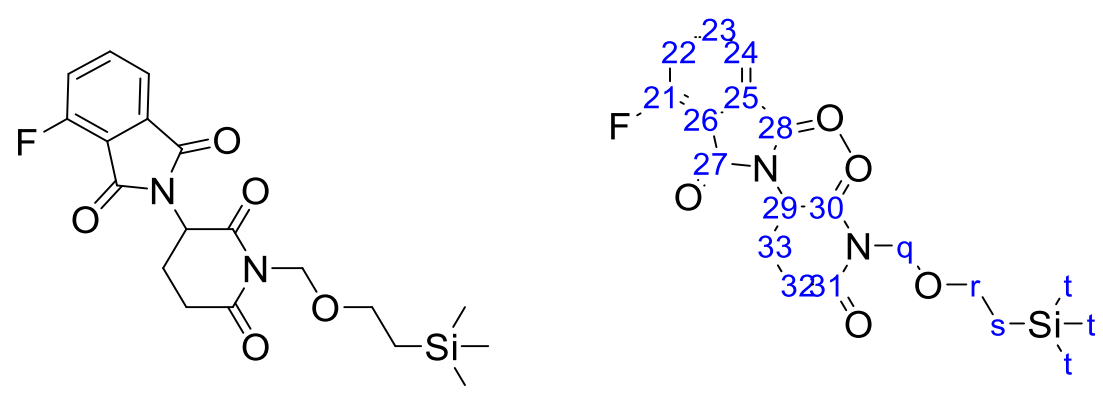

4-fluorothalidomide (210 mg, 0.68 mmol, 1 eq) and DBU (156 µL, 1.0 mmol, 1.5 eq) were dissolved in DMF (4 mL). SEMCl (150 µL, 0.8 mmol, 1.2 eq) was added dropwise at 0°C and the reaction stirred at room temperature for 2 hr. The reaction was quenched with $NH_4Cl_{(aq,\ saturated)}$ and extracted with EtOAc (x3). The combined organic layers were washed with brine, dried with Na_2SO_4 and the solvent removed *in vacuo* to give the title compound as a yellow oil (312 mg, quantitative). **^{1}H NMR (500 MHz, d$_6$-DMSO)** δ 7.96 (m, 1H, H23), 7.78 (d, *J*=7.2 Hz, 1H, H24), 7.74 (t, *J*=8.5 Hz, 1H, H22), 5.29 (dd, *J*=13.1 and 5.4 Hz, 1H, H29), 5.08 (s, 2H, Hq), 3.58-3.46 (m, 2H, Hr), 3.07-3.00 and 2.82-2.78 (m, 2H, H32), 2.59-2.50 and 2.12-2.07 (m, 1H, H33) 0.84 (m, 2H, Hs), -0.02 (s, 9H, Ht). **^{13}C NMR (125 MHz, d$_6$-DMSO)** δ 171.6 and 169.7 (C30 and C31), 166.0 and 163.9 (C27 and 28), 156.8 (d, $^1J_{C\text{-}F}$=262 Hz, C21), 138.1 (d, $^3J_{C\text{-}F}$=7.9 Hz, C23), 133.4 (C25), 123.0 (d, $^2J_{C\text{-}F}$=19.6 Hz, C22), 120.0 (d, $^4J_{C\text{-}F}$=3.0 Hz, C24), 117.0 (d, $^2J_{C\text{-}F}$=12.5 Hz, C26), 68.3 (Cq), 66.0 (Cr), 49.6 (C29), 31.1 (C32), 20.8 (C33), 17.4 (Cs), -1.4 (Ct). **HRMS (ESI)** *m/z* calcd for $C_{19}H_{23}FN_2O_5Si$ [M+Na]$^+$: 429.1252; found 429.1258.

2-(2,6-dioxo-1-((2-(trimethylsilyl)ethoxy)methyl)piperidin-3-yl)-4-((17-hydroxy-3,6,9,12,15-pentaoxaheptadecyl)amino)isoindoline-1,3-dione, (16m).

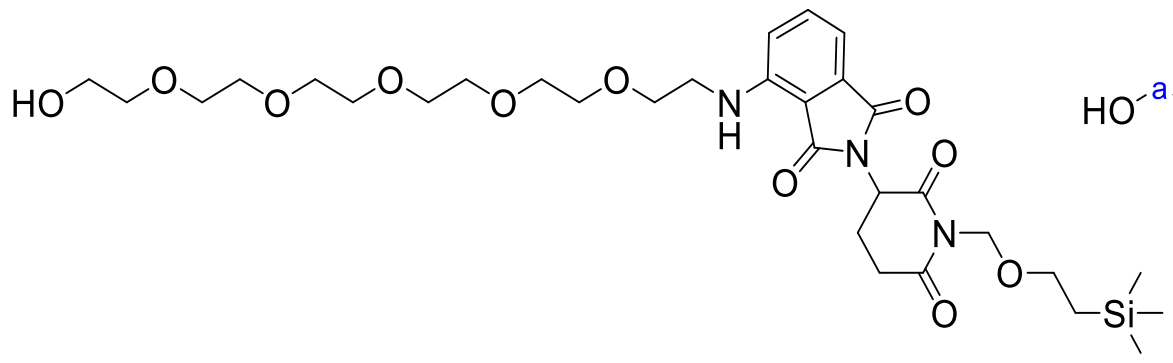 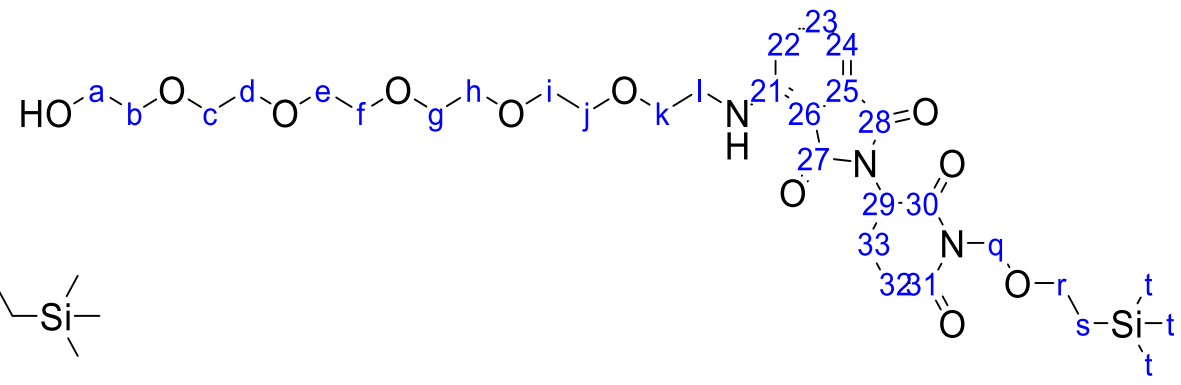

General method D was followed with CRBN ligand **14** (27 mg, 0.07 mmol) and linker {CAS: 39160-70-8} to give the title compound as a green oil (36 mg, 78%).**^{1}H NMR (500 MHz, CDCl$_3$)** δ 7.46 (dd, *J*=8.5 and 7.5 Hz, 1H, H23), 7.06 (d, *J*=6.8 Hz, 1H, H24), 6.92 (d, *J*=8.5 Hz, 1H, H22), 6.44 (m, 1H, 21-NH), 5.24 (s, 2H, Hq), 4.92 (m, 1H, H29), 3.70-3.56 (m, 24H, Hr and Ha-k), 3.46 (m, 2H, Hl), 3.01-2.92 (m, 1H, H32), 2.80-2.71 (m, 2H, H32 and H33), 2.09-2.05 (m, 1H, H33), 0.92 (m, 2H, Hs), -0.02 (s, 9H, Ht). a-OH not identified. **^{13}C NMR (125 MHz, CDCl$_3$)** δ 171.2 (C30 or C31), 169.4 (C27 or C28), 169.2 (C30 or C31), 167.8 (C27 or C28), 146.9 (C21), 136.1 (C23), 132.6 (C25), 116.9 (C22), 111.7 (C24), 110.4 (C26), 72.7-69.7 (Cb-Ck), 69.2 (Cq), 67.4 (Cr), 61.7 (Ca), 49.6 (C29), 42.5 (Cl), 32.1 (C32), 22.0 (C33), 18.1 (Cs), -1.3 (Ct). **HRMS (ESI)** *m/z* calcd for $C_{31}H_{49}N_3O_{11}Si$ $[M+H]^+$: 668.3209; found 668.3214.

2-(2,6-dioxo-1-((2-(trimethylsilyl)ethoxy)methyl)piperidin-3-yl)-4-((6-hydroxyhexyl)amino)isoindoline-1,3-dione, (16n).

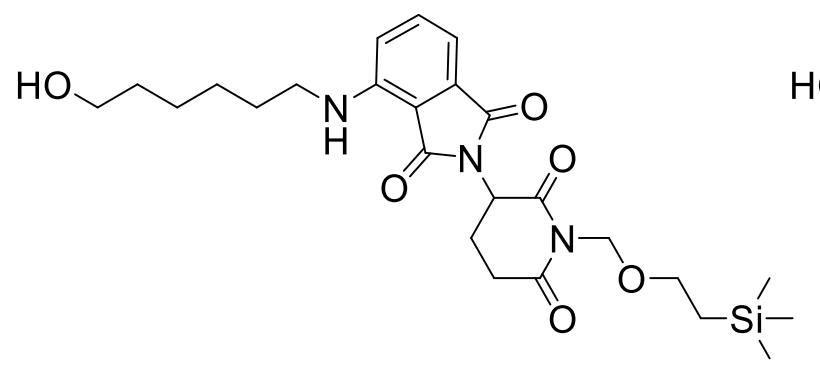 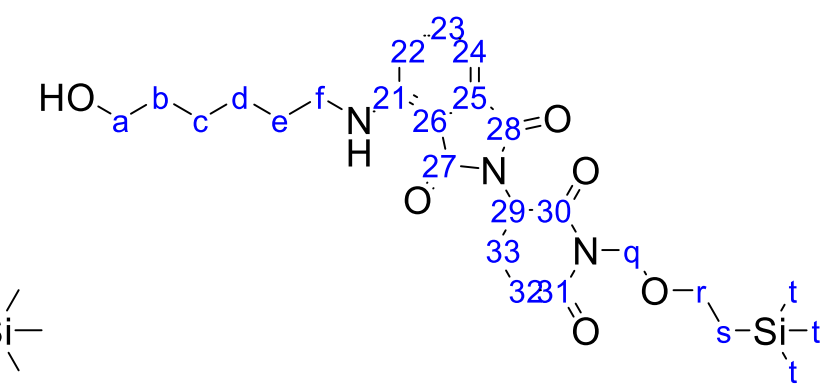

General method D was followed with CRBN ligand **14** (49 mg, 0.12 mmol) and 6-amino-1-hexanol to give the title compound as a yellow/green solid (29 mg, 47%).**^{1}H NMR (500 MHz, CDCl$_3$)** δ 7.47 (dd, *J*=8.5 and 7.2 Hz, 1H, H23), 7.07 (d, *J*=7.0 Hz, 1H, H24), 6.86 (d, *J*=8.5 Hz, 1H, H22),6.21 (m, 1H, 21-NH), 5.26 (s, 2H, Hq), 4.92 (dd, *J*=12.6 and 5.3 Hz, 1H, H29), 3.67-3.56 (m, 4H, Ha and Hr), 3.26 (m, 2H, Hf), 3.02-2.94 (m, 1H, H32), 2.81-2.72 (m, 2H, H32 and H33), 2.13-2.05 (m, 1H, H33), 1.67 (m, 2H, He), 1.58 (m, 2H, Hb), 1.48-1.39 (m, 4H, Hc and Hd), 0.94 (m, 2H, Hs), -0.01 (s, 9H, Ht). a-OH not identified. **^{13}C NMR (125 MHz, CDCl$_3$)** δ 171.2 (C30 or C31), 169.7 (C27 or C28), 169.3 (C30 or C31), 167.8 (C27 or C28), 147.1 (C21), 136.2 (C23), 132.7 (C25), 116.7 (C22), 111.5 (C24), 110.1 (C26), 69.3 (Cq), 67.5 (Cr), 62.9 (Ca), 49.7 (C29), 42.7 (Cf), 32.7 (Cb), 32.2 (C32), 29.3 and 26.7 and 25.6 (Cc, Cd

and Ce), 22.1 (C33), 18.2 (Cs), -1.3 (Ct). **HRMS (ESI)** *m/z* calcd for $C_{25}H_{37}N_3O_6Si$ $[M+Na]^+$: 526.2344 ; found 526.2346.

2-(2,6-dioxo-1-((2-(trimethylsilyl)ethoxy)methyl)piperidin-3-yl)-4-((10-hydroxydecyl)amino)isoindoline-1,3-dione, (16o).

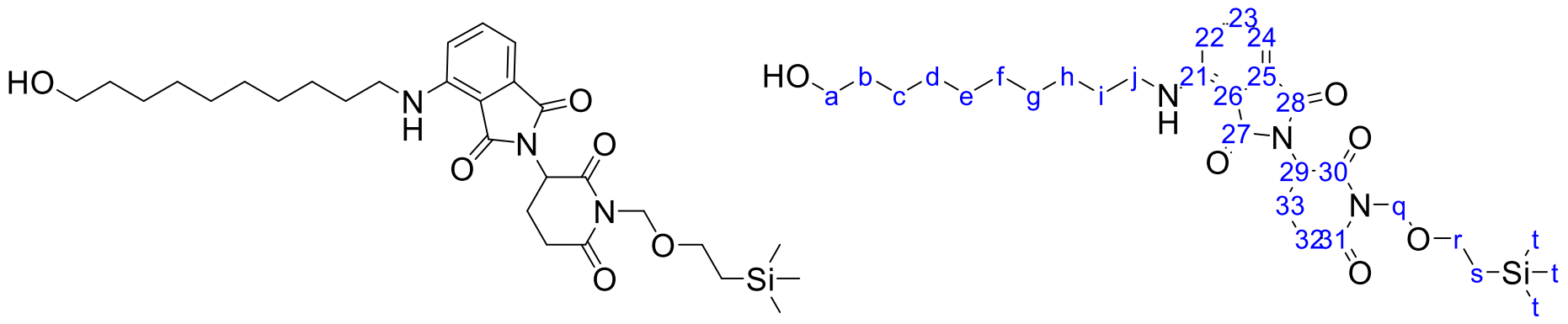

General method D was followed with CRBN ligand **14** (50 mg, 0.12 mmol) and 10-amino-1-decanol to give the title compound as a yellow/green oil (51 mg, 69%). **^{1}H NMR (400 MHz, $CDCl_3$)** δ 7.46 (dd, *J*=8.4 and 7.3 Hz, 1H, H23), 7.05 (d, *J*=7.1 Hz, 1H, H24), 6.86 (d, *J*=8.5 Hz, 1H, H22), 6.20 (t, *J*=4.9 Hz, 1H, 21-NH), 5.25 (s, 2H, Hq), 4.92 (dd, *J*=12.5 and 4.9 Hz, 1H, H29), 3.61 (m, 4H, Ha and Hr), 3.23 (m, 2H, Hj), 2.98-2.95 (m, 1H, H32), 2.81-2.73 (m, 2H, H32 and H33), 2.10-2.05 (m, 1H, H33), 1.63 (m, 2H, Hi), 1.54 (m, 2H, Hb), 1.41-1.29 (m, 12H, Hc-Hh), 0.93 (m, 2H, Hs), -0.01 (s, 9H, Ht). a-OH not identified. **^{13}C NMR (101 MHz, $CDCl_3$)** δ 171.2 (C30 or C31), 169.6 (C27 or C28), 169.3 (C30 or C31), 167.8 (C27 or C28), 147.1 (C21), 136.1 (C23), 132.6 (C25), 116.6 (C22), 111.3 (C24), 110.0 (C26), 69.2 (Cq), 67.4 (Cr), 63.1 (Ca), 49.6 (C29), 42.7 (Cj), 32.8 (Cb), 32.1 (C32), 29.6-25.8 (Cc-Ci), 22.1 (C33), 18.2 (Cs), -1.3 (Ct). **HRMS (ESI)** *m/z* calcd for $C_{29}H_{45}N_3O_6Si$ $[M+Na]^+$: 582.2970; found 582.2982.

PROTAC M

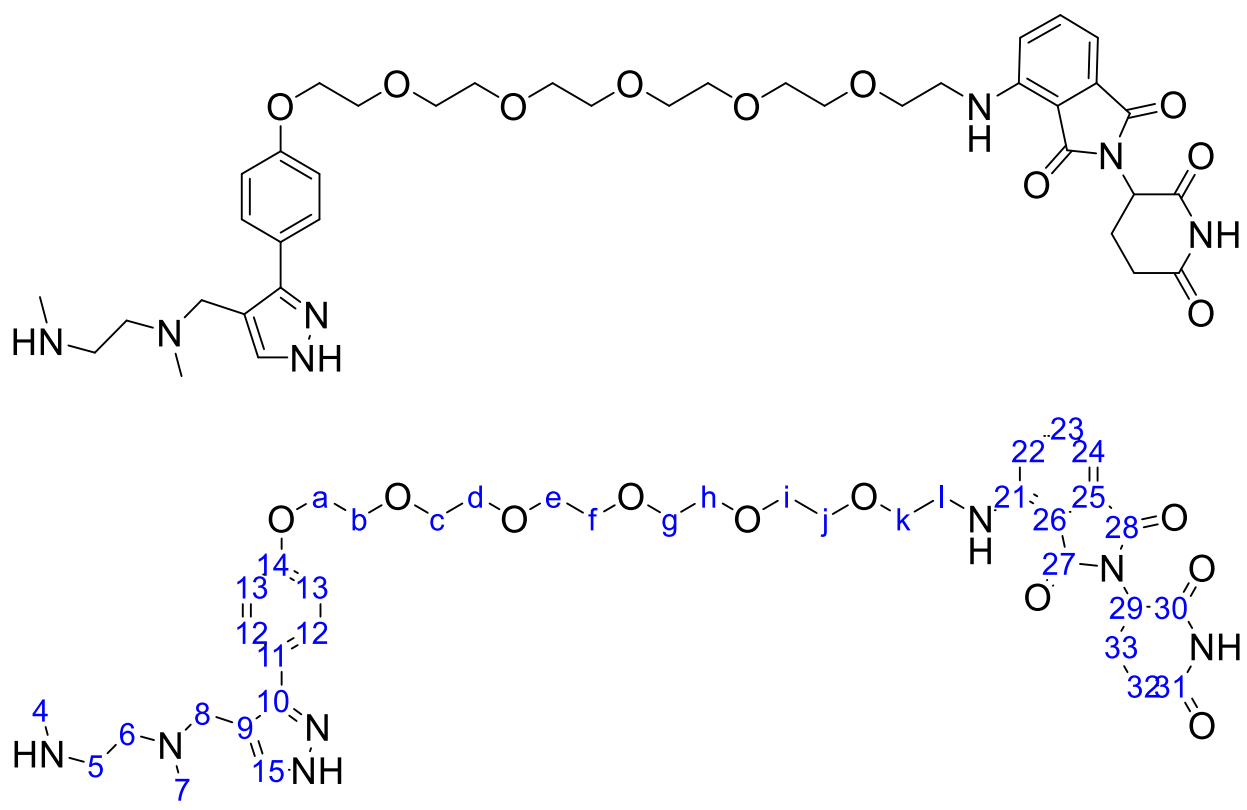

General method E was followed with **16m** (18 mg, 0.03 mmol) to give a yellow oil (19 mg) that was used without further purification. The resulting yellow oil, PRMT1 ligand **4** (12 mg, 0.03 mmol, 1 eq) and Cs_2CO_3 (16 mg, 0.05 mmol, 2 eq) were dissolved in MeCN (3 mL) and stirred at room

temperature overnight. The solvent was removed *in vacuo* and the residue dissolved in DCM (1 mL) and TFA (1 mL) was added dropwise and the mixture stirred at room temperature for 1 hr. The solvent was removed *in vacuo* and the residue dissolved in MeCN (600 µL) and $NH_4OH_{(aq, 35\%)}$ (200 µL) was added and the reaction stirred for 5 min. The mixture was re-acidified by the dropwise addition of $HCl_{(aq, 37\%)}$ until pH 1 was reached. The solvent was removed *in vacuo* and the residue purified by preparative HPLC (5-35% MeCN (0.05% TFA) in H_2O (0.1% TFA) over 20 min) and lyophilised to give the title compound as a green oil (7 mg, 38%). **^{1}H NMR (700 MHz, d$_6$-DMSO)** δ 11.09 (s, 1H, 30-N<u>H</u>), 10.06 (br s, 1H, 15-N<u>H</u>), 8.70 (br s, 2H, 4-NH_2^+), 7.85 (s, 1H, H15), 7.58 (dd, *J*=8.4 and 7.2 Hz, 1H, H23), 7.50 (d, *J*=8.5 Hz, 2H, H12), 7.14 (d, *J*=8.6 Hz, 1H, H22), 7.06 (d, *J*=8.6 Hz, 2H, H13), 7.07 (d, *J*=7.0 Hz, 1H, H24), 6.60 (m, 1H, 21-N<u>H</u>), 5.05 (dd, *J*=12.9 and 5.5 Hz, 1H, H29), 4.34 (br s, 2H, H8), 4.13 (m, 2H, Ha), 3.76 (m, 2H, Hb), 3.61 (m, 2H, Hk), 3.59-3.48 (m, 16H, Hc-Hj), 3.46 (m, 1H, Hl), 3.22 and 3.14 (two br s, 4H, H5 and H6), 2.89 (m, 1H, H32), 2.66-2.50 (m, 8H, H4 and H7 and H32 and H33), 2.02 (m, 1H, H33). **^{13}C NMR (176 MHz, d$_6$-DMSO)** δ 172.8 and 170.1 (C30 and C31), 168.9 and 167.3 (C27 and C28), 158.6 (C14), 158.3 (q, $^2J_{C-F}$=33.8 Hz, $CF_3\underline{C}OO^-$), 146.4 (C21), 136.2 (C23), 132.1 (C25), 129.5 (C12), 117.5 (C22), 116.3(q, $^1J_{C-F}$=295.2 Hz, $\underline{C}F_3COO^-$), 114.8 (C13), 110.7 (C24), 109.2 (C26), 69.9-69.9 (Cc-Cj), 68.9 (Cb and Ck), 67.2 (Ca), 50.2 (C8), 49.4 (C5 or C6), 48.6 (C29), 42.5 (C5 or C6), 41.7 (Cl), ~39.3 and 32.7 (C4 and C7), 31.0 (C32), 22.1 (C33). Peak indicated by HSQC δ 137.1 (C15). C9, C10 and C11 not identified. **HPLC** (5-95% MeCN (0.05% TFA) in H_2O (0.0.5% TFA) over 15 min) R_t = 6.95 min. Purity= 99%. **HRMS (ESI)** *m/z* calcd for $C_{39}H_{53}N_7O_{10}$ $[M+H]^+$: 780.3927; found 780.3923.

PROTAC N

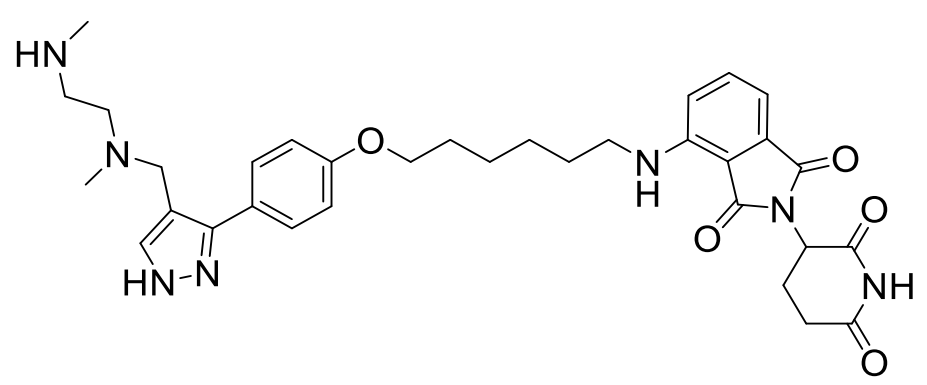

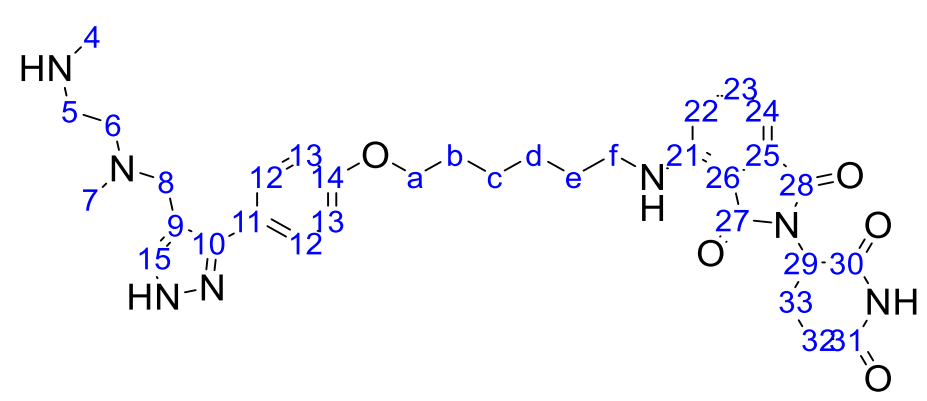

General method E was followed with **16n** (32 mg, 0.06 mmol) to give a yellow oil (80 mg) that was used without further purification. The resulting yellow oil, PRMT1 ligand **4** (28 mg, 0.06 mmol, 1 eq), Cs_2CO_3 (42 mg, 0.12 mmol, 2 eq) and NaI (9 mg, 0.06 mmol, 1 eq) were dissolved in MeCN (3 mL) and heated at 60°C overnight. The solvent was removed *in vacuo* and the residue dissolved in DCM (1 mL) and $HCl_{(aq, 37\%)}$ (1 mL) was added dropwise and the mixture stirred at room temperature for 1 hr. The solvent was removed *in vacuo* and the residue dissolved in MeCN (600 µL) and $NH_4OH_{(aq, 35\%)}$ (200 µL) was added and the reaction stirred for 5 min. The mixture was re-acidified by the dropwise addition of $HCl_{(aq, 37\%)}$ until pH 1 was reached. The mixture was then diluted with water and washed

with DCM. The solvent was removed from the aqueous layer under a stream of compressed air. The residue was then purified by preparative HPLC (5-60% MeCN (0.05% TFA) in H_2O (0.1% TFA) over 30 min) and lyophilised to give the title compound as a green oil (10 mg, 27%). **^{1}H NMR (700 MHz, d_6-DMSO)** δ 11.09 (s, 1H, 30-NH), 10.23 (br s, 1H, 15-NH), 8.82 (br s, 2H, 4-NH_2^+), 7.87 (s, 1H, H15), 7.58 (dd, *J*=8.5 and 7.1 Hz, 1H, H23), 7.48 (d, *J*=8.7 Hz, 2H, H12), 7.10 (d, *J*=8.6 Hz, 1H, H22), 7.03 (m, 3H, H13 and H24), 6.55 (br s, 1H, 21-NH), 5.05 (dd, *J*=12.9 and 5.5 Hz, 1H, H29), 4.34 (br s, 2H, H8), 4.01 (m, 2H, Ha), 3.32 (m, 2H, Hf), 3.25 and 3.18 (two br s, 4H, H5 and H6), 2.88 (m, 1H, H32), 2.62-2.51 (m, 8H, H4 and H7 and H32 and H33), 2.02 (m, 1H, H33), 1.75 (m, 2H, Hb), 1.62 (m, 2H, He), 1.48 (m, 2H, Hc), 1.43 (m, 2H, Hd). **^{13}C NMR (176 MHz, d_6-DMSO)** δ 172.8 and 170.1 (C30 and C31), 169.0 and 167.3 (C27 and C28), 158.8 (C14), 158.4 (q, $^2J_{C\text{-}F}$=34.8 Hz, $CF_3\underline{C}OO^-$), 146.4 (C21), 136.3 (C23), 132.2 (C25), 129.5 (C12), 117.2 (C22), 114.8 (C13), 110.4 (C24), 109.0 (C26), 67.5 (Ca), 50.1 (C8), 49.4 (C5 or C6), 48.5 (C29), 42.5 (C5 or C6), 41.8 (Cf), ~39.2 and 32.7 (C4 and C7), 31.0 (C32), 28.7 (Ce), 28.6 (Cb), 26.1 (Cd), 25.3 (Cc), 22.2 (C33). C9, C10, C11 and C15 not identified. **HPLC** (5-95% MeCN (0.05% TFA) in H_2O (0.0.5% TFA) over 15 min) R_t = 7.97 min. Purity= 99%. **HRMS (ESI)** *m/z* calcd for $C_{33}H_{41}N_7O_5$ $[M+H]^+$: 616.3242; found 616.3237.

PROTAC O

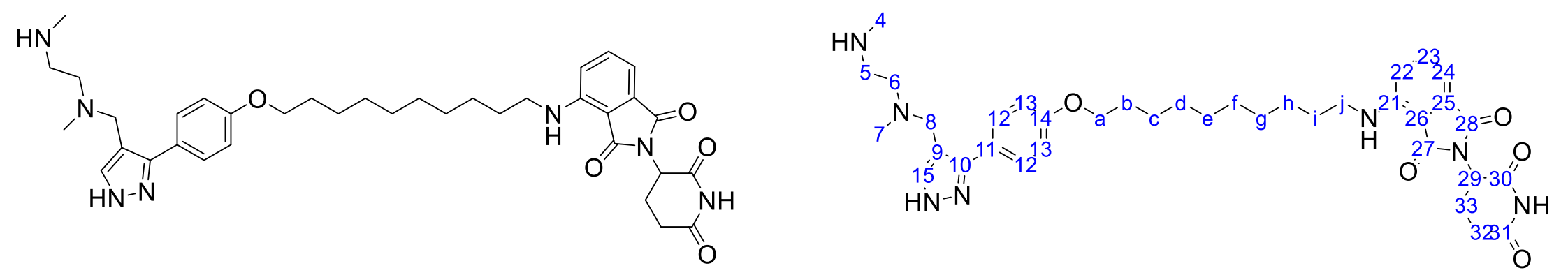

General method E was followed with **16o** (51 mg, 0.09 mmol, 1 eq) to give a yellow oil (90 mg) that was used without further purification. The resulting yellow oil, PRMT1 ligand **4** (38 mg, 0.09mmol, 1 eq), Cs_2CO_3 (59 mg, 0.18 mmol, 2 eq) and NaI (14 mg, 0.09 mmol, 1 eq) were dissolved in MeCN (3 mL) and heated at 60°C overnight. The solvent was removed *in vacuo* and the residue dissolved in DCM (1 mL) and TFA (1 mL) was added dropwise and the mixture stirred at room temperature for 1 hr. The solvent was removed *in vacuo* and the residue dissolved in MeCN (600 µL) and $NH_4OH_{(aq,\ 35\%)}$ (200 µL) was added and the reaction stirred for 5 min. The mixture was re-acidified by the dropwise addition of $HCl_{(aq,\ 37\%)}$ until pH 1 was reached. The mixture was then diluted with water and extracted with DCM. The organic layer was concentrated *in vacuo* and purified by preparative HPLC (5-60% MeCN (0.05% TFA) in H_2O (0.1% TFA) over 30 min) and lyophilised to give the title compound as a green oil (3 mg, 5%). **^{1}H NMR (500 MHz, d_6-DMSO)** δ 11.09 (s, 1H, 30-NH), 10.26 (br s, 1H, 15-NH), 8.83 (br s, 2H, 4-NH_2^+), 7.88 (s, 1H, H15), 7.58 (dd, *J*=8.5 and 7.1 Hz, 1H, H23), 7.48 (d, *J*=8.7 Hz, 2H,

H12), 7.09 (d, *J*=8.7 Hz, 1H, H22), 7.02 (m, 3H, H13 and H24), 6.52 (br s, 1H, 21-NH), 5.04 (dd, *J*=12.8 and 5.5 Hz, 1H, H29), 4.33 (br s, 2H, H8), 3.99 (m, 2H, Ha), 3.29 (m, 2H, Hj), 3.25 and 3.20 (two br s, 4H, H5 and H6), 2.88 (m, 1H, H32), 2.64-2.52 (m, 8H, H4 and H7 and H32 and H33), 2.02 (m, 1H, H33), 1.72 (m, 2H, Hb), 1.57 (m, 2H, Hi), 1.41 (m, 2H, Hc), 1.31 (m, 10H, Hd-h). **^{13}C NMR (125 MHz, d_6-DMSO)** δ 172.9 and 170.1 (C30 and C31), 169.0 and 167.3 (C27 and C28), 158.9 (C14), 158.3 (q, $^2J_{C\text{-}F}$=33.9 Hz, $CF_3\underline{C}OO^-$), 146.5 (C21), 136.3 (C23), 132.2 (C25), 129.6 (C12), 117.2 (C22), 116.2 (q, $^1J_{C\text{-}F}$=274.4 Hz, $\underline{C}F_3COO^-$), 114.8 (C13), 110.4 (C24), 109.0 (C26), 67.6 (Ca), 50.2 (C8), 49.4 (C5 or C6), 48.6 (C29), 42.6 (C5 or C6), 41.9 (Cj), ~39.5 and 32.7 (C4 and C7), 31.0 (C32), 29.0-28.8 (Ce-Ch), 28.7 (Ci and Cb), 26.1 (Cd), 25.3 (Cc), 22.2 (C33). Peak indicated by HSQC δ 137.7 (C15). C9, C10 and C11 not identified. **HPLC** (5-95% MeCN (0.05% TFA) in H_2O (0.0.5% TFA) over 15 min) R_t = 9.67 min. Purity= 95%. **HRMS (ESI)** *m/z* calcd for $C_{37}H_{49}N_7O_5$ $[M+H]^+$: 671.3790; found 671.3757.

5.3.3 PRMT1 Ligand 4

Synthetic Scheme

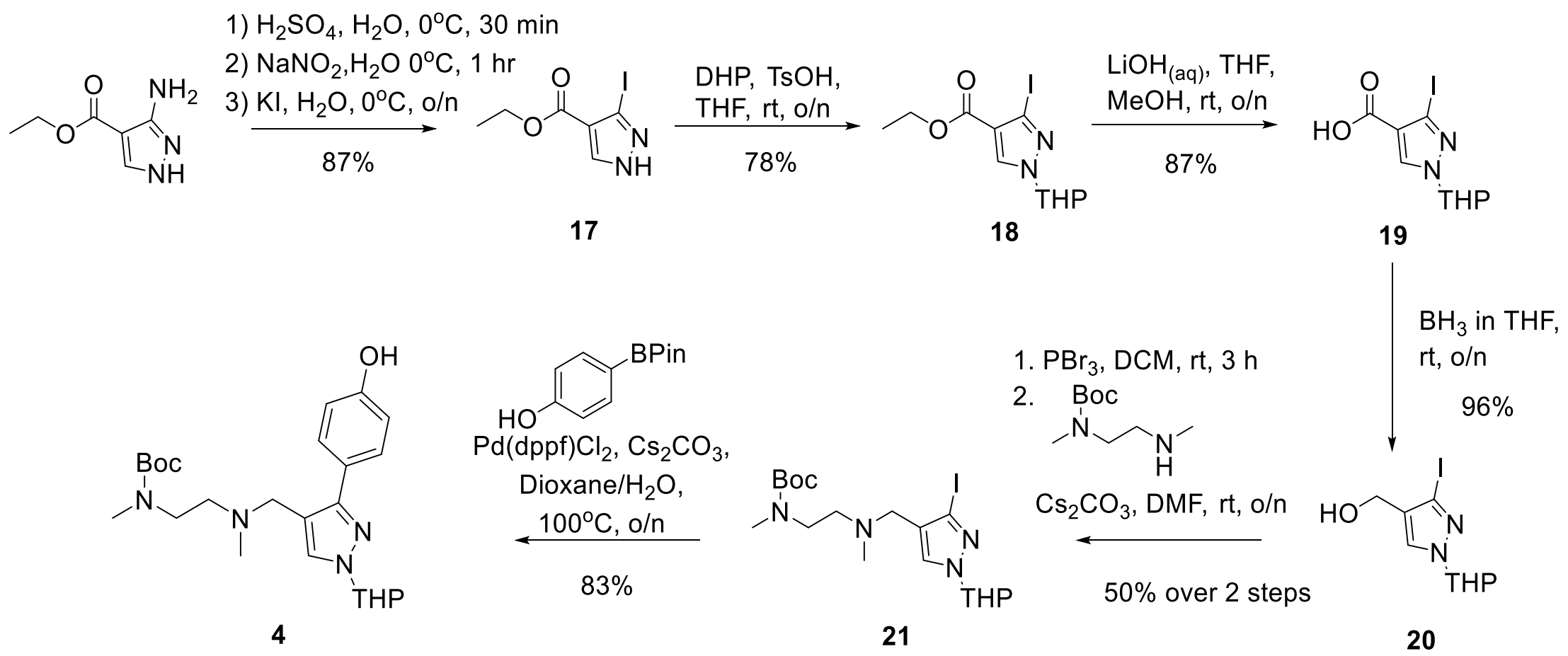

Ethyl 3-iodo-1*H*-pyrazole-4-carboxylate, (17).

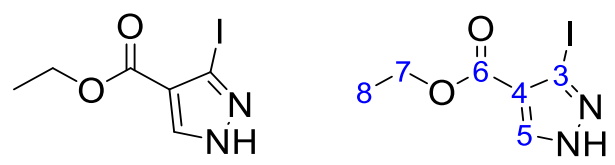

Ethyl 3-amino-1*H*-pyrazole-4-carboxylate (2.5 g, 16.1 mmol, 1 eq) was added portionwise to a solution of $H_2SO_{4(aq,\ 95\text{-}99\%)}$ (5.3 mL) in water (4.4 mL) at 0°C. The mixture was stirred at 0°C for 30 min. A solution of $NaNO_2$ (1.7 g, 24.2 mmol, 1.5 eq) in water (7.4 mL) was added dropwise at 0°C, and the mixture was stirred at 0°C for an additional hour. Next a solution of KI (5.4 g, 32.3 mmol, 2 eq) in

water (7.4 mL) was added dropwise at 0°C. The mixture was stirred at room temperature overnight and then extracted with EtOAc (x3) and the combined organic layers were washed with $Na_2SO_{3(aq, saturated)}$ (x3) and brine. The resulting organic layer was dried with Na_2SO_4 and the solvent removed *in vacuo* to give the title compound as a yellow solid (3.7 g, 87%). **^{1}H NMR (500 MHz, d_6-DMSO)** δ 13.76 (s, 1H, NH), 8.27 (s, 1H, H5), 4.21 (q, *J*= 7.1 Hz, 2H, H7), 1.27 (t, *J*=7.0 Hz, 3H, H8. Data are in accordance with those reported previously in literature[63].

Ethyl 3-iodo-1-(oxan-2-yl)-1*H*-pyrazole-4-carboxylate, (18).

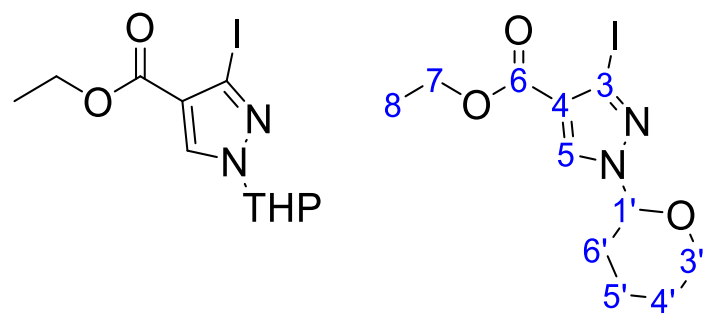

17 (7.2 g, 27.0 mmol, 1 eq) and TsOH (460 mg. 2.7 mmol, 0.1 eq) were dissolved in THF (40 mL). 3,4-dihydro-H-pyran (7.4 mL, 81.2 mmol, 3 eq) was added dropwise at 0°C and the resulting solution stirred overnight at room temperature. The solvent was then removed *in vacuo* and the residue diluted with water and extracted with EtOAc (x2). The combined organic layers were washed with brine (x2), dried with Na_2SO_4 and the solvent removed *in vacuo*. The residue was purified by silica gel chromatography (0-15% EtOAc in PE) to give the title compound as an orange oil (7.4 g, 78%). **^{1}H NMR (500 MHz, d_6-DMSO)** δ 8.40 (s, 1H, H5), 5.45 (dd, *J*=10.0 and 2.4 Hz, 1H, H1'), 4.22 (q, *J*=7.0 Hz, 2H, H7), 3.94-3.90 and 3.63-3.58 (m, 2H, H3'), 2.11-2.03 (m, 1H, H6'), 1.92-1.87 (m, 2H, H6' and H5'), 1.68-1.59 (m, 1H, H5'), 1.55-1.50 (m, 2H, H4'), 1.27 (t, *J*=7.0 Hz, 3H, H8). Data are in accordance with those reported previously in literature[63].

3-iodo-1-(tetrahydro-2*H*-pyran-2-yl)-1*H*-pyrazole-4-carboxylic acid, (19).

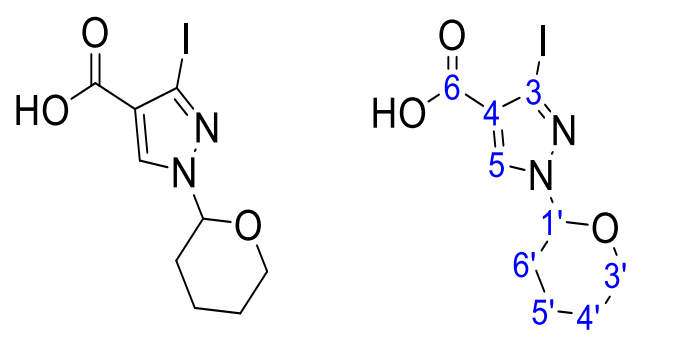

18 (3.0 g, 8.6 mmol, 1 eq) was dissolved in THF (6 mL) and methanol (6 mL). LiOH (600 mg, 25.7 mmol, 3 eq) in water (4.5 mL) was added dropwise with stirring at 0°C and the resulting solution stirred overnight at room temperature. The solvent was removed *in vacuo* and the residue diluted with $HCl_{(aq, 1M)}$ and extracted with DCM (x3). The combined organic layers were washed with water,

dried with Na_2SO_4 and the solvent removed *in vacuo* to give the title compound as a white solid (2.4 g, 87%). **^{1}H NMR (500 MHz, d$_6$-DMSO)** δ 12.59 (s, 1H, 6-COOH) 8.32 (s, 1H, H5), 5.43 (dd, *J*= 2.4 and 10.0 Hz, 1H, H1’), 3.92 and 3.64-3.58 (m 2H, H3’), 2.12-2.02 (m, 1H, H6’), 1.94-1.86 (m, 2H, H6’ and H5’), 1.68-1.58 (m, 1H, H5’), 1.55-1.49 (m, 2H, H4’). Data are in accordance with those reported previously in literature[63].

3-iodo-1-(oxan-2-yl)-1*H*-pyrazol-4-yl]methanol. (20).

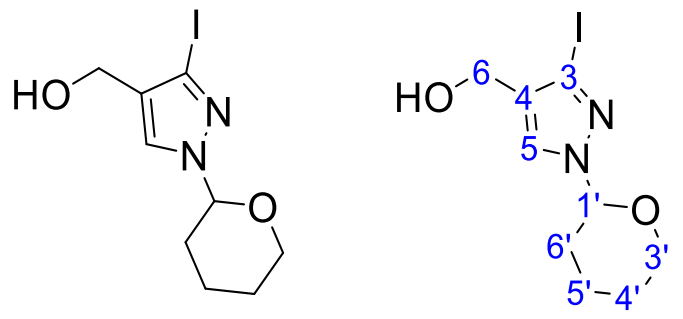

To **19** (2.3 g, 7 mmol, 1 eq) was added a 1M solution of BH_3 in THF (35 mL, 35 mmol, 5 eq) dropwise at 0°C and the resulting solution was stirred overnight at room temperature. The resulting mixture was quenched with $NH_4Cl_{(aq,\ saturated)}$, diluted with water and extracted with EtOAc (x3). The combined organic layers were washed with brine, dried with Na_2SO_4 and the solvent removed *in vacuo* to give the title compound as a pale yellow oil (2.2 g, 96%). **^{1}H NMR (500 MHz, d$_6$-DMSO)** δ 7.73 (s, 1H, H5), 5.35 (dd, *J*=10.1 and 2.5 Hz, 1H, H1’), 4.91 (t, *J*=5.2 Hz, 6-OH), 4.20 (d, *J*=5.0 Hz, 2H, H6), 3.91-3.87 and 3.62-3.57 (m, 2H, H3’), 2.07-2.00 (m, 1H, H6’), 1.93-1.83 (m, 2H, H6’ and H5’), 1.68-1.59 (m, 1H, H5’), 1.53-1.46 (m, 2H, H4’). Data are in accordance with those reported previously in literature[63].

***tert*-butyl (2-((3-iodo-1-(tetrahydro-2*H*-pyran-2-yl)-1*H*-pyrazol-4-yl)methyl)(methyl)amino)ethyl) (methyl)carbamate, (21).**

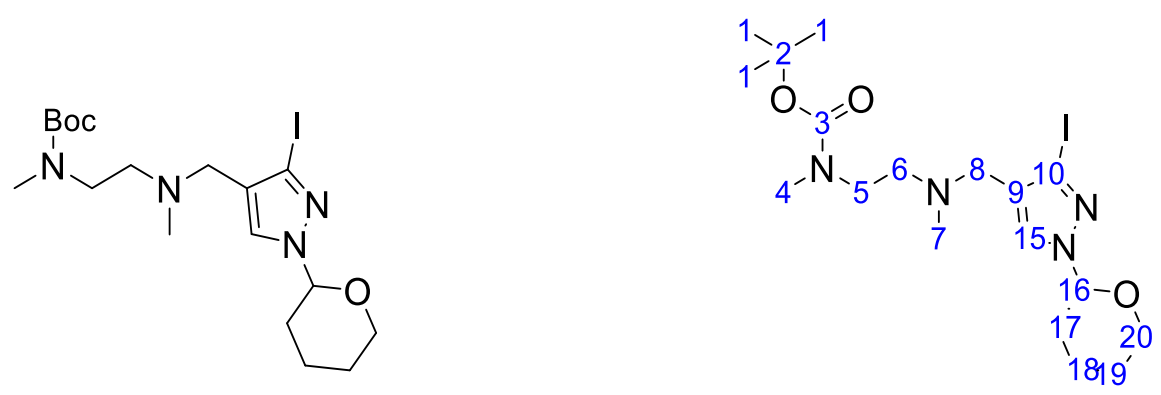

To a solution of **20** (1.0 g, 3.3 mmol, 1 eq) in DCM (20 mL) was added PBr_3 (780 µL, 9.8 mmol, 3 eq) dropwise at 0°C. The solution was stirred at room temperature for 3 hr. The reaction was quenched with $NH_4Cl_{(aq,\ saturated)}$ and extracted with DCM (x5). The combined organic layers were washed with brine, dried with Na_2SO_4 and the solvent removed *in vacuo* to give a pale yellow oil (750 mg). This oil (750 mg), *N*-(tert-butoxycarbonyl)-*N*-methyl-2-(methylamino)ethylamine (1.2 g, 6.6 mmol, 2 eq) and K_2CO_3 (450 mg, 3.3 mol, 1 eq) were dissolved in DMF (20 mL) and stirred overnight at room

temperature. The resulting mixture was diluted with water and extracted with EtOAc (x2). The combined organic layers were washed with brine, dried with Na_2SO_4 and the solvent removed *in vacuo*. The residue was purified by silica gel chromatography (0-4% MeOH in DCM) to give the title compound as a pale yellow oil (790 mg, 50% over two steps). **^{1}H NMR (500 MHz, d$_6$-DMSO)** δ 7.70 and 7.69 (s, 1H, H15), 5.34 (dd, *J*=10.3 and 2.3 Hz, 1H, H16), 3.90-3.57 (m, 2H, H20), 3.25 (m, 4H, H8 and H5), 2.77 and 2.73 (s, 3H, H4), 2.40 (t, *J*=6.7 Hz, 2H, H6), 2.15 and 2.13 (s, 3H, H7), 2.07- 2.00 (m, 1H, H17), 1.91 -1.85 (m, 2H, H17 and H18), 1.65-1.60 (m, 1H, H18), 1.52 -1.48 (m, 2H, H19), 1.38 and 1.35 (s, 9H, H1). Rotamers present. Data are in accordance with those reported previously in literature[63].

***tert*-butyl (2-(((3-(4-hydroxyphenyl)-1-(tetrahydro-2*H*-pyran-2-yl)-1*H*-pyrazol-4-yl)methyl)(methyl)amino)ethyl)(methyl)carbamate, (4).**

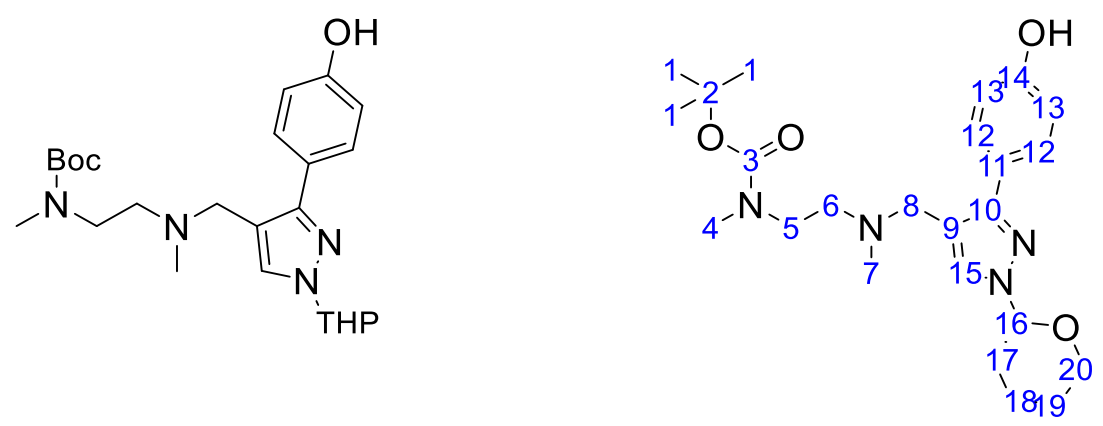

21 (2.3 g, 4.8 mmol, 1 eq), 4-hydroxybenzeneboronic acid pinacol ester (2.1 g, 9.5 mmol, 2 eq) and Cs_2CO_3 (4.7 g, 14.3 mmol, 3 eq) were dissolved in dioxane (15 mL) and water (1.5 mL). Pd(dppf)Cl_2 (170 mg, 0.2 mmol, 0.05 eq) was then added and the resulting mixture heated overnight at 100°C. The resulting solution then was cooled to room temperature, diluted with water and extracted with DCM (x3). The combined organic layers were washed with water, dried with Na_2SO_4 and the solvent removed *in vacuo*. The residue was purified by automated column chromatography (C18 Gold, 5-95% MeCN in 0.1 M $NH_4OH_{(aq)}$ over 40 CV) to give a dark brown solid (1.75 g, 83%). **^{1}H NMR (400 MHz, d$_6$-DMSO)** δ 9.42 (s, 1H, 14-OH), 7.75 (s, 1H, H15), 7.63 (d, *J*=8.3 Hz, 2H, H12), 6.79 (d, *J*=8.5 Hz, 2H, H13), 5.34 (dd, *J*=9.5 and 2.1 Hz, 1H, H16), 3.92 and 3.62 (m, 2H, H20), 3.32 (s, 2H, H8), 3.28 (m, 2H, H5), 2.74 and 2.72 (s, 3H, H4), 2.46 (t, *J*=6.3 Hz, 2H, H6), 2.16 (s, 3H, H7), 2.10-2.07 (m, 1H, H17), 1.95-1.89 (m, 2H, H17 and H18), 1.69-1.64 (m, 1H, H18), 1.56-1.50 (m, 2H, H19), 1.36 and 1.34 (s, 9H, H1). Rotamers present. **^{13}C NMR (100.6 MHz, d$_6$-DMSO)** δ 156.8 (C14), 154.7 (C3), 149.6 (C10), 130.3 (C15), 128.9 (C12), 124.6 (C11), 115.2 (C9), 115.1 (C13), 86.7 (C16), 78.2 (C2), 66.8 (C20), 54.5 and 54.2 (C6), 52.3 (C8), 45.9 and 45.2 (C5) 41.2 (C7), 33.9 (C4), 29.8 (C17), 28.0 (C1), 24.6 (C19), 22.1 (C18). Rotamers present. **HRMS (ESI)** *m/z* calcd for $C_{24}H_{36}N_4O_4$ $[M+H]^+$: 445.2809; found 445.2822.

4-(4-((methyl(2-(methylamino)ethyl)amino)methyl)-1*H*-pyrazol-3-yl)phenol, (Deprotected-4).

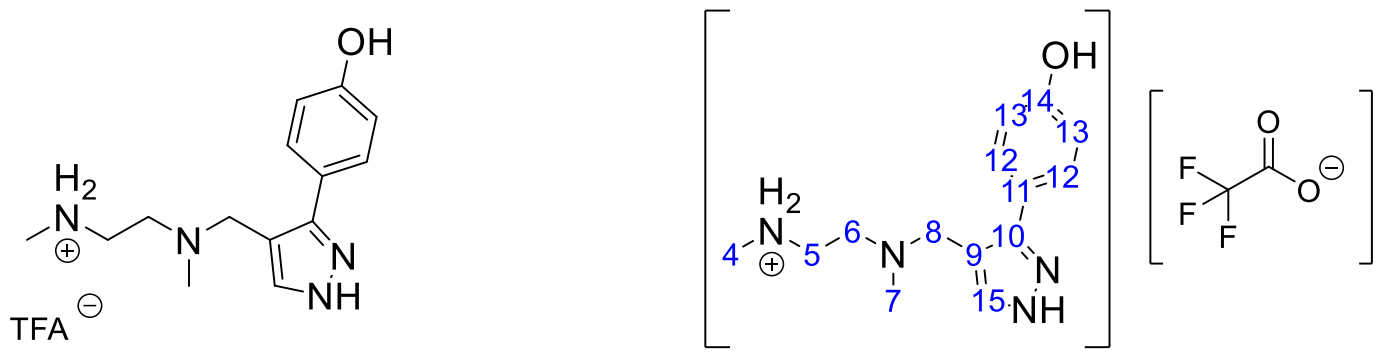

4 (37 mg, 0.08 mmol, 1 eq) was diluted in MeOH (1 mL) and $HCl_{(aq,\ 37\%)}$ (1 mL) was added dropwise and the mixture stirred at room temperature for 1 hr. The solvent was then removed *in vacuo* and the residue and purified by preparative HPLC (5-20% MeCN (0.05% TFA) in H_2O (0.1% TFA) over 10 min) and lyophilised to give the title compound as a pale orange solid (11.5 mg, 54%). **^{1}H NMR (500 MHz, d$_6$-DMSO)** δ 10.30 and 9.82 (two br s, 2H, 13-O<u>H</u> and 15-N<u>H</u>), 8.87 (s, 2H, 4-N<u>H</u>$_2^+$), 7.83 (s, 1H, H15), 7.37 (d, *J*=8.6 Hz, 2H, H12), 6.88 (d, *J*=6.8 Hz, 2H, H13), 4.33 (s, 2H, H8), 3.29 (m, 4H, H5 and H6), 2.63 (s, 3H, H4), 2.55 (s, 3H, H7). **^{13}C NMR (125 MHz, d$_6$-DMSO)** δ 158.6 (q, $^2J_{C\text{-}F}$ = 33.0 Hz, $CF_3\underline{C}OO^-$), 157.9 (C14), 129.6 (C12), 116.5 (q, $^1J_{C\text{-}F}$ =295.7 Hz, $\underline{C}F_3COO^-$), 115.7 (C13), 50.1 (C8), 49.3 and 42.5 (C5 and C6), 39.1 (C4), 32.6 (C7). Peaks indicated by HSQC δ 137.2 (C15). Peaks indicated by HMBC δ 147.1 (C10), 121.1 (C11), 105.4 (C9). **HRMS (ESI)** *m/z* calcd for $C_{14}H_{20}N_4O$[M+H]$^+$: 261.1710; found 261.1732.

5.3.4 VHL ligand 1

Synthesised in-house by the procedure published in Steinebach *et al.*[64].

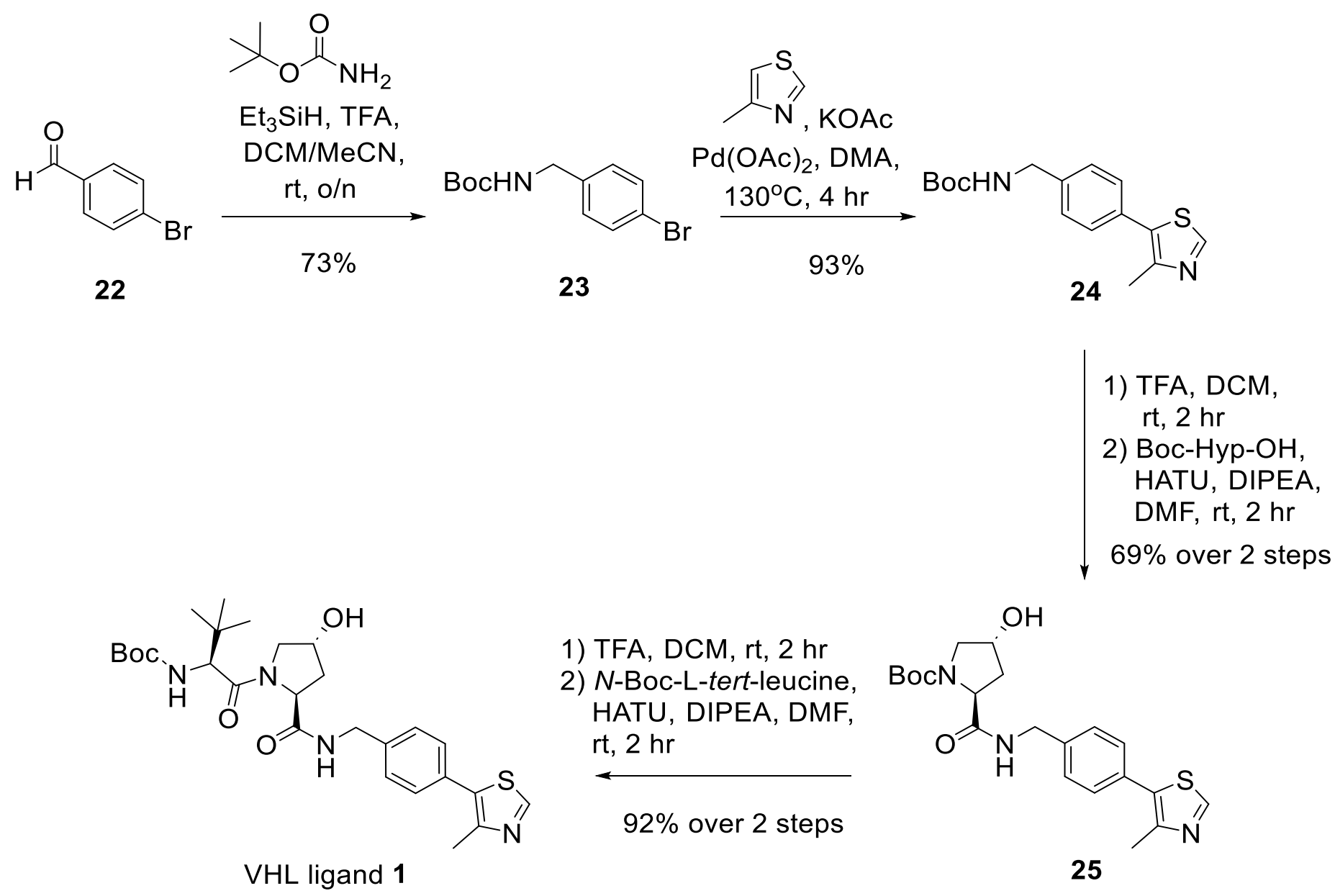

7. NMR Spectra

(3a)

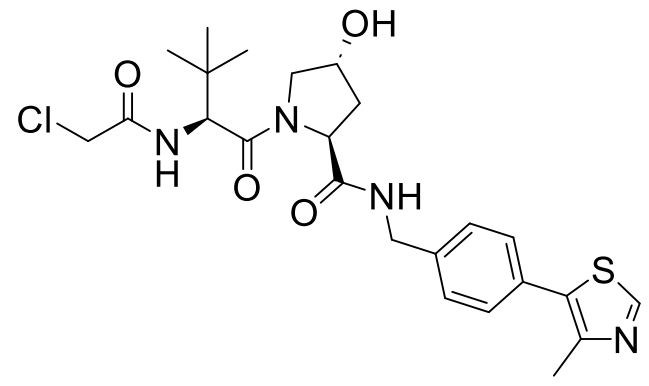

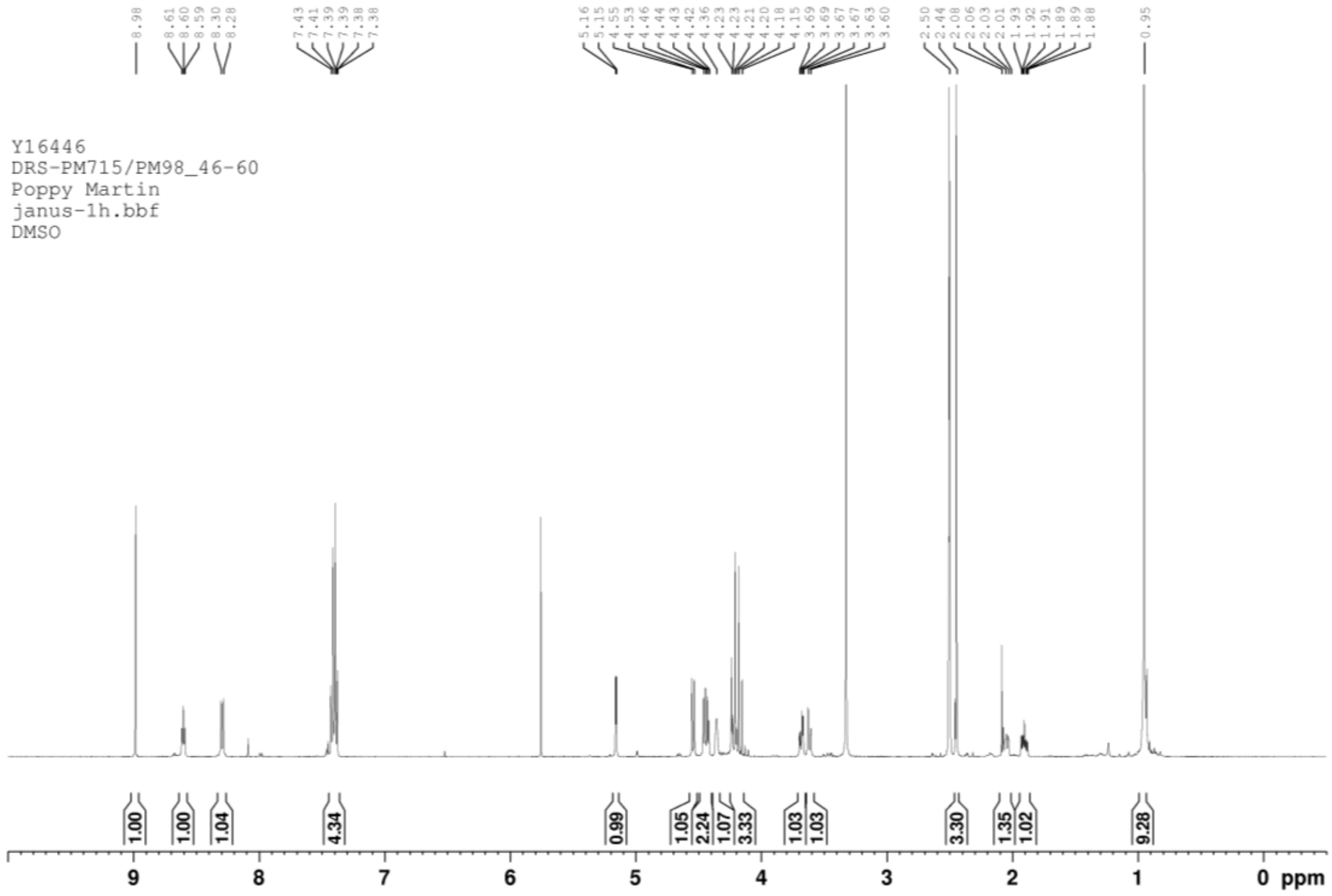

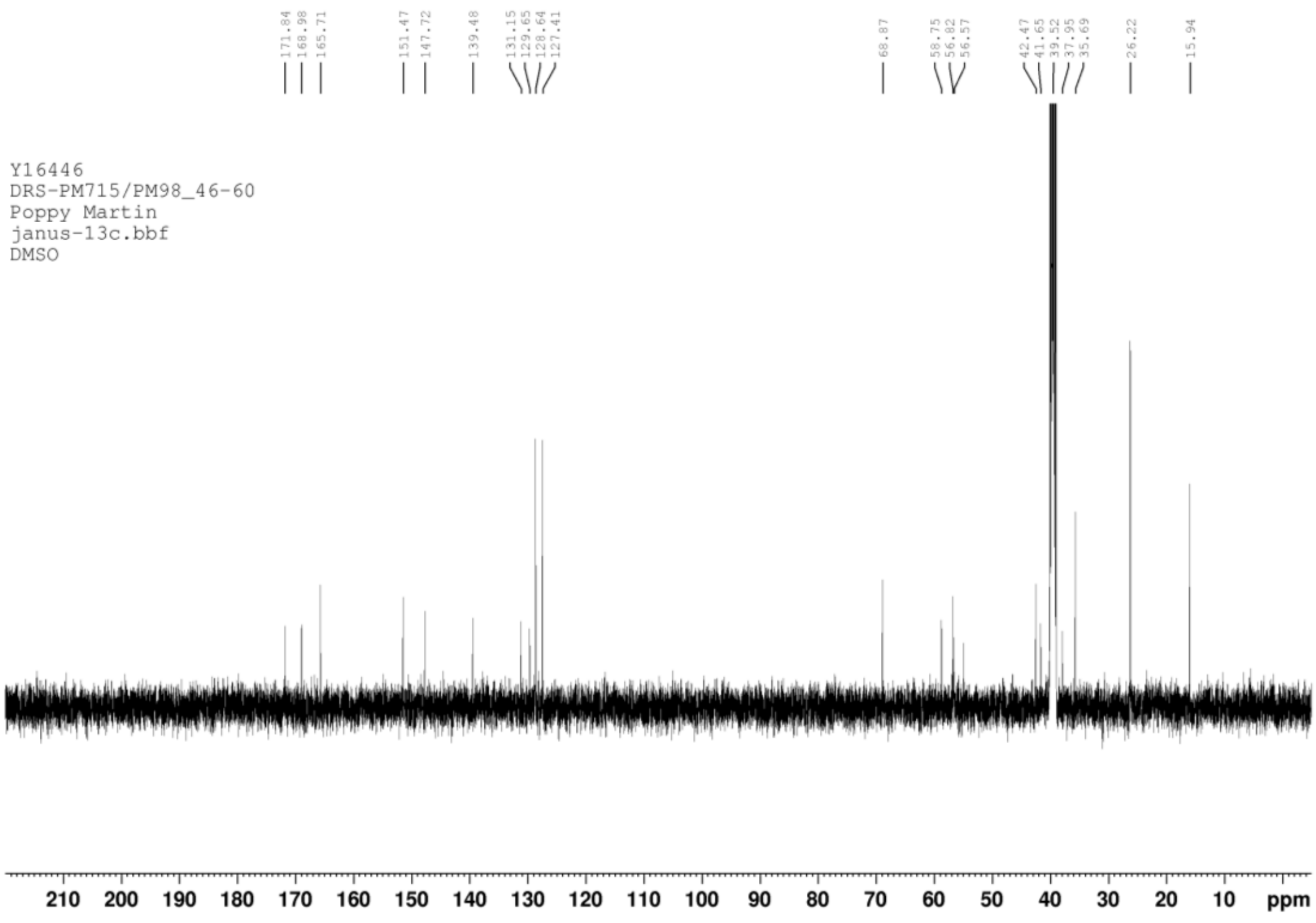

(3b)

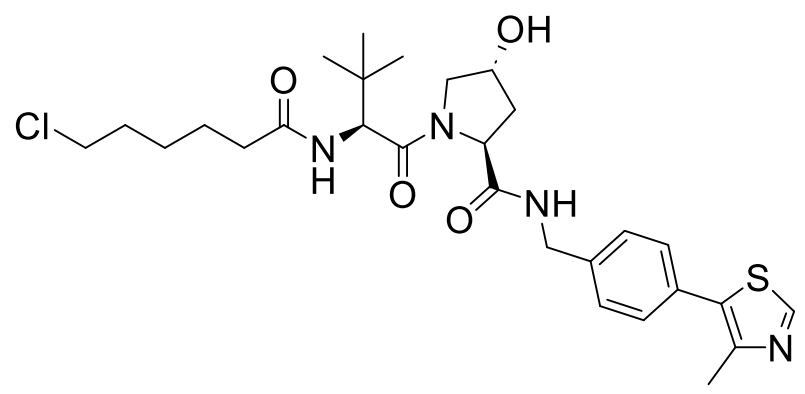
OH
O
Cl
N
H
N
O
O
NH
S
N

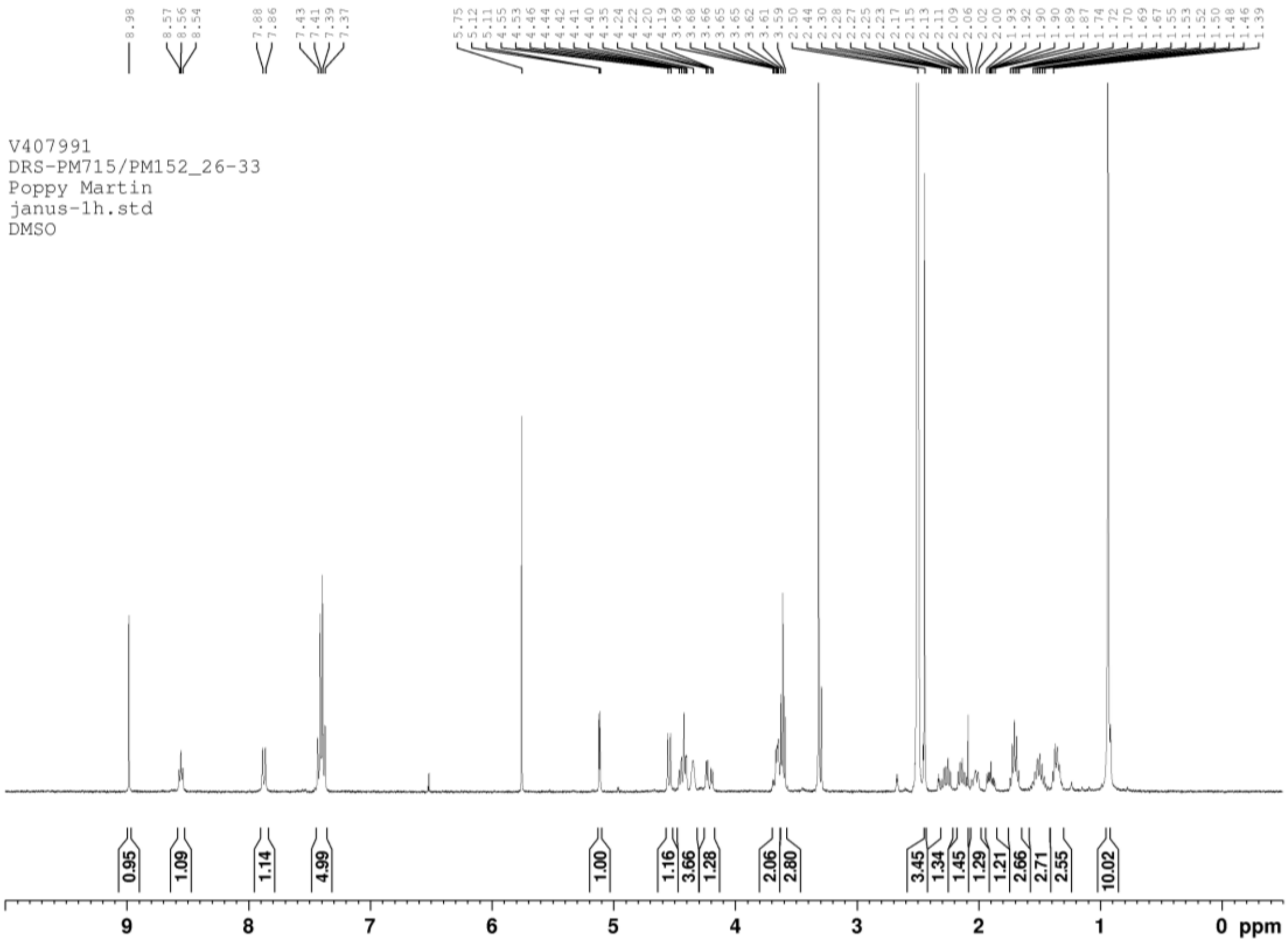
V407991
DRS-PM715/PM152_26-33
Poppy Martin
janus-1h.std
DMSO
ppm

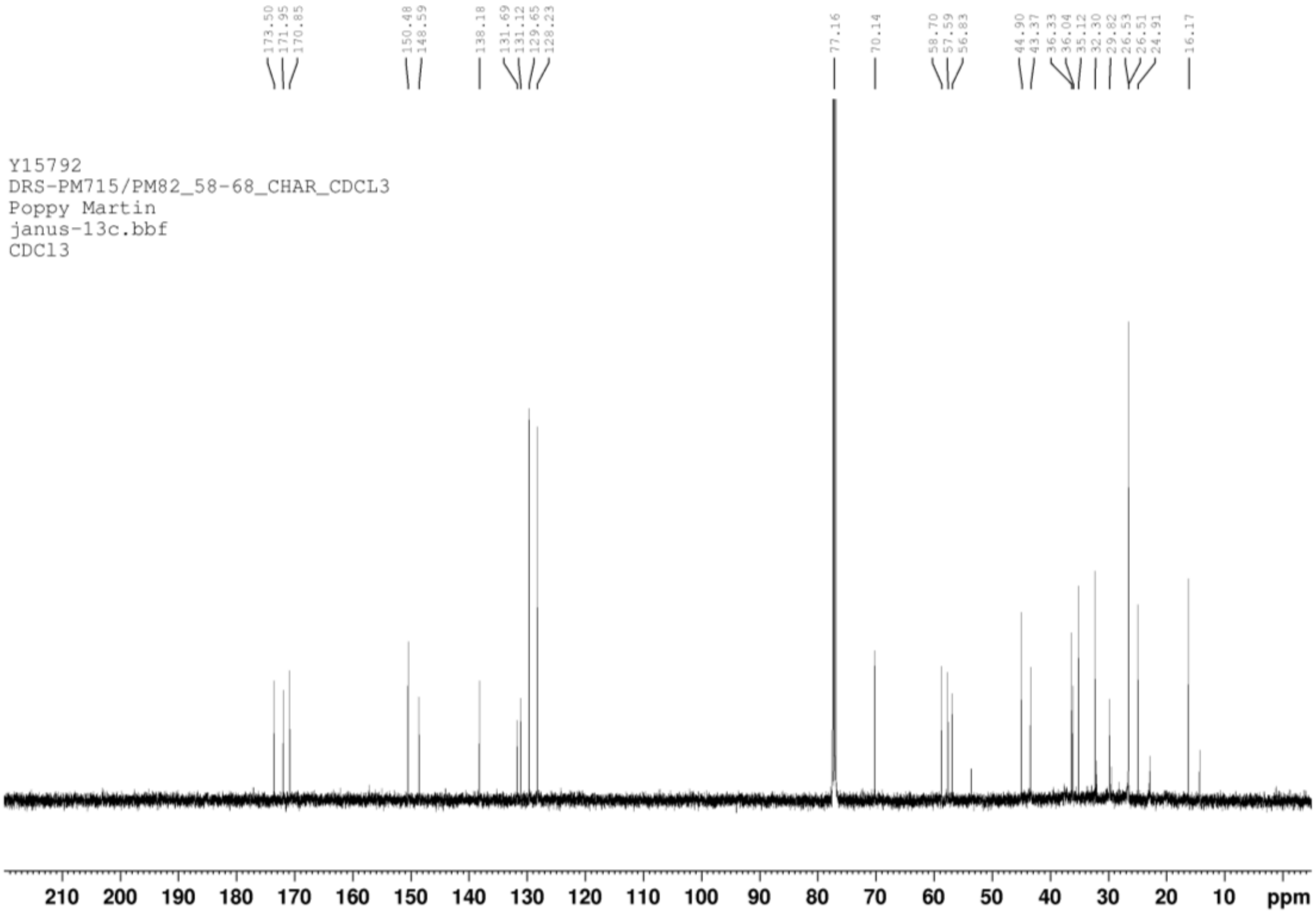
Y15792
DRS-PM715/PM82_58-68_CHAR_CDCL3
Poppy Martin
janus-13c.bbf
CDCl3
ppm

(3c)

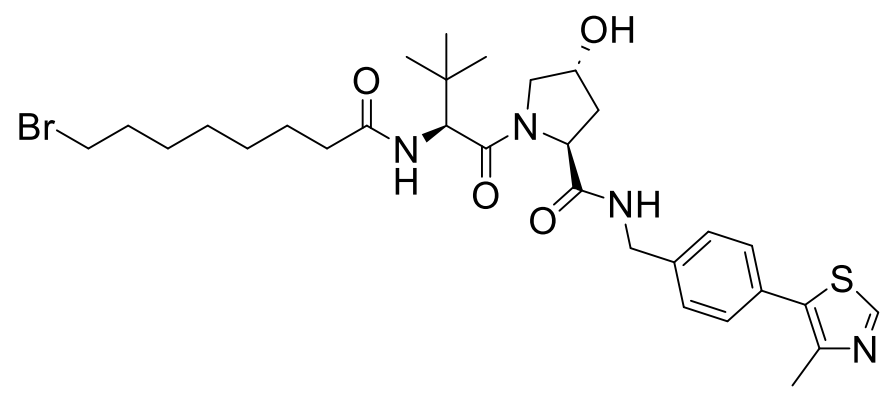
OH
O
Br
N
H
N
O
O
NH
S
N

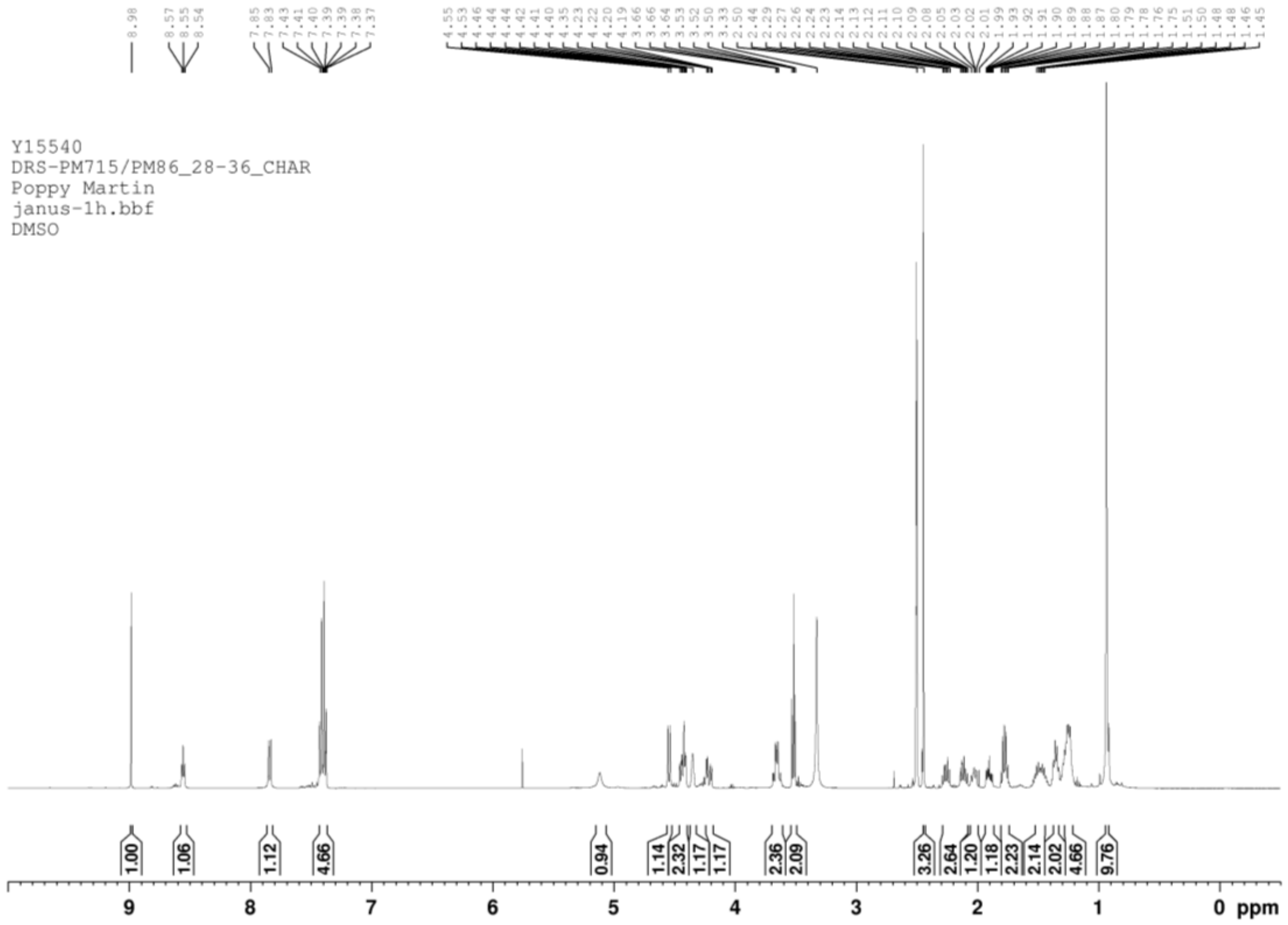
Y15540
DRS-PM715/PM86_28-36_CHAR
Poppy Martin
janus-1h.bbf
DMSO
9
8
7
6
5
4
3
2
1
0 ppm

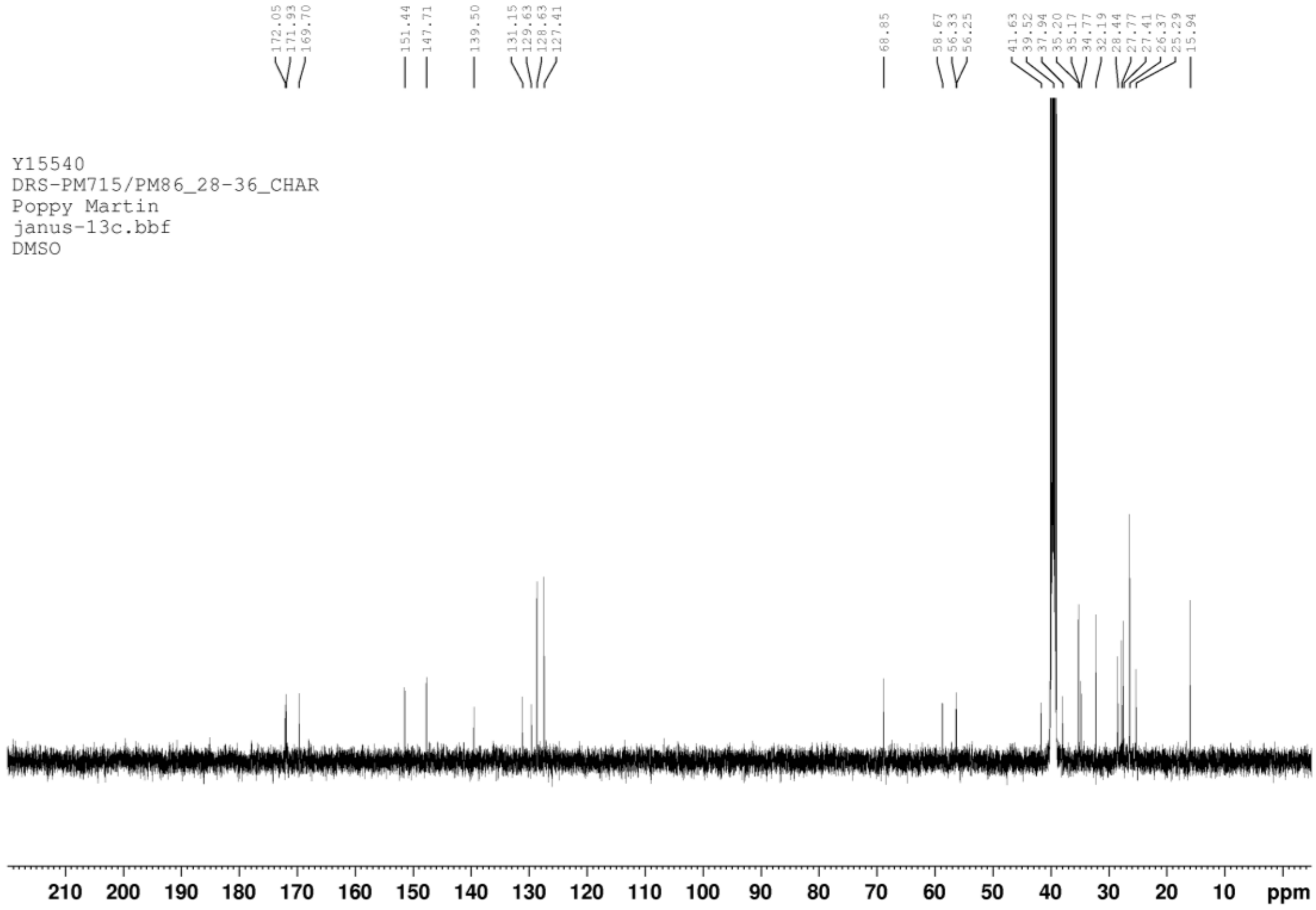
Y15540
DRS-PM715/PM86_28-36_CHAR
Poppy Martin
janus-13c.bbf
DMSO
210
200
190
180
170
160
150
140
130
120
110
100
90
80
70
60
50
40
30
20
10
ppm

(3d)

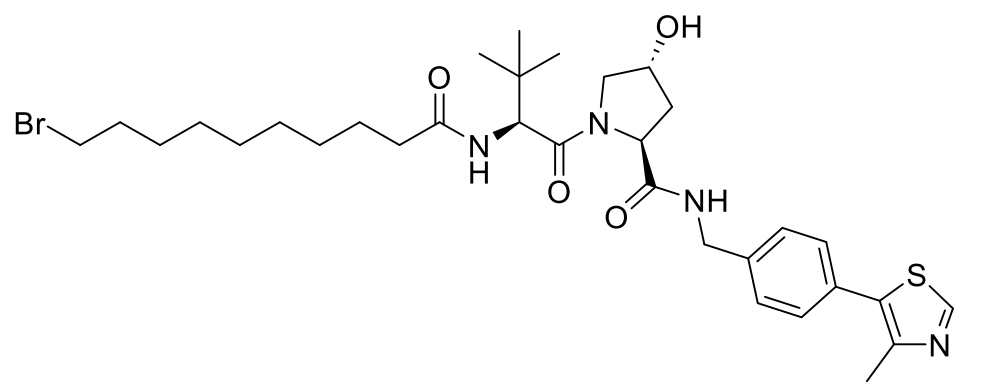

Br
O
N
H
OH
N
O
O
NH
S
N

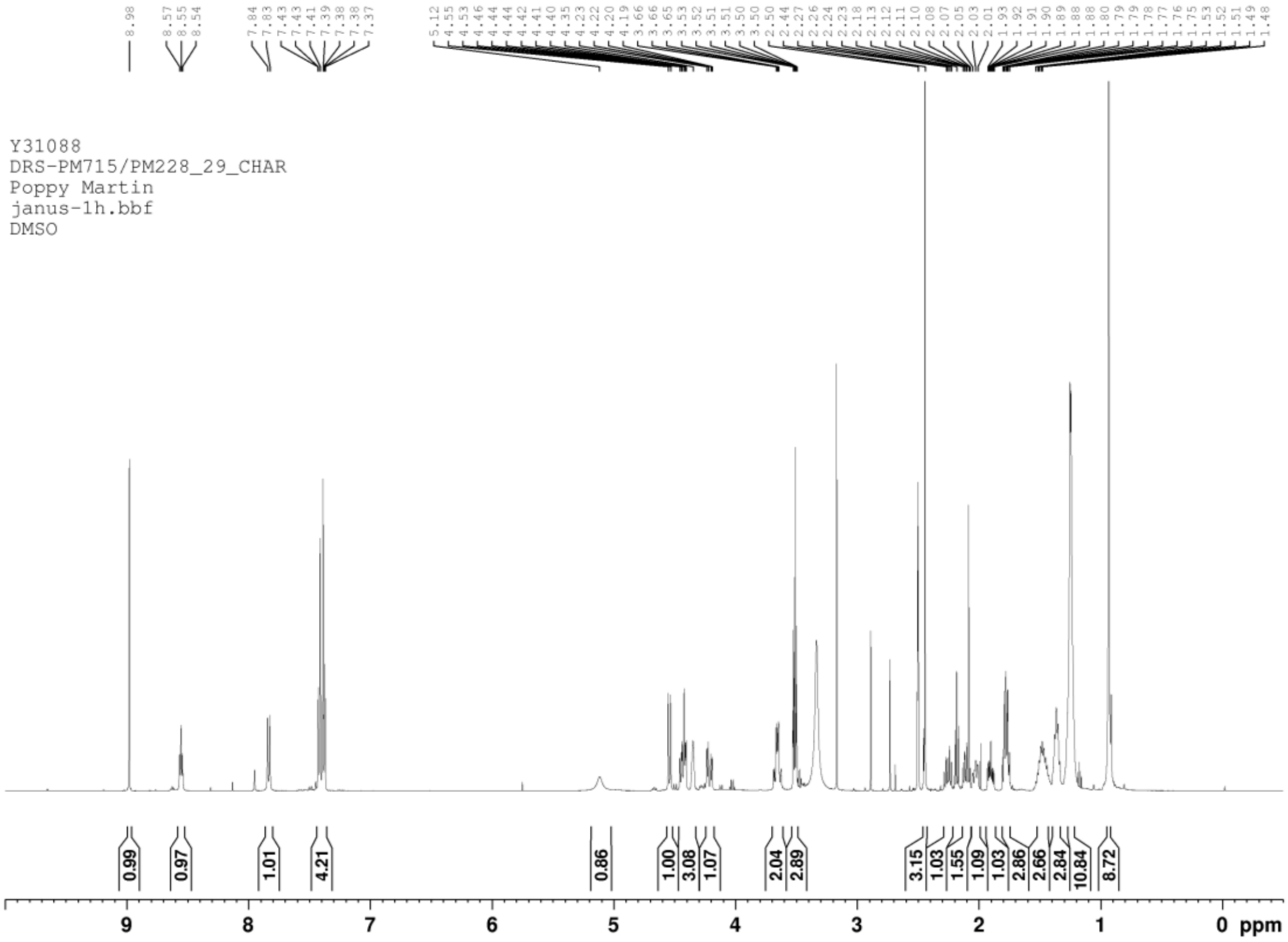

8.98 8.57 8.55 8.54 7.84 7.83 7.43 7.43 7.41 7.39 7.38 7.38 7.37 5.12 4.55 4.53 4.46 4.44 4.44 4.42 4.41 4.40 4.35 4.23 4.22 4.20 4.19 3.66 3.66 3.65 3.53 3.52 3.51 3.51 3.50 3.50 2.50 2.44 2.27 2.26 2.24 2.23 2.18 2.13 2.12 2.11 2.10 2.08 2.07 2.05 2.03 2.01 1.93 1.92 1.91 1.90 1.89 1.88 1.88 1.80 1.79 1.79 1.78 1.77 1.76 1.75 1.53 1.52 1.51 1.49 1.48
Y31088
DRS-PM715/PM228_29_CHAR
Poppy Martin
janus-1h.bbf
DMSO
0.99 0.97 1.01 4.21 0.86 1.00 3.08 1.07 2.04 2.89 3.15 1.03 1.55 1.09 1.03 2.86 2.66 2.84 10.84 8.72
9 8 7 6 5 4 3 2 1 0 ppm

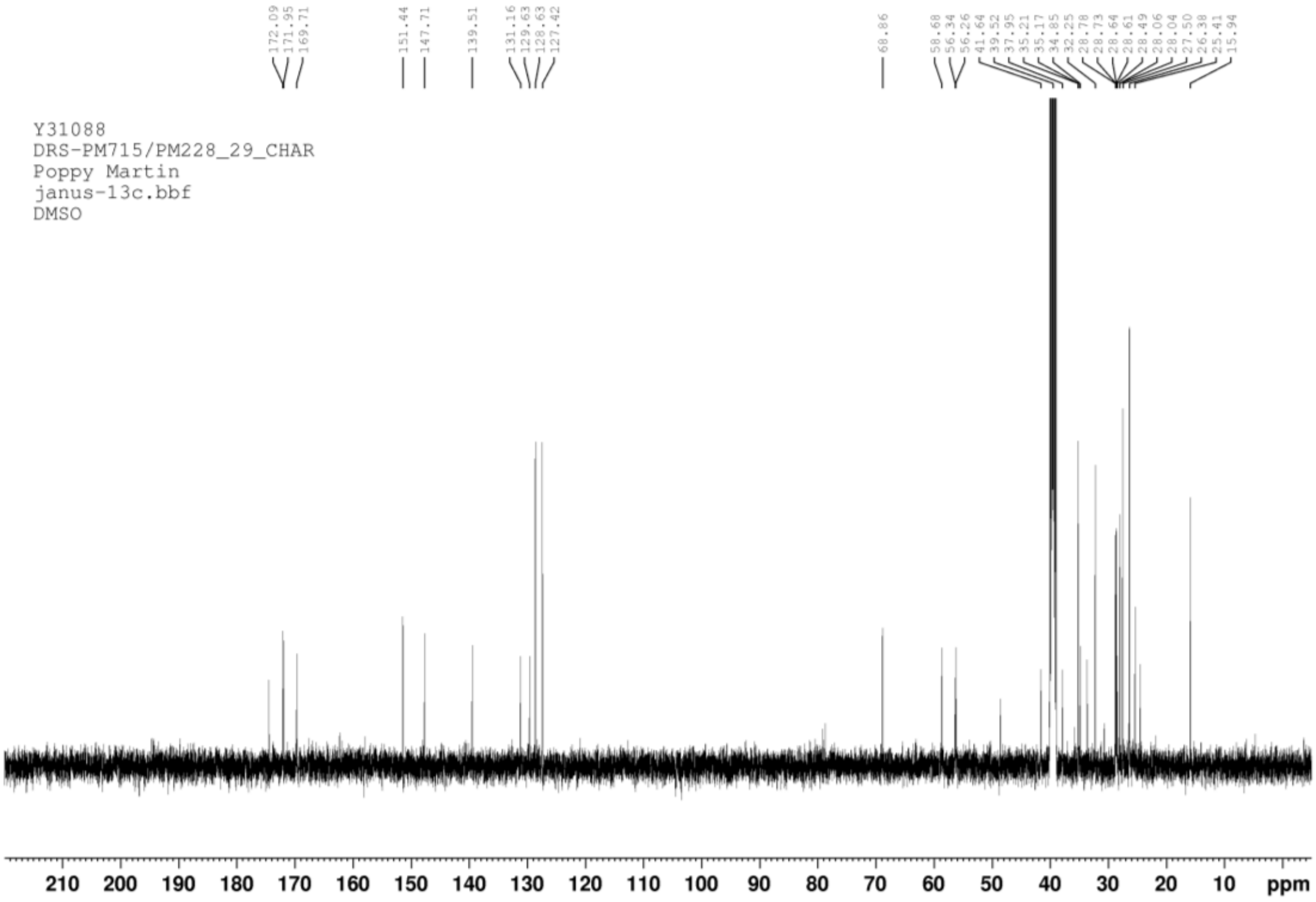

172.09 171.95 169.71 151.44 147.71 139.51 131.16 129.63 128.63 127.42 68.86 58.68 56.34 56.26 41.64 39.52 37.95 35.21 35.17 34.85 32.25 28.78 28.73 28.64 28.61 28.49 28.06 28.04 27.50 26.38 25.41 15.94
Y31088
DRS-PM715/PM228_29_CHAR
Poppy Martin
janus-13c.bbf
DMSO
210 200 190 180 170 160 150 140 130 120 110 100 90 80 70 60 50 40 30 20 10 ppm

(3e)

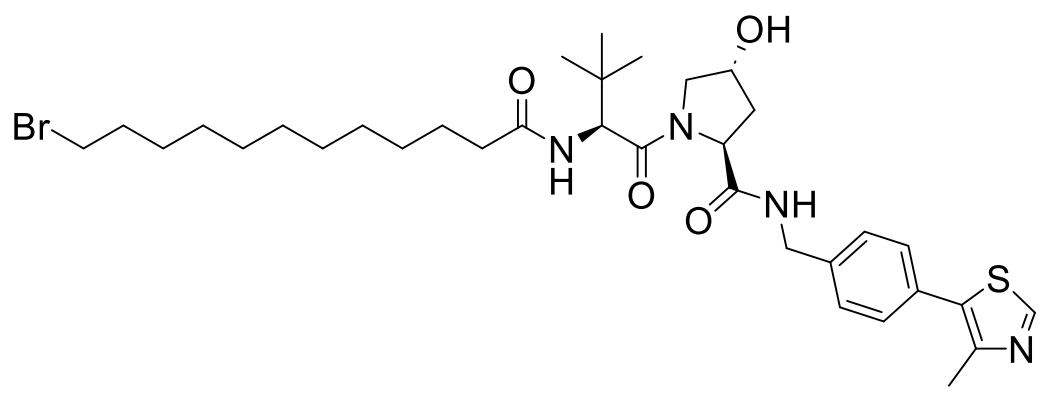

Br
O
N
H
O
N
OH
O
NH
S
N

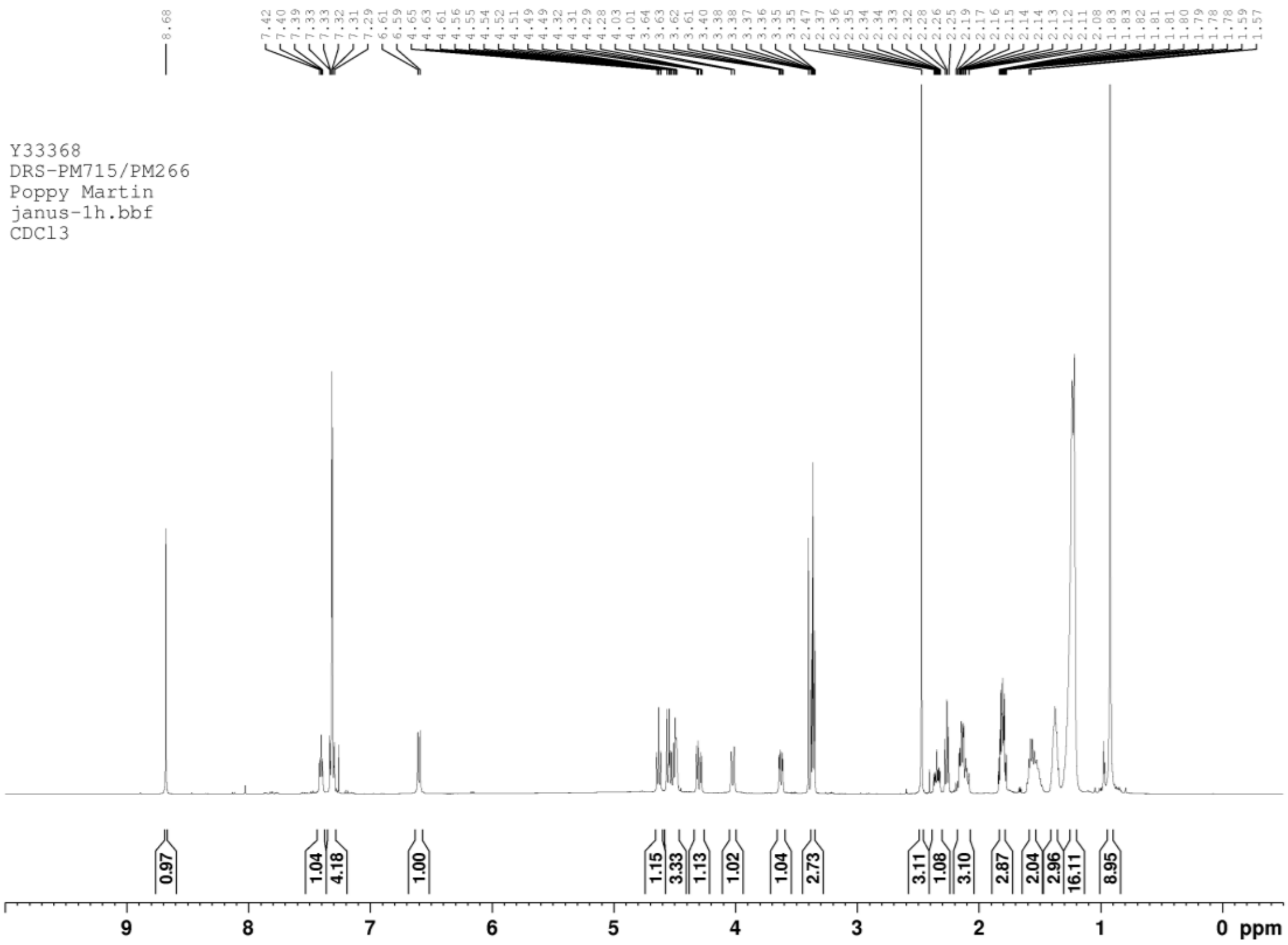

Y33368
DRS-PM715/PM266
Poppy Martin
janus-1h.bbf
CDCl3
ppm

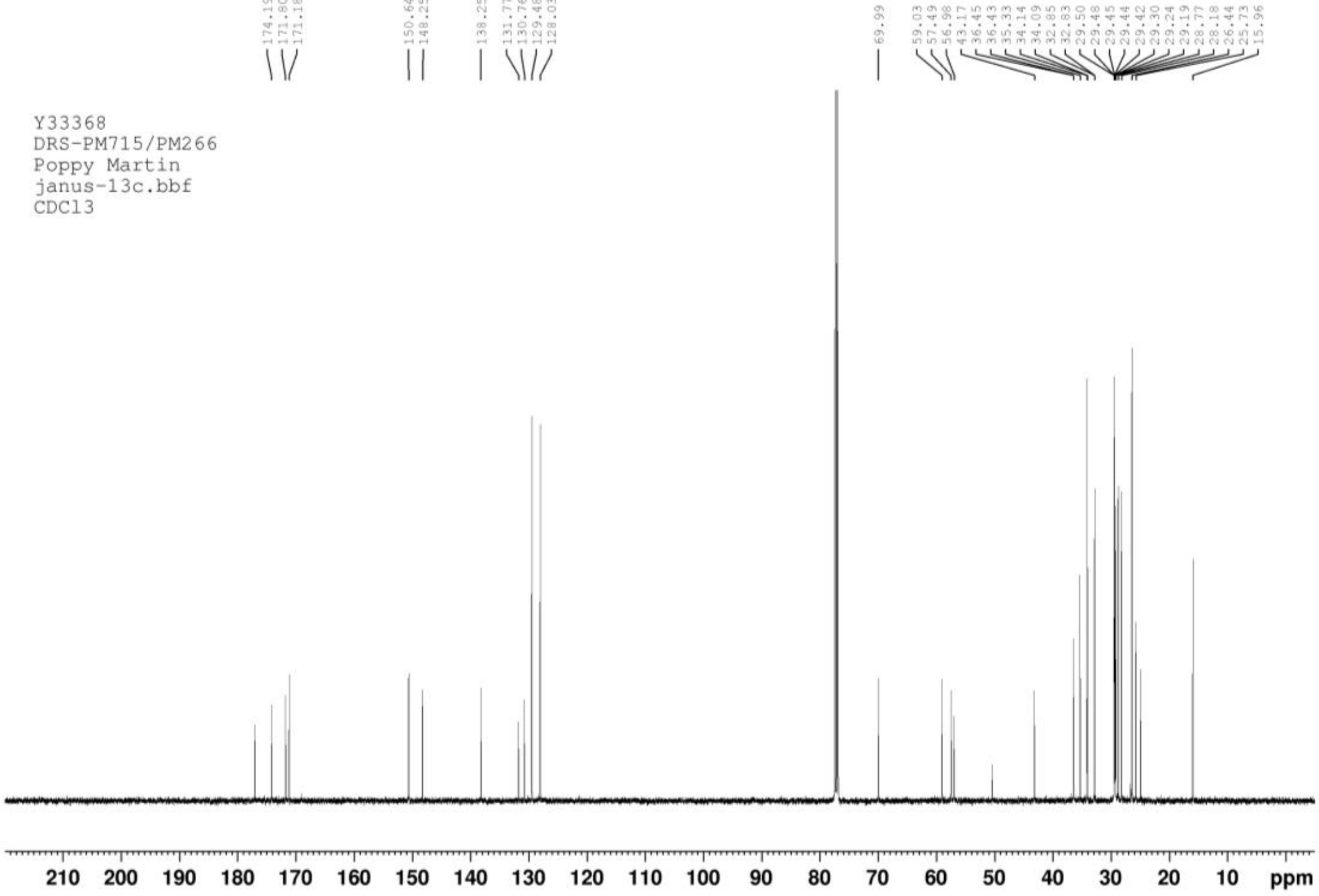

Y33368
DRS-PM715/PM266
Poppy Martin
janus-13c.bbf
CDCl3
ppm

(3f)

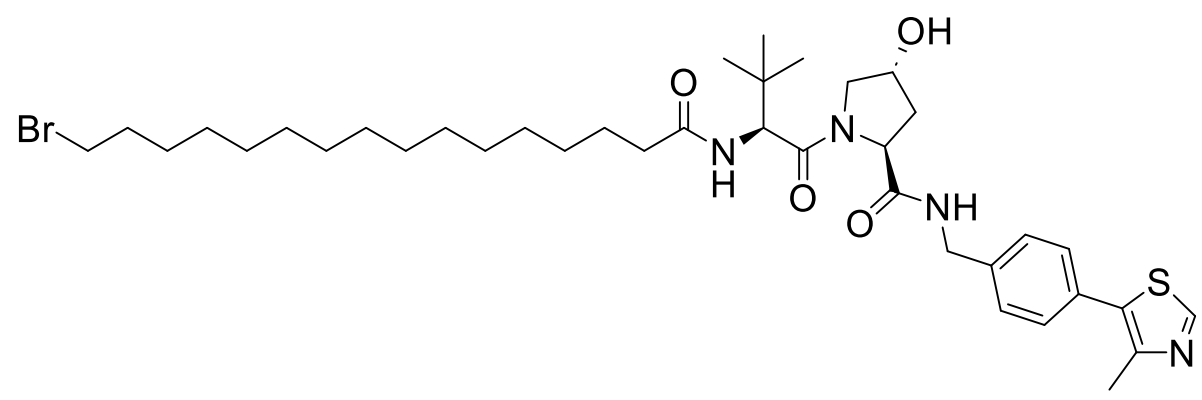
Br
O
N
H
O
N
OH
O
NH
S
N

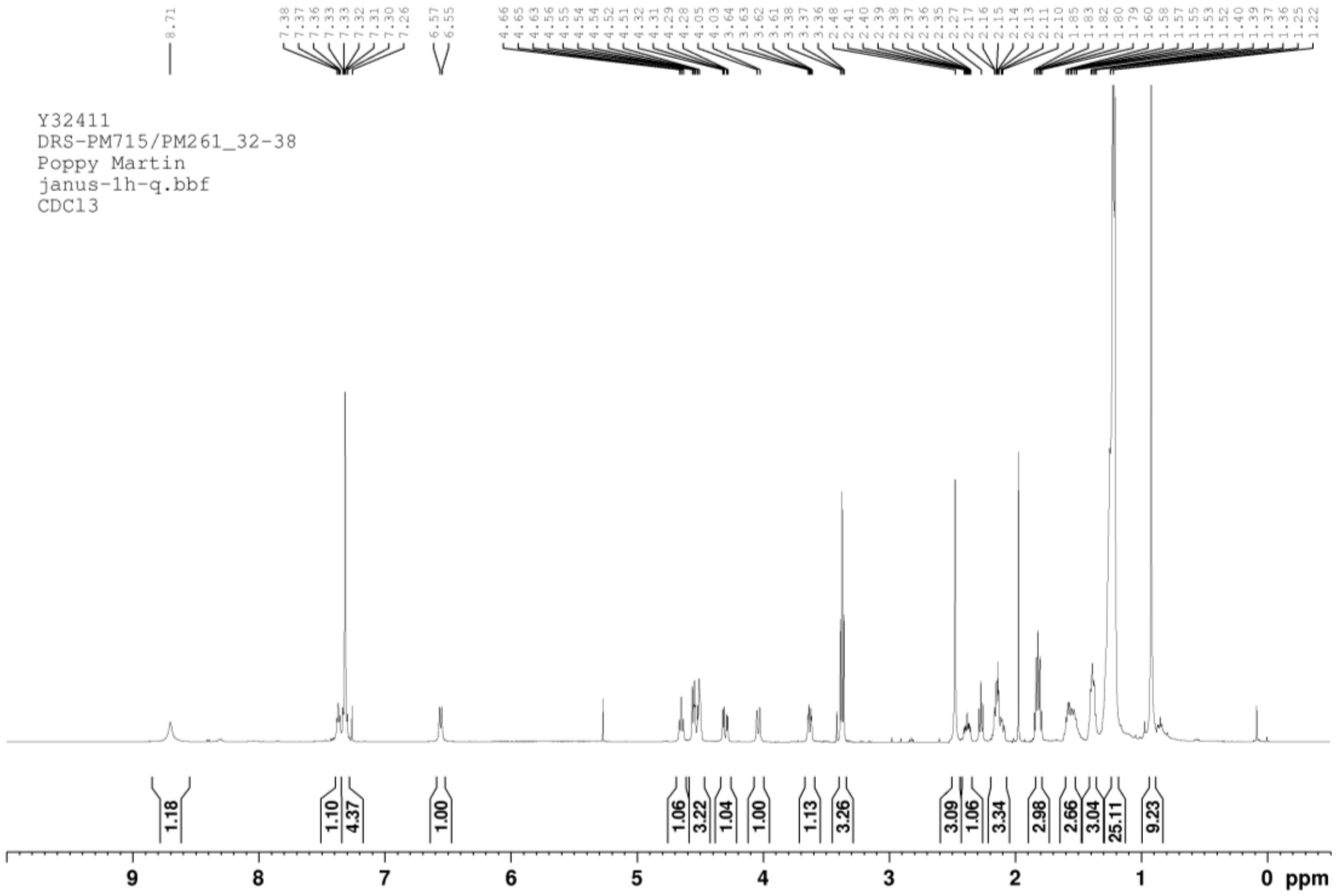
Y32411
DRS-PM715/PM261_32-38
Poppy Martin
janus-1h-q.bbf
CDCl3
ppm

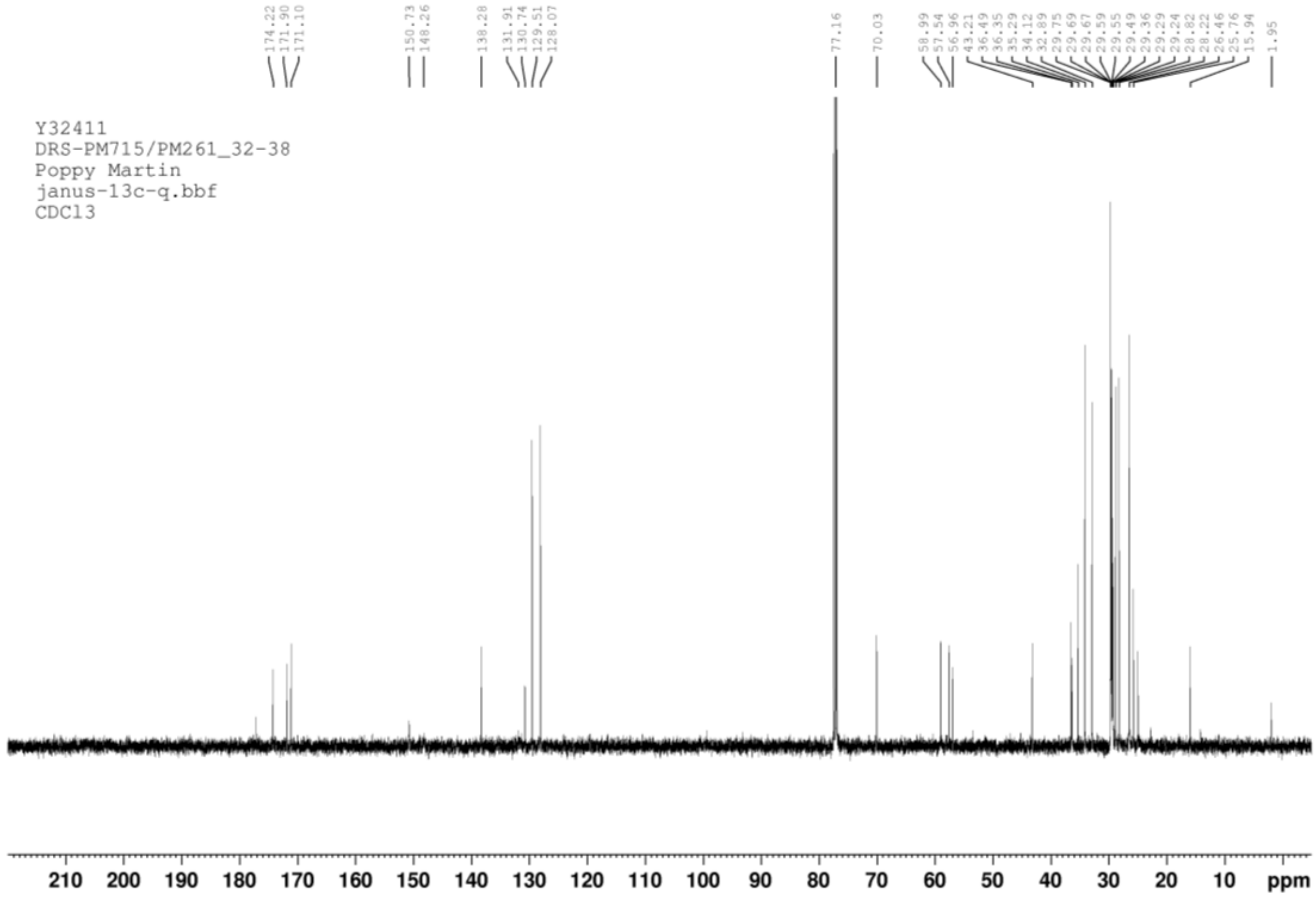
Y32411
DRS-PM715/PM261_32-38
Poppy Martin
janus-13c-q.bbf
CDCl3
ppm

(3g)

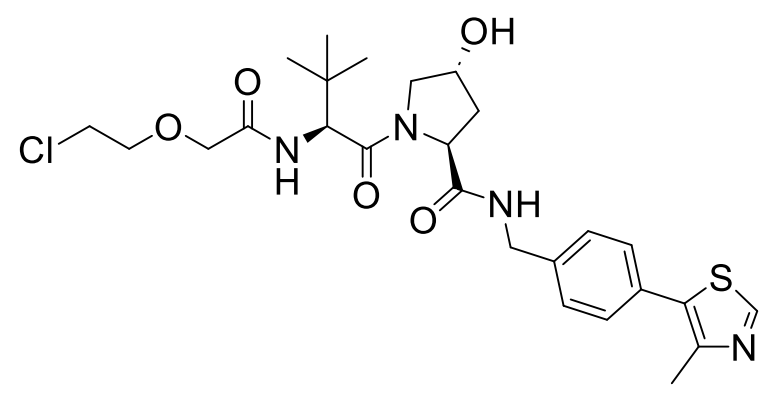
OH
Cl
O
O
N
H
N
O
O
NH
S
N

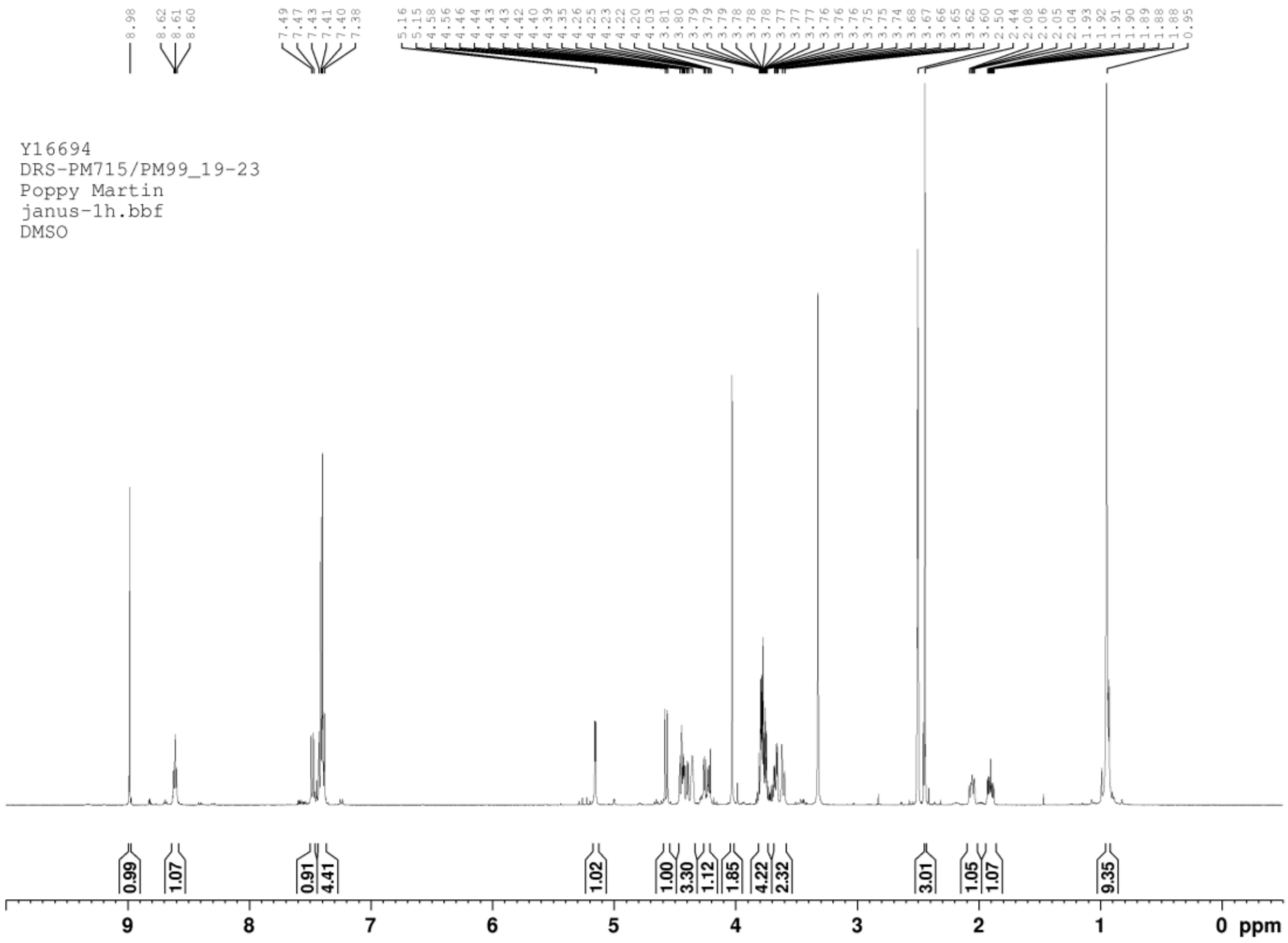
Y16694
DRS-PM715/PM99_19-23
Poppy Martin
janus-1h.bbf
DMSO
0.99
1.07
0.91
4.41
1.02
1.00
3.30
1.12
1.85
4.22
2.32
3.01
1.05
1.07
9.35
ppm

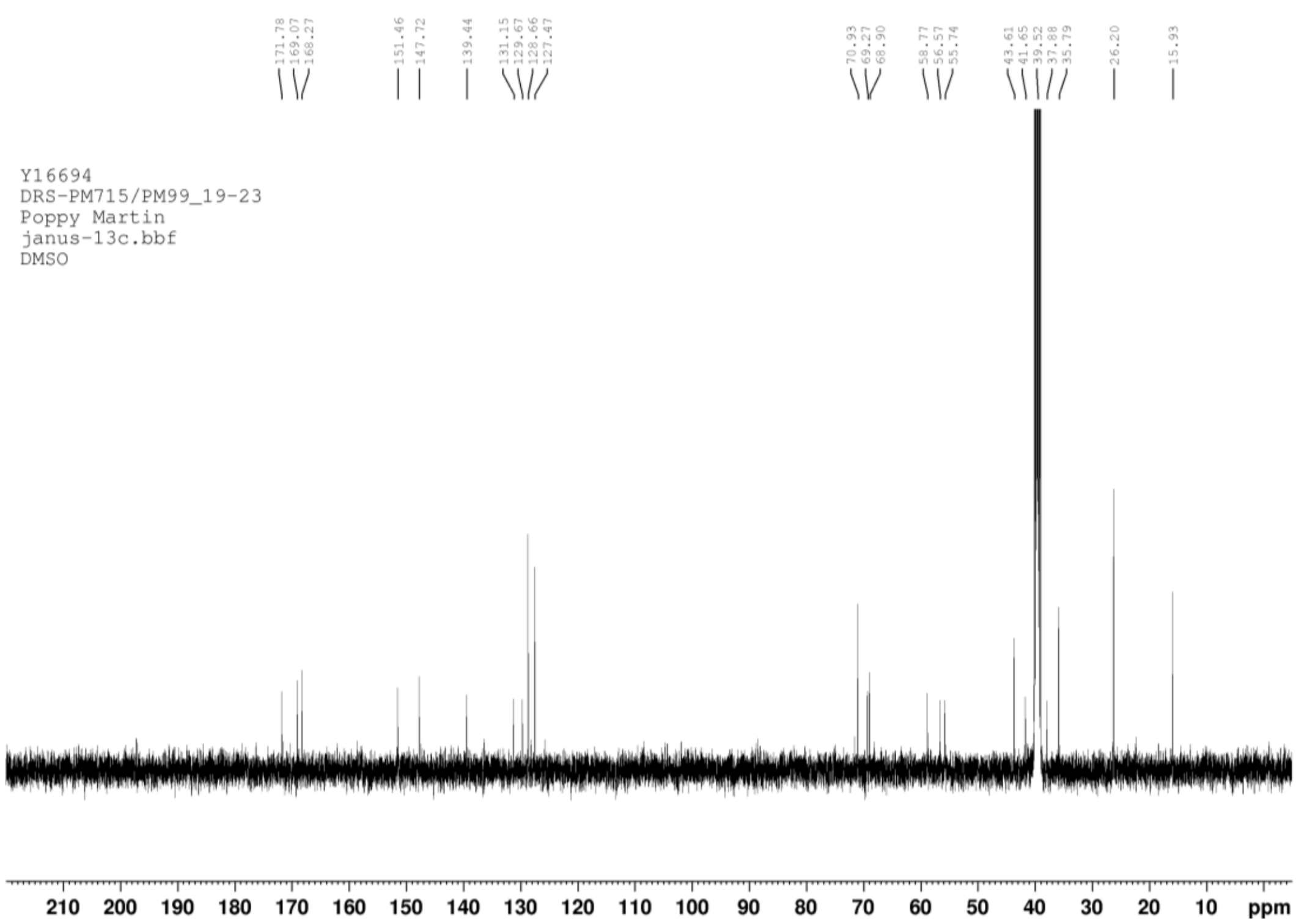
171.78
169.07
168.27
151.46
147.72
139.44
131.15
129.67
128.66
127.47
70.93
69.27
68.90
58.77
56.57
55.74
43.61
41.65
39.52
37.88
35.79
26.20
15.93
Y16694
DRS-PM715/PM99_19-23
Poppy Martin
janus-13c.bbf
DMSO
ppm

(3h)

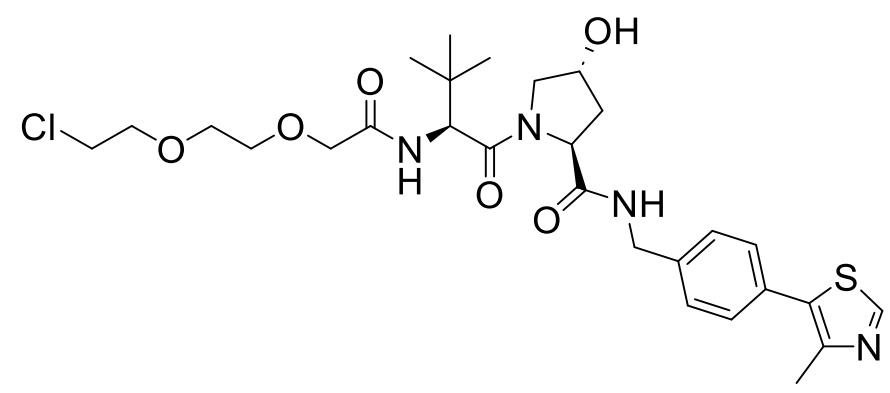

Cl
O
O
O
N
H
O
N
OH
O
NH
S
N

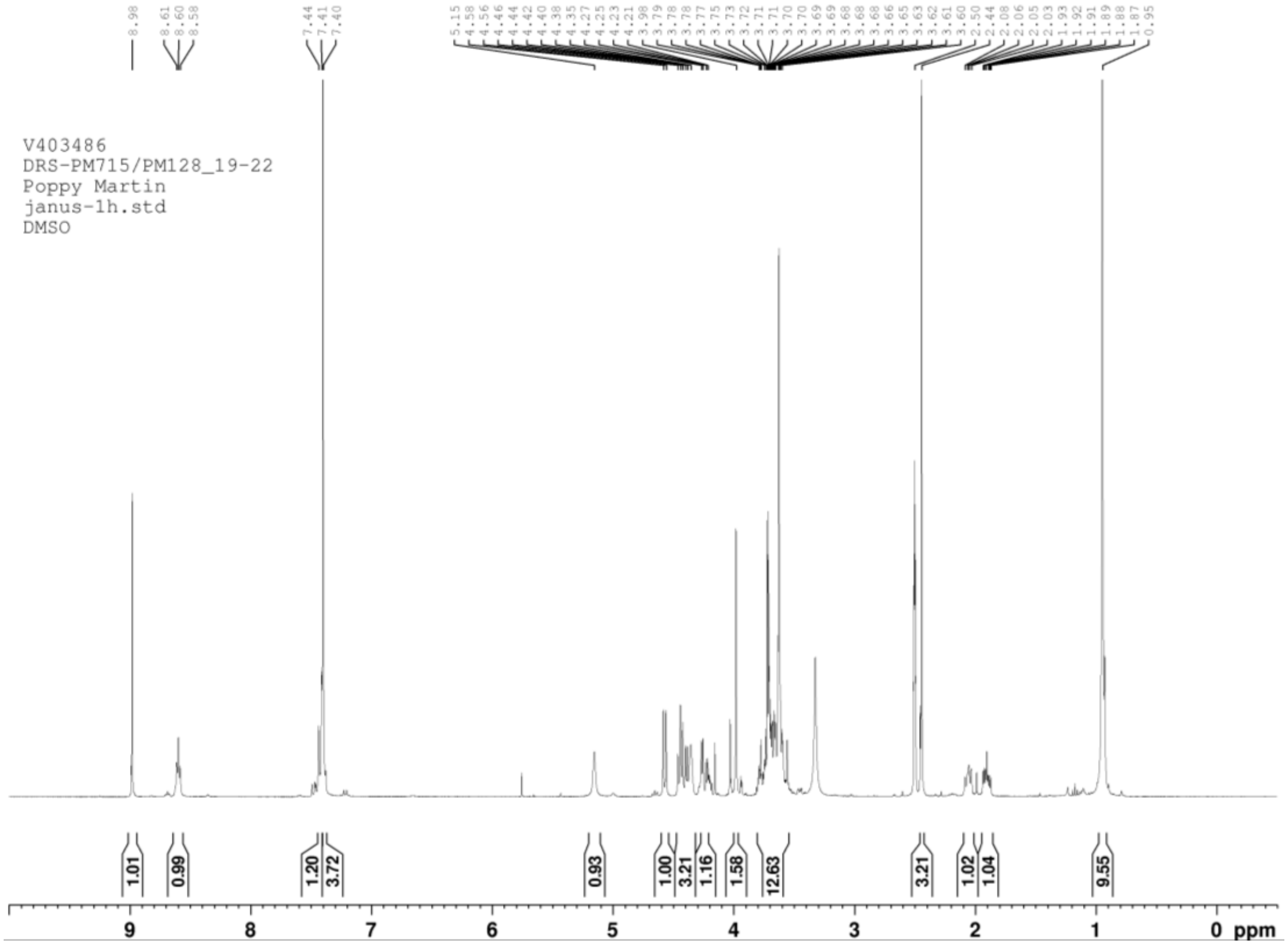

V403486
DRS-PM715/PM128_19-22
Poppy Martin
janus-1h.std
DMSO
1.01
0.99
1.20
3.72
0.93
1.00
3.21
1.16
1.58
12.63
3.21
1.02
1.04
9.55
9
8
7
6
5
4
3
2
1
0 ppm

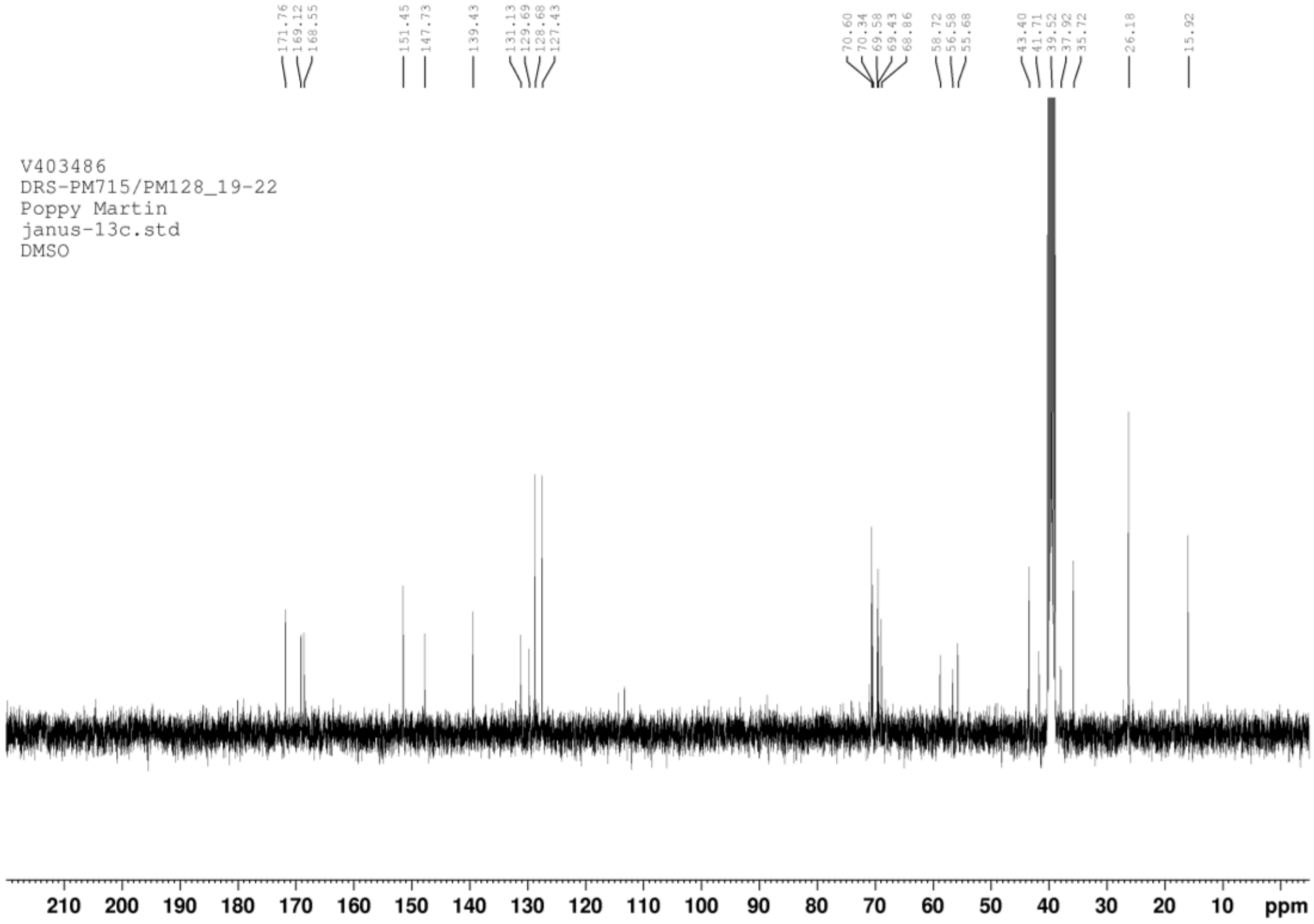

171.76
169.12
168.55
151.45
147.73
139.43
131.13
129.69
128.68
127.43
70.60
70.34
69.58
69.43
68.86
58.72
56.58
55.68
43.40
41.71
39.52
37.92
35.72
26.18
15.92
V403486
DRS-PM715/PM128_19-22
Poppy Martin
janus-13c.std
DMSO
210
200
190
180
170
160
150
140
130
120
110
100
90
80
70
60
50
40
30
20
10
ppm

(3i)

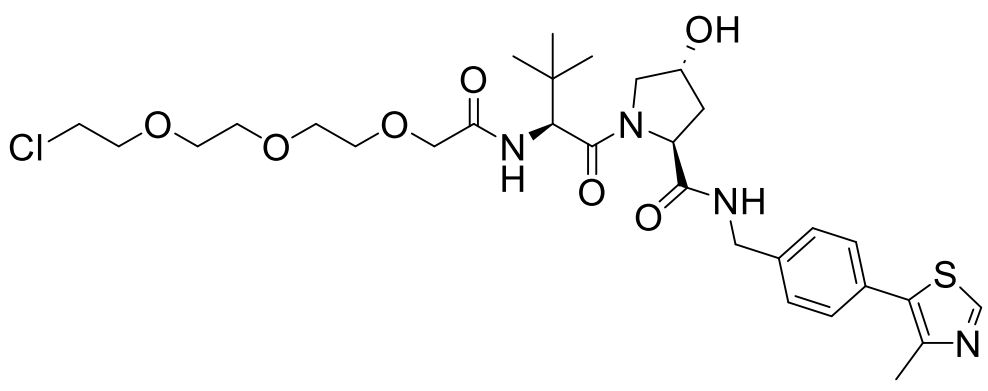
Cl
O
O
O
O
H
N
O
N
OH
O
NH
S
N

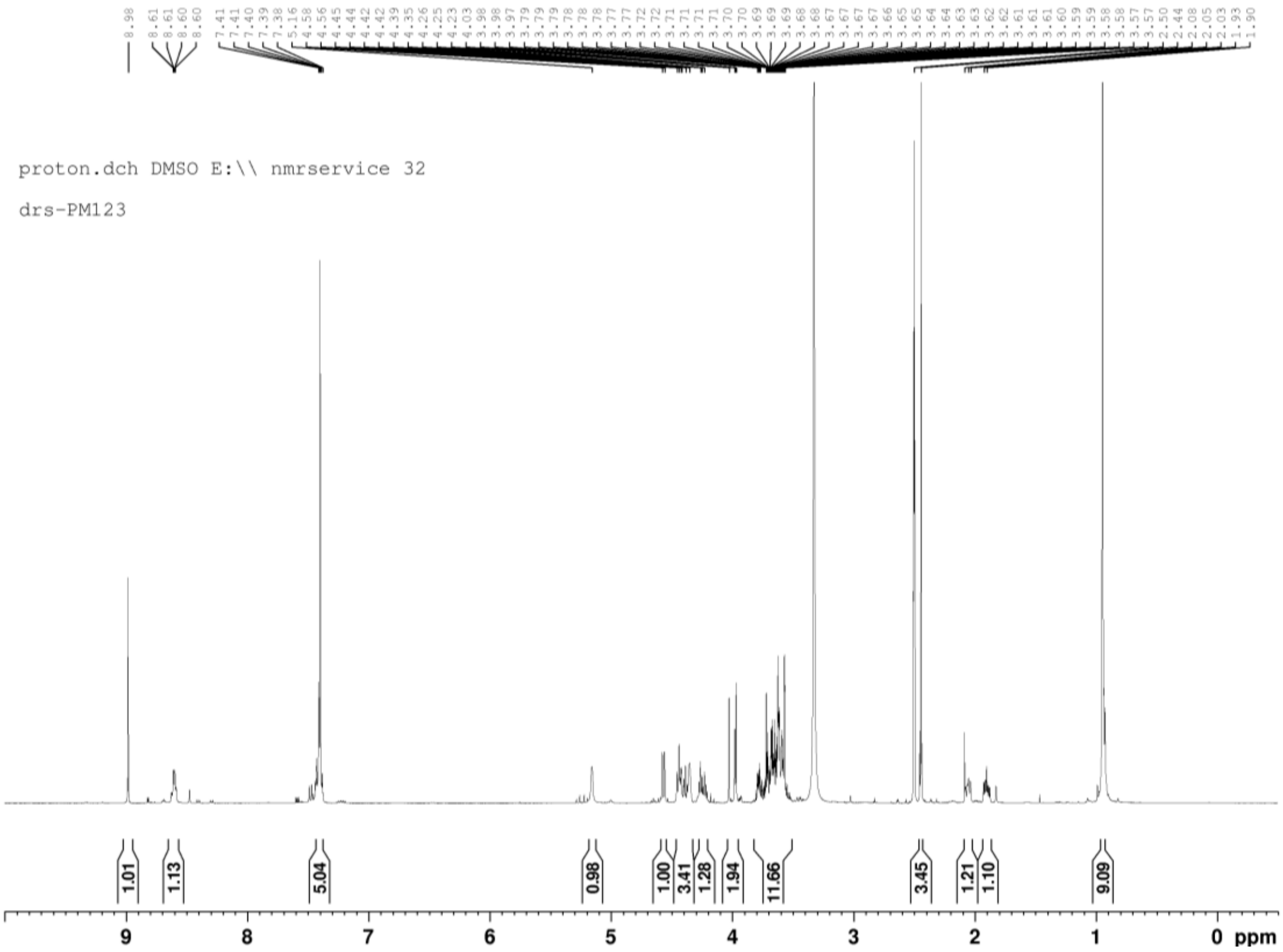
proton.dch DMSO E:\\ nmrservice 32
drs-PM123
1.01
1.13
5.04
0.98
1.00
3.41
1.28
1.94
11.66
3.45
1.21
1.10
9.09
9
8
7
6
5
4
3
2
1
0 ppm

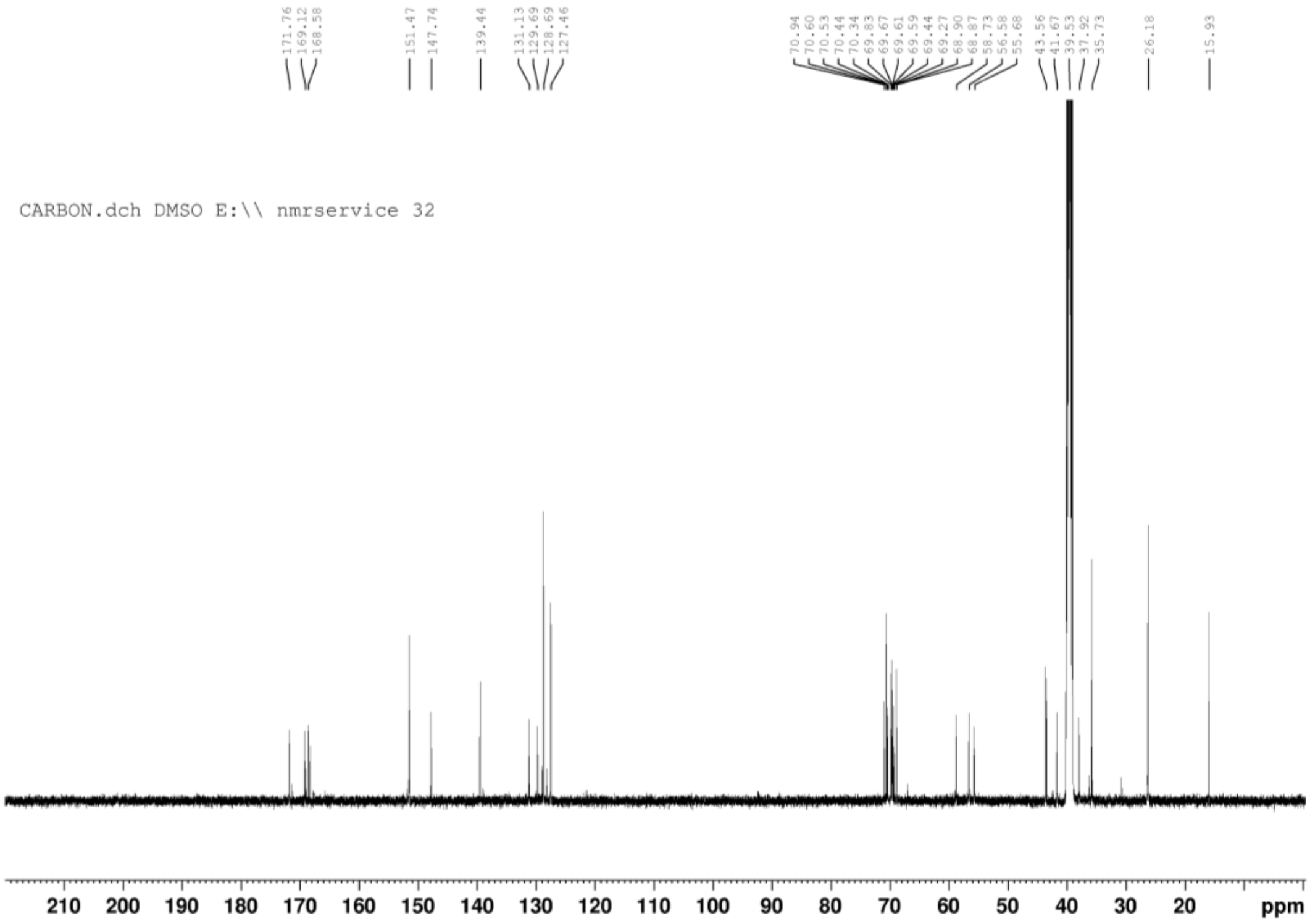
CARBON.dch DMSO E:\\ nmrservice 32
210
200
190
180
170
160
150
140
130
120
110
100
90
80
70
60
50
40
30
20
ppm

(4)

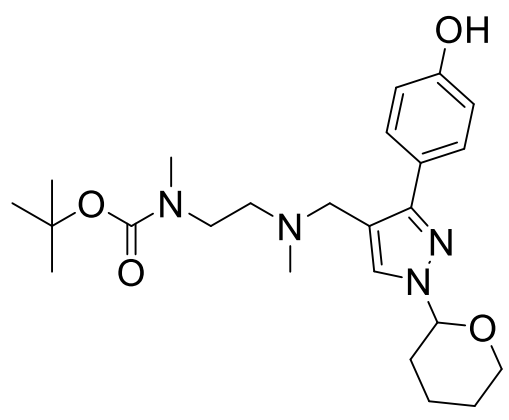
OH
O
N
N
N
O
N
O

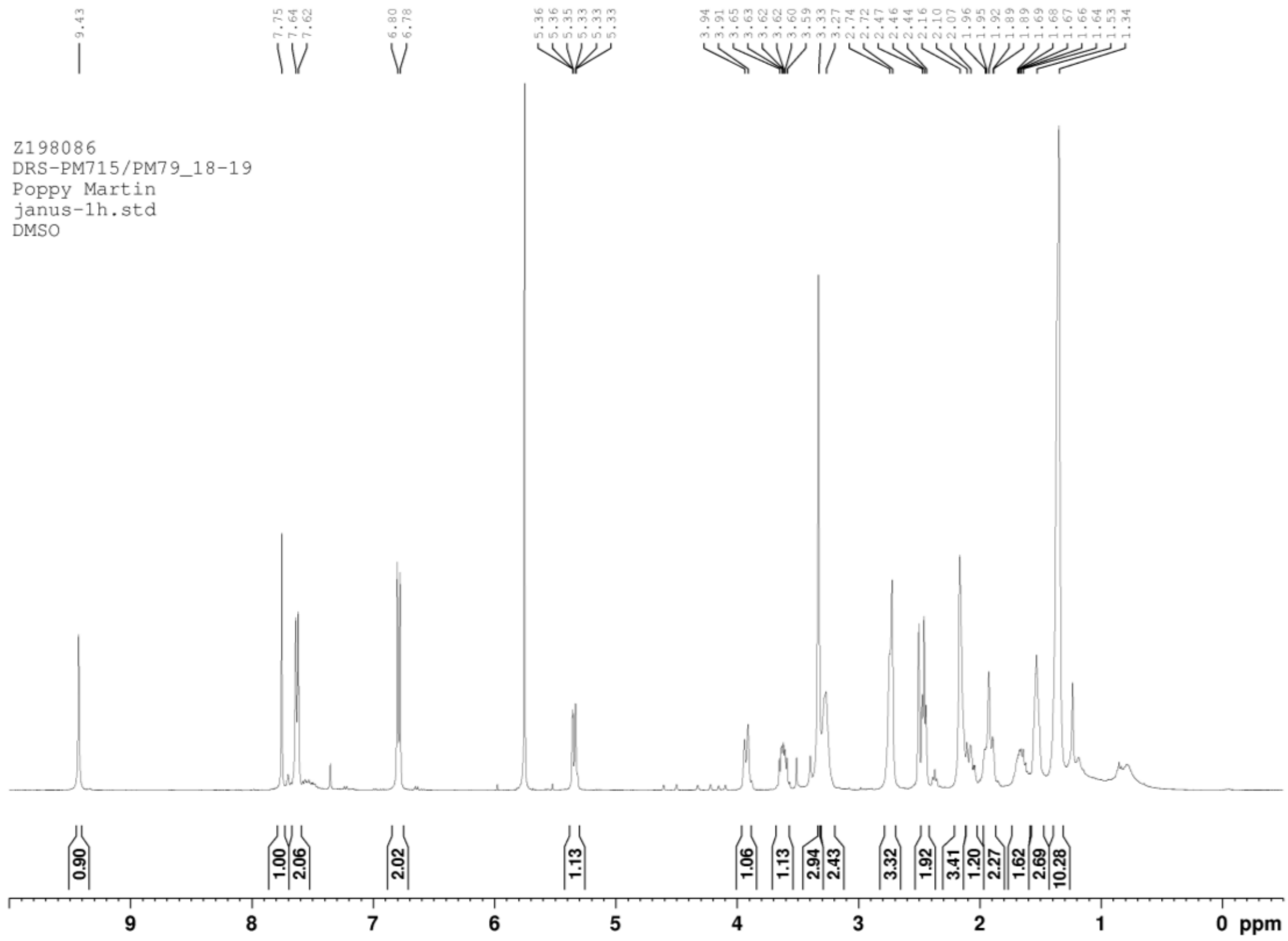
9.43
7.75
7.64
7.62
6.80
6.78
5.36
5.36
5.35
5.35
5.33
5.33
5.33
3.94
3.91
3.65
3.63
3.62
3.62
3.60
3.59
3.33
3.27
2.74
2.72
2.47
2.46
2.44
2.16
2.10
2.07
1.96
1.95
1.92
1.89
1.89
1.69
1.68
1.67
1.66
1.64
1.53
1.34
Z198086
DRS-PM715/PM79_18-19
Poppy Martin
janus-1h.std
DMSO
0.90
1.00
2.06
2.02
1.13
1.06
1.13
2.94
2.43
3.32
1.92
3.41
1.20
2.27
1.62
2.69
10.28
9
8
7
6
5
4
3
2
1
0 ppm

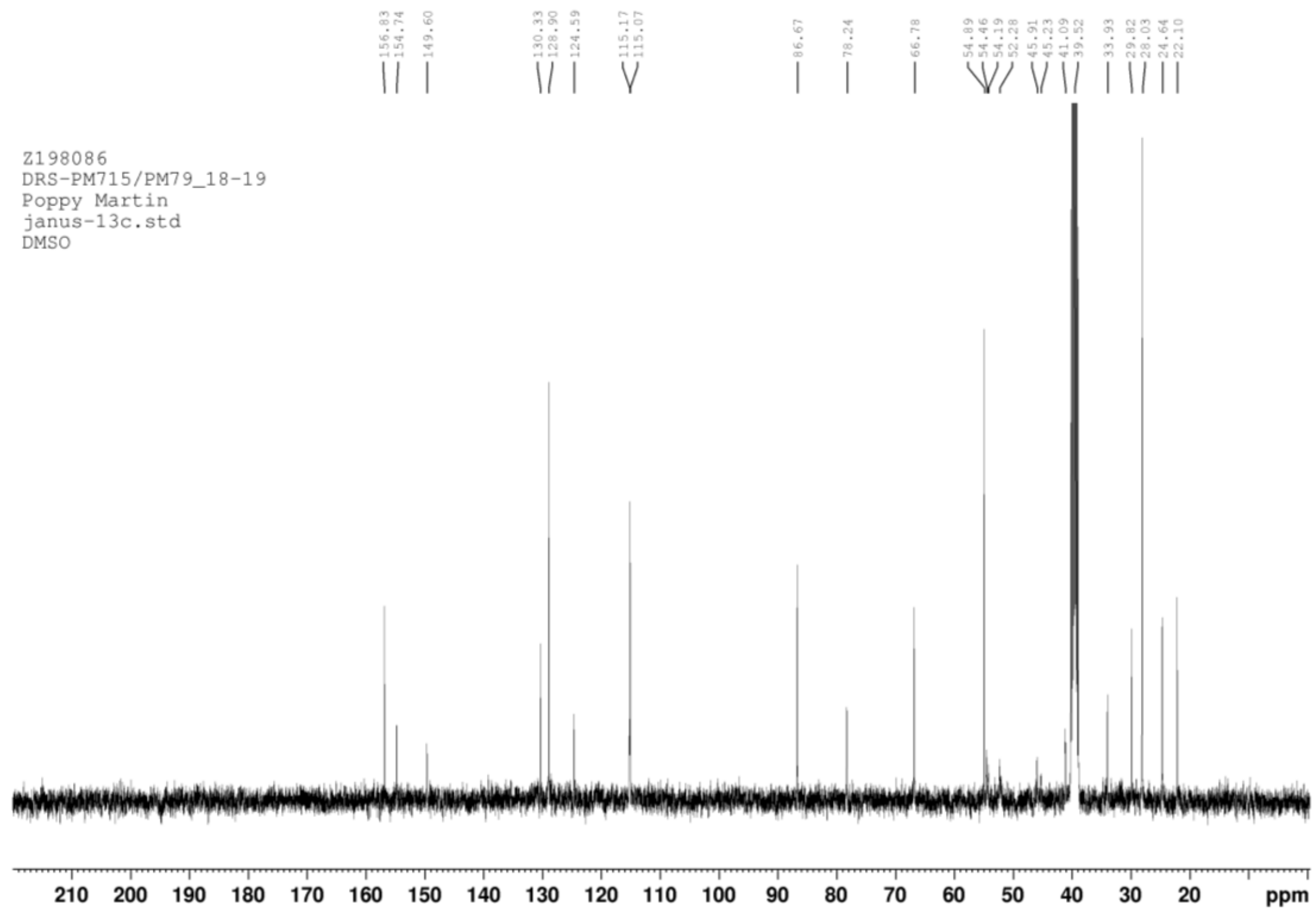
156.83
154.74
149.60
130.33
128.90
124.59
115.17
115.07
86.67
78.24
66.78
54.89
54.46
54.19
52.28
45.91
45.23
41.09
39.52
33.93
29.82
28.03
24.64
22.10
Z198086
DRS-PM715/PM79_18-19
Poppy Martin
janus-13c.std
DMSO
210
200
190
180
170
160
150
140
130
120
110
100
90
80
70
60
50
40
30
20
ppm

(deprotected-4)

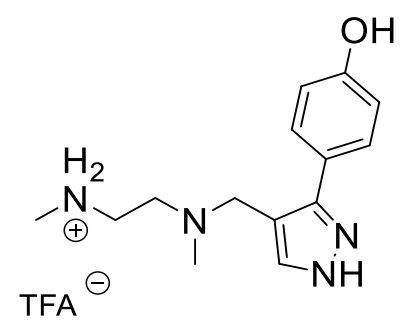

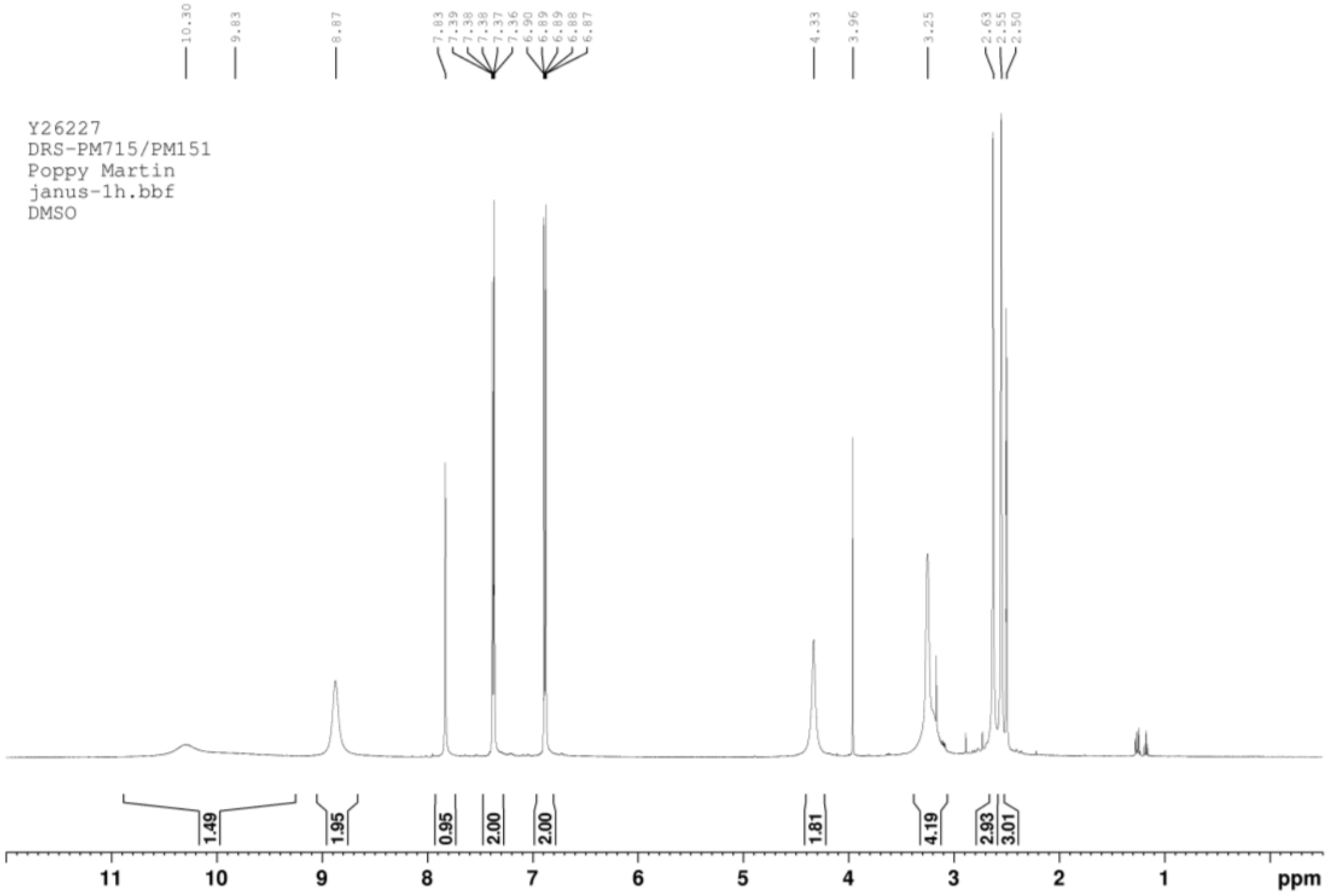

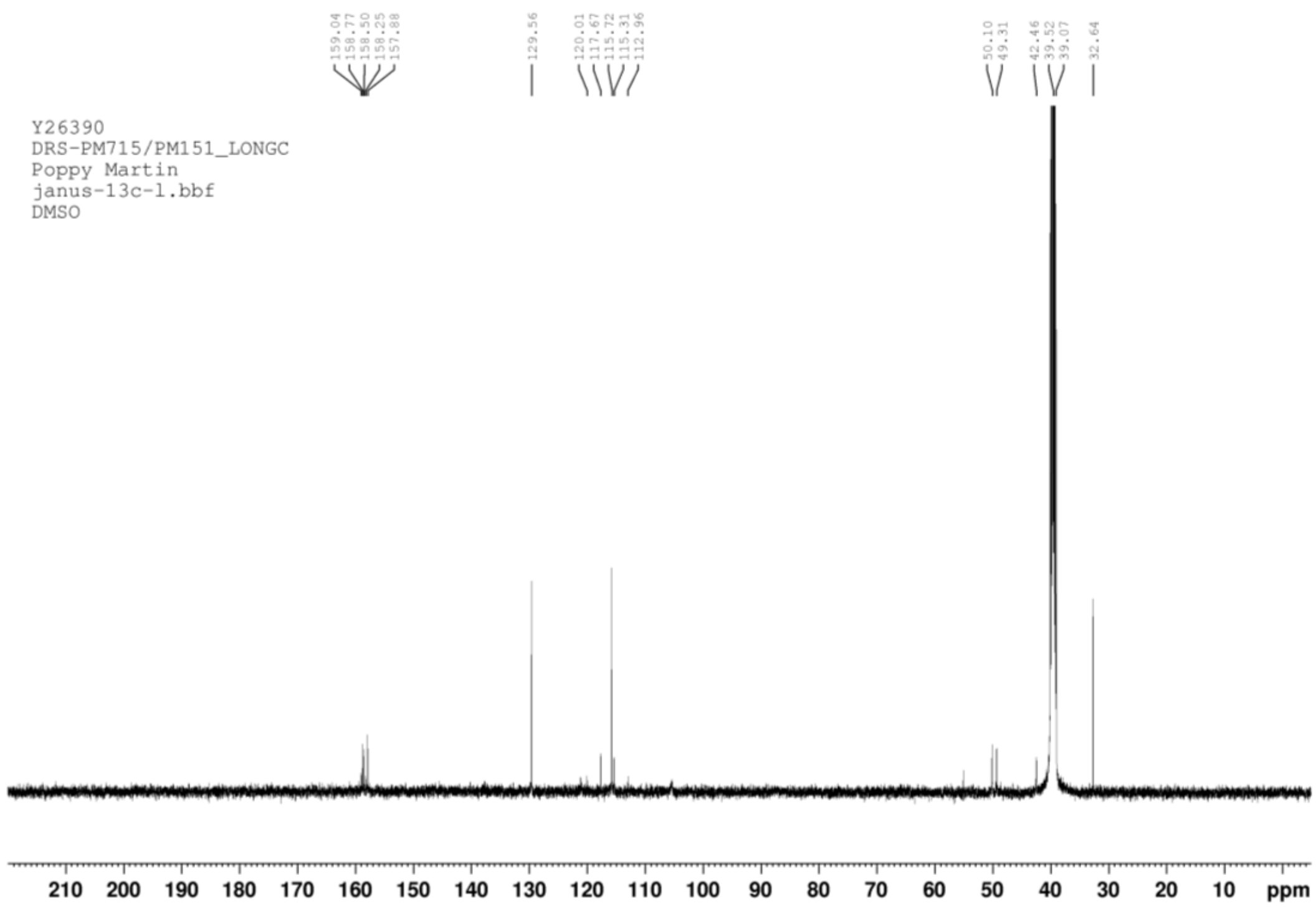

PROTAC A

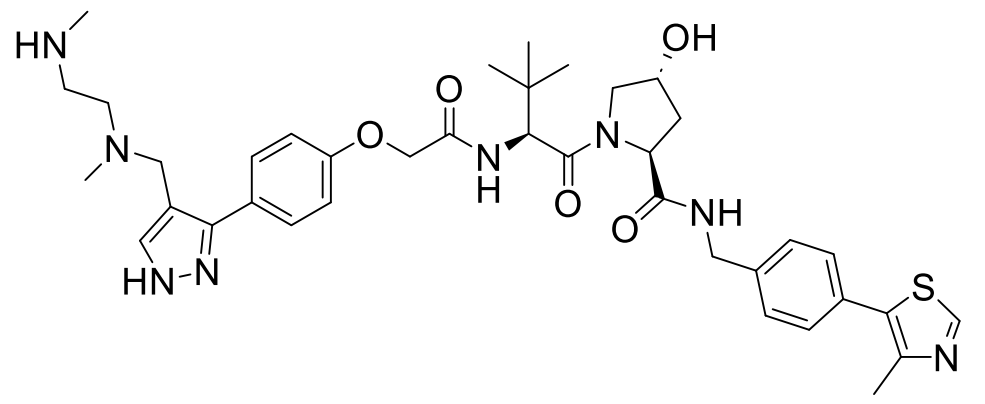
HN
N
HN-N
O
O
H
N
OH
O
N
O
NH
S
N

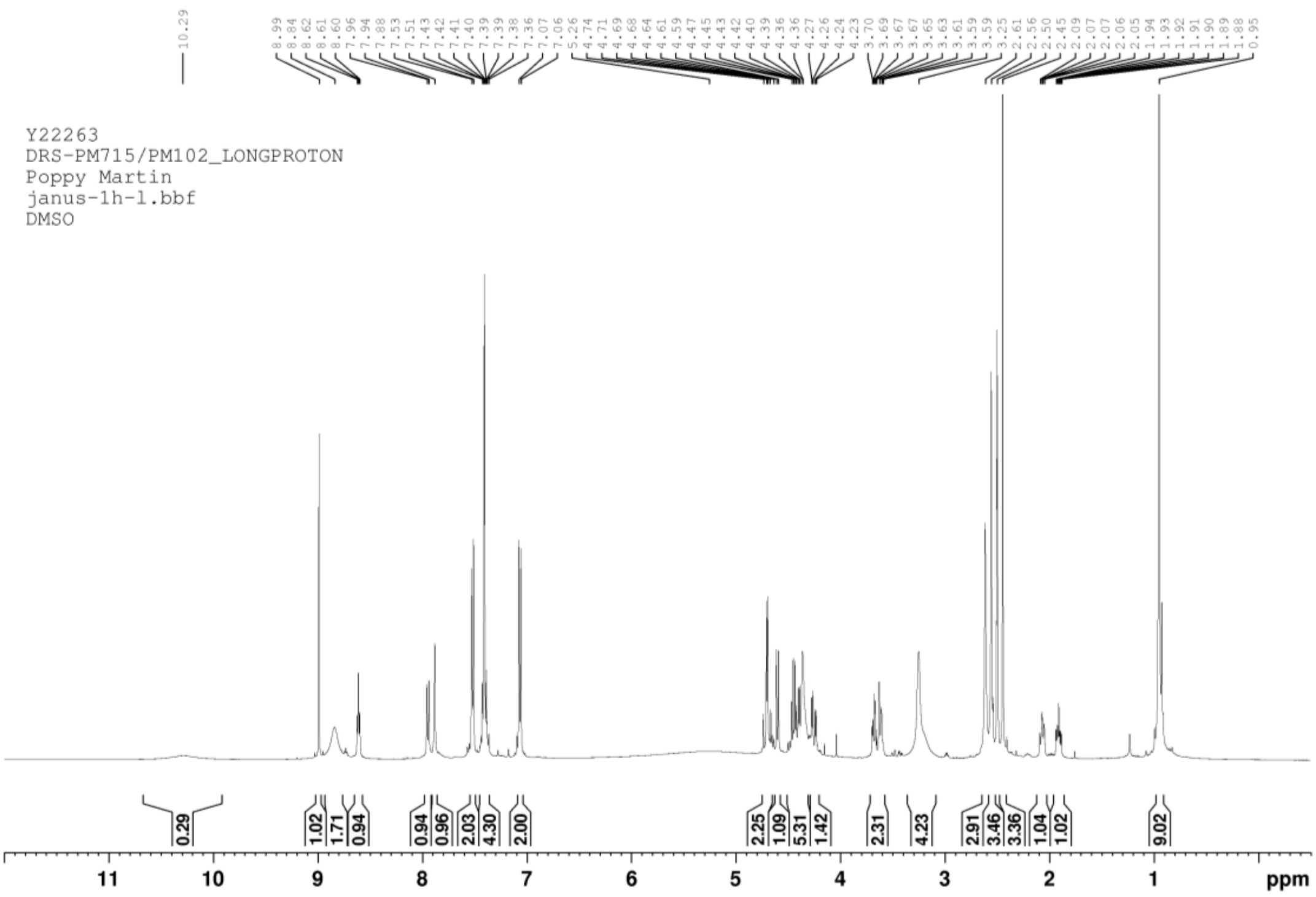
Y22263
DRS-PM715/PM102_LONGPROTON
Poppy Martin
janus-1h-1.bbf
DMSO
ppm

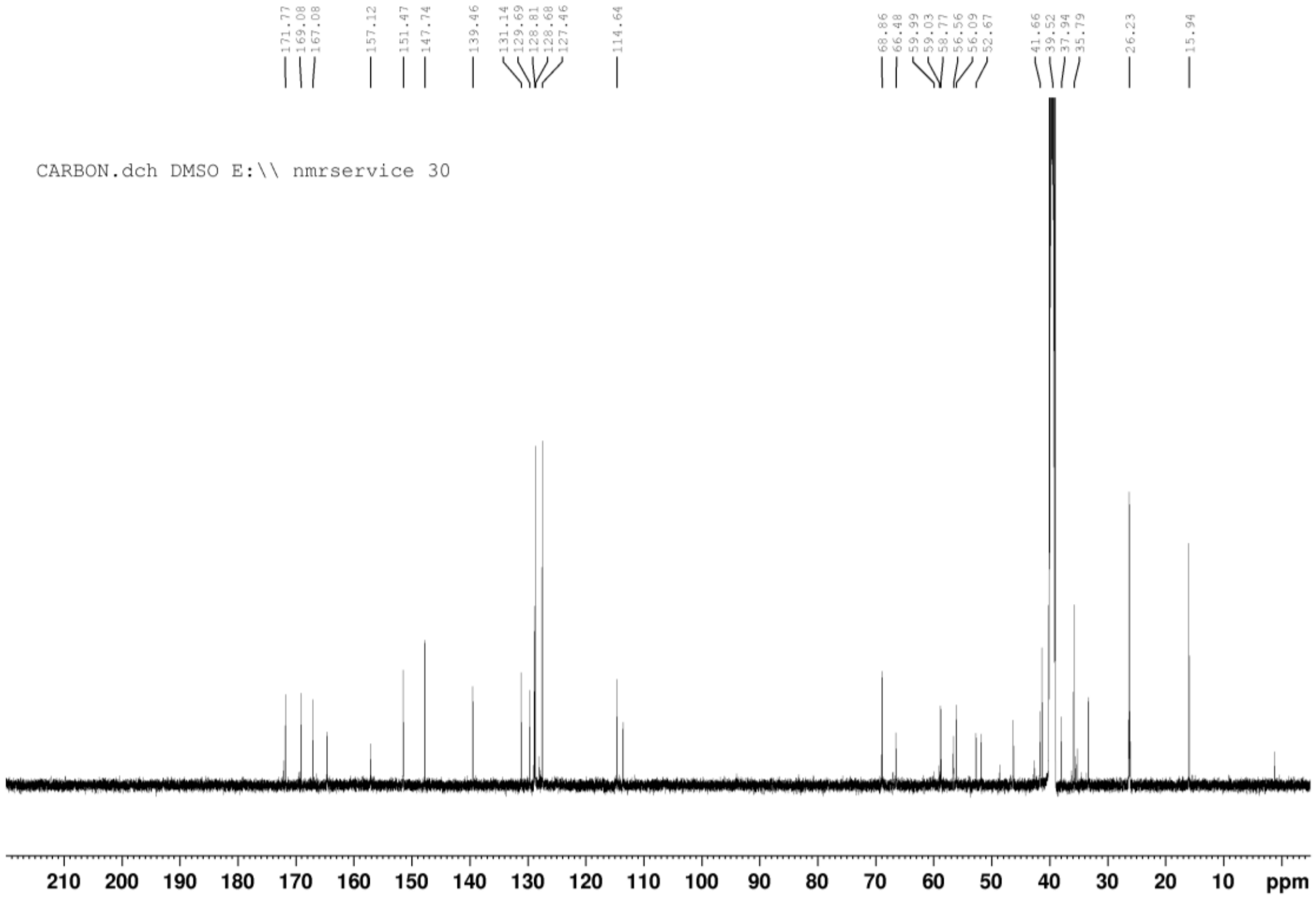
CARBON.dch DMSO E:\\ nmrservice 30
ppm

PROTAC B

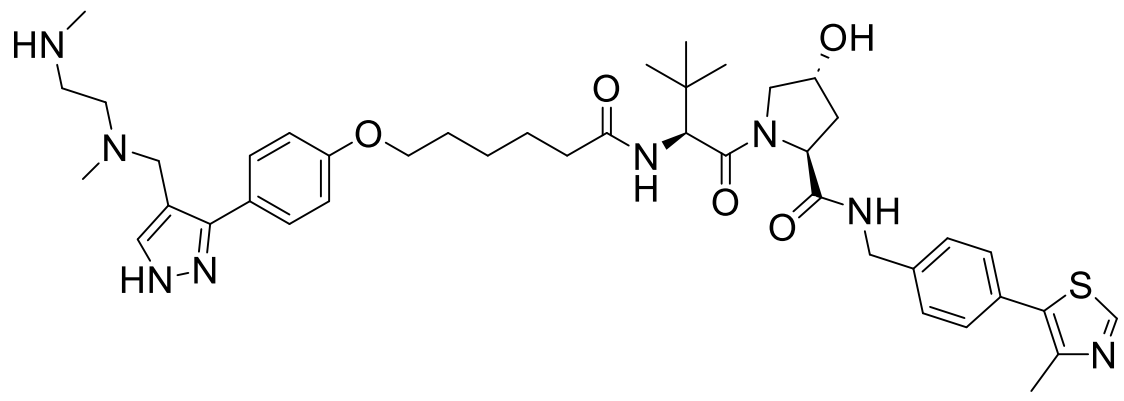

HN
N
O
O
NH
N
N
H
OH
O
NH
O
HN-N
S
N

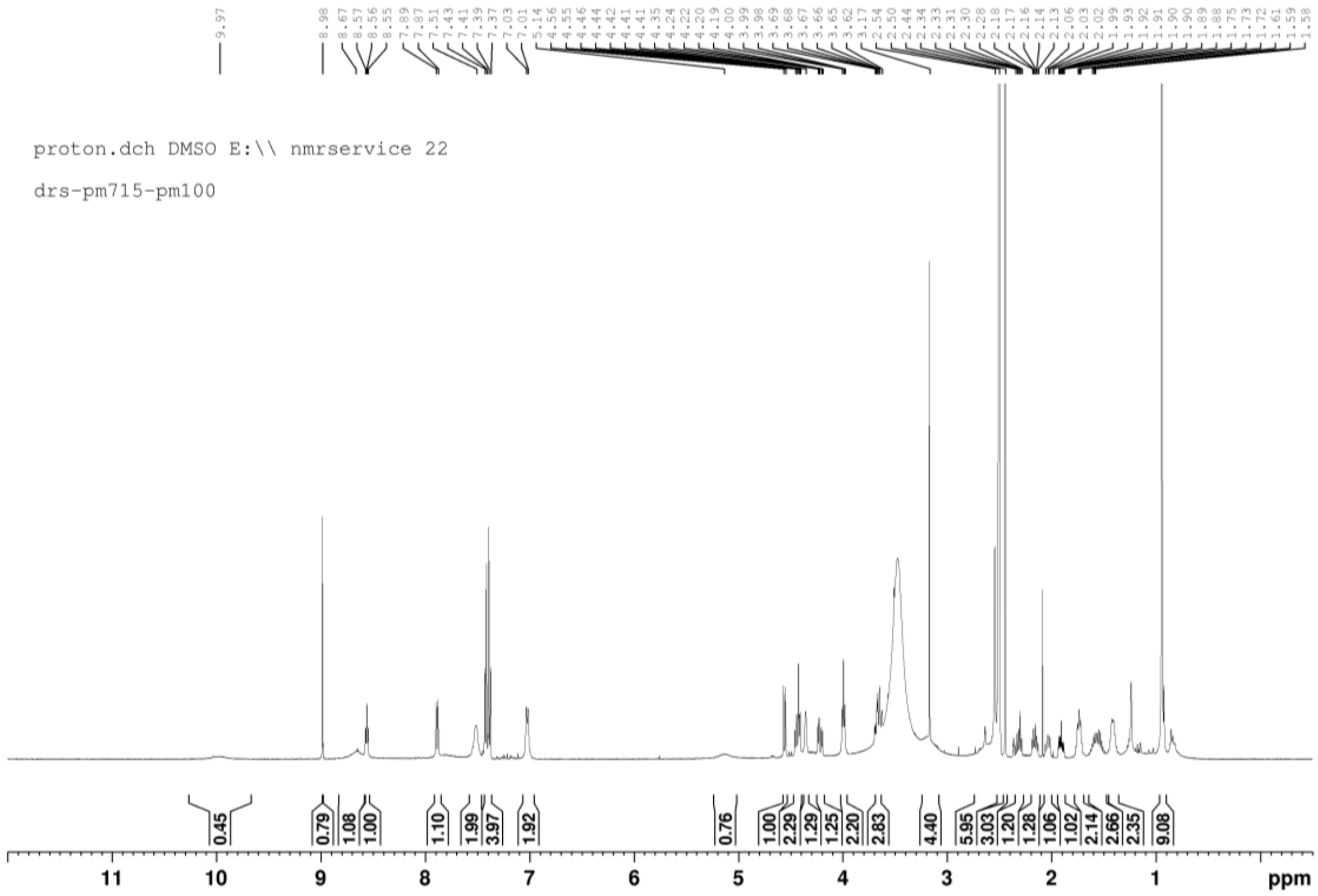

proton.dch DMSO E:\\ nmrservice 22
drs-pm715-pm100
ppm

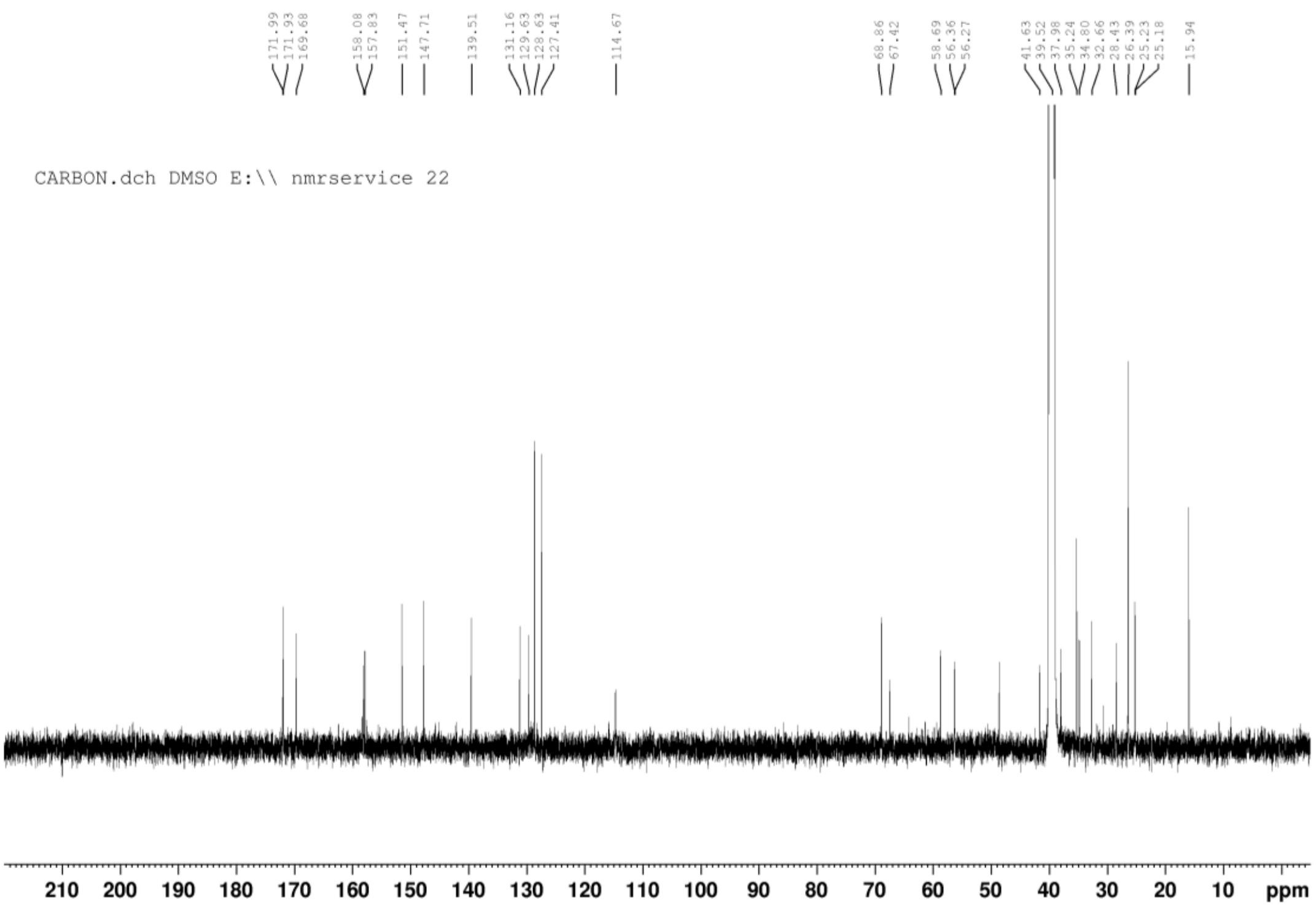

CARBON.dch DMSO E:\\ nmrservice 22
ppm

PROTAC C

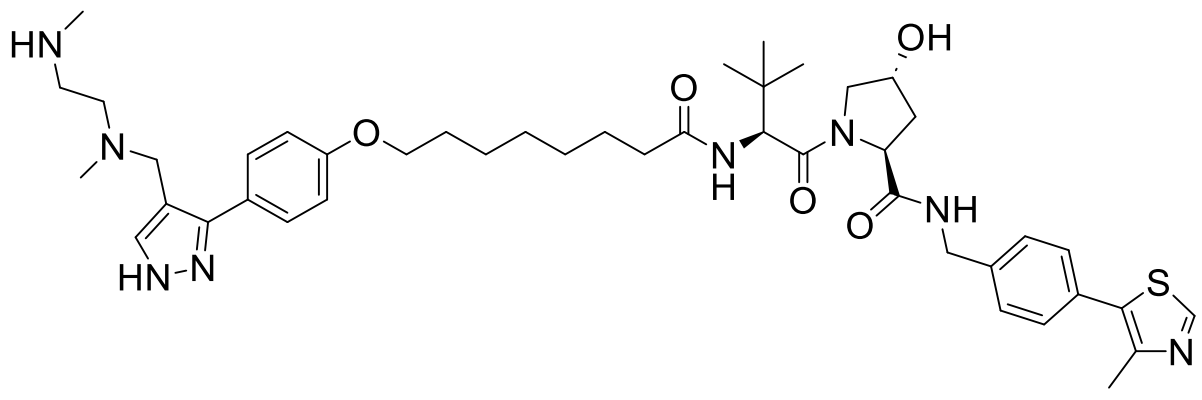
HN
N
O
O
N
H
O
N
OH
O
NH
S
N
HN-N

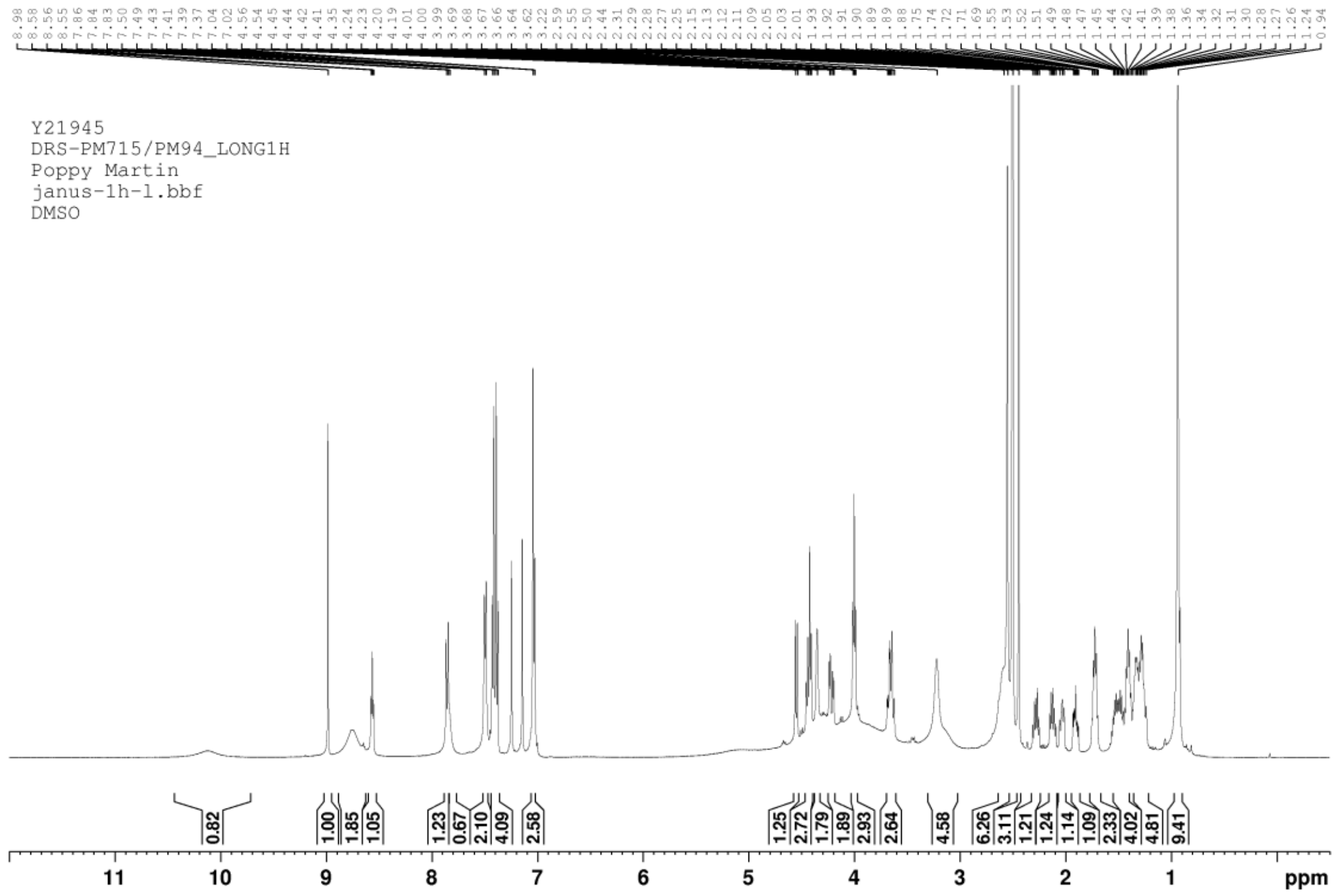
Y21945
DRS-PM715/PM94_LONG1H
Poppy Martin
janus-1h-1.bbf
DMSO
0.82
1.00
1.85
1.05
1.23
0.67
2.10
4.09
2.58
1.25
2.72
1.79
1.89
2.93
2.64
4.58
6.26
3.11
1.21
1.24
1.14
1.09
2.33
4.02
4.81
9.41
11
10
9
8
7
6
5
4
3
2
1
ppm

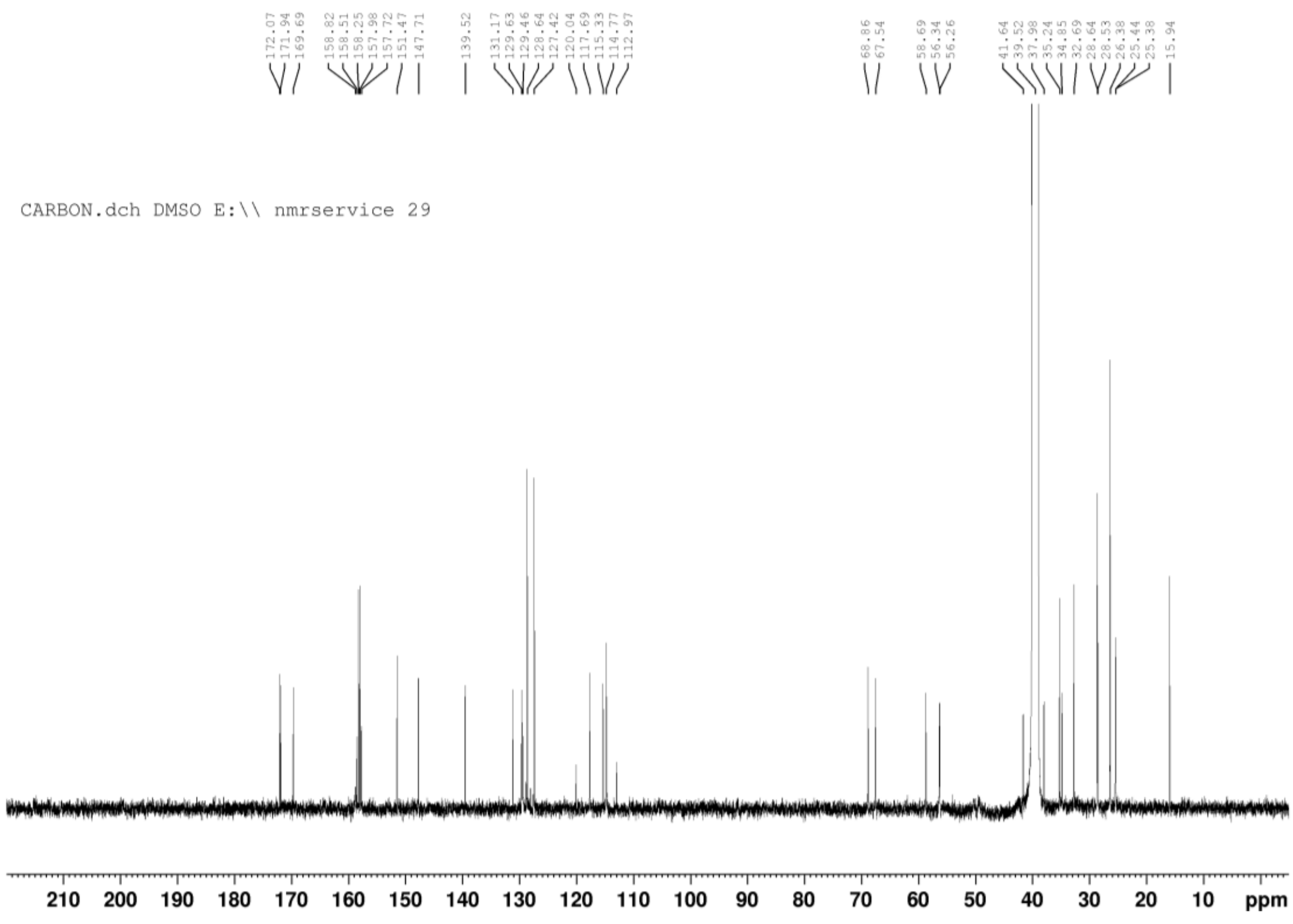
172.07
171.94
169.69
158.82
158.51
158.25
157.98
157.72
151.47
147.71
139.52
131.17
129.63
129.46
128.64
127.42
120.04
117.69
115.33
114.77
112.97
68.86
67.54
58.69
56.34
56.26
41.64
39.52
37.98
35.24
34.85
32.69
28.64
28.53
26.38
25.44
25.38
15.94
CARBON.dch DMSO E:\\ nmrservice 29
210
200
190
180
170
160
150
140
130
120
110
100
90
80
70
60
50
40
30
20
10
ppm

PROTAC D

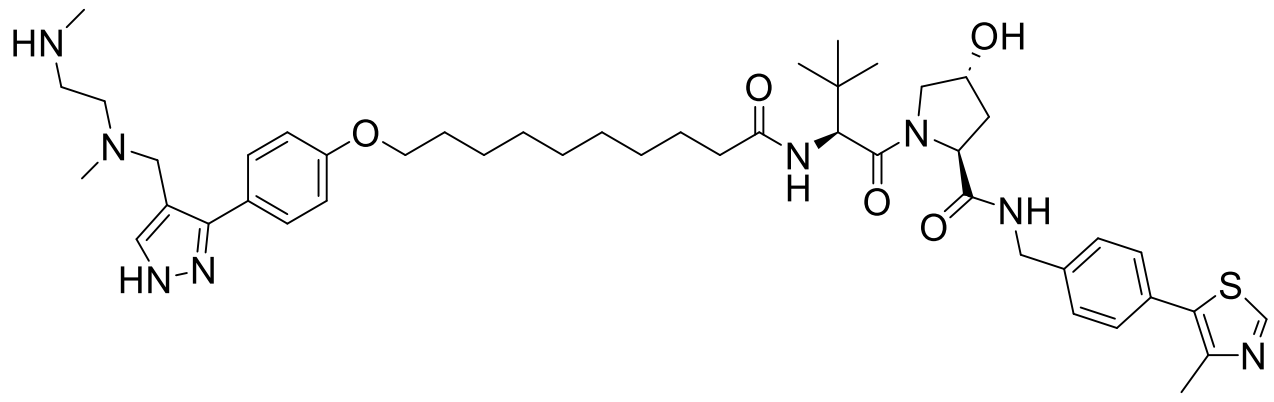

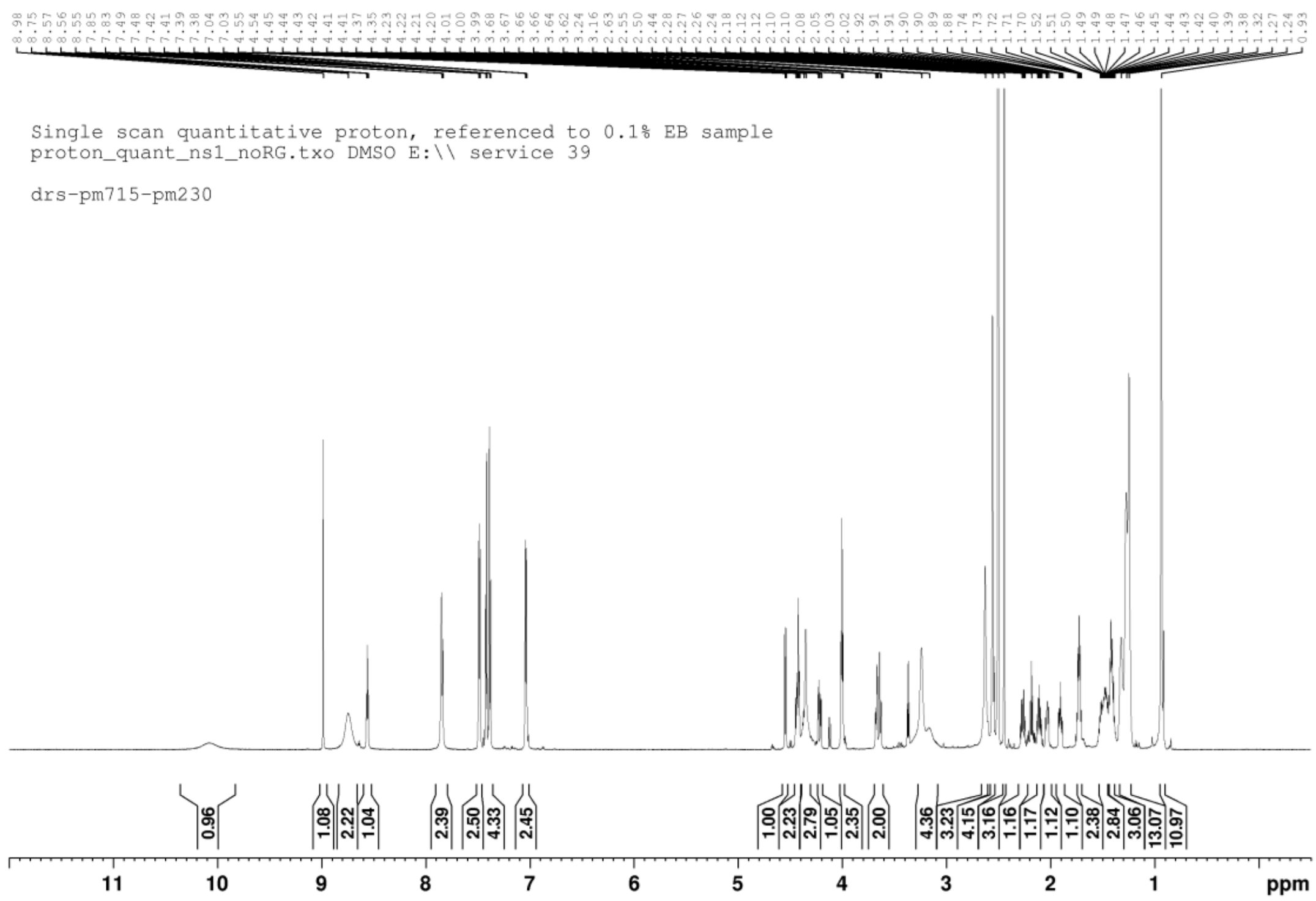
Single scan quantitative proton, referenced to 0.1% EB sample
proton_quant_ns1_noRG.txo DMSO E:\\ service 39
drs-pm715-pm230
ppm

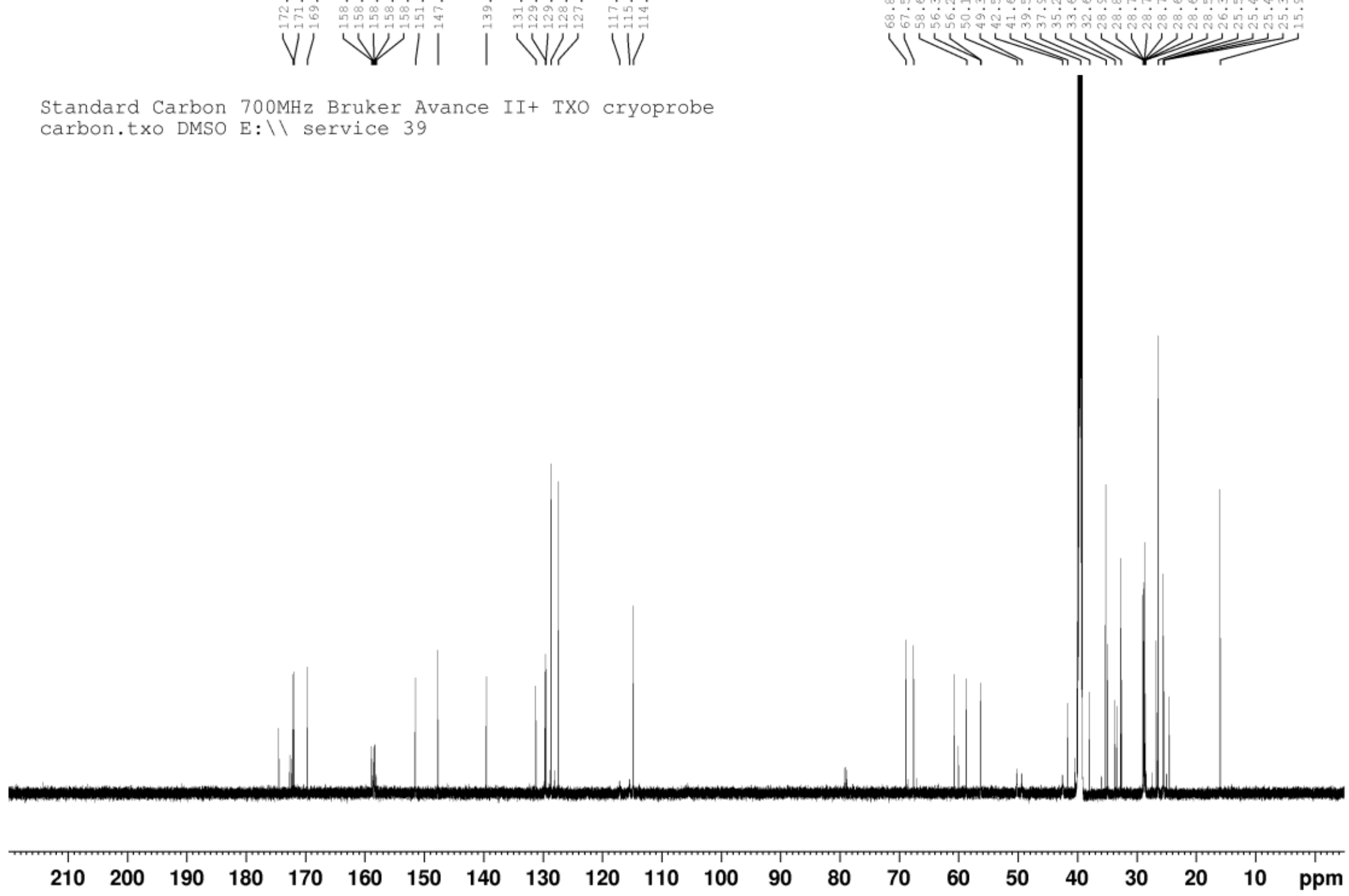
Standard Carbon 700MHz Bruker Avance II+ TXO cryoprobe
carbon.txo DMSO E:\\ service 39
ppm

PROTAC E

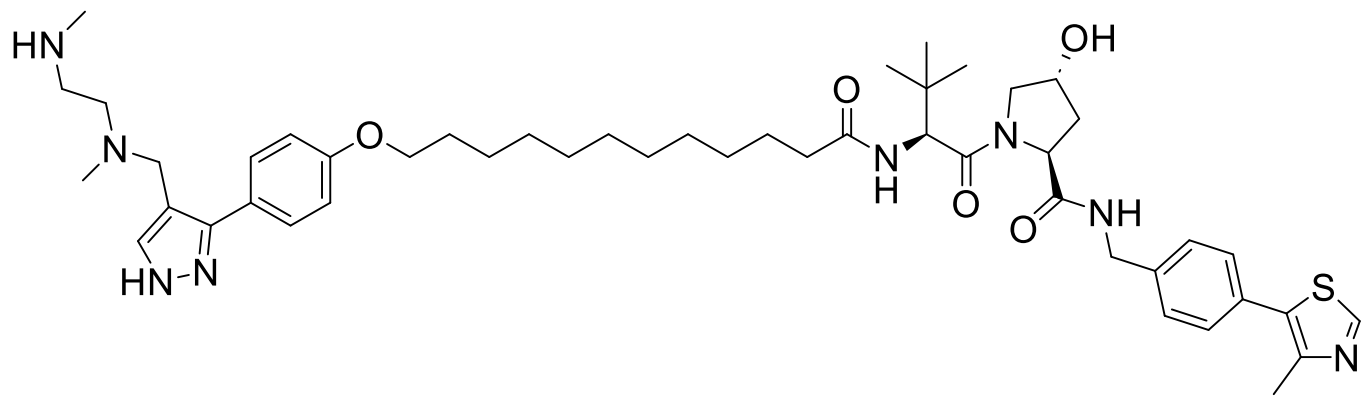

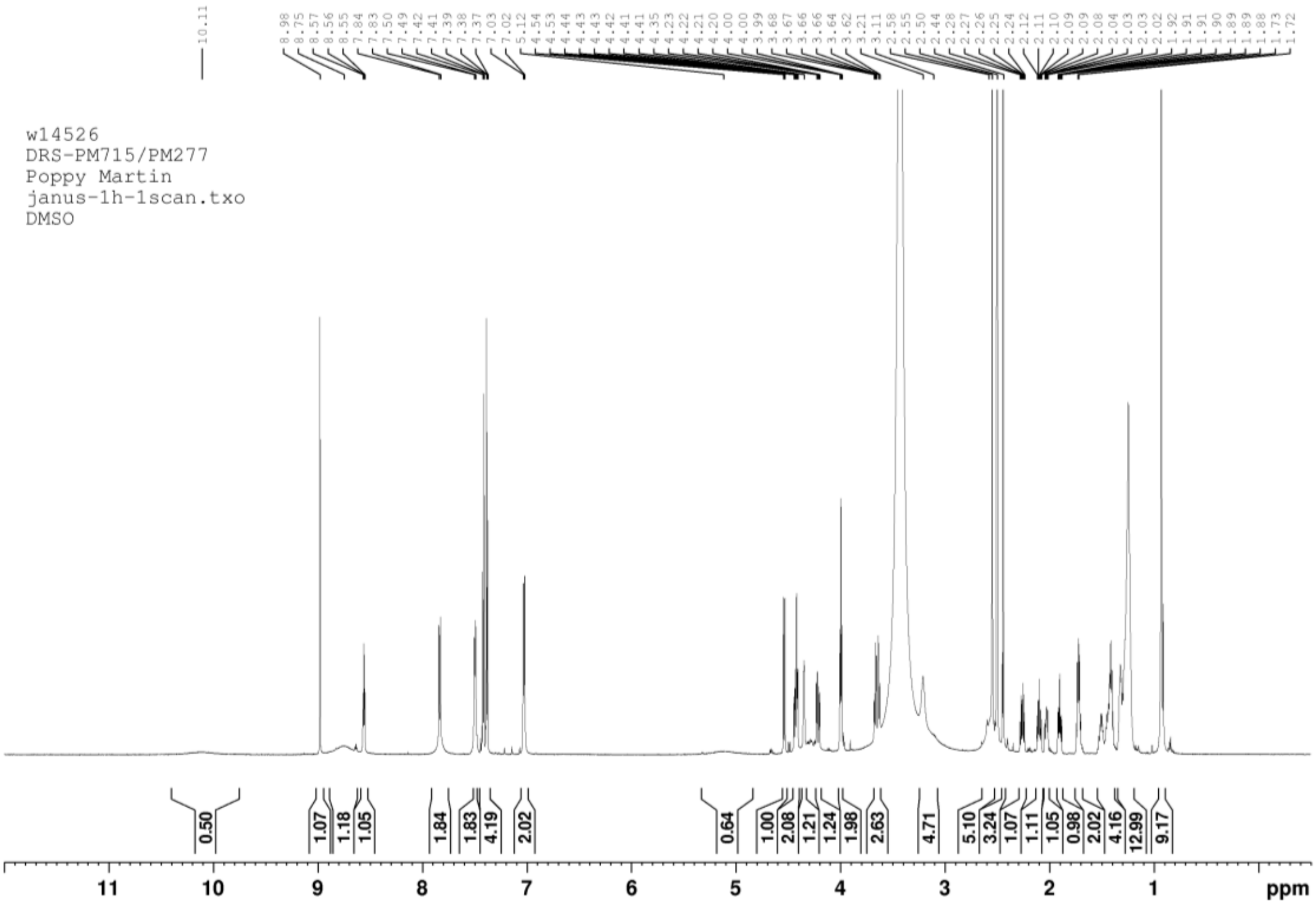
w14526
DRS-PM715/PM277
Poppy Martin
janus-1h-1scan.txo
DMSO
ppm

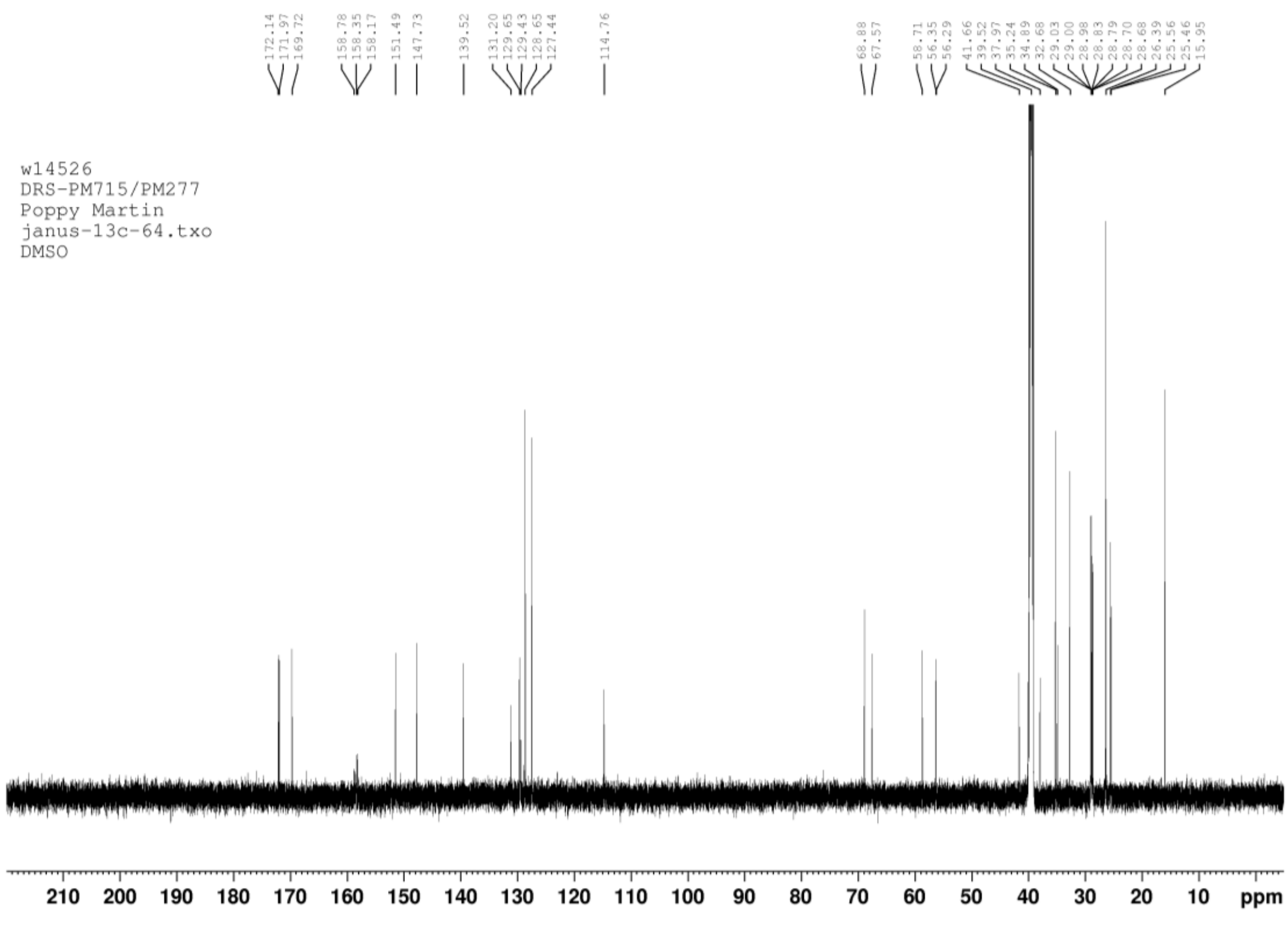
w14526
DRS-PM715/PM277
Poppy Martin
janus-13c-64.txo
DMSO
ppm

PROTAC F

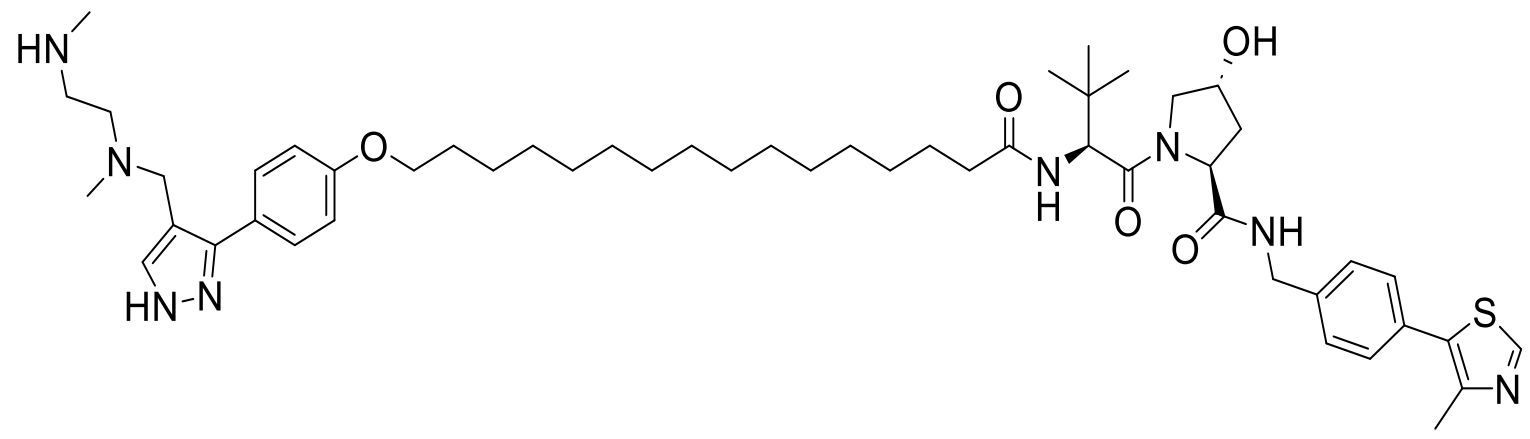
HN
N
HN-N
O
O
N
H
O
OH
N
O
NH
S
N

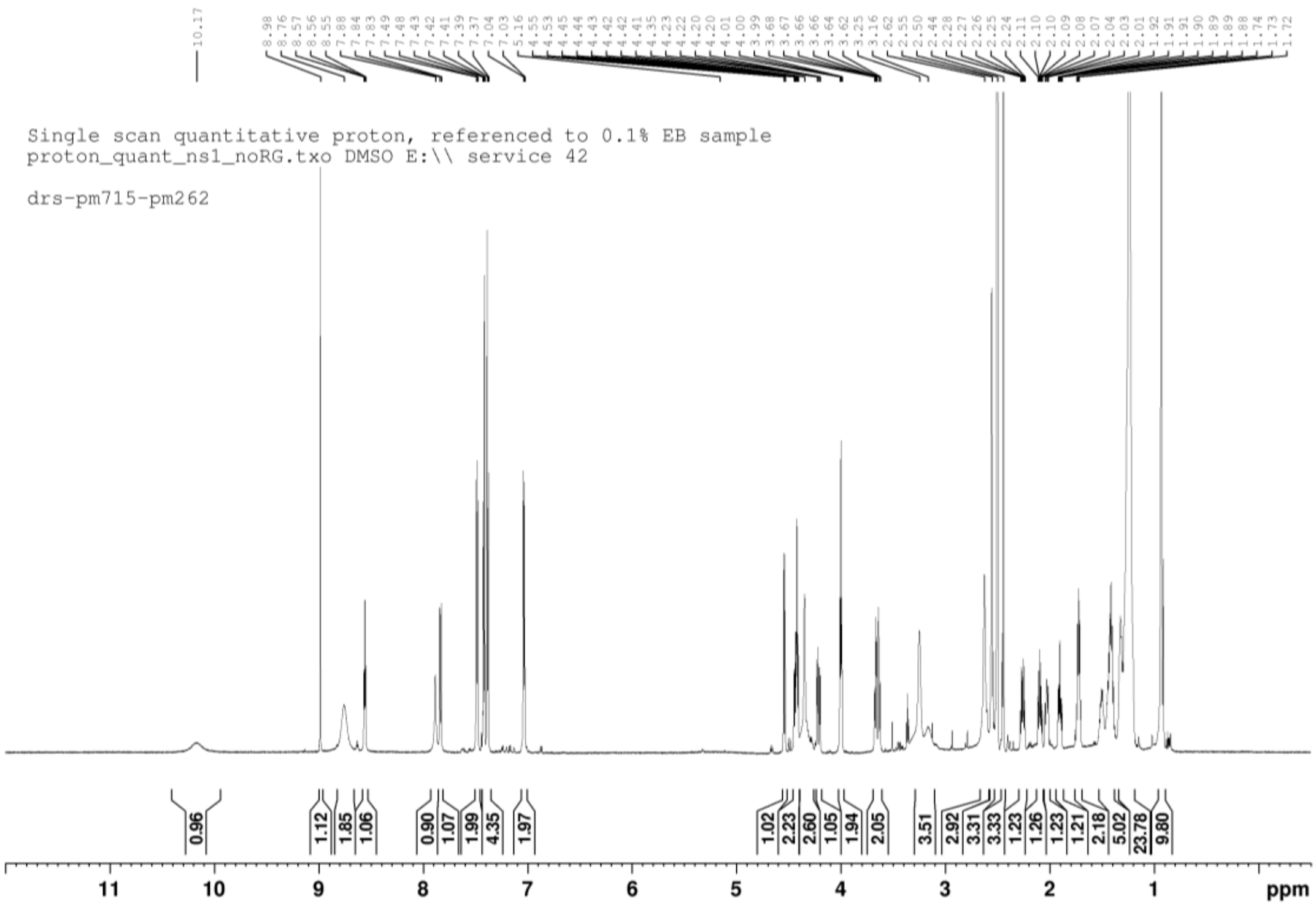
Single scan quantitative proton, referenced to 0.1% EB sample
proton_quant_ns1_noRG.txo DMSO E:\\ service 42
drs-pm715-pm262
ppm

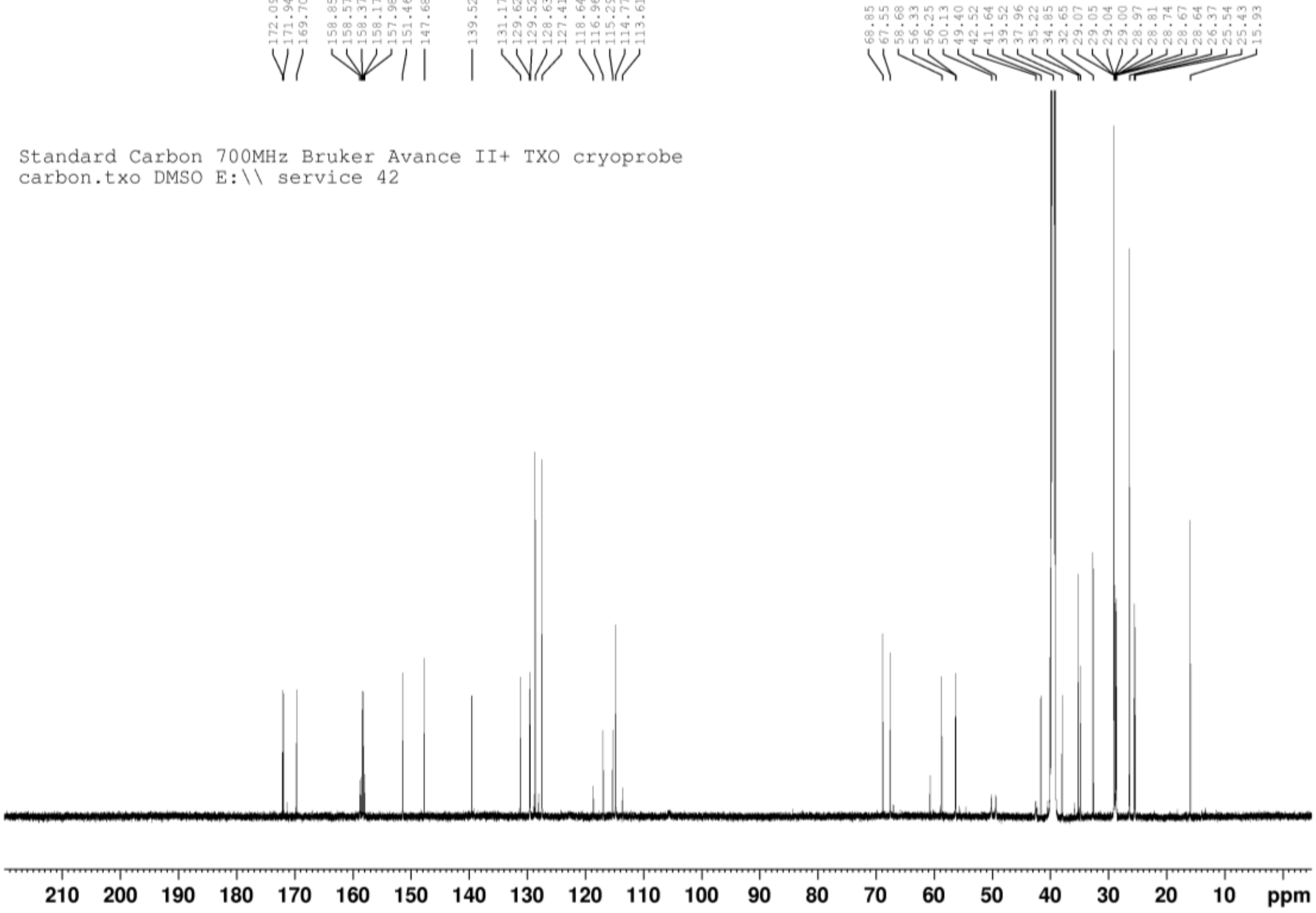
Standard Carbon 700MHz Bruker Avance II+ TXO cryoprobe
carbon.txo DMSO E:\\ service 42
ppm

PROTAC G

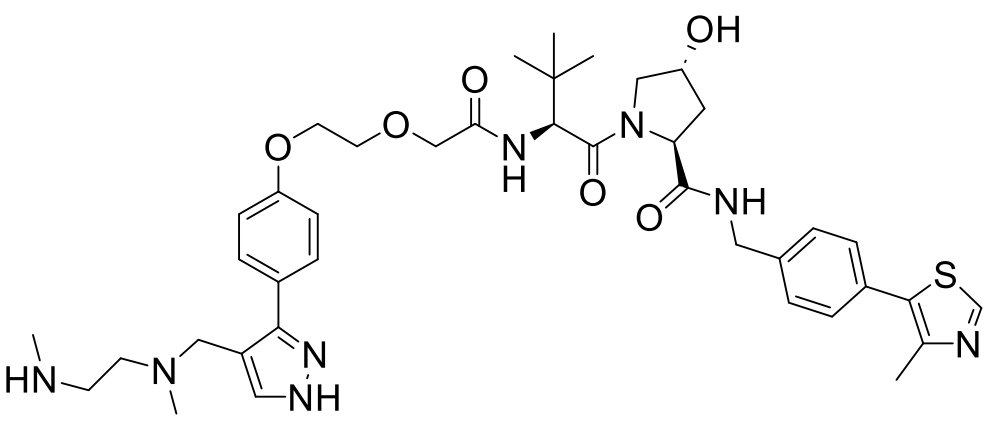

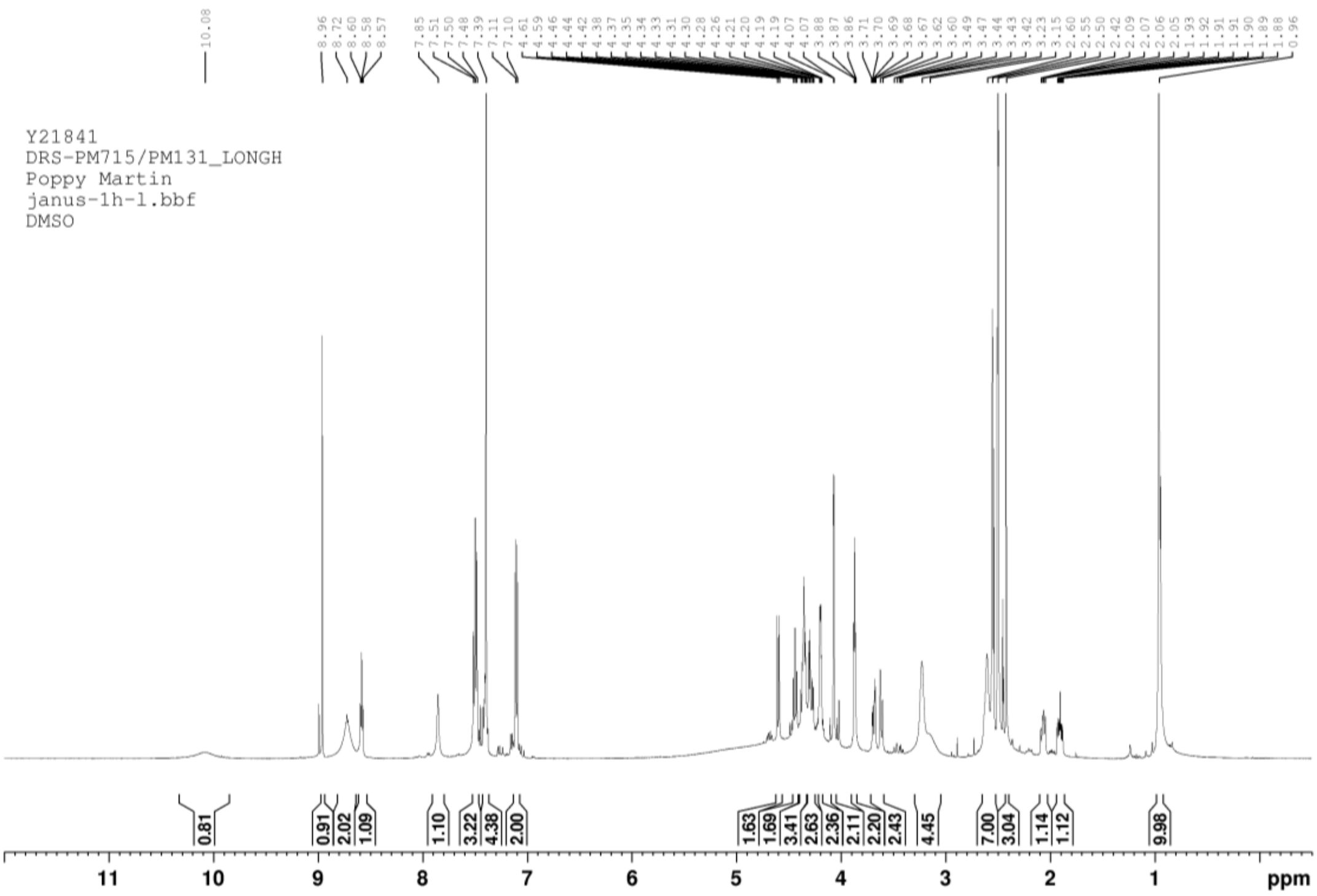
Y21841
DRS-PM715/PM131_LONGH
Poppy Martin
janus-1h-1.bbf
DMSO
ppm

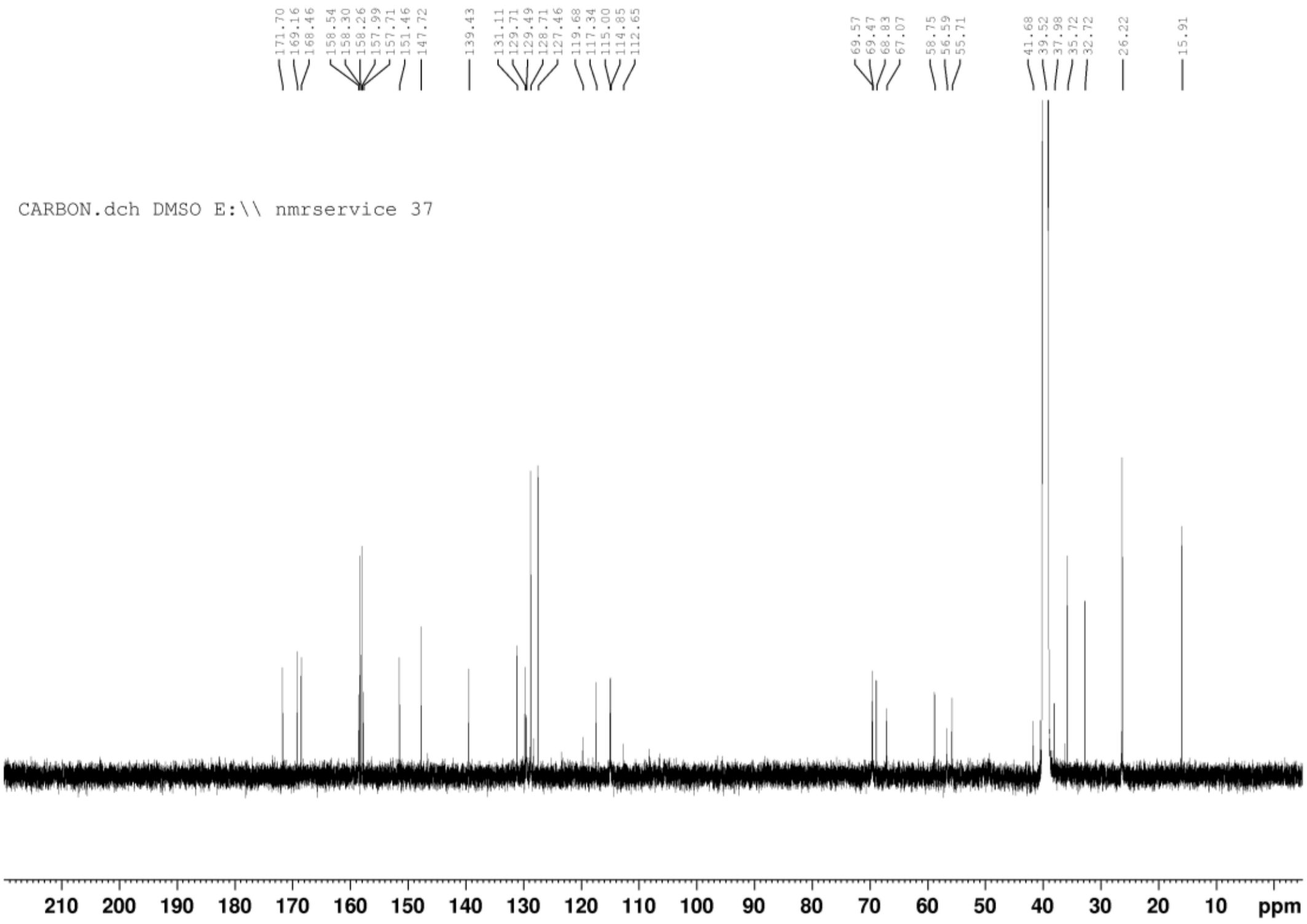
171.70 169.16 168.46 158.54 158.30 158.26 157.99 157.71 151.46 147.72 139.43 131.11 129.71 129.49 128.71 127.46 119.68 117.34 115.00 114.85 112.65 69.57 69.47 68.83 67.07 58.75 56.59 55.71 41.68 39.52 37.98 35.72 32.72 26.22 15.91
CARBON.dch DMSO E:\\ nmrservice 37
ppm

PROTAC H

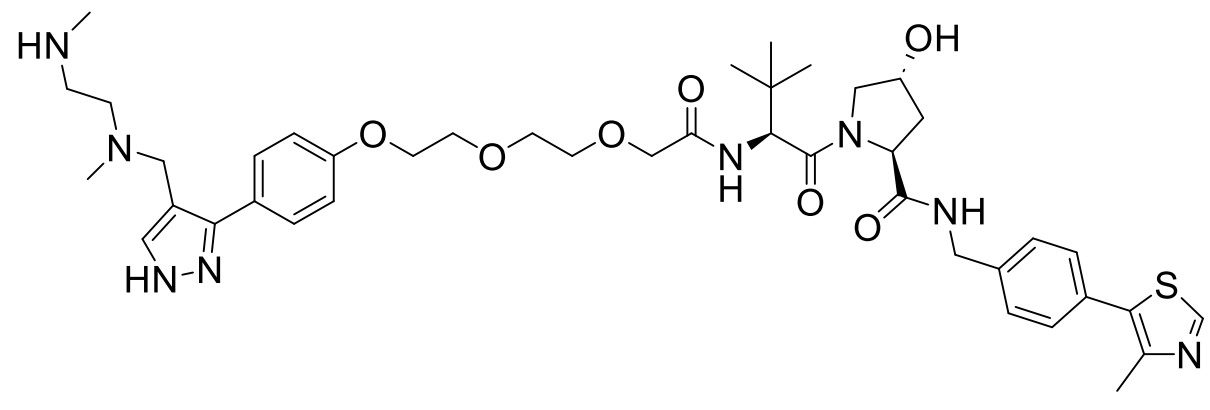

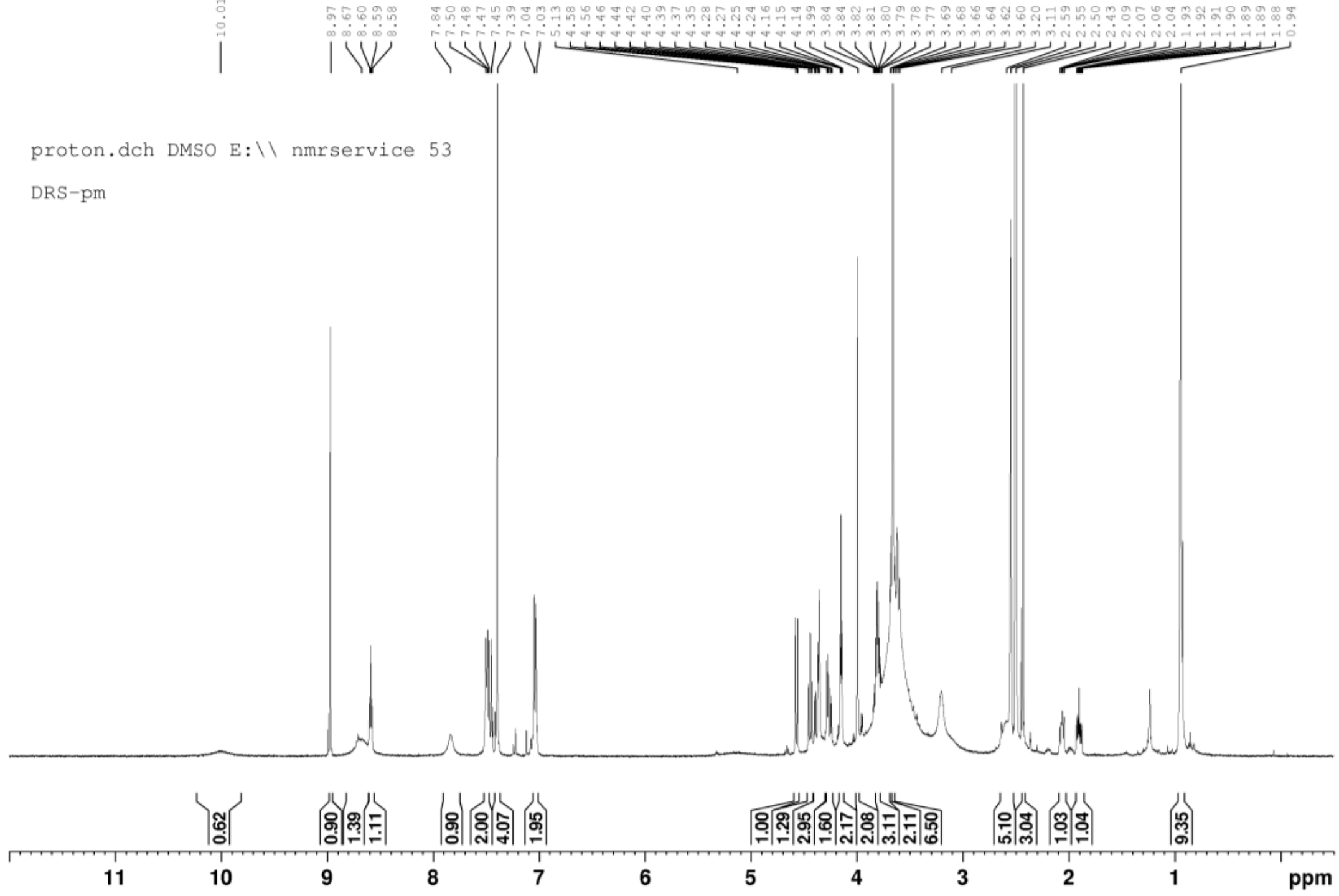
proton.dch DMSO E:\\ nmrservice 53
DRS-pm
ppm

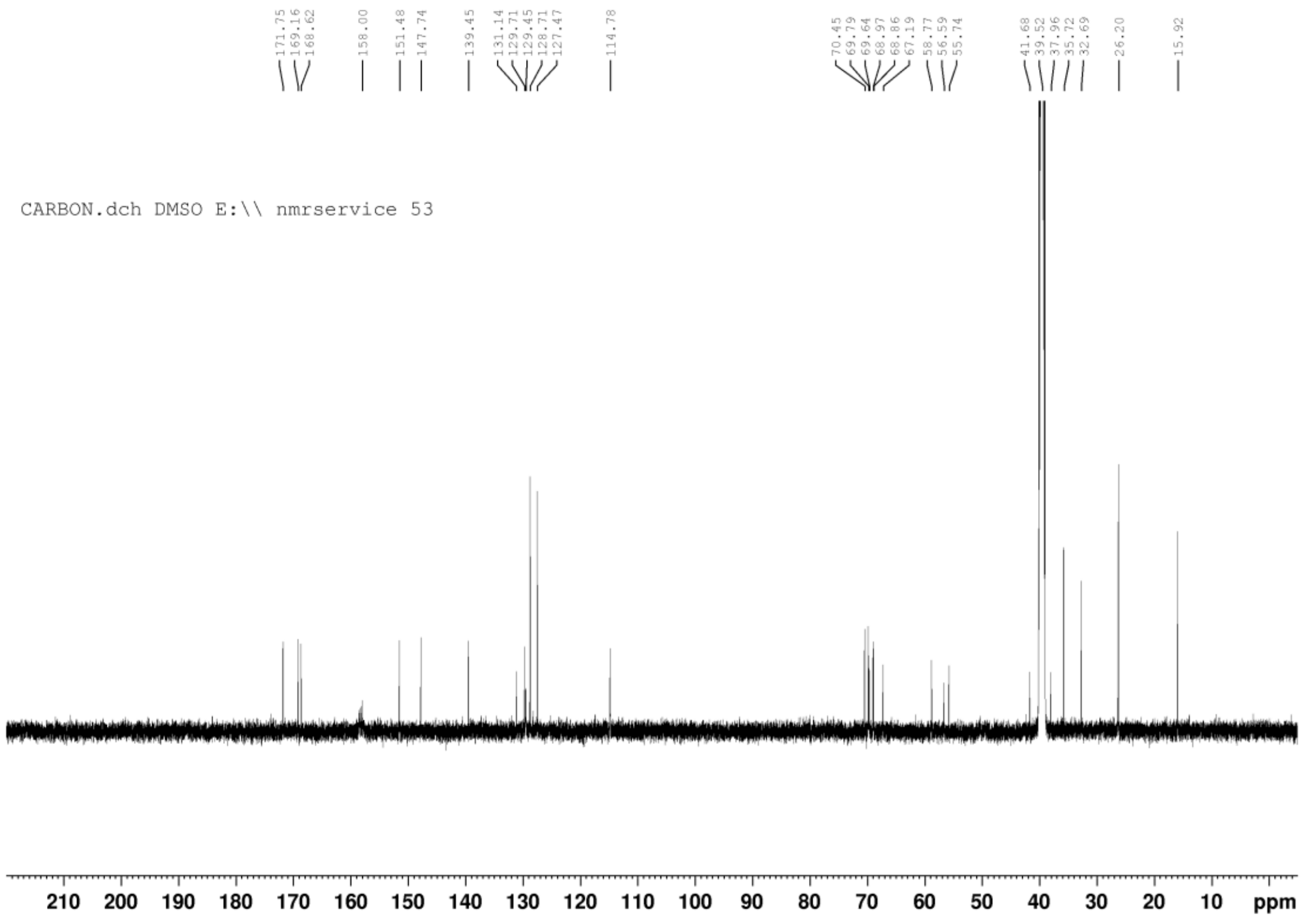
CARBON.dch DMSO E:\\ nmrservice 53
ppm

PROTAC I

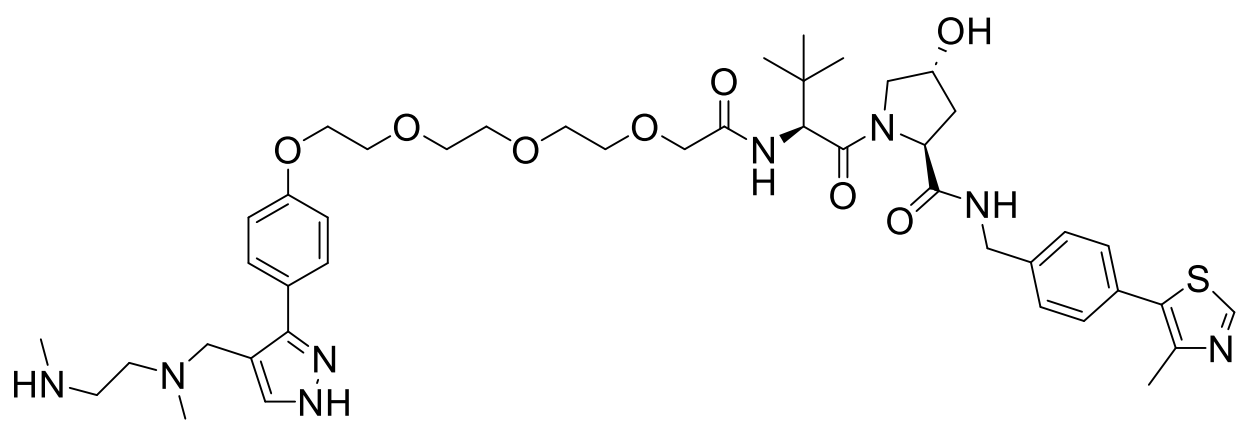

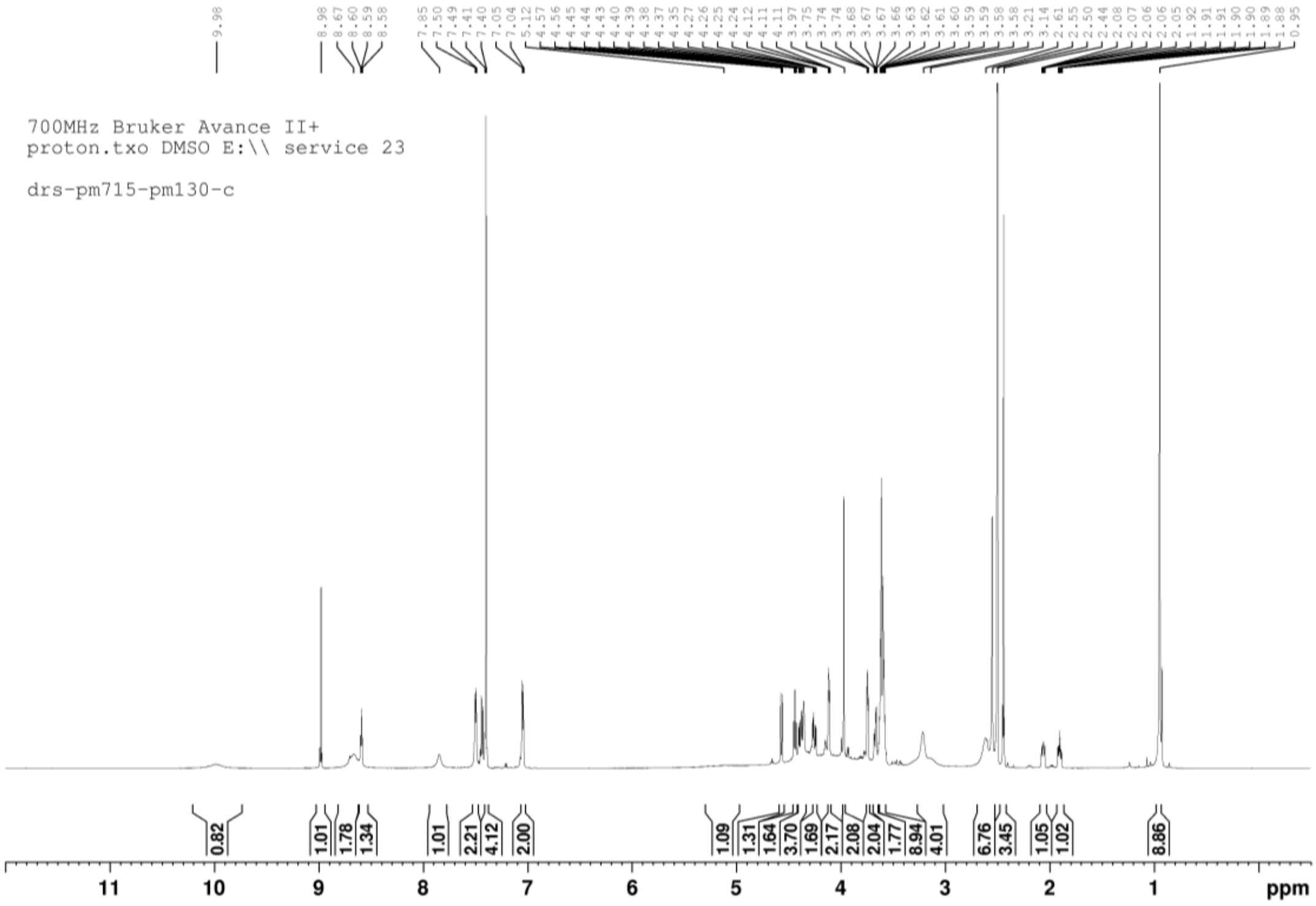
700MHz Bruker Avance II+
proton.txo DMSO E:\\ service 23
drs-pm715-pm130-c
ppm

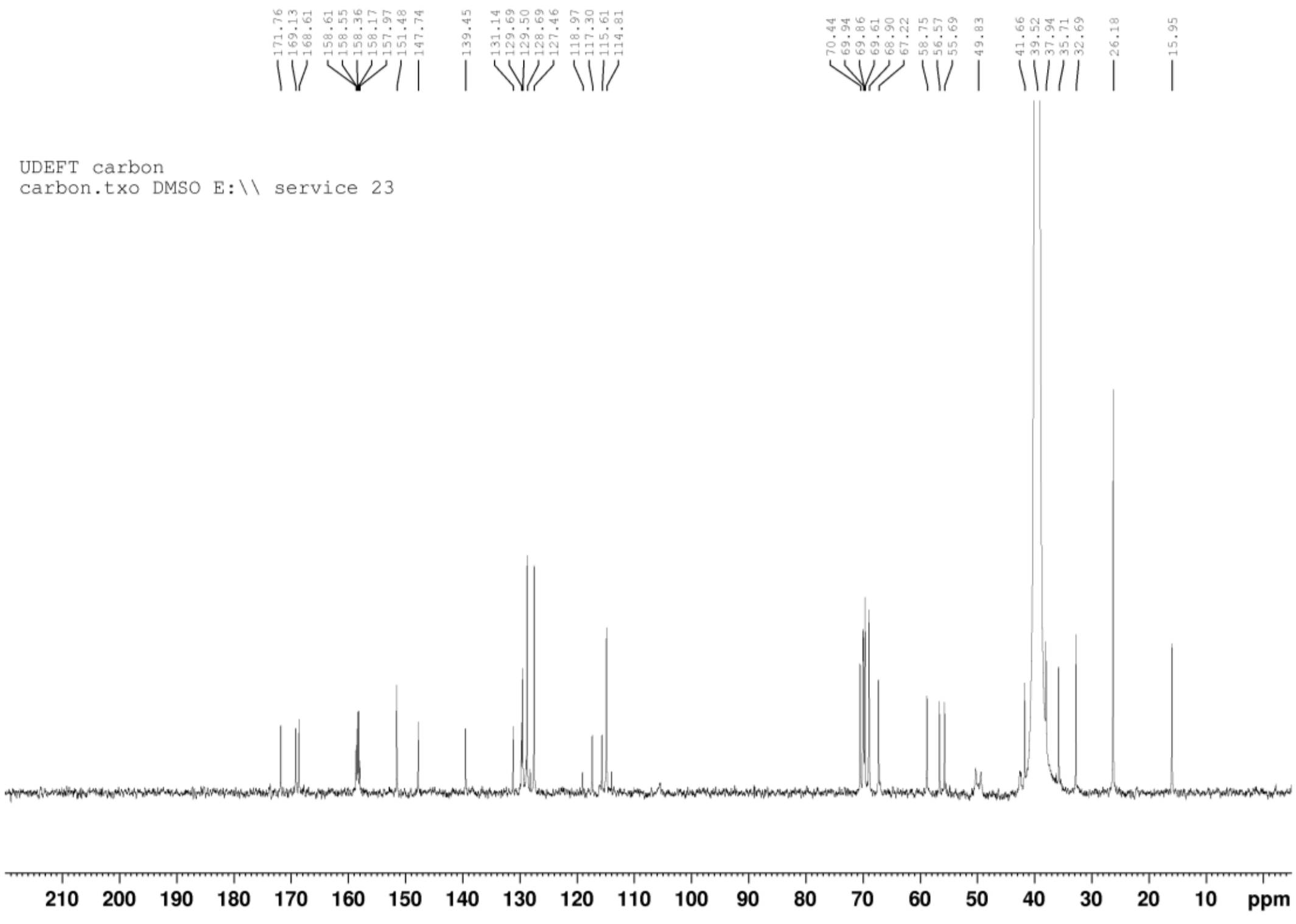
UDEFT carbon
carbon.txo DMSO E:\\ service 23
ppm

(5)

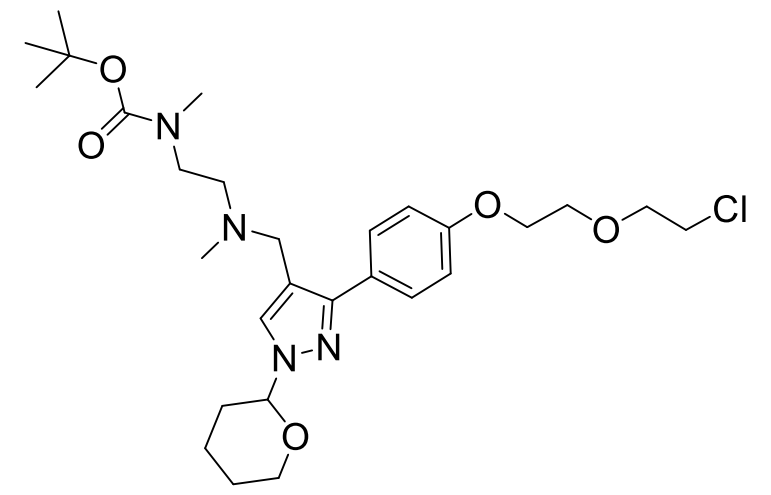
O
O
N
N
O
O
Cl
N
N
O

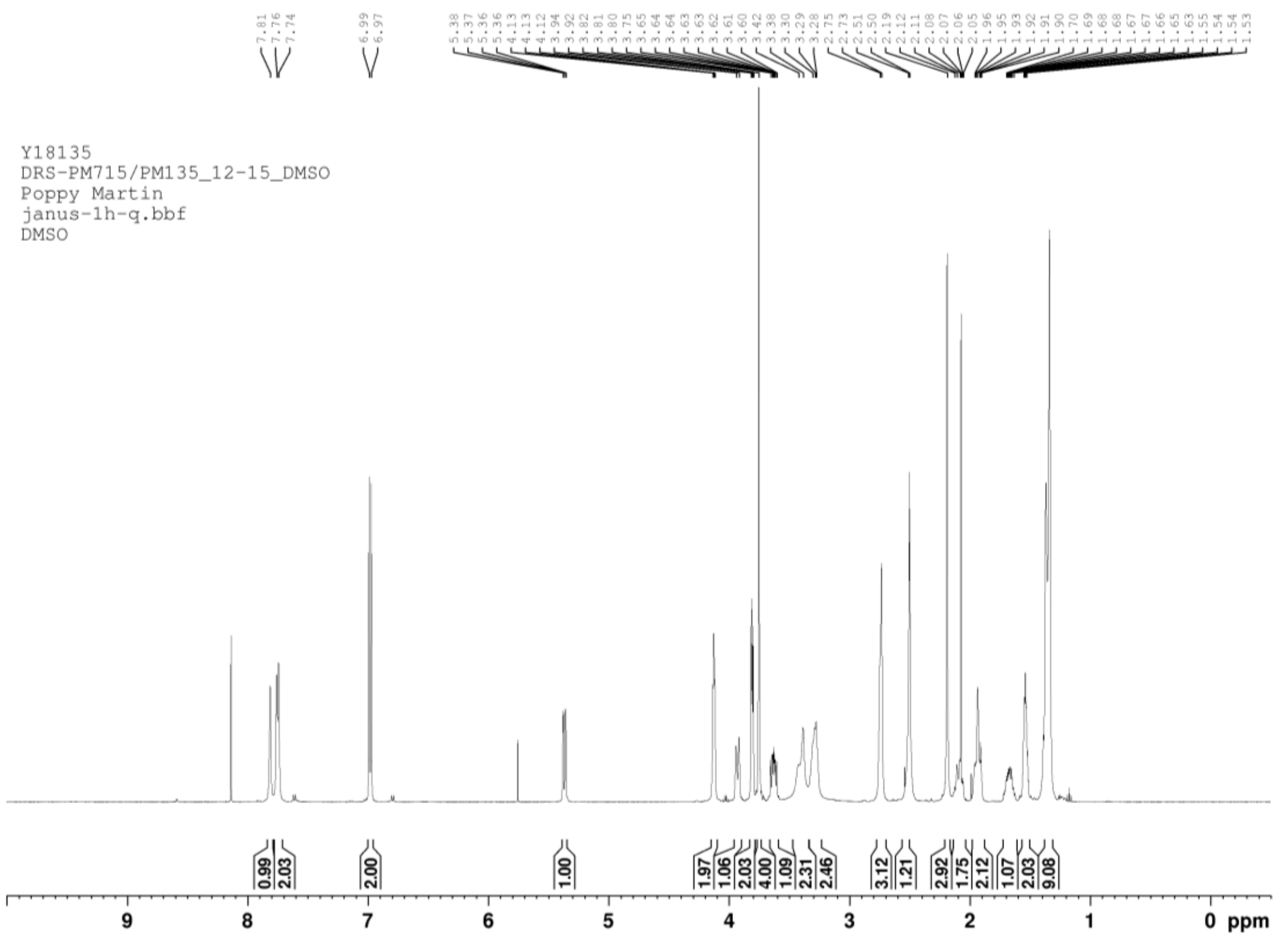
Y18135
DRS-PM715/PM135_12-15_DMSO
Poppy Martin
janus-1h-q.bbf
DMSO
ppm

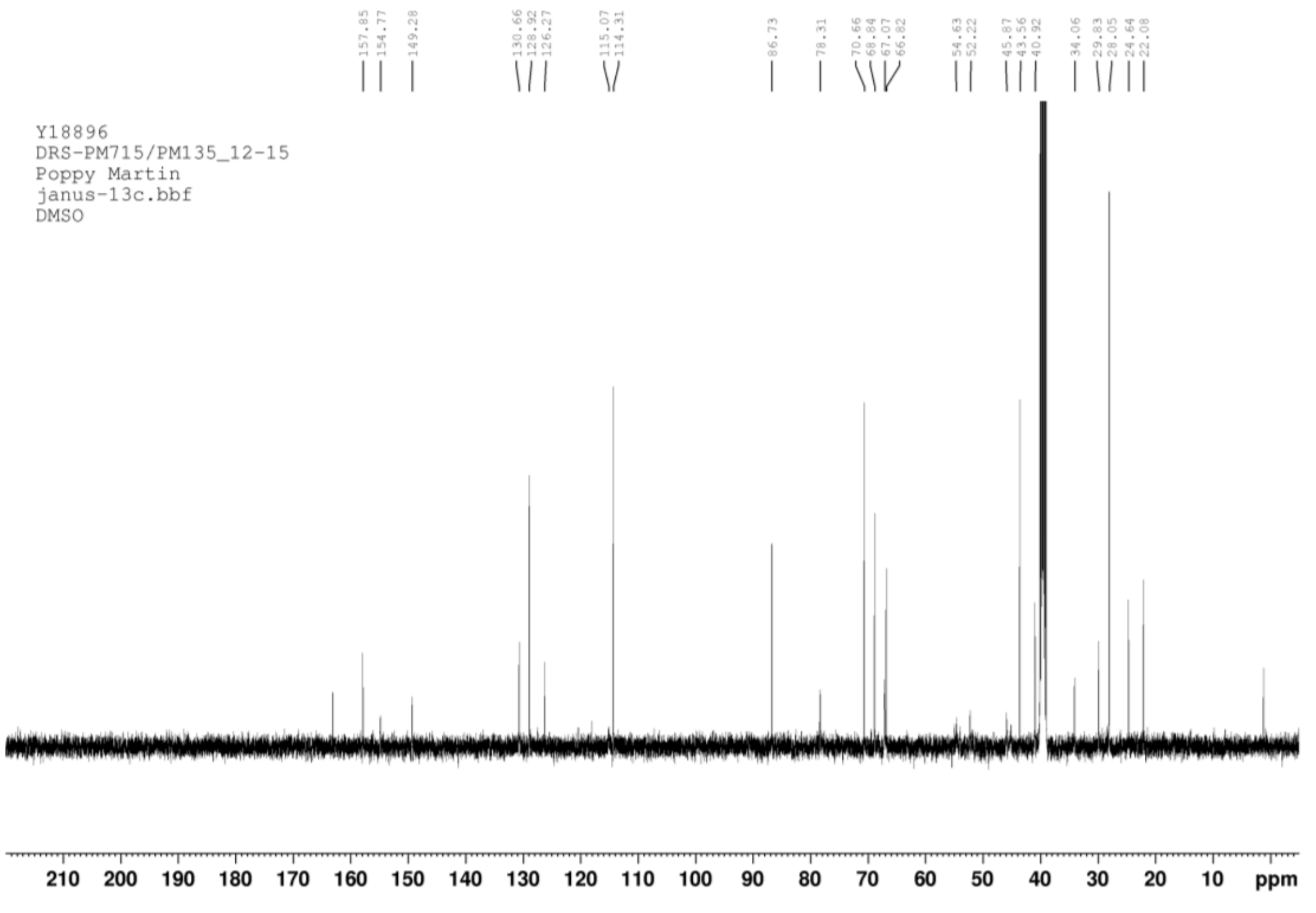
157.85
154.77
149.28
130.66
128.92
126.27
115.07
114.31
86.73
78.31
70.66
68.84
67.07
66.82
54.63
52.22
45.87
43.56
40.92
34.06
29.83
28.05
24.64
22.08
Y18896
DRS-PM715/PM135_12-15
Poppy Martin
janus-13c.bbf
DMSO
ppm

(7)

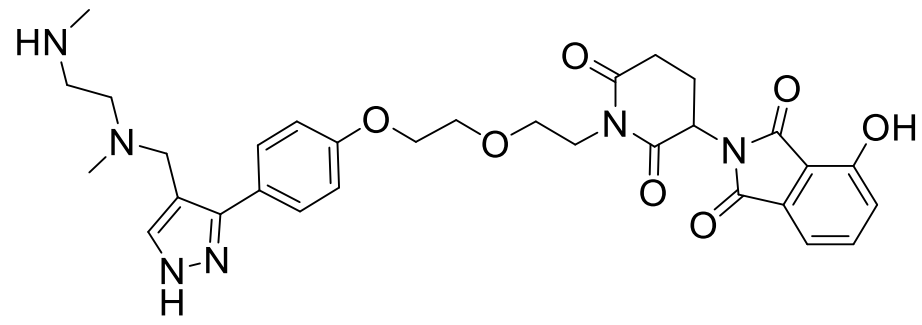
HN
N
O
O
N
O
O
N
O
O
OH
N
N
H

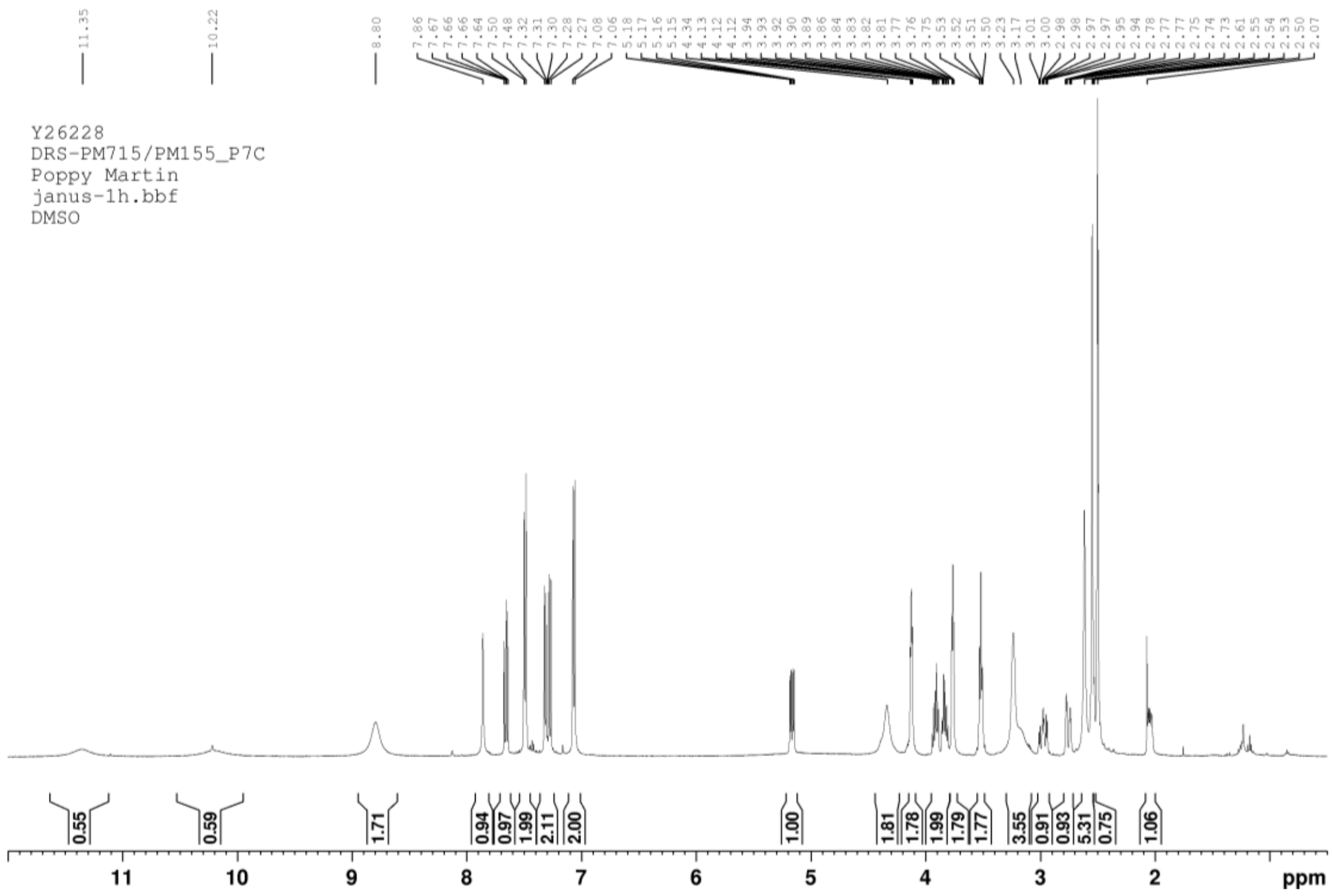
11.35
10.22
8.80
7.86 7.67 7.66 7.66 7.64 7.50 7.48 7.32 7.31 7.30 7.28 7.27 7.08 7.06 5.18 5.17 5.16 5.15 4.34 4.13 4.12 4.12 3.94 3.93 3.92 3.90 3.89 3.86 3.84 3.83 3.82 3.81 3.77 3.76 3.75 3.53 3.52 3.51 3.50 3.23 3.17 3.01 3.00 2.98 2.98 2.97 2.97 2.95 2.94 2.78 2.77 2.77 2.75 2.74 2.73 2.61 2.55 2.54 2.53 2.50 2.07
Y26228
DRS-PM715/PM155_P7C
Poppy Martin
janus-1h.bbf
DMSO
0.55
0.59
1.71
0.94
0.97
1.99
2.11
2.00
1.00
1.81
1.78
1.99
1.79
1.77
3.55
0.91
0.93
5.31
0.75
1.06
11
10
9
8
7
6
5
4
3
2
ppm

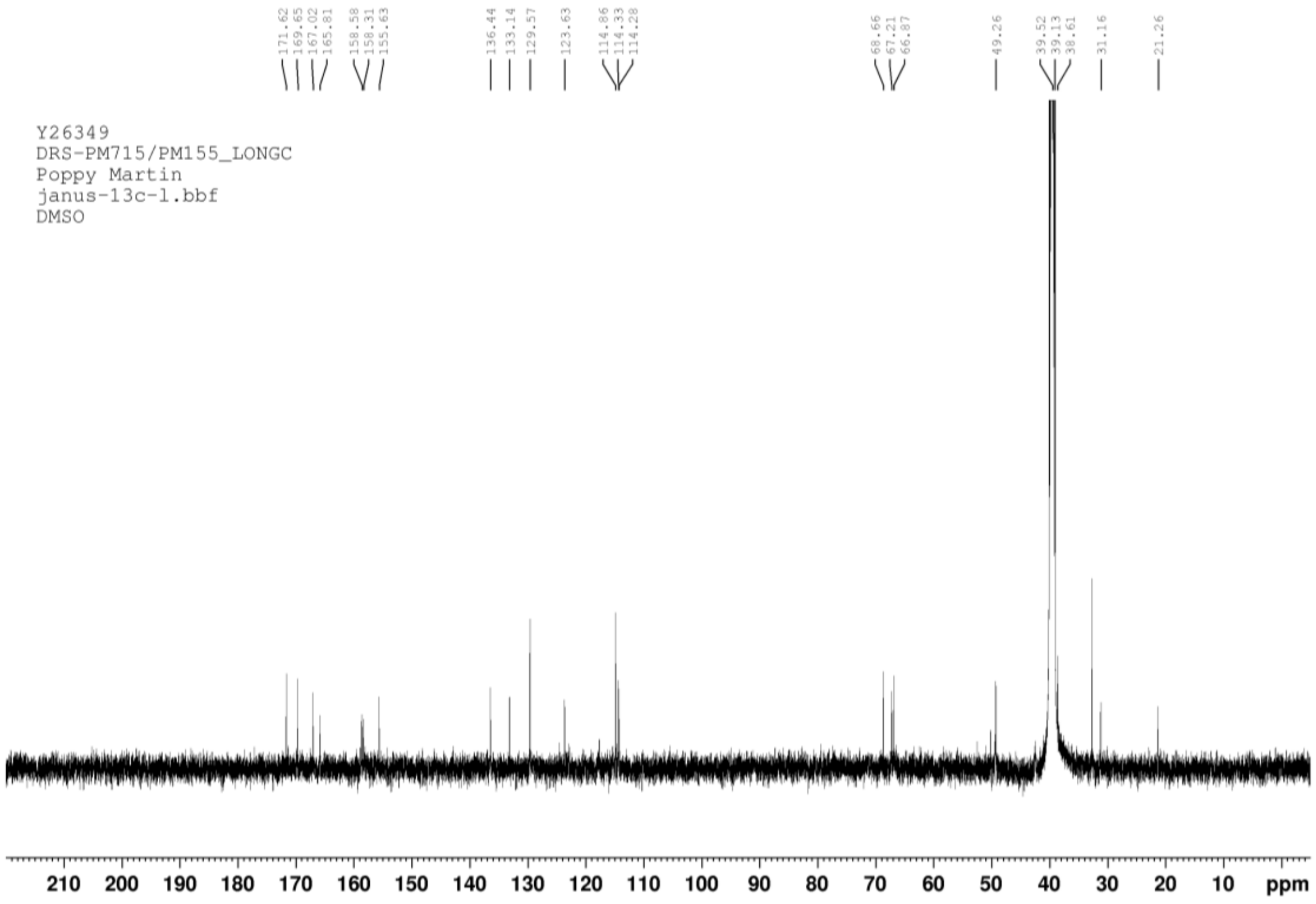
171.62 169.65 167.02 165.81 158.58 158.31 155.63
136.44 133.14 129.57 123.63 114.86 114.33 114.28
68.66 67.21 66.87
49.26
39.52 39.13 38.61
31.16
21.26
Y26349
DRS-PM715/PM155_LONGC
Poppy Martin
janus-13c-l.bbf
DMSO
210 200 190 180 170 160 150 140 130 120 110 100 90 80 70 60 50 40 30 20 10 ppm

(9j)

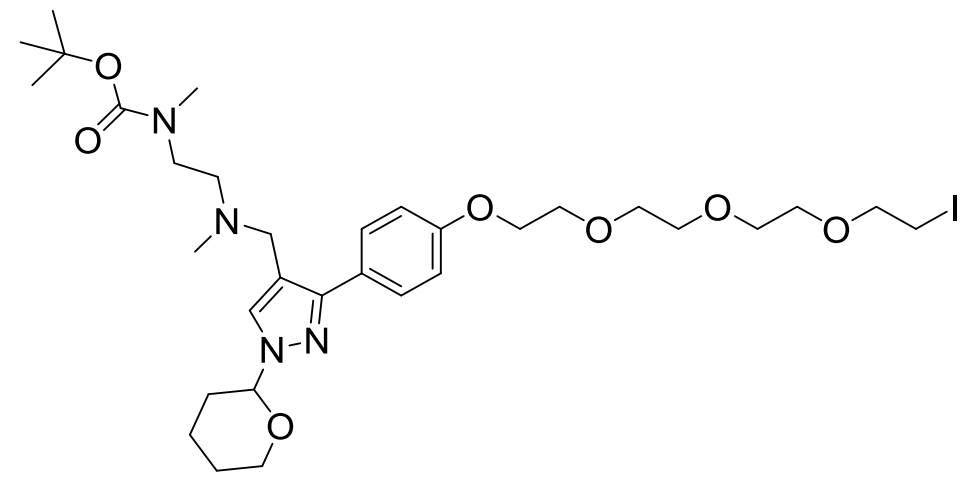

O
O
N
N
O
O
O
O
I
N
N
O

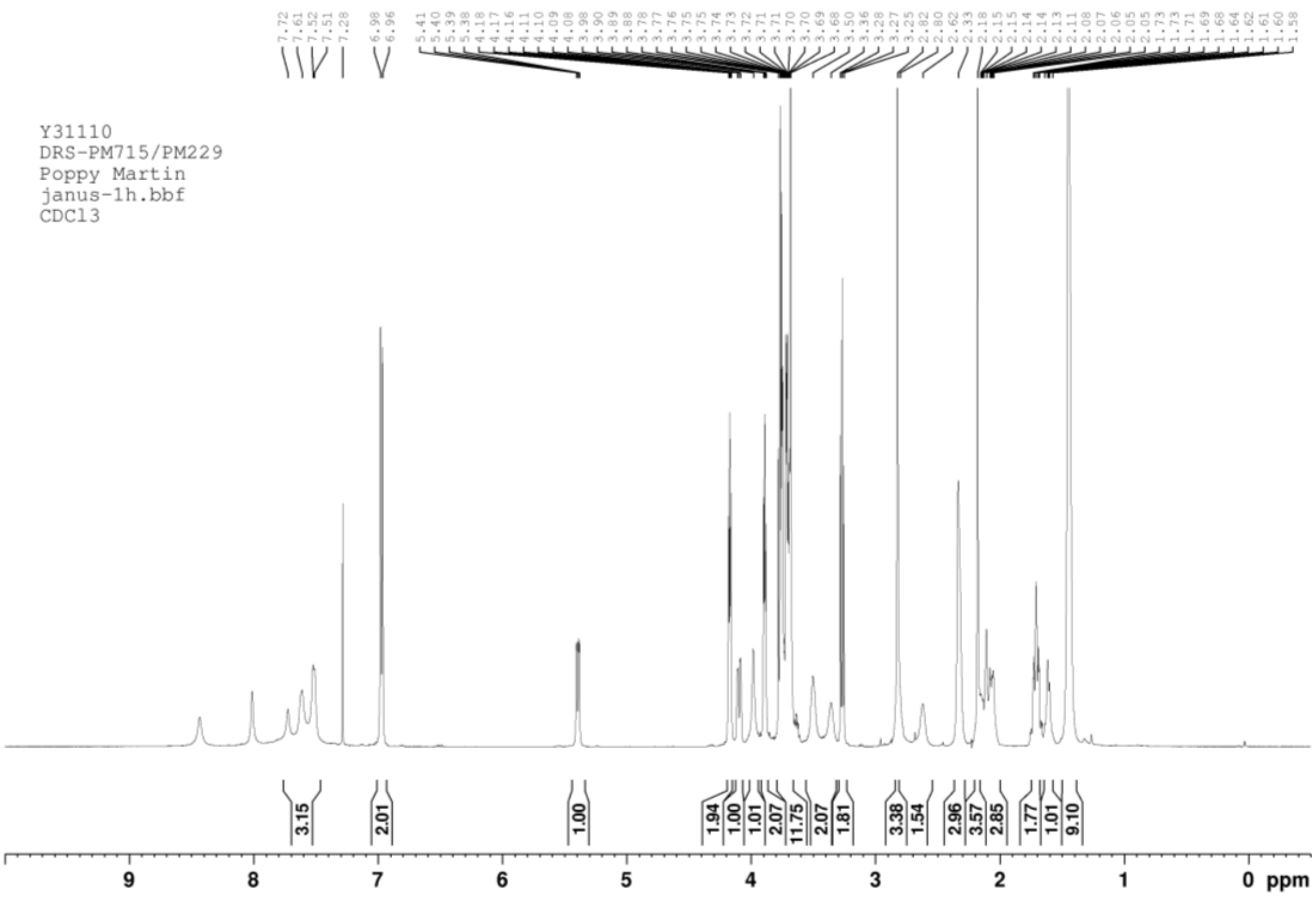

Y31110
DRS-PM715/PM229
Poppy Martin
janus-1h.bbf
CDCl3
3.15
2.01
1.00
1.94
1.00
1.01
2.07
11.75
2.07
1.81
3.38
1.54
2.96
3.57
2.85
1.77
1.01
9.10
9 8 7 6 5 4 3 2 1 0 ppm

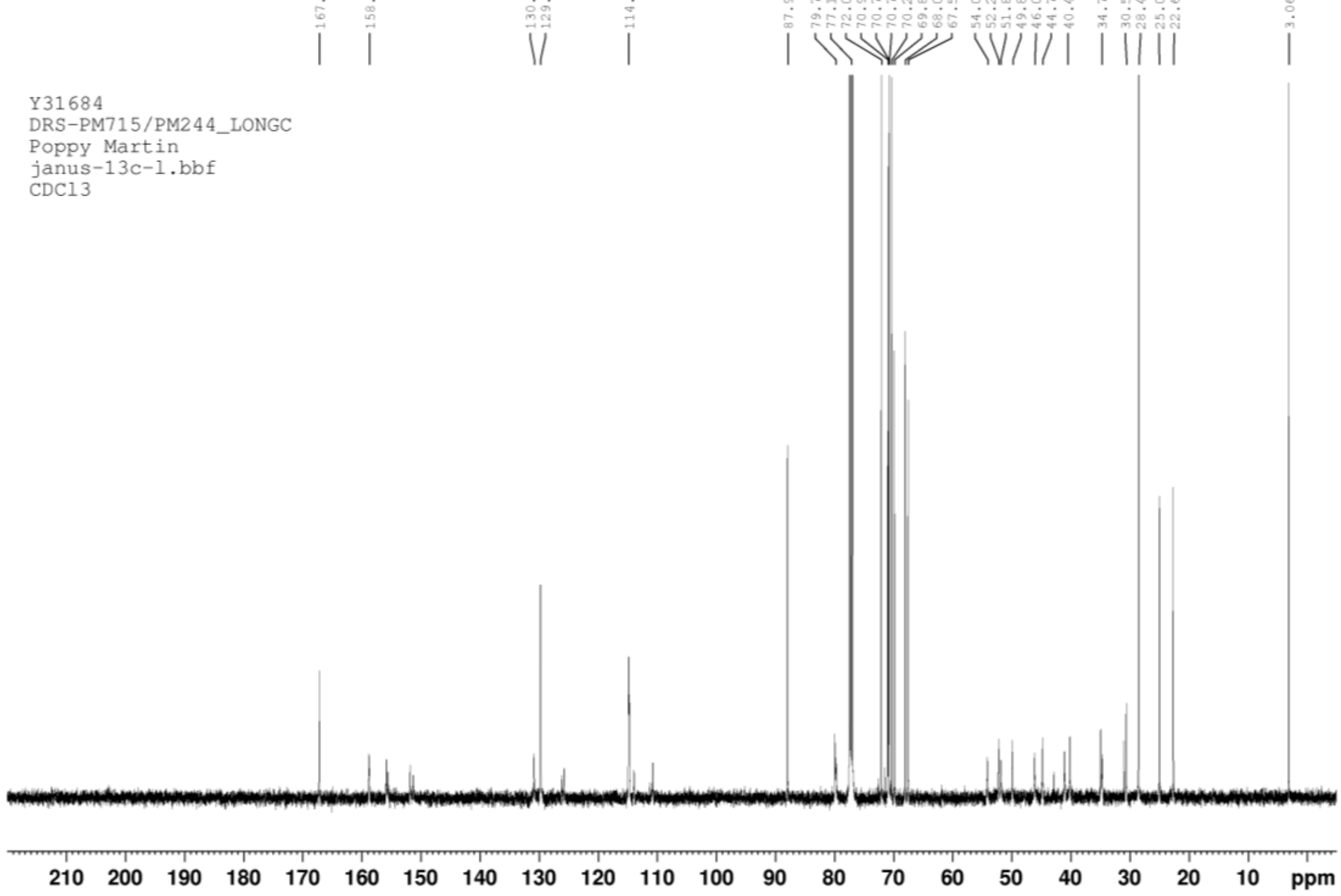

167.18
158.69
130.81
129.73
114.82
87.91
79.78
77.16
72.02
70.92
70.78
70.71
70.28
69.80
68.02
67.50
54.07
52.20
51.82
49.83
46.05
44.74
40.49
34.76
30.59
28.46
25.00
22.60
3.06
Y31684
DRS-PM715/PM244_LONGC
Poppy Martin
janus-13c-l.bbf
CDCl3
210 200 190 180 170 160 150 140 130 120 110 100 90 80 70 60 50 40 30 20 10 ppm

(9k)

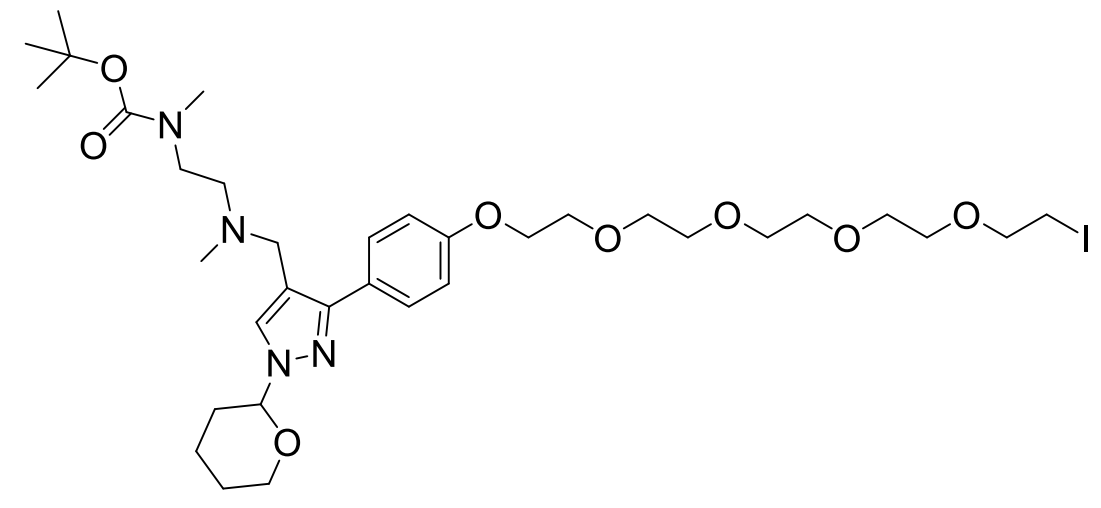

O
O
N
N
O
O
O
O
O
I
N
N
O

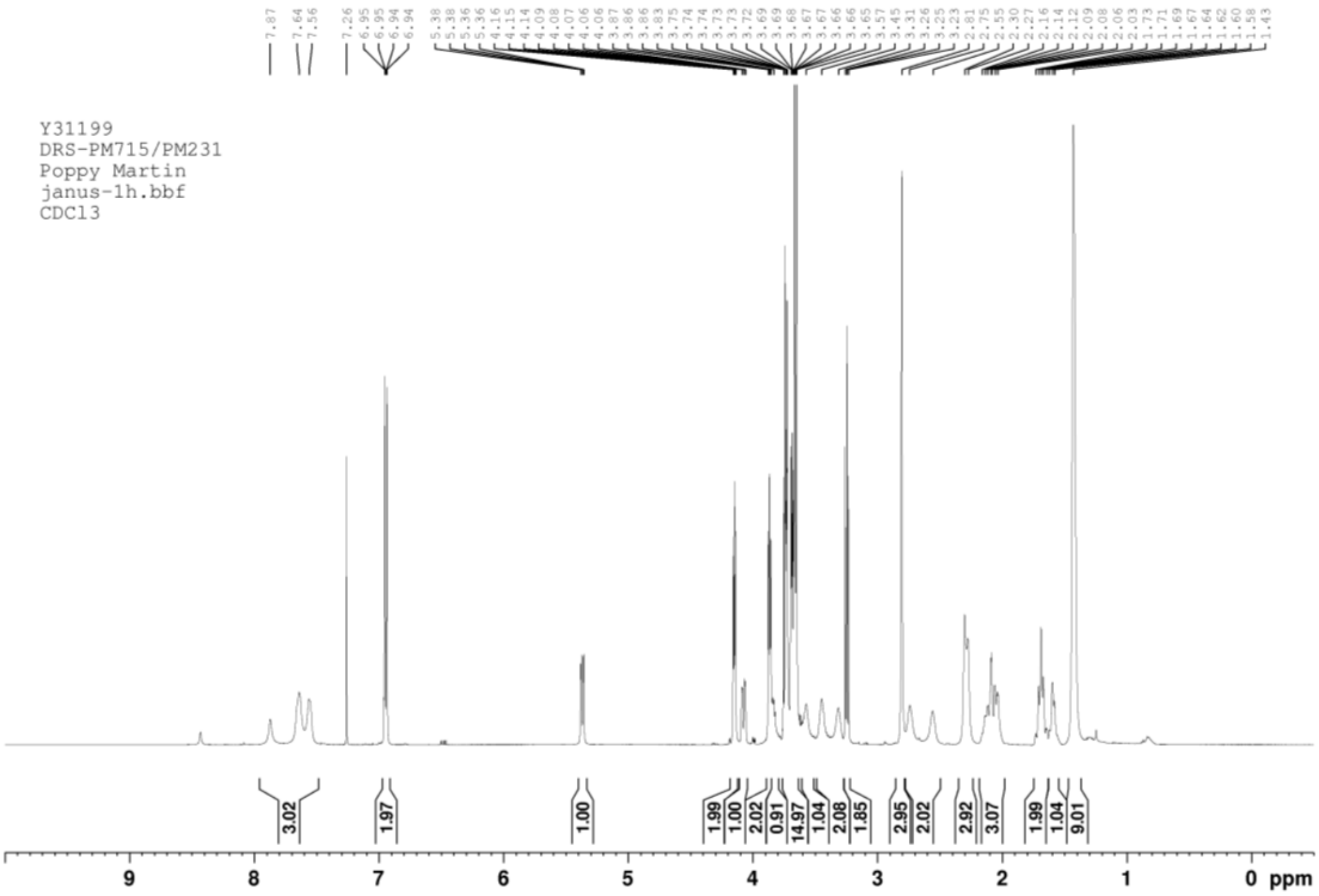

7.87
7.64
7.56
7.26
6.95
6.95
6.94
6.94
5.38
5.38
5.36
5.36
4.16
4.15
4.14
4.09
4.08
4.07
4.06
4.06
3.87
3.86
3.85
3.83
3.75
3.74
3.74
3.73
3.73
3.73
3.72
3.69
3.69
3.68
3.67
3.67
3.66
3.66
3.65
3.57
3.45
3.31
3.26
3.25
3.23
2.81
2.75
2.55
2.30
2.27
2.16
2.14
2.12
2.09
2.08
2.06
2.03
1.73
1.71
1.69
1.67
1.64
1.62
1.60
1.58
1.43
Y31199
DRS-PM715/PM231
Poppy Martin
janus-1h.bbf
CDCl3
3.02
1.97
1.00
1.99
1.00
2.02
0.91
14.97
1.04
2.08
1.85
2.95
2.02
2.92
3.07
1.99
1.04
9.01
9
8
7
6
5
4
3
2
1
0 ppm

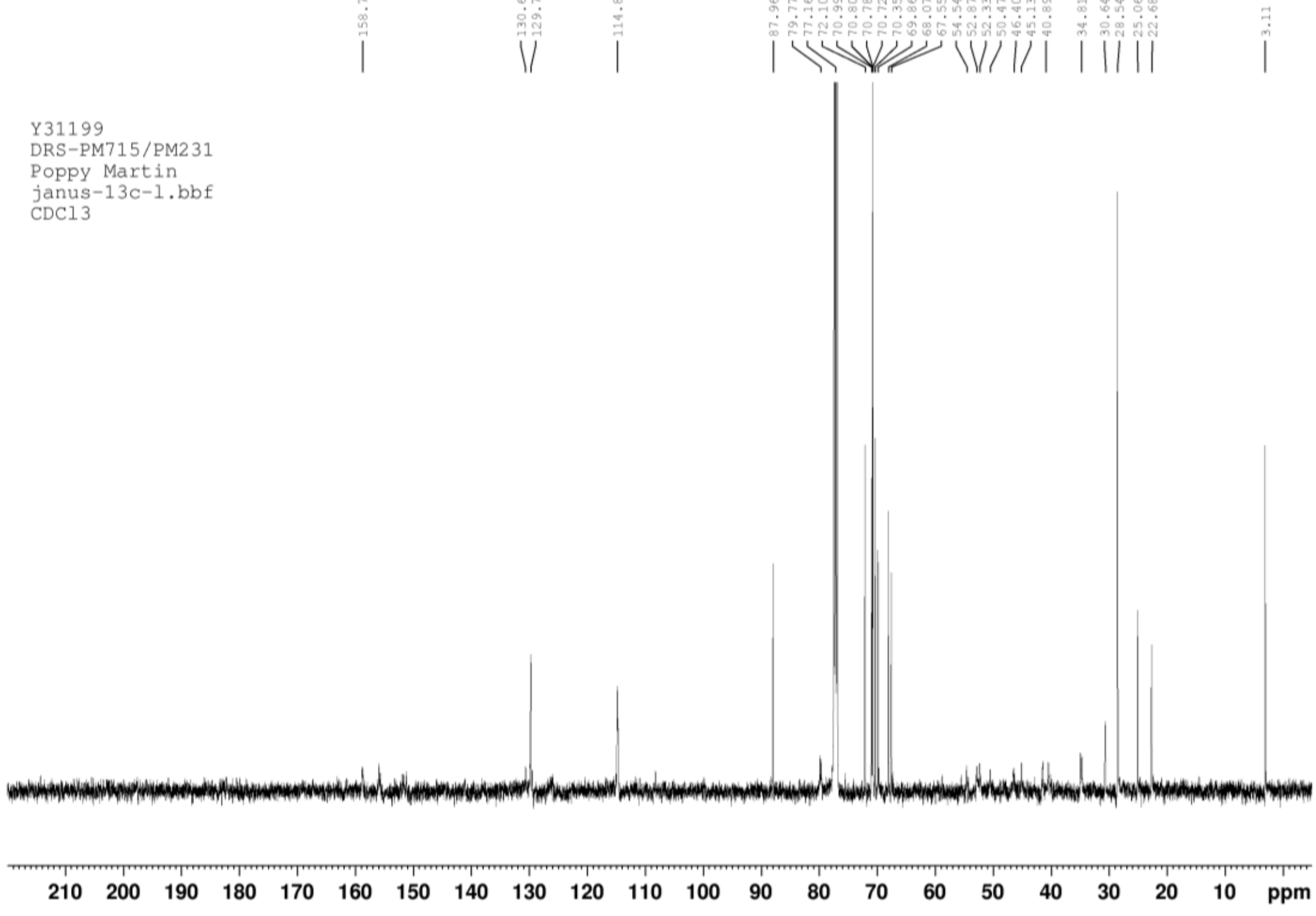

158.75
130.65
129.74
114.83
87.96
79.77
77.16
72.10
70.99
70.80
70.78
70.72
70.35
69.86
68.07
67.55
54.54
52.87
52.13
50.47
46.40
45.13
40.89
34.81
30.64
28.54
25.06
22.68
3.11
Y31199
DRS-PM715/PM231
Poppy Martin
janus-13c-1.bbf
CDCl3
210
200
190
180
170
160
150
140
130
120
110
100
90
80
70
60
50
40
30
20
10
ppm

(9l)

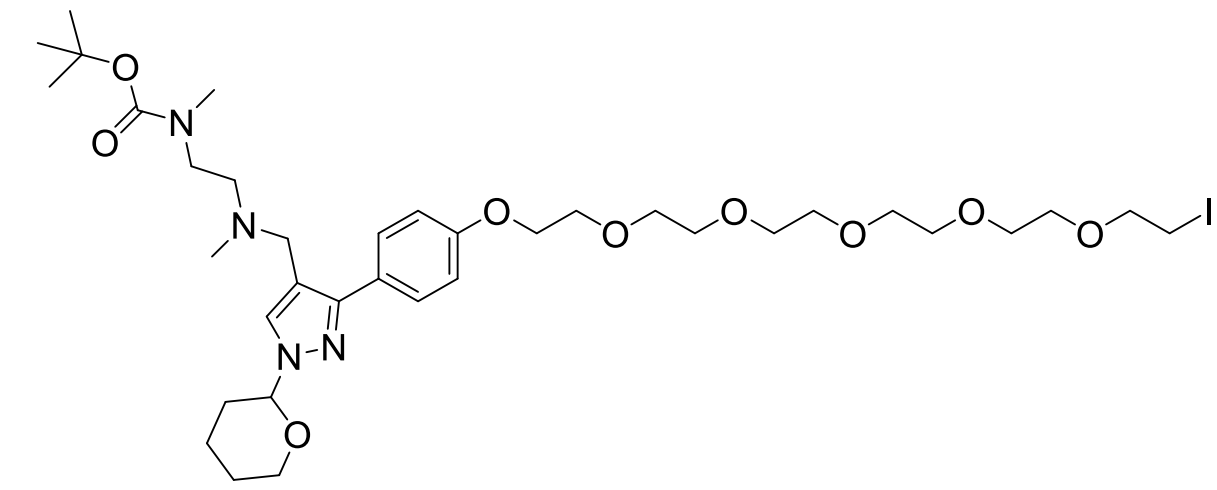

O
N
N
N
N
O
O
O
O
O
O
O
I

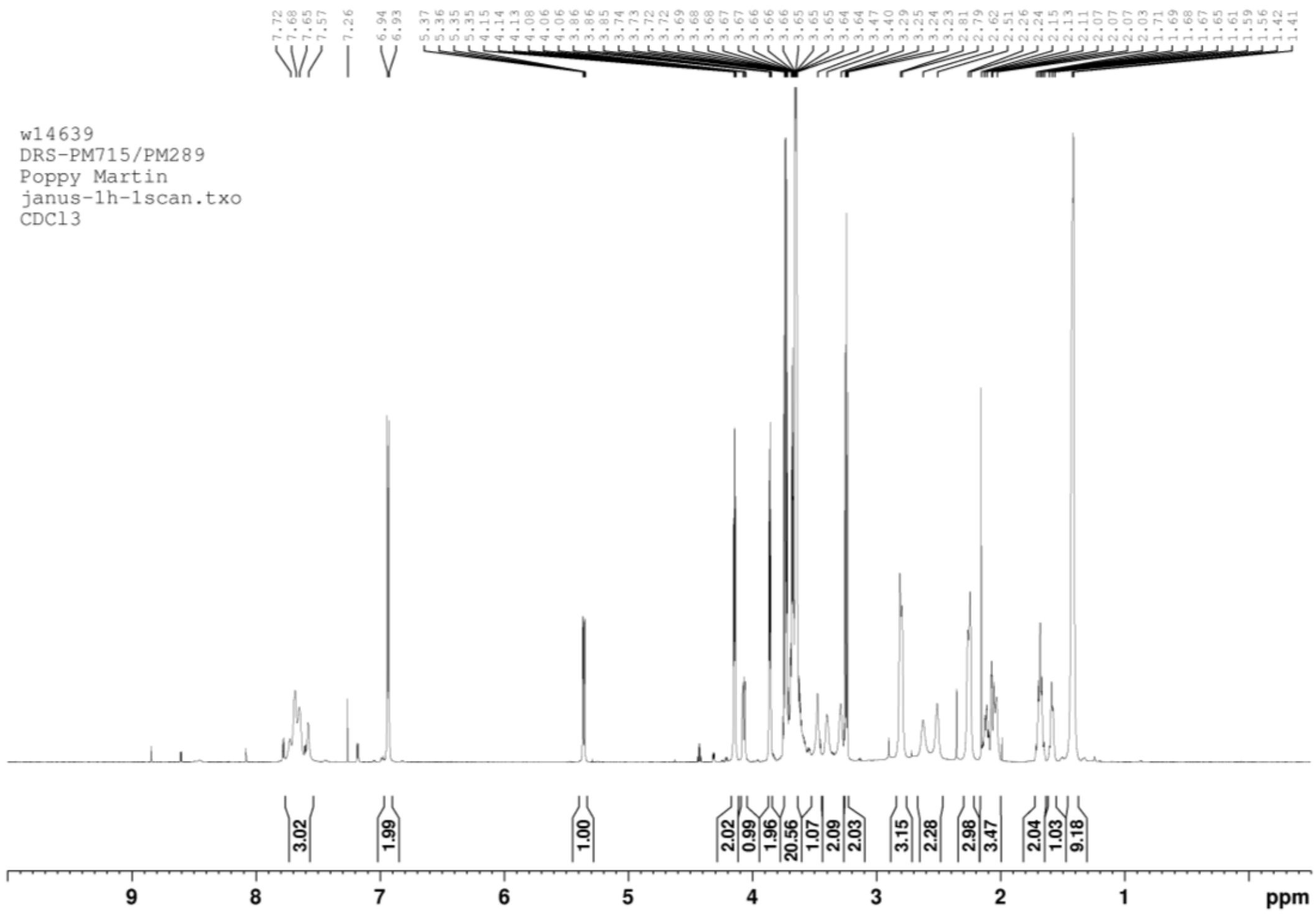

w14639
DRS-PM715/PM289
Poppy Martin
janus-1h-1scan.txo
CDCl3
3.02
1.99
1.00
2.02
0.99
1.96
20.56
1.07
2.09
2.03
3.15
2.28
2.98
3.47
2.04
1.03
9.18
ppm

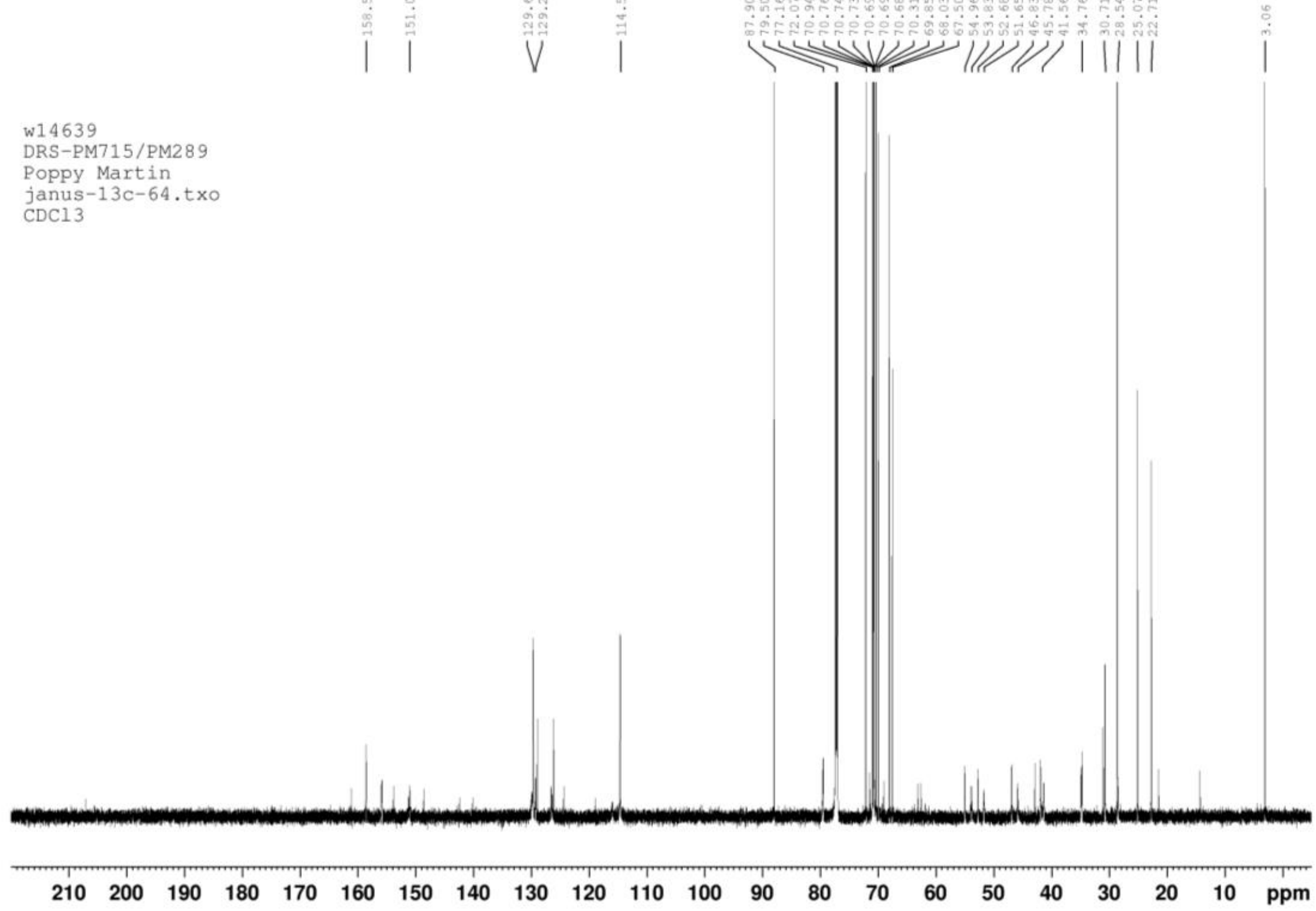

w14639
DRS-PM715/PM289
Poppy Martin
janus-13c-64.txo
CDCl3
ppm

(11)

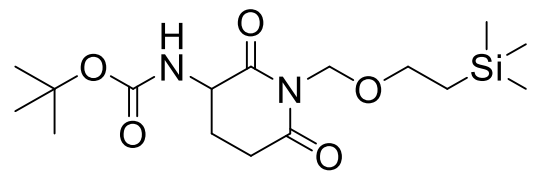
H
N
O
O
O
N
O
Si
O

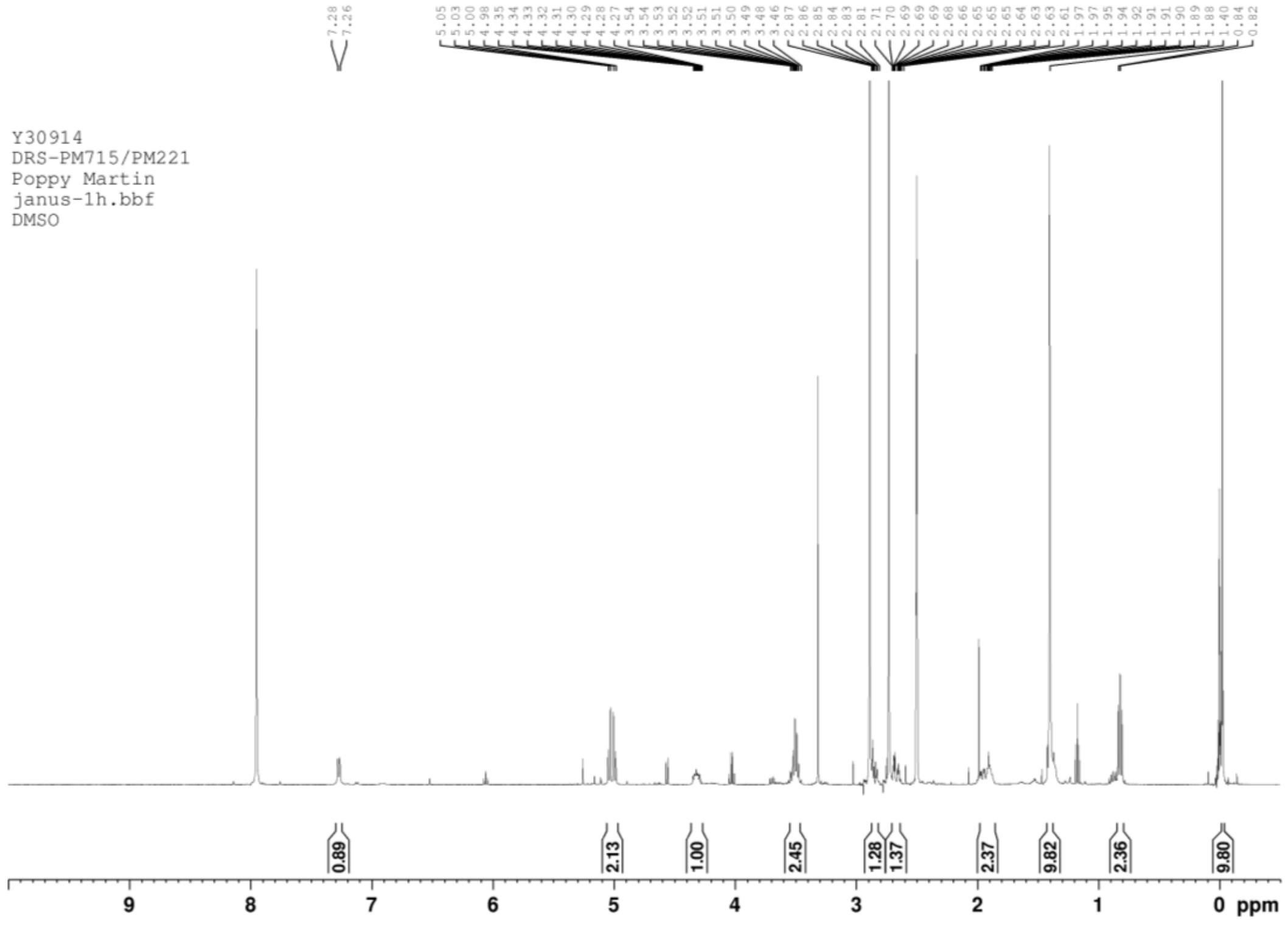
7.28
7.26
5.05
5.03
5.00
4.98
4.35
4.34
4.33
4.32
4.31
4.30
4.29
4.28
4.27
3.54
3.53
3.52
3.52
3.51
3.51
3.50
3.49
3.48
3.46
2.87
2.86
2.85
2.84
2.83
2.81
2.71
2.70
2.69
2.69
2.69
2.68
2.66
2.65
2.65
2.65
2.64
2.63
2.63
2.61
1.97
1.95
1.94
1.92
1.91
1.91
1.90
1.89
1.88
1.40
0.84
0.82
Y30914
DRS-PM715/PM221
Poppy Martin
janus-1h.bbf
DMSO
0.89
2.13
1.00
2.45
1.28
1.37
2.37
9.82
2.36
9.80
9
8
7
6
5
4
3
2
1
0 ppm

(12)

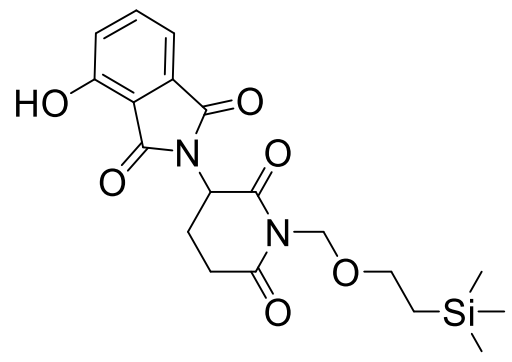
HO
O
N
O
N
O
O
O
Si

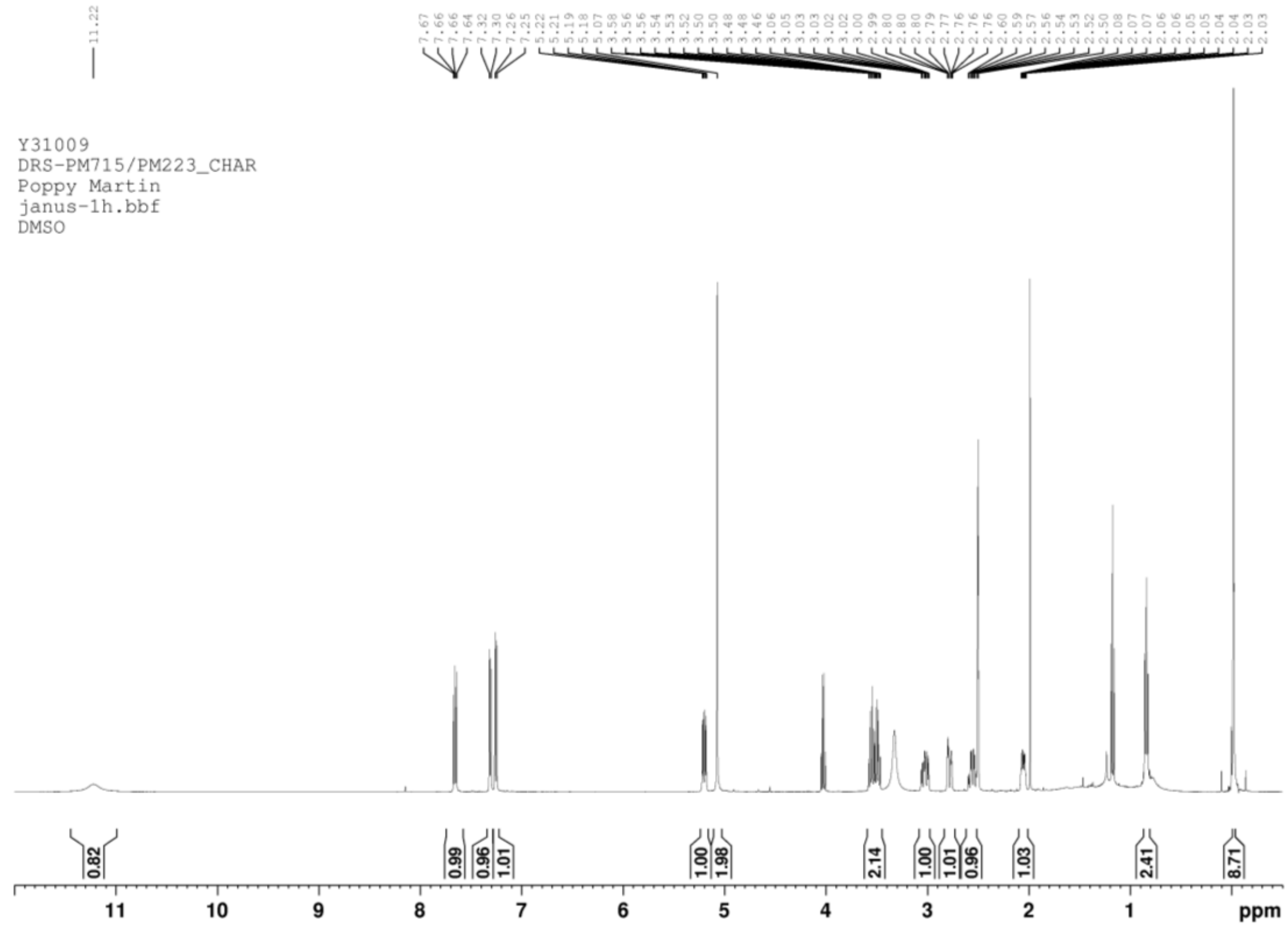
Y31009
DRS-PM715/PM223_CHAR
Poppy Martin
janus-1h.bbf
DMSO
0.82
0.99
0.96
1.01
1.00
1.98
2.14
1.00
1.01
0.96
1.03
2.41
8.71
11 10 9 8 7 6 5 4 3 2 1 ppm

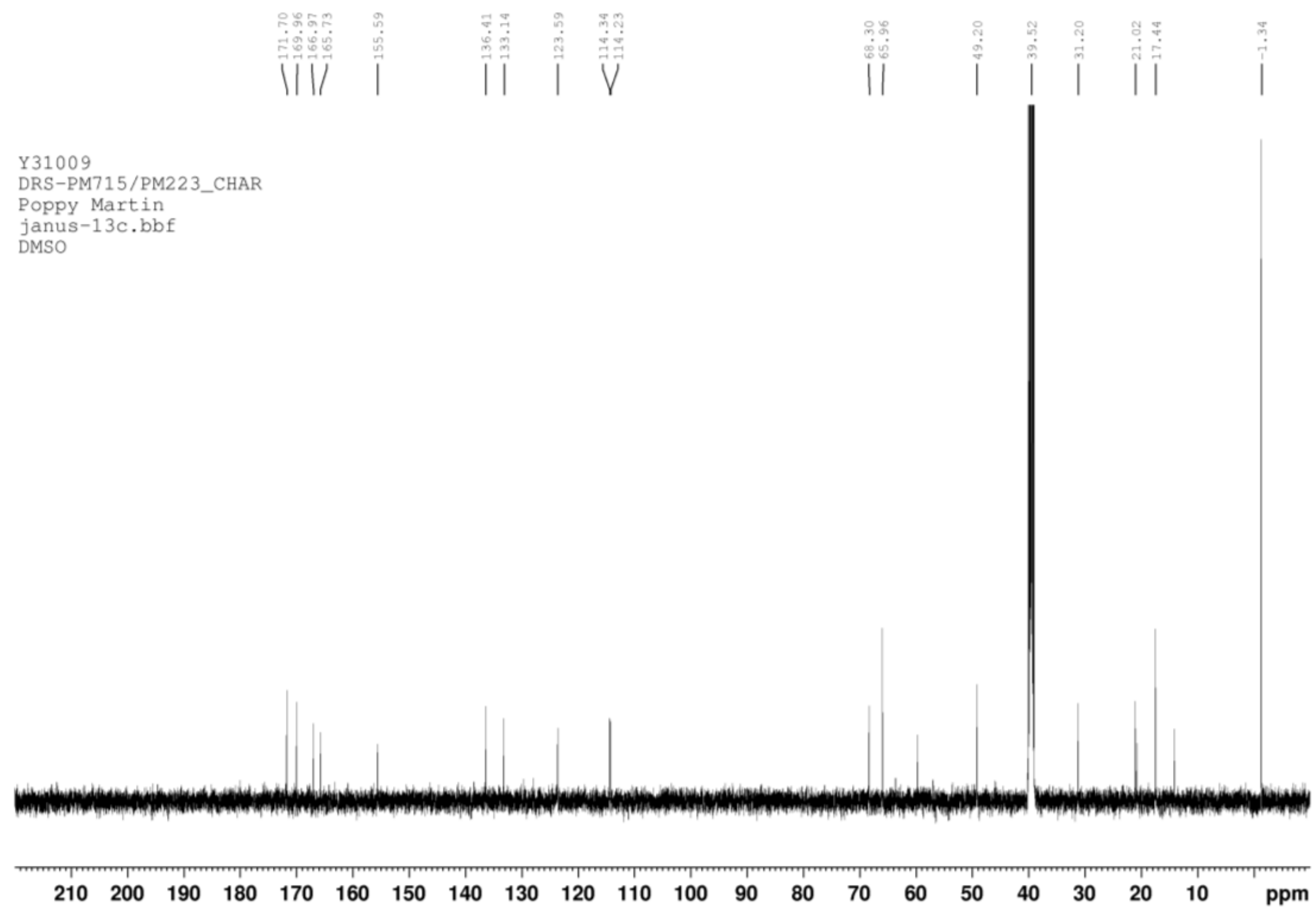
171.70
169.96
166.97
165.73
155.59
136.41
133.14
123.59
114.34
114.23
68.30
65.96
49.20
39.52
31.20
21.02
17.44
-1.34
Y31009
DRS-PM715/PM223_CHAR
Poppy Martin
janus-13c.bbf
DMSO
210 200 190 180 170 160 150 140 130 120 110 100 90 80 70 60 50 40 30 20 10 ppm

PROTAC L

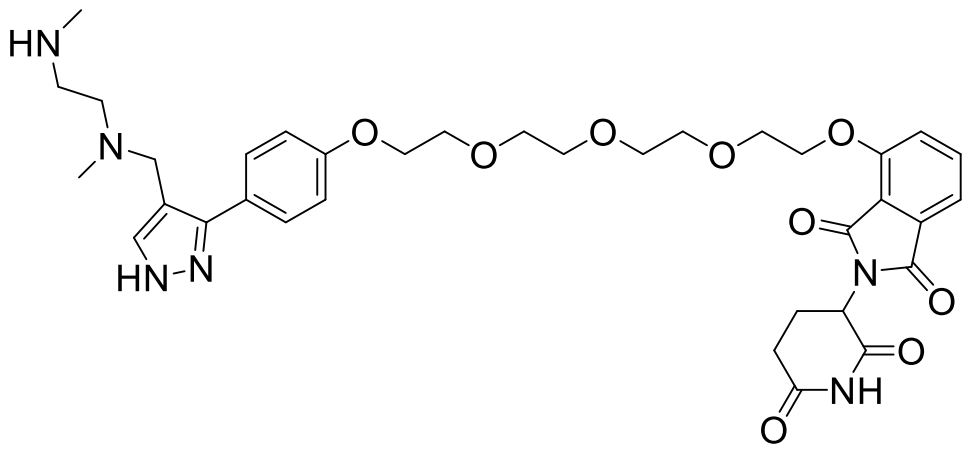

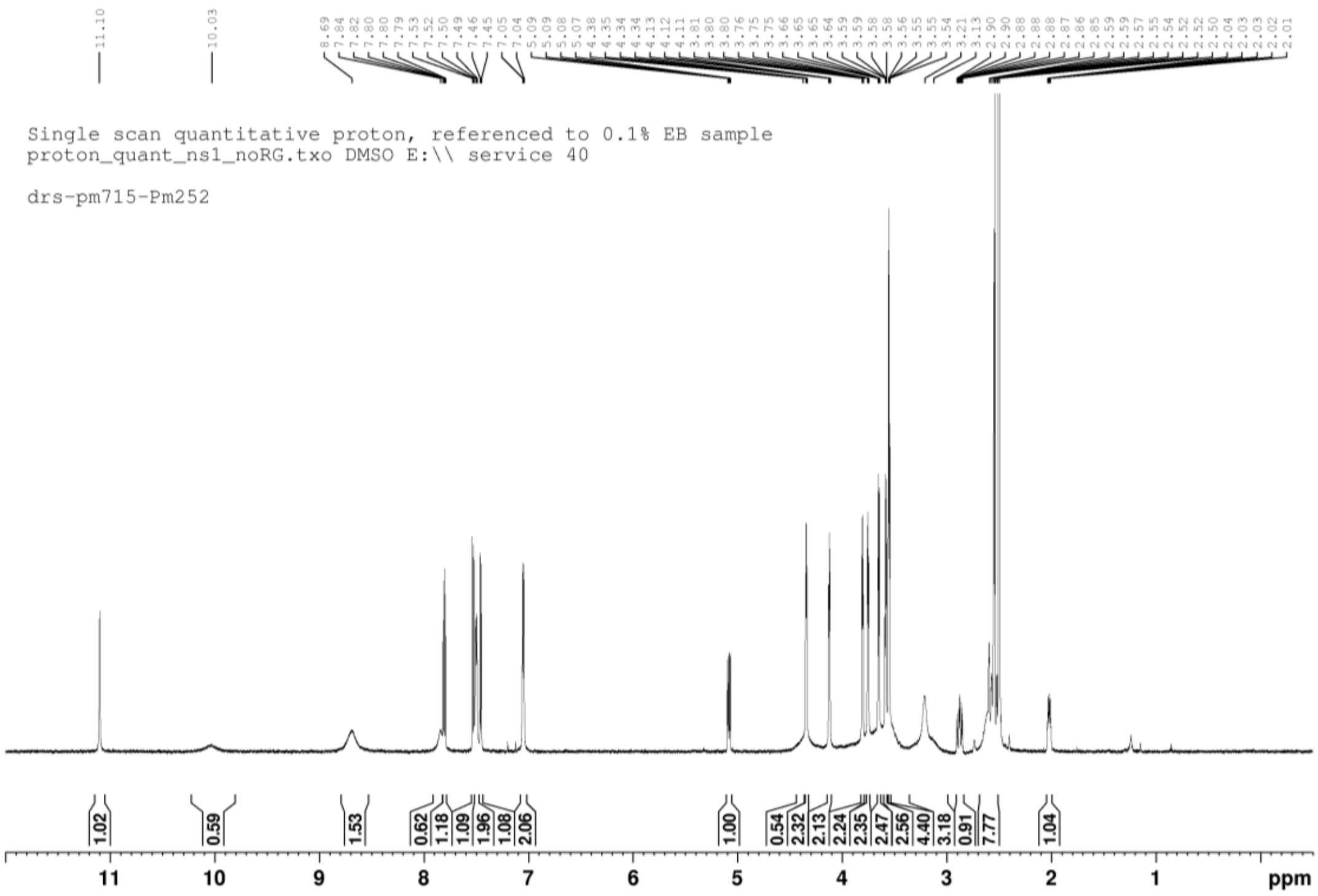
Single scan quantitative proton, referenced to 0.1% EB sample
proton_quant_ns1_noRG.txo DMSO E:\\ service 40
drs-pm715-Pm252
ppm

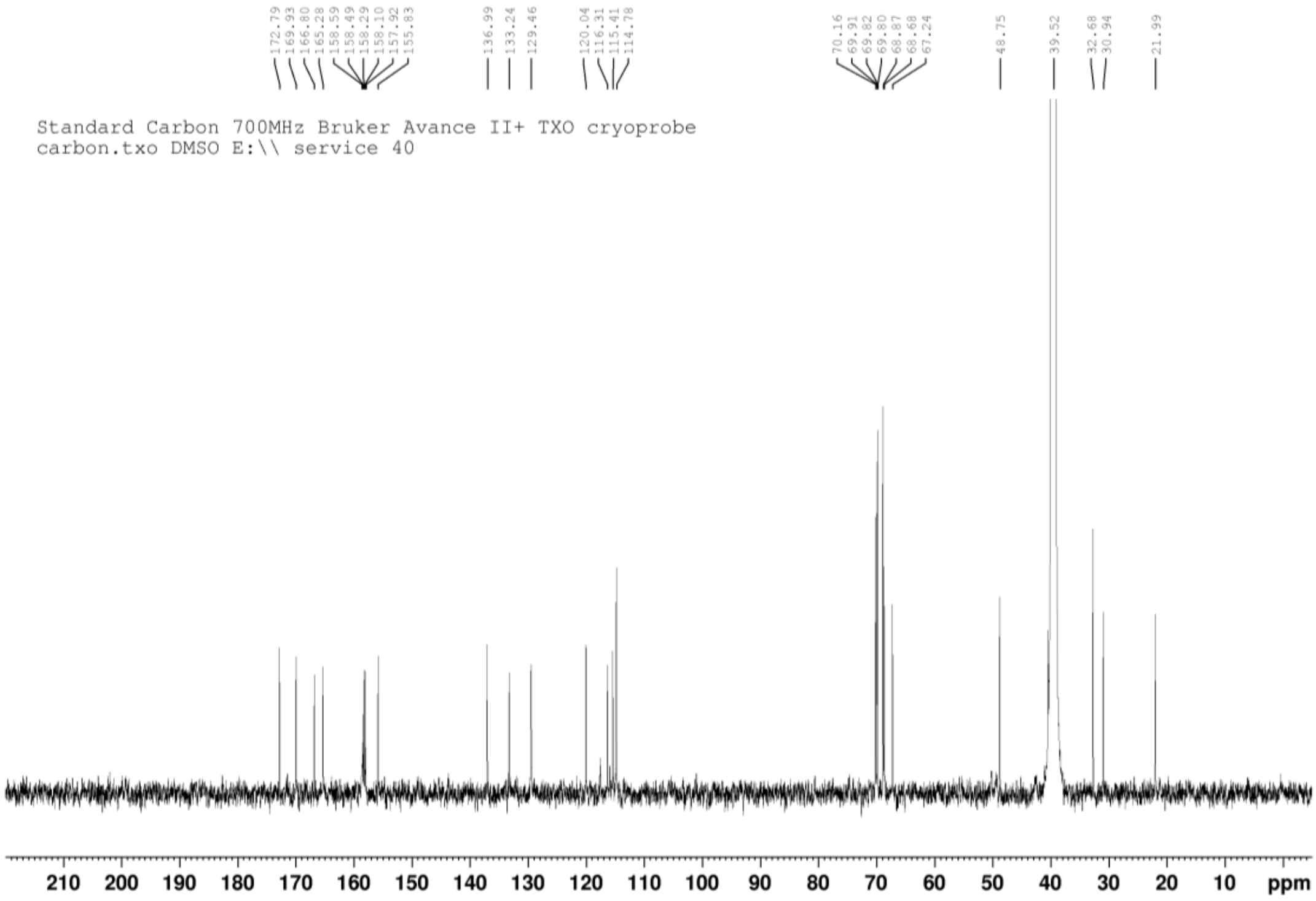
Standard Carbon 700MHz Bruker Avance II+ TXO cryoprobe
carbon.txo DMSO E:\\ service 40
ppm

PROTAC M

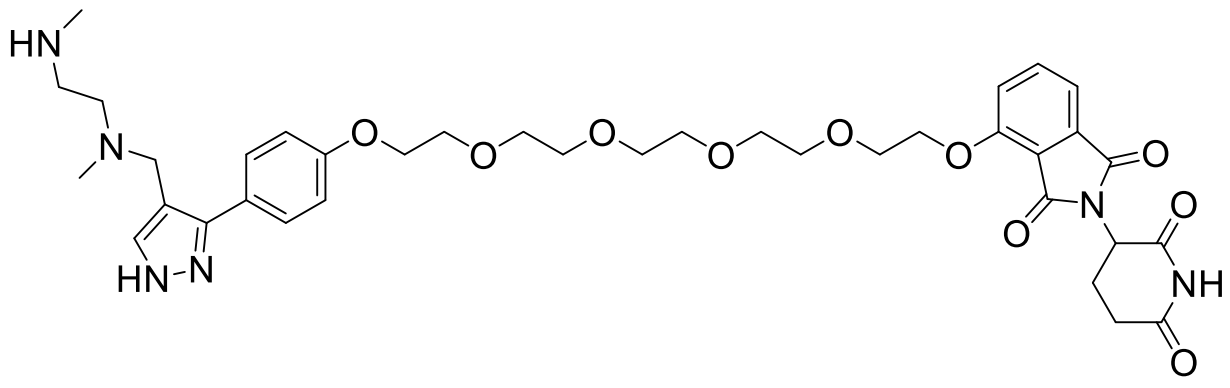
HN
N
O
HN-N
NH

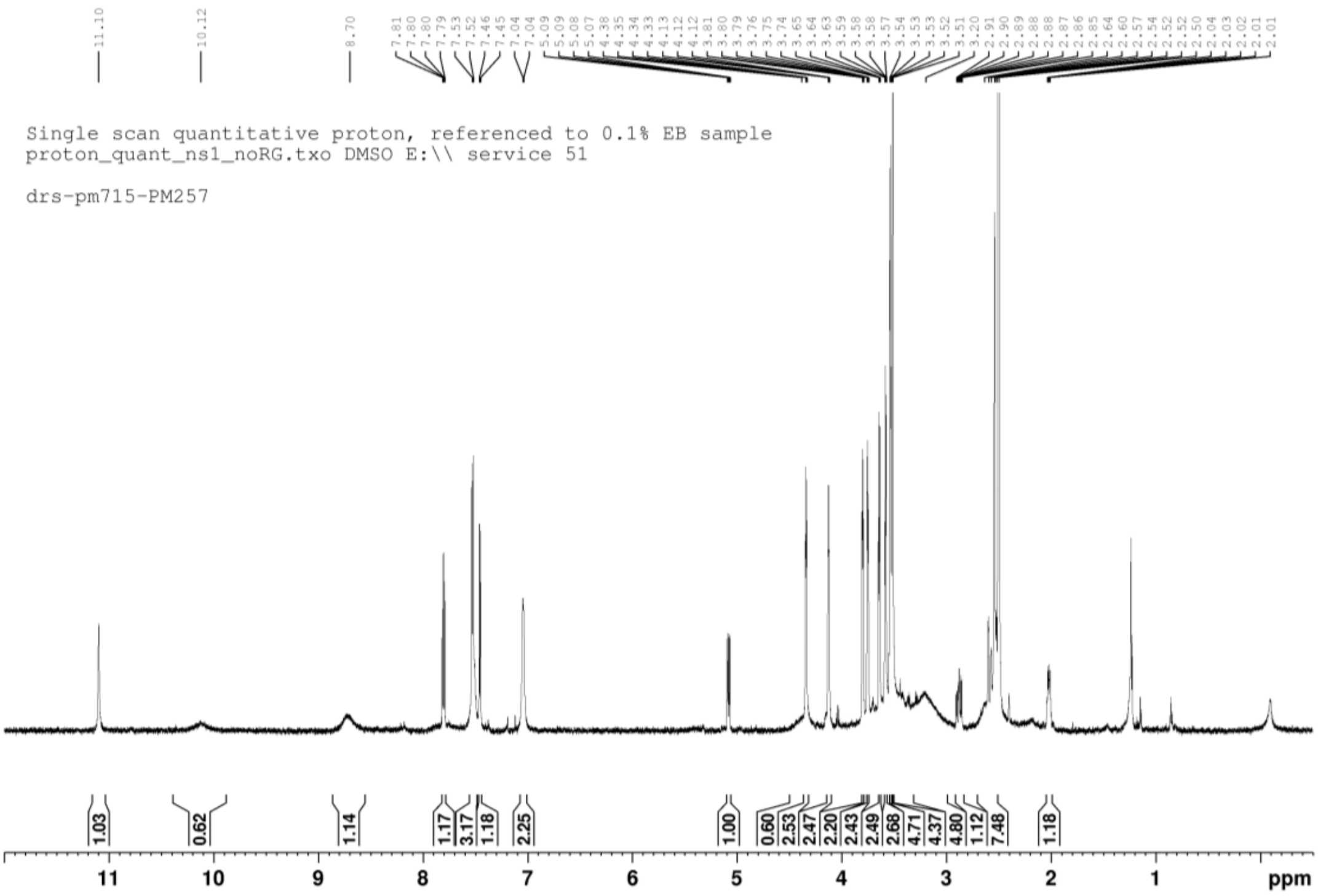
Single scan quantitative proton, referenced to 0.1% EB sample
proton_quant_ns1_noRG.txo DMSO E:\\ service 51
drs-pm715-PM257
ppm

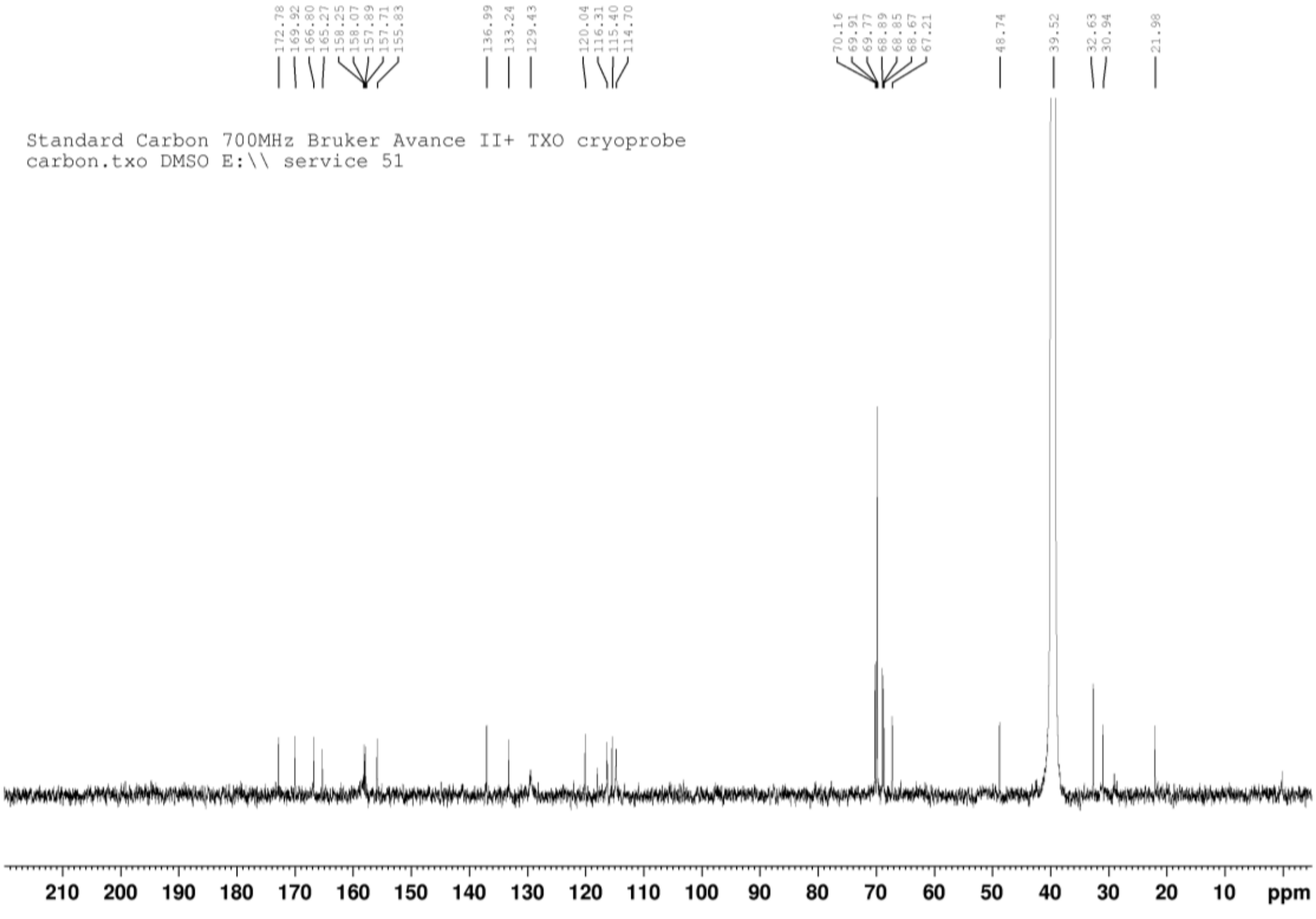
Standard Carbon 700MHz Bruker Avance II+ TXO cryoprobe
carbon.txo DMSO E:\\ service 51
ppm

PROTAC N

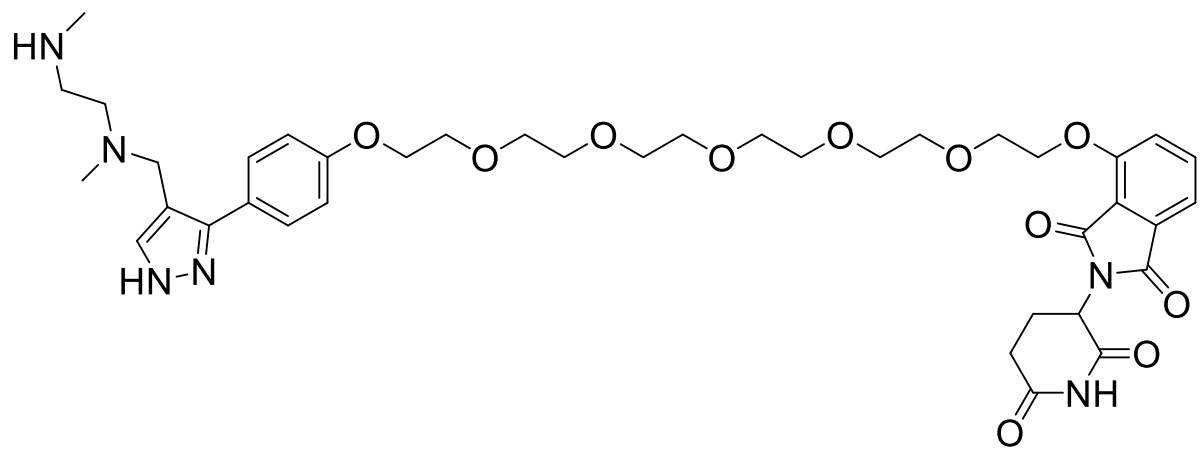

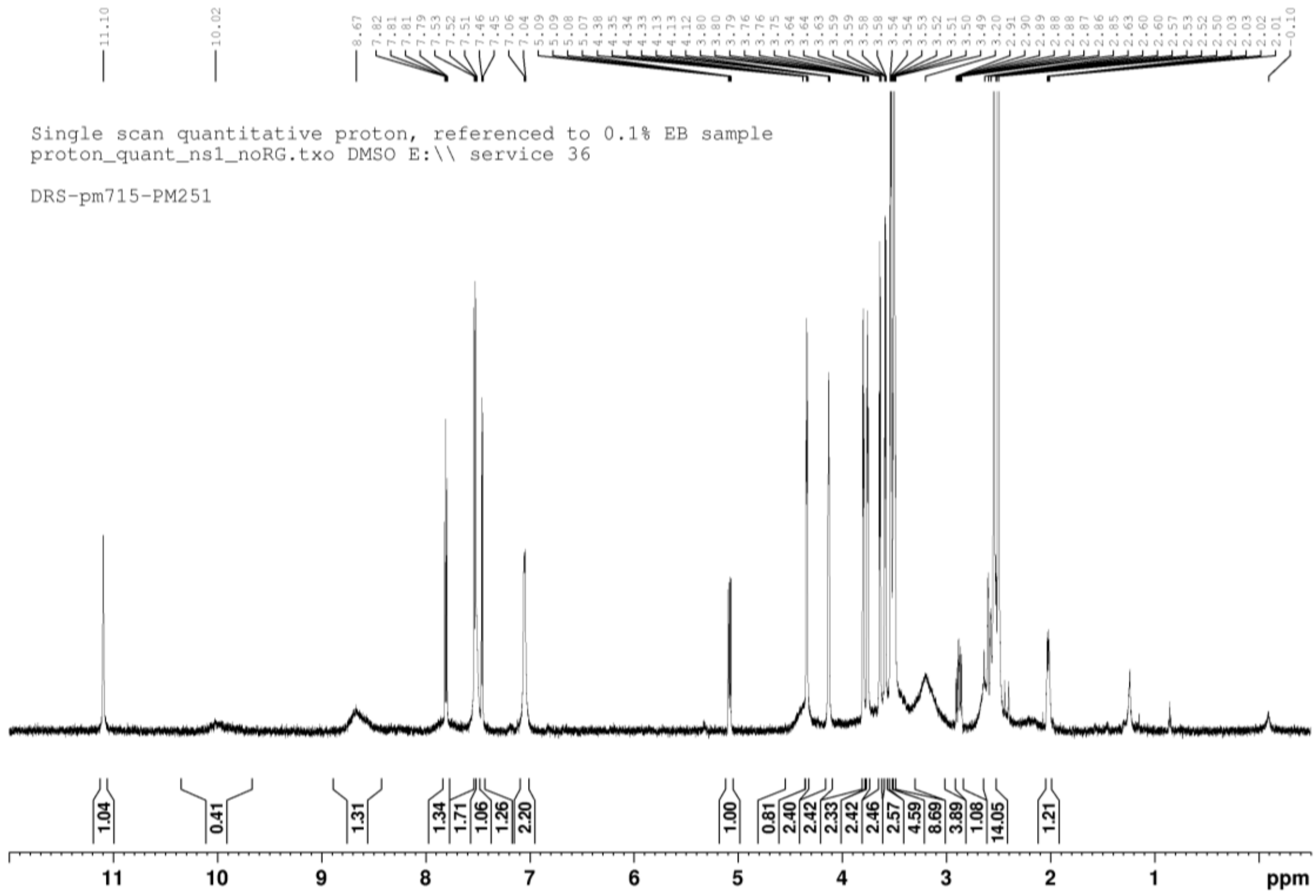
Single scan quantitative proton, referenced to 0.1% EB sample
proton_quant_ns1_noRG.txo DMSO E:\\ service 36
DRS-pm715-PM251
1.04
0.41
1.31
1.34
1.71
1.06
1.26
2.20
1.00
0.81
2.40
2.42
2.33
2.42
2.46
2.57
4.59
8.69
3.89
1.08
14.05
1.21
ppm

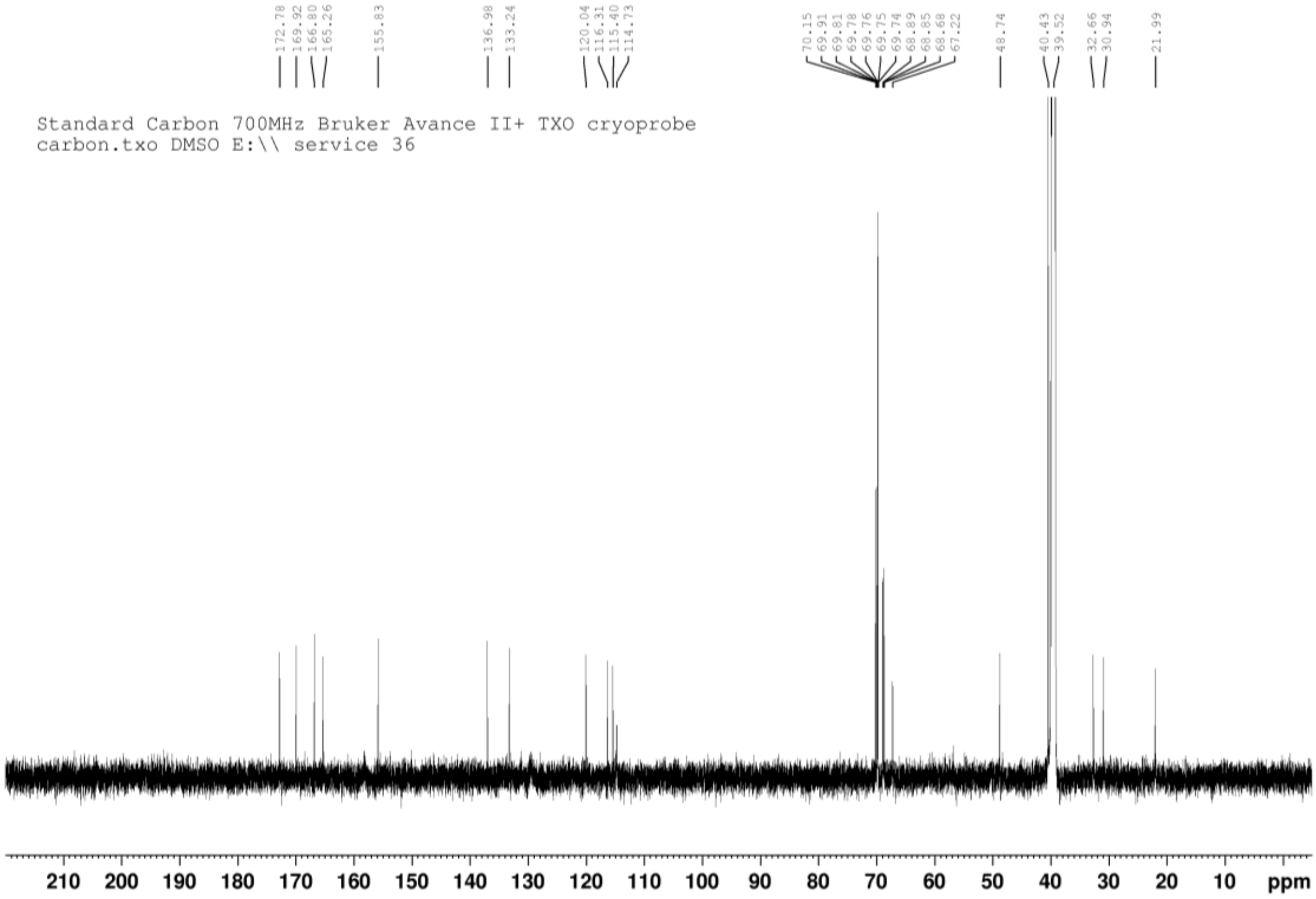
172.78
169.92
166.80
165.26
155.83
136.98
133.24
120.04
116.31
115.40
114.73
70.15
69.91
69.81
69.78
69.76
69.75
69.74
68.89
68.85
68.68
67.22
48.74
40.43
39.52
32.66
30.94
21.99
Standard Carbon 700MHz Bruker Avance II+ TXO cryoprobe
carbon.txo DMSO E:\\ service 36
ppm

(14)

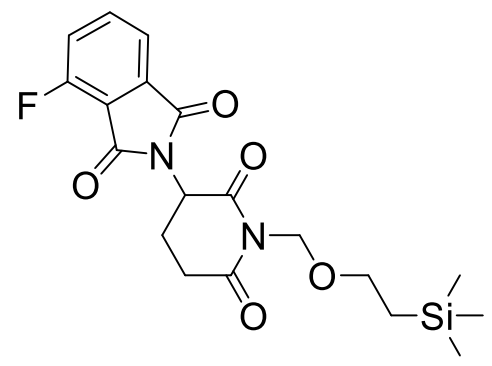
F
O
N
O
O
N
O
O
Si

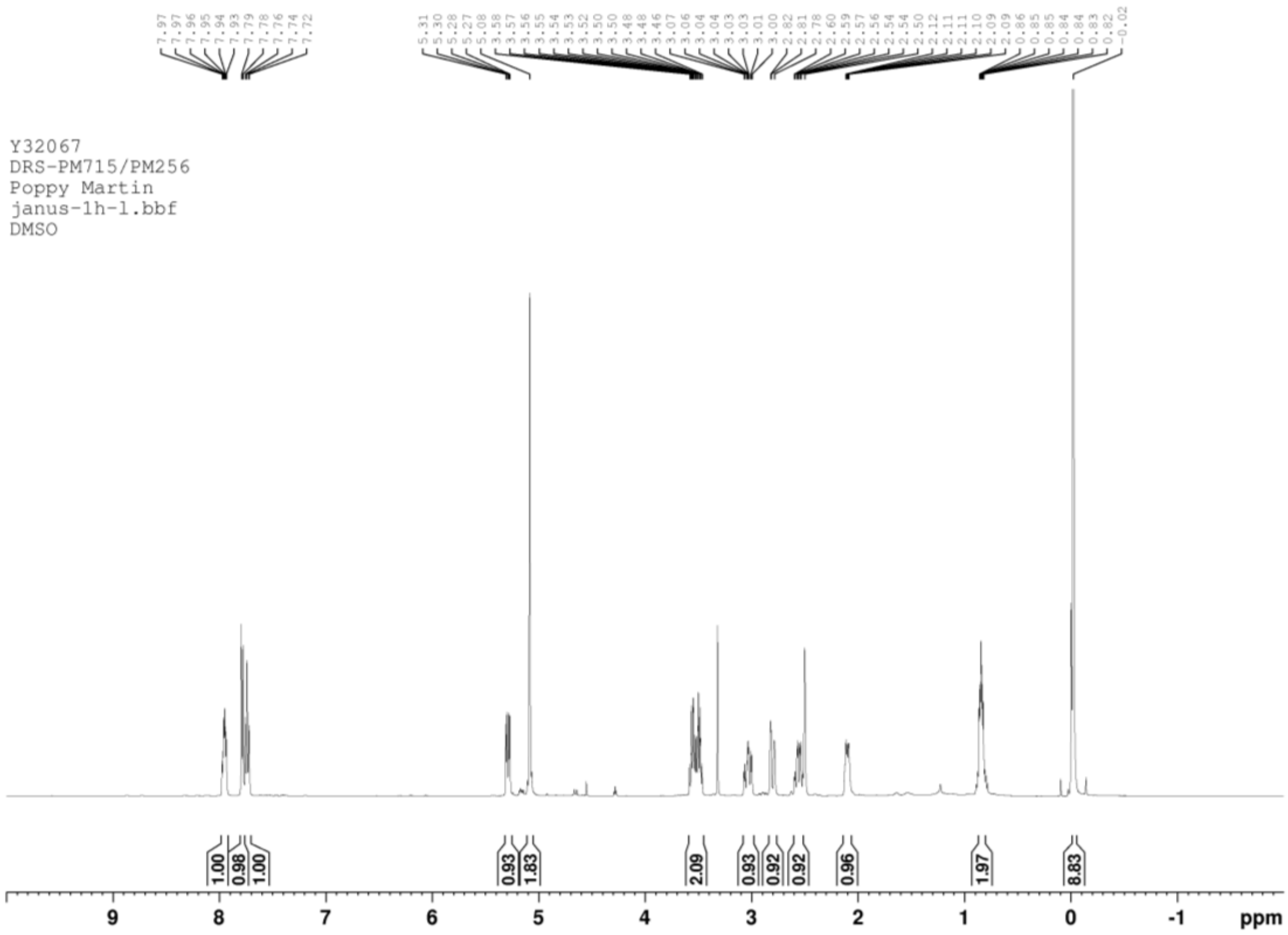
Y32067
DRS-PM715/PM256
Poppy Martin
janus-1h-1.bbf
DMSO
1.00
0.98
1.00
0.93
1.83
2.09
0.93
0.92
0.92
0.96
1.97
8.83
9
8
7
6
5
4
3
2
1
0
-1
ppm

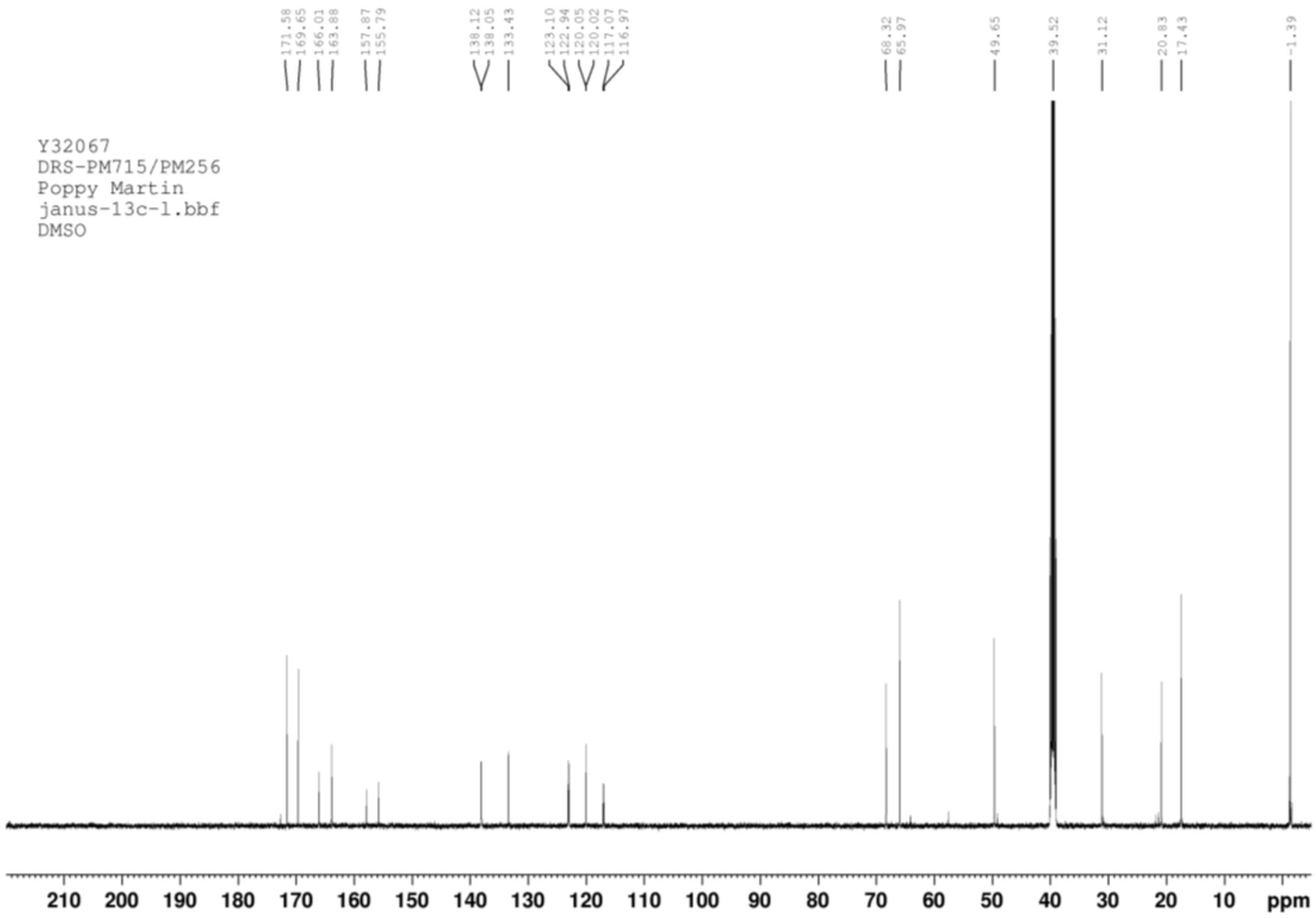
171.58
169.65
166.01
163.88
157.87
155.79
138.12
138.05
133.43
123.10
122.94
120.05
120.02
117.07
116.97
68.32
65.97
49.65
39.52
31.12
20.83
17.43
-1.39
Y32067
DRS-PM715/PM256
Poppy Martin
janus-13c-1.bbf
DMSO
210
200
190
180
170
160
150
140
130
120
110
100
90
80
70
60
50
40
30
20
10
ppm

(16m)

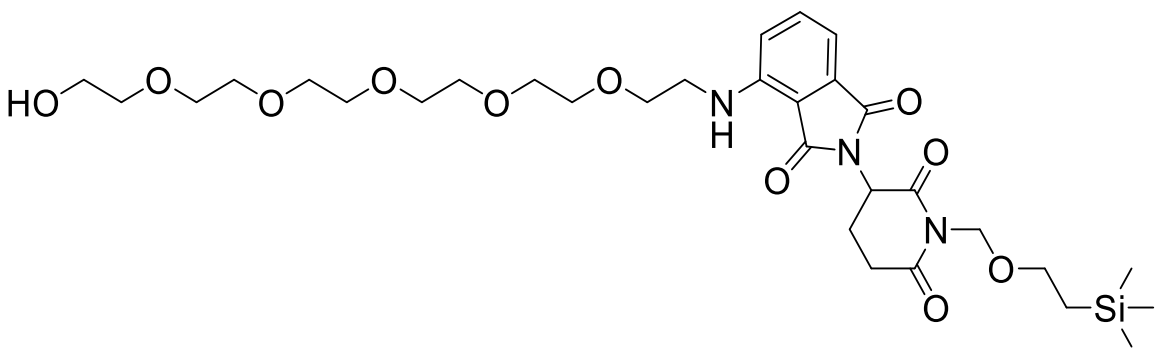
HO
O
O
O
O
O
N
H
O
N
O
O
N
O
O
Si

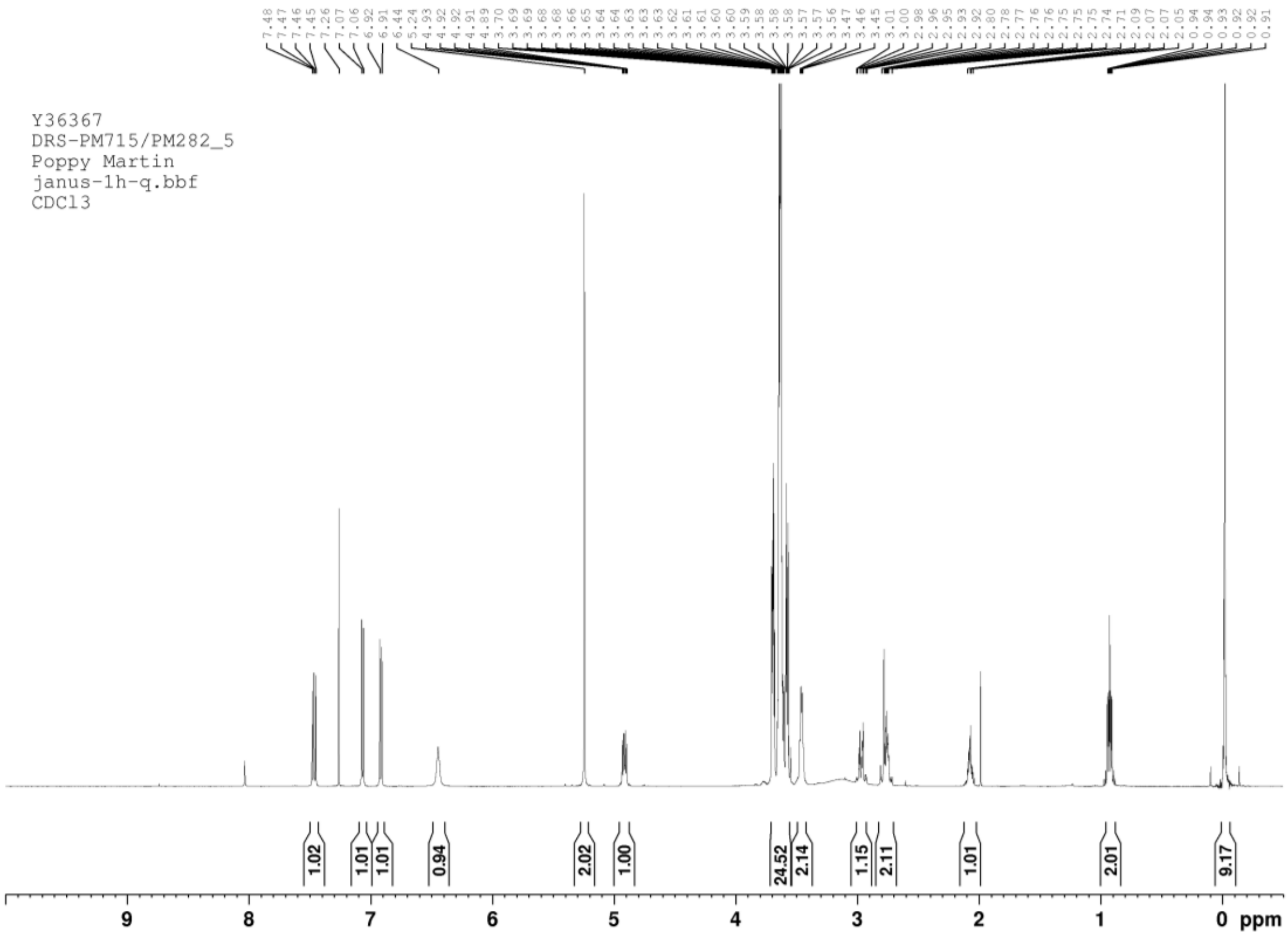
7.48 7.47 7.46 7.45 7.26 7.07 7.06 6.92 6.91 6.44 5.24 4.93 4.92 4.92 4.91 4.91 4.89 3.70 3.69 3.69 3.68 3.68 3.66 3.65 3.64 3.64 3.63 3.63 3.62 3.61 3.61 3.60 3.60 3.59 3.58 3.58 3.58 3.57 3.57 3.56 3.47 3.46 3.45 3.01 3.00 2.98 2.96 2.95 2.93 2.92 2.80 2.78 2.77 2.76 2.76 2.75 2.75 2.74 2.71 2.09 2.07 2.07 2.05 0.94 0.93 0.93 0.92 0.92 0.91
Y36367
DRS-PM715/PM282_5
Poppy Martin
janus-1h-q.bbf
CDCl3
1.02 1.01 1.01 0.94 2.02 1.00 24.52 2.14 1.15 2.11 1.01 2.01 9.17
9 8 7 6 5 4 3 2 1 0 ppm

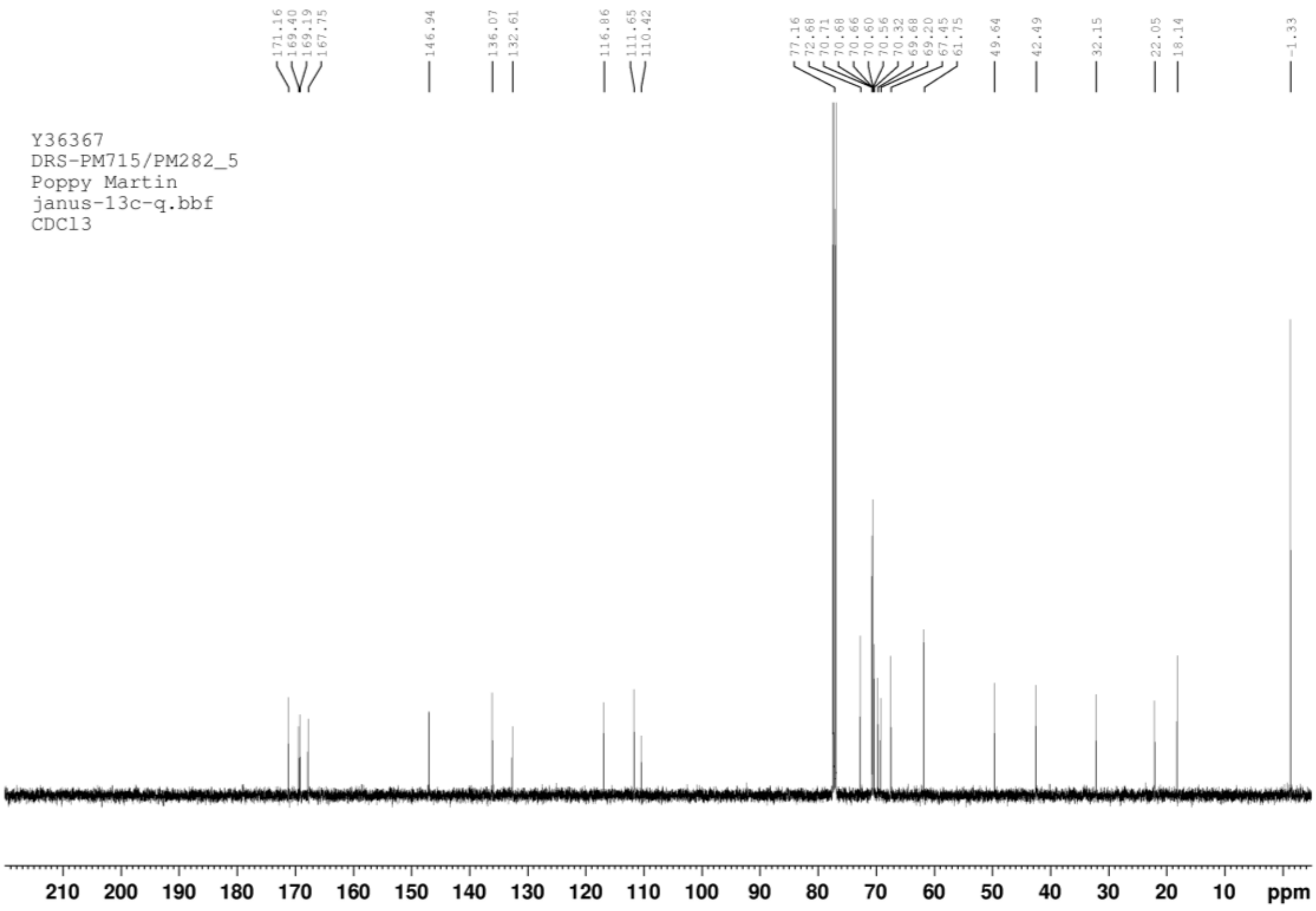
171.16 169.40 169.19 167.75 146.94 136.07 132.61 116.86 111.65 110.42 77.16 72.68 70.71 70.68 70.66 70.60 70.56 70.32 69.68 69.20 67.45 61.75 49.64 42.49 32.15 22.05 18.14 -1.33
Y36367
DRS-PM715/PM282_5
Poppy Martin
janus-13c-q.bbf
CDCl3
210 200 190 180 170 160 150 140 130 120 110 100 90 80 70 60 50 40 30 20 10 ppm

(16n)

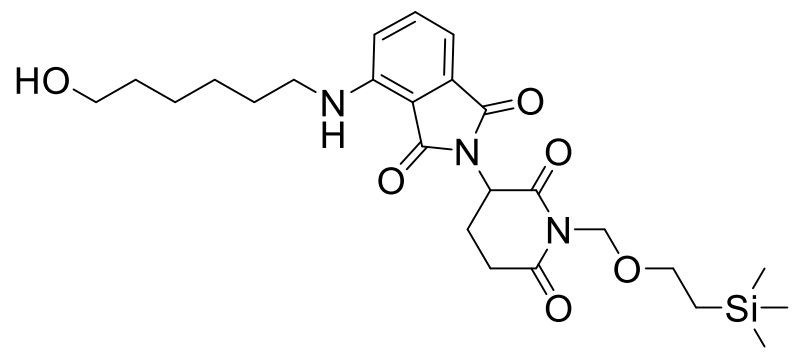
HO
N
H
O
N
O
O
N
O
O
Si

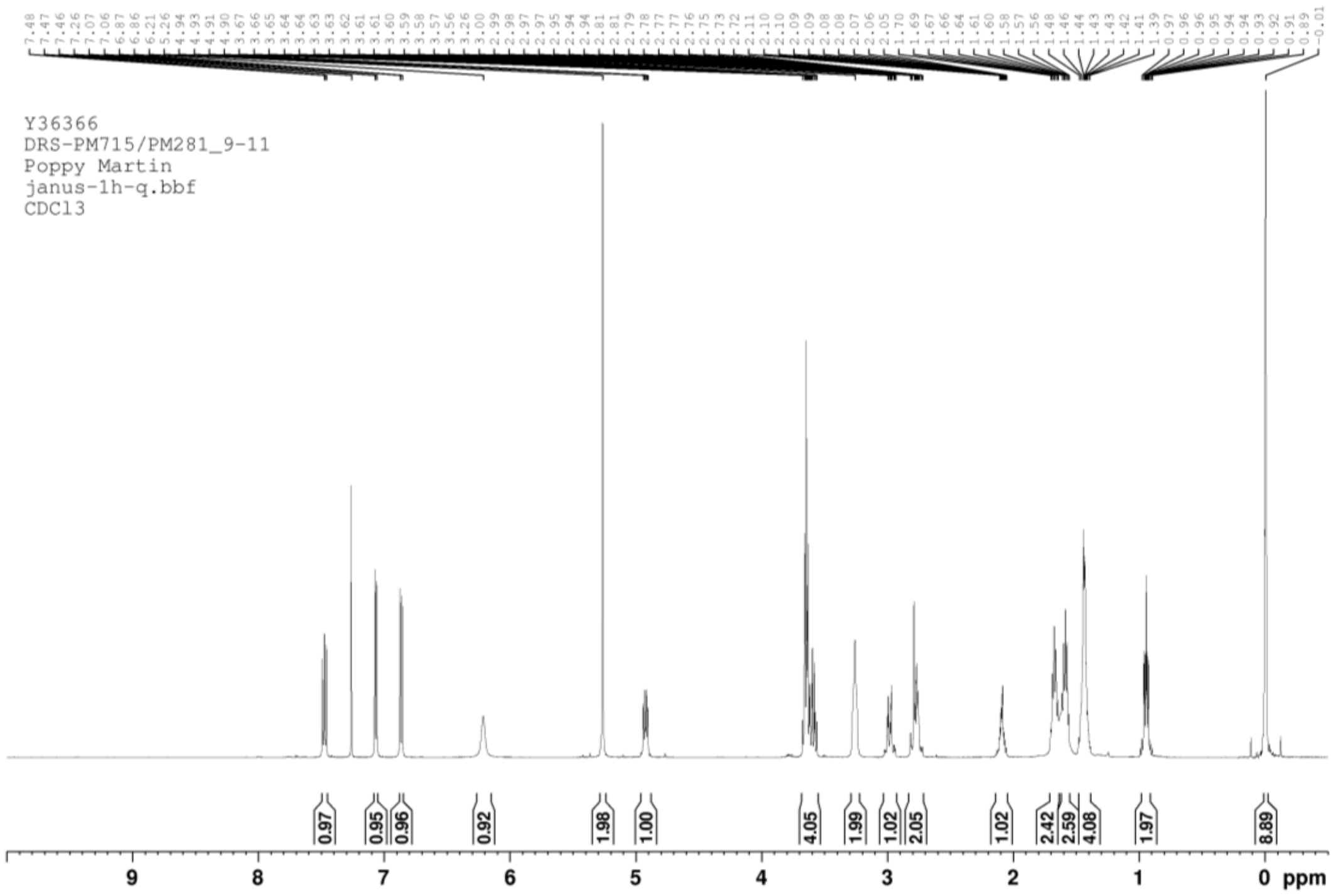
Y36366
DRS-PM715/PM281_9-11
Poppy Martin
janus-1h-q.bbf
CDCl3
0.97
0.95
0.96
0.92
1.98
1.00
4.05
1.99
1.02
2.05
1.02
2.42
2.59
4.08
1.97
8.89
9
8
7
6
5
4
3
2
1
0 ppm

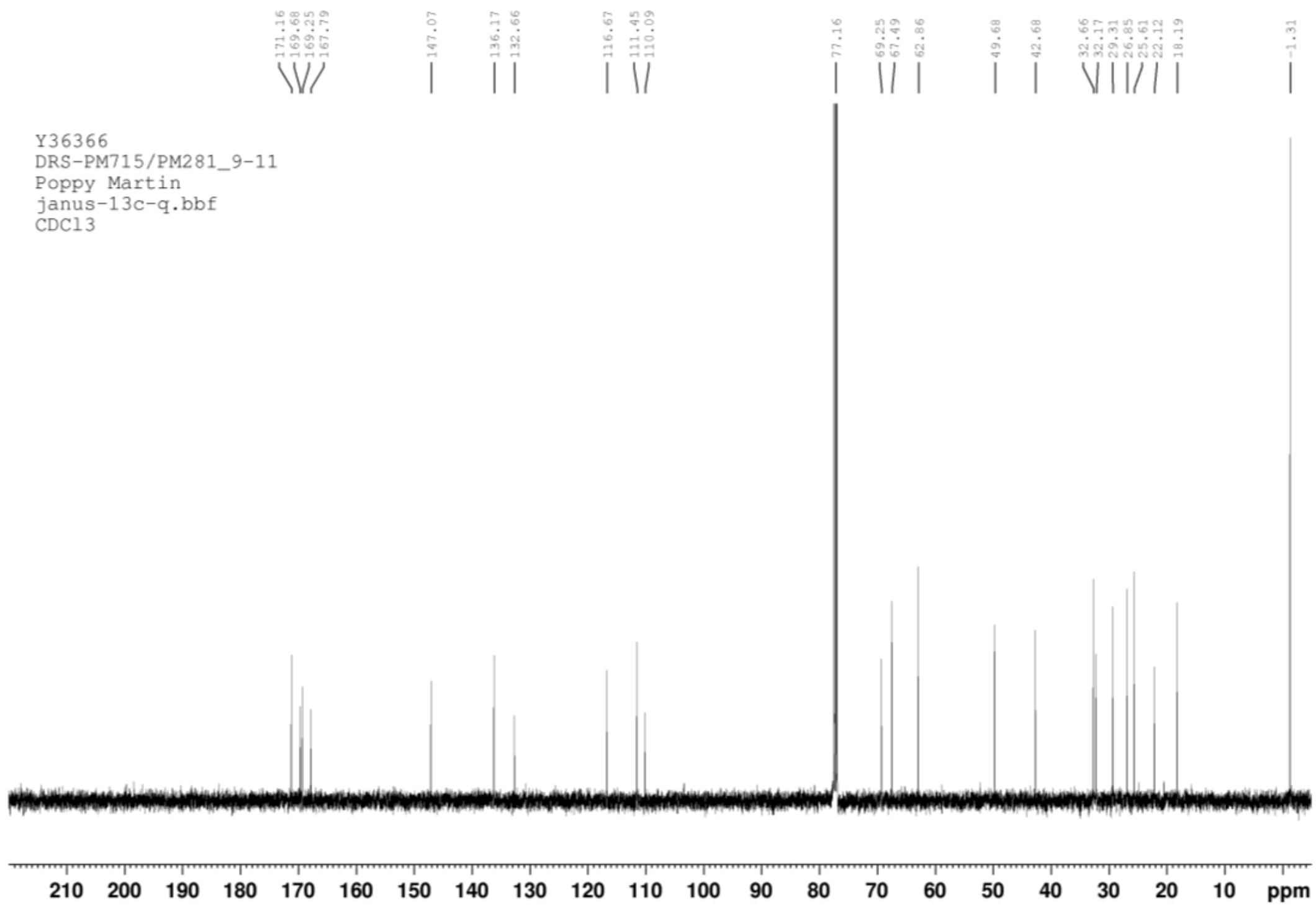
171.16
169.68
169.25
167.79
147.07
136.17
132.66
116.67
111.45
110.09
77.16
69.25
67.49
62.86
49.68
42.68
32.66
32.17
29.31
26.85
25.61
22.12
18.19
-1.31
Y36366
DRS-PM715/PM281_9-11
Poppy Martin
janus-13c-q.bbf
CDCl3
210 200 190 180 170 160 150 140 130 120 110 100 90 80 70 60 50 40 30 20 10 ppm

(16o)

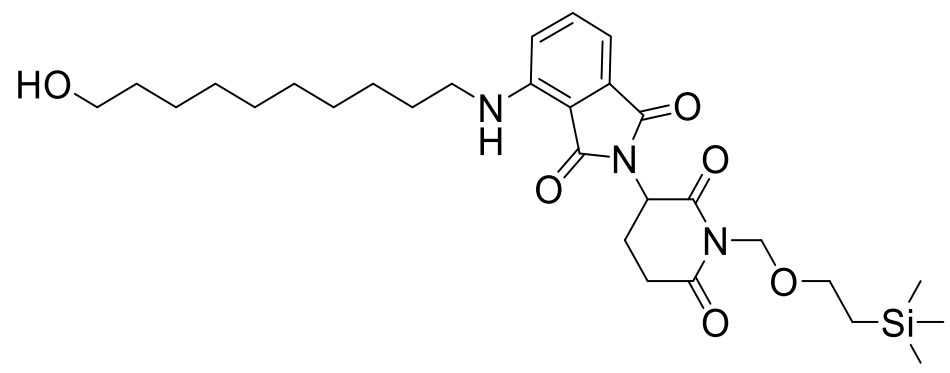
HO
N
H
O
N
O
O
N
O
O
Si

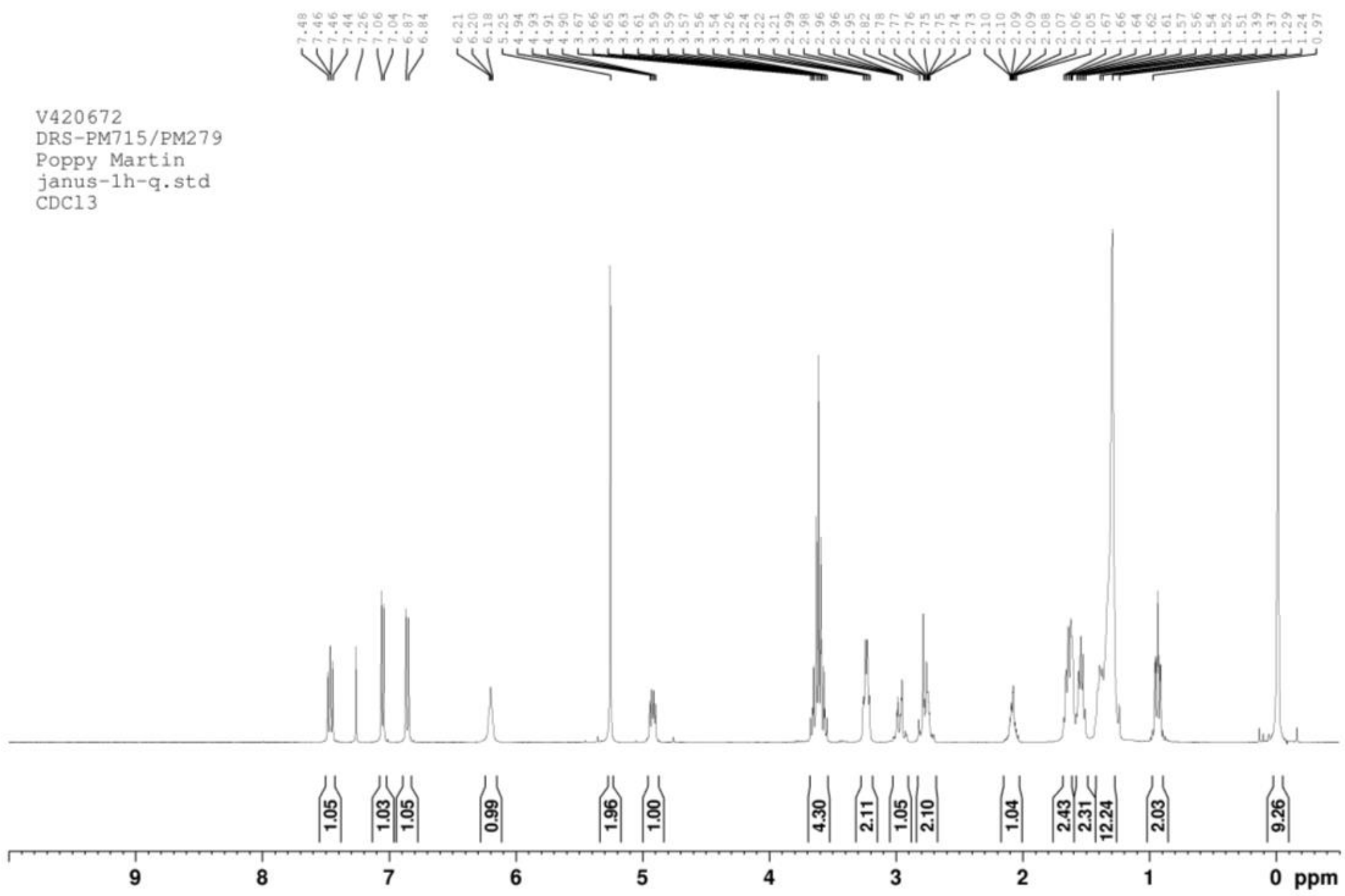
V420672
DRS-PM715/PM279
Poppy Martin
janus-1h-q.std
CDCl3
1.05
1.03
1.05
0.99
1.96
1.00
4.30
2.11
1.05
2.10
1.04
2.43
2.31
12.24
2.03
9.26
ppm

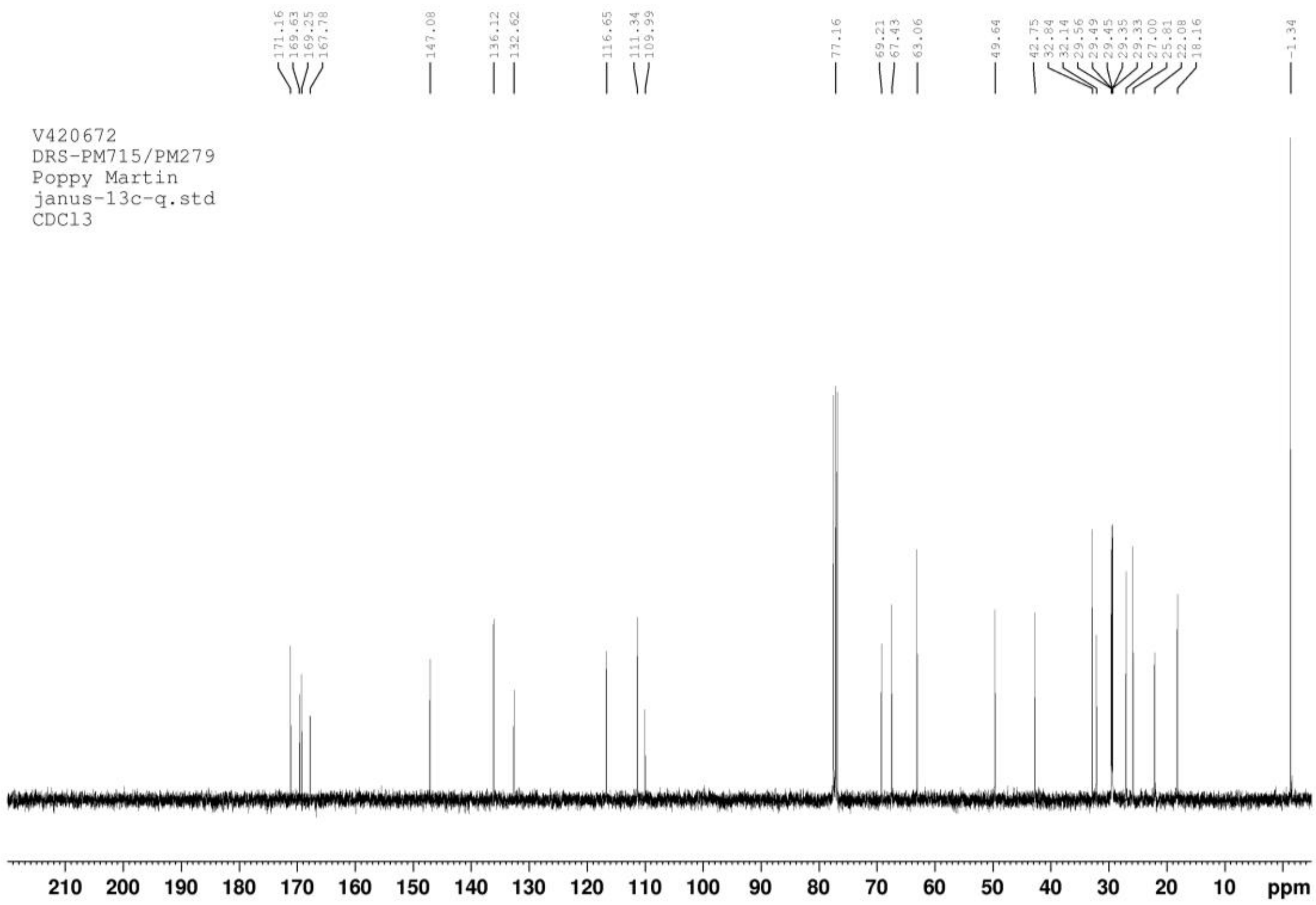
171.16
169.63
169.25
167.78
147.08
136.12
132.62
116.65
111.34
109.99
77.16
69.21
67.43
63.06
49.64
42.75
32.84
32.14
29.56
29.49
29.45
29.35
29.33
27.00
25.81
22.08
18.16
-1.34
V420672
DRS-PM715/PM279
Poppy Martin
janus-13c-q.std
CDCl3
ppm

PROTAC M

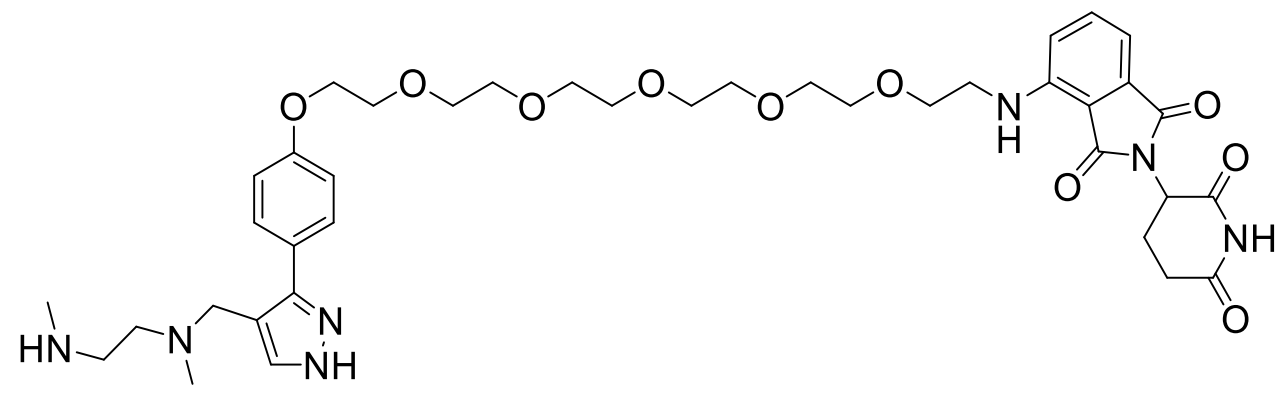
O
O
O
O
O
O
N
H
O
N
O
O
NH
O
HN
N
N
NH

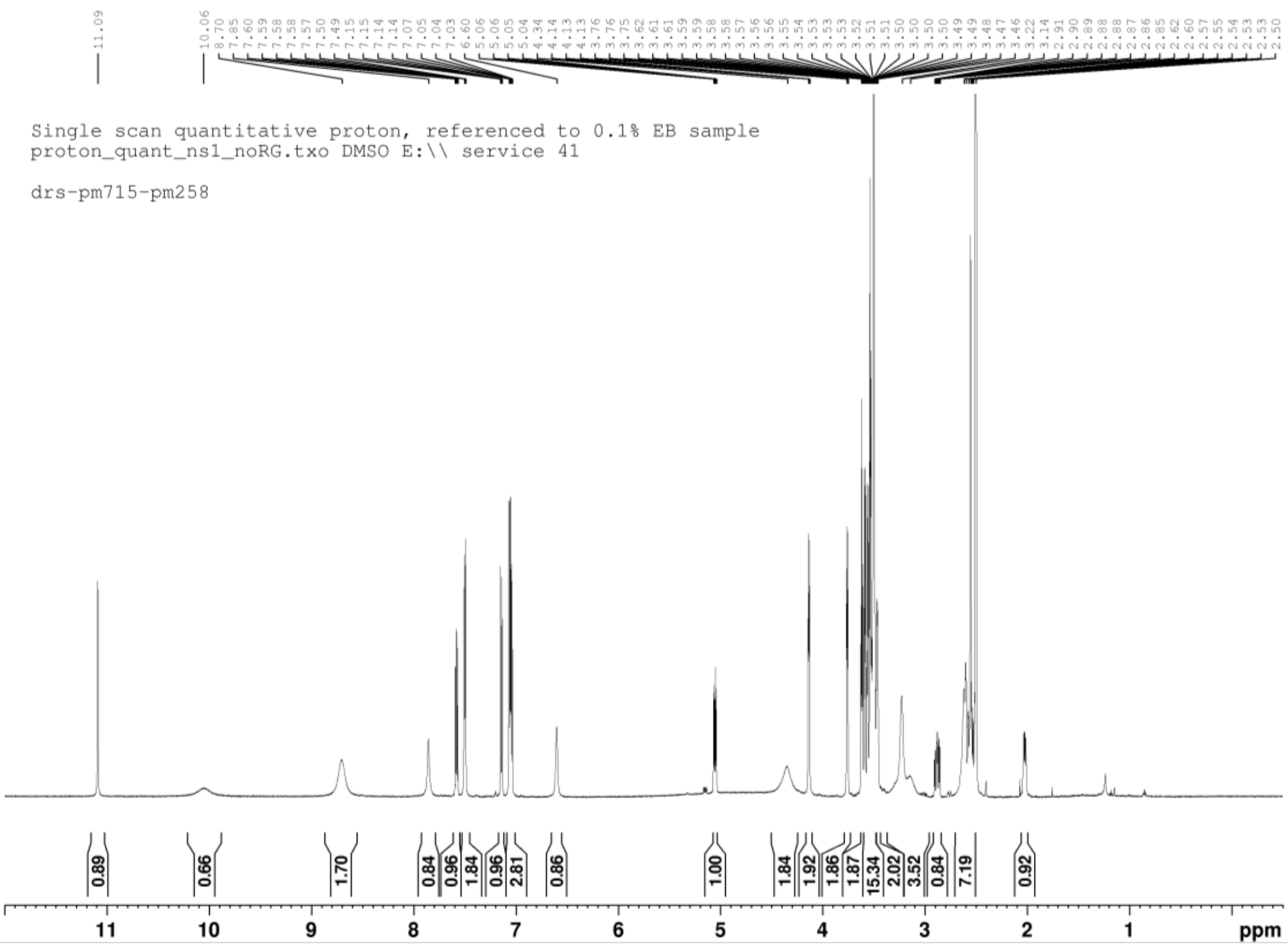
Single scan quantitative proton, referenced to 0.1% EB sample
proton_quant_ns1_noRG.txo DMSO E:\\ service 41
drs-pm715-pm258
0.89
0.66
1.70
0.84
0.96
1.84
0.96
2.81
0.86
1.00
1.84
1.92
1.86
1.87
15.34
2.02
3.52
0.84
7.19
0.92
ppm

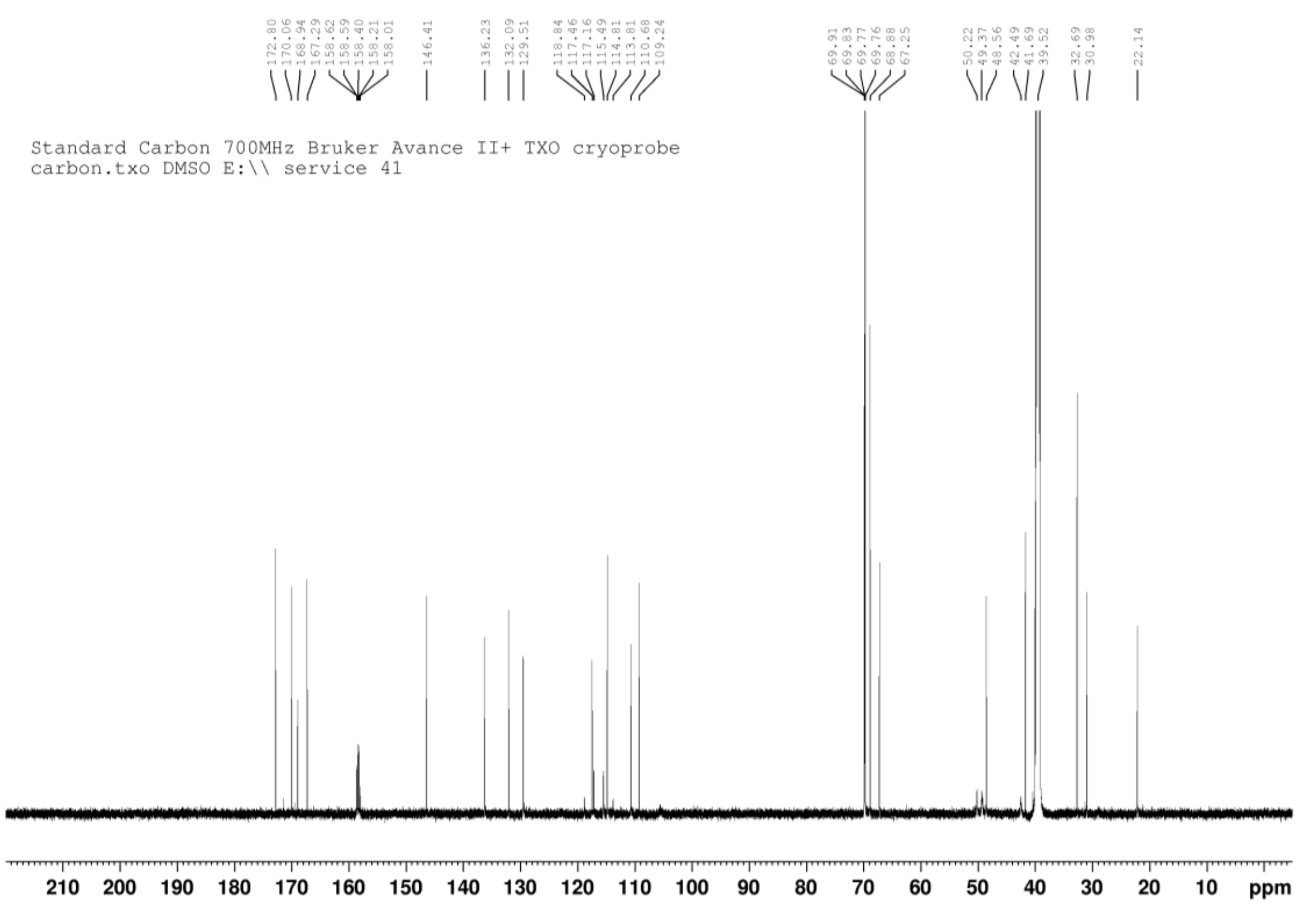
Standard Carbon 700MHz Bruker Avance II+ TXO cryoprobe
carbon.txo DMSO E:\\ service 41
ppm

PROTAC N

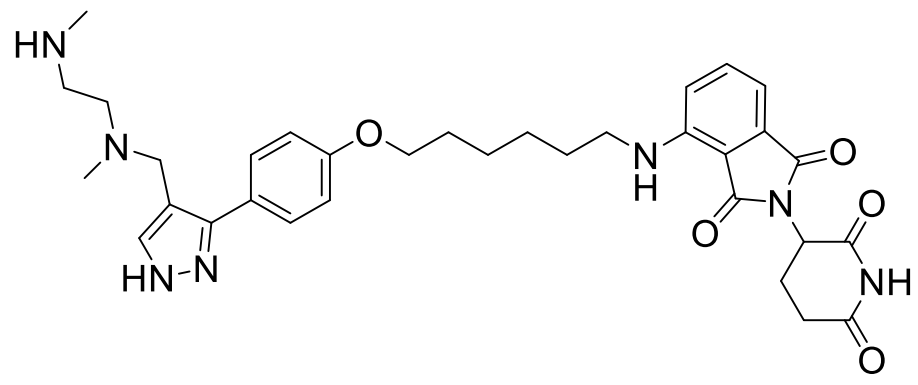
HN
N
O
N
H
O
N
O
O
NH
O
HN-N

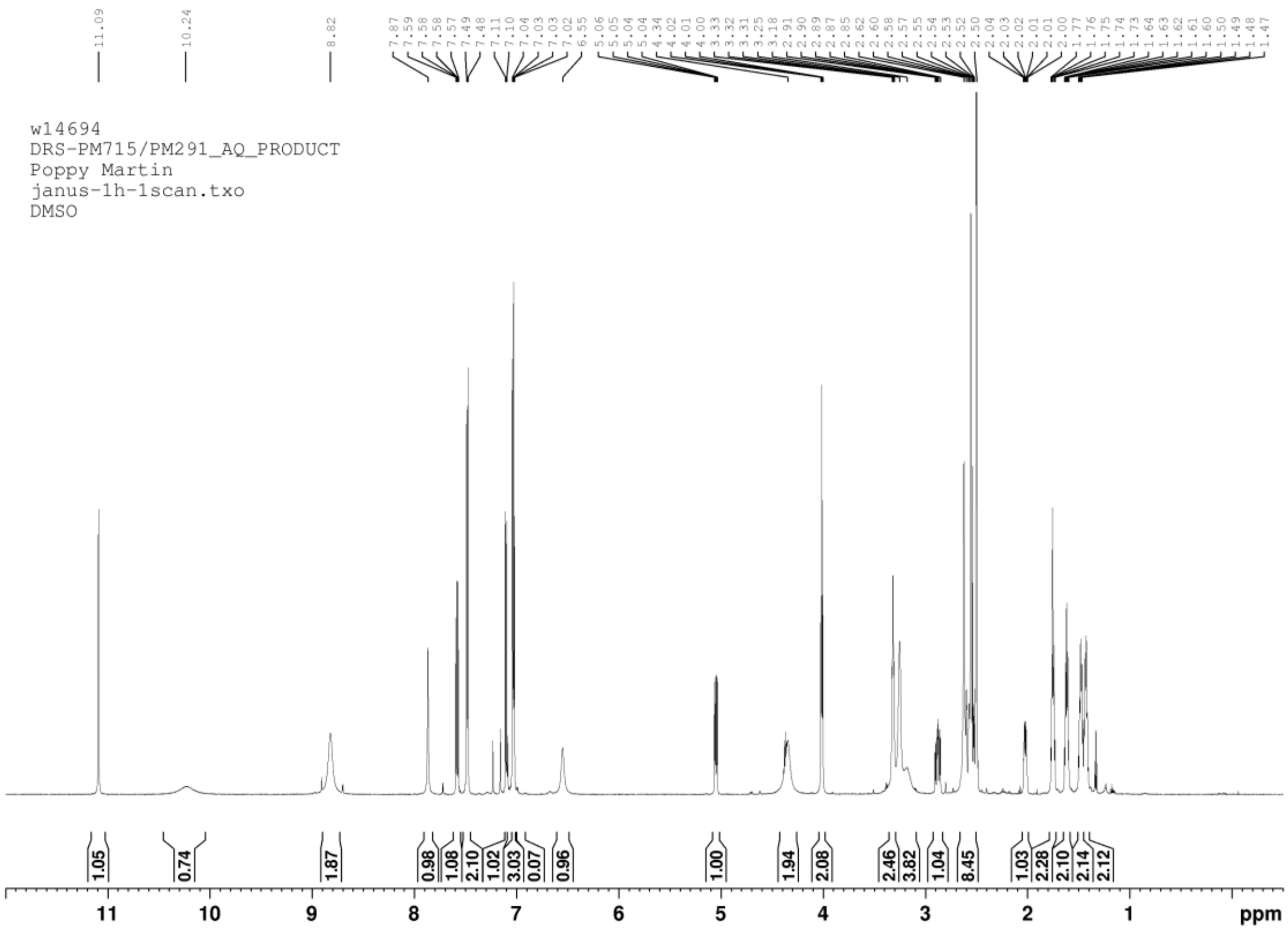
w14694
DRS-PM715/PM291_AQ_PRODUCT
Poppy Martin
janus-1h-1scan.txo
DMSO
11.09
10.24
8.82
7.87 7.59 7.58 7.58 7.57 7.49 7.48 7.11 7.10 7.04 7.03 7.03 7.02 6.55
5.06 5.05 5.04 4.34 4.02 4.01 4.00 3.33 3.32 3.31 3.25 3.18 2.91 2.90 2.89 2.87 2.85 2.62 2.60 2.58 2.57 2.55 2.54 2.53 2.52 2.50 2.04 2.03 2.02 2.01 2.01 2.00 1.77 1.76 1.75 1.74 1.73 1.64 1.63 1.62 1.61 1.60 1.50 1.49 1.48 1.47
1.05
0.74
1.87
0.98
1.08
2.10
1.02
3.03
0.07
0.96
1.00
1.94
2.08
2.46
3.82
1.04
8.45
1.03
2.28
2.10
2.14
2.12
11
10
9
8
7
6
5
4
3
2
1
ppm

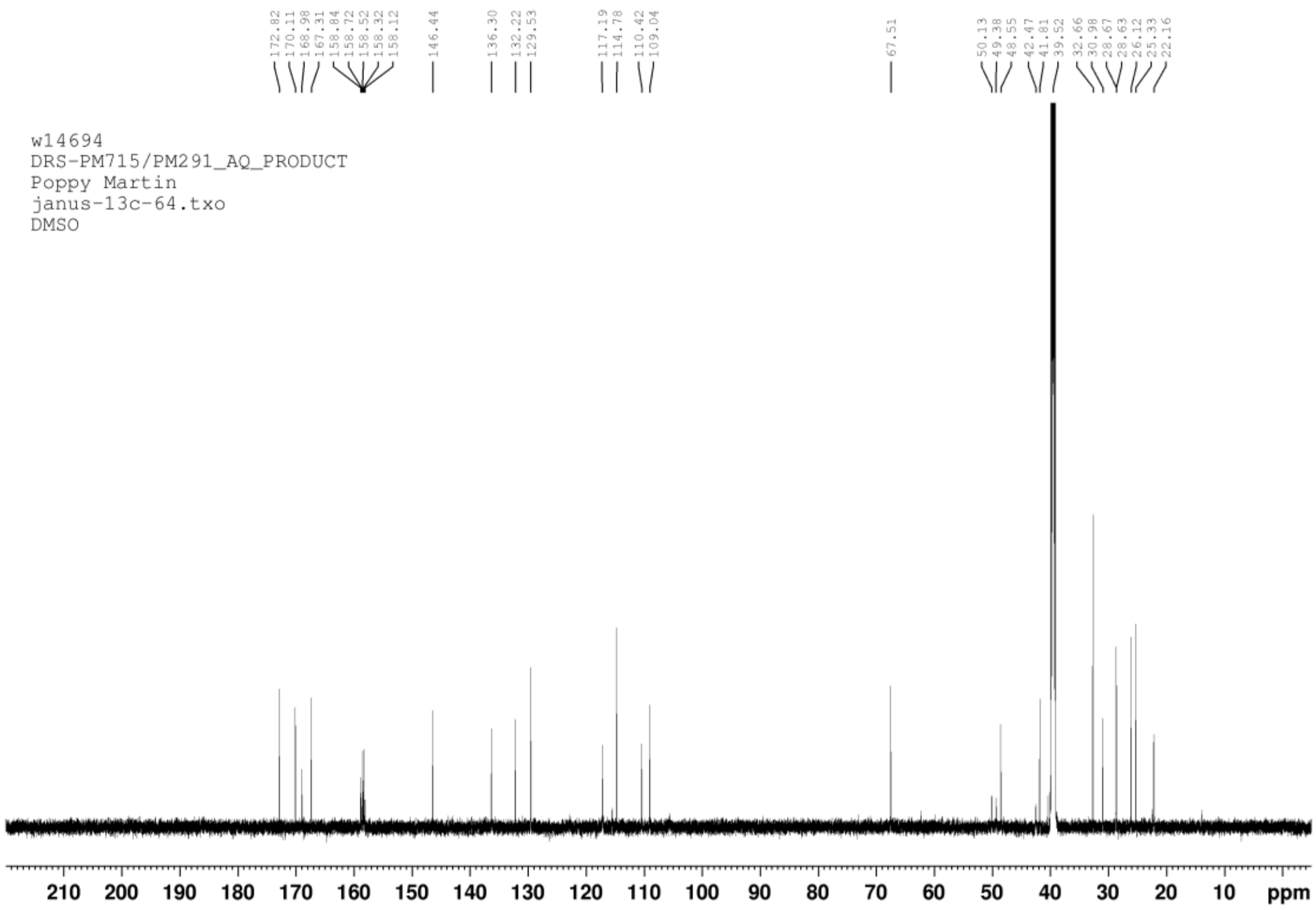
w14694
DRS-PM715/PM291_AQ_PRODUCT
Poppy Martin
janus-13c-64.txo
DMSO
172.82 170.11 168.98 167.31 158.84 158.72 158.52 158.32 158.12
146.44
136.30 132.22 129.53
117.19 114.78 110.42 109.04
67.51
50.13 49.38 48.55 42.47 41.81 39.52 32.66 30.98 28.67 28.63 26.12 25.33 22.16
210
200
190
180
170
160
150
140
130
120
110
100
90
80
70
60
50
40
30
20
10
ppm

PROTAC O

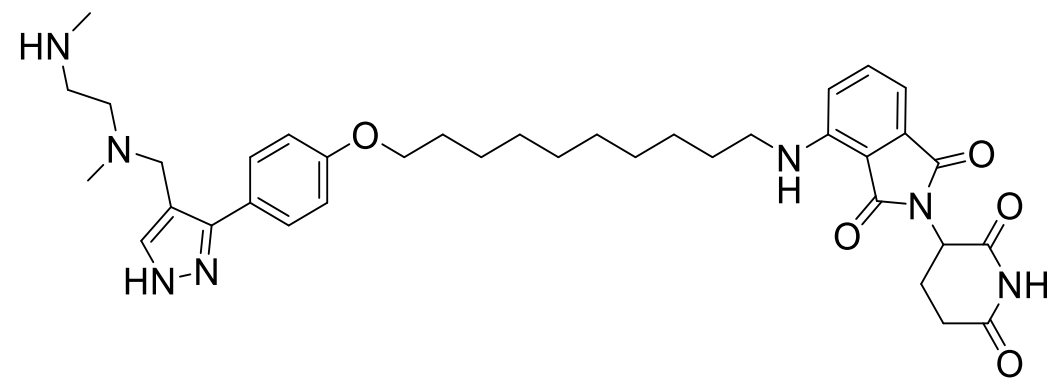
HN
N
O
N
H
O
N
O
O
NH
O
HN-N

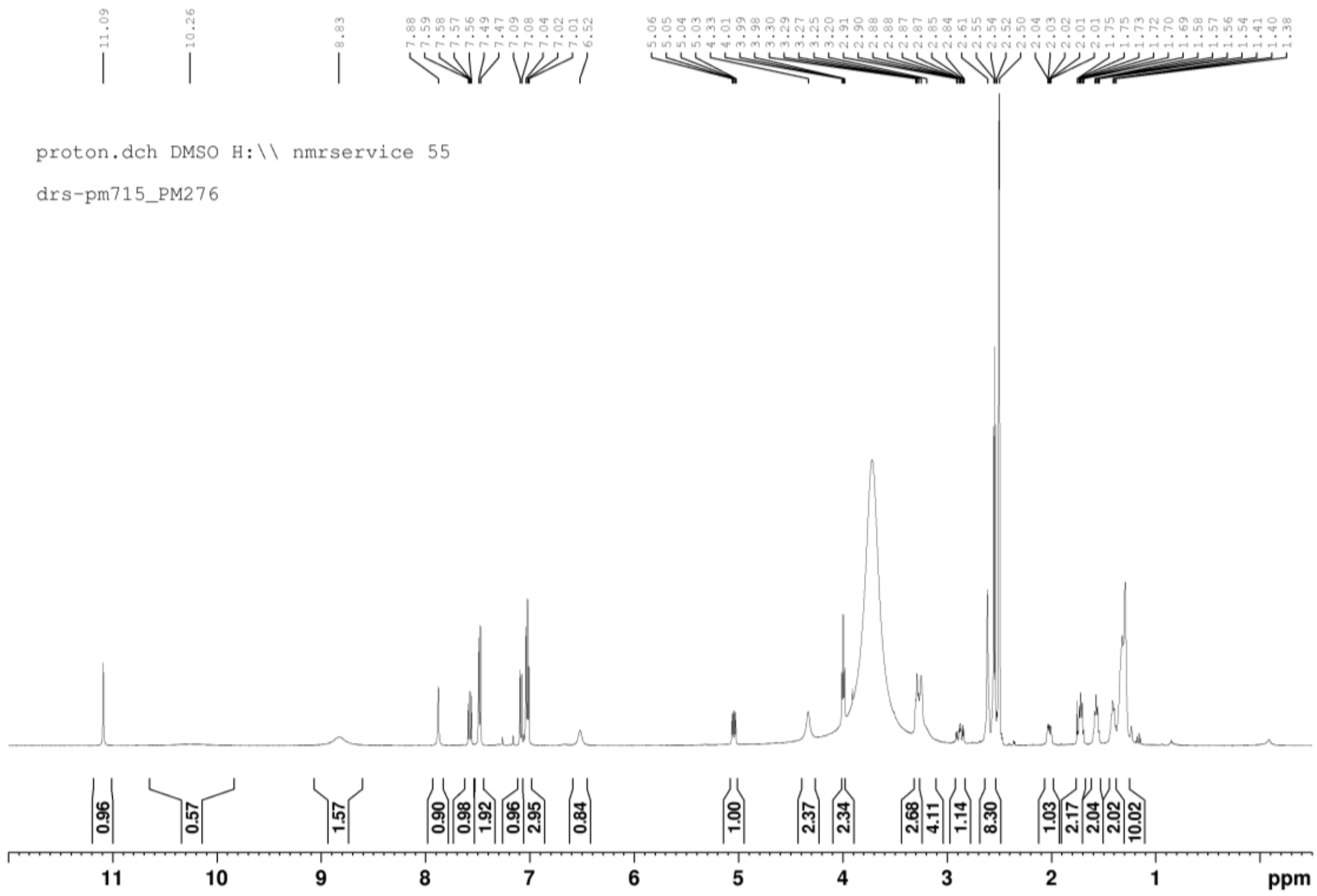
proton.dch DMSO H:\\ nmrservice 55
drs-pm715_PM276
ppm

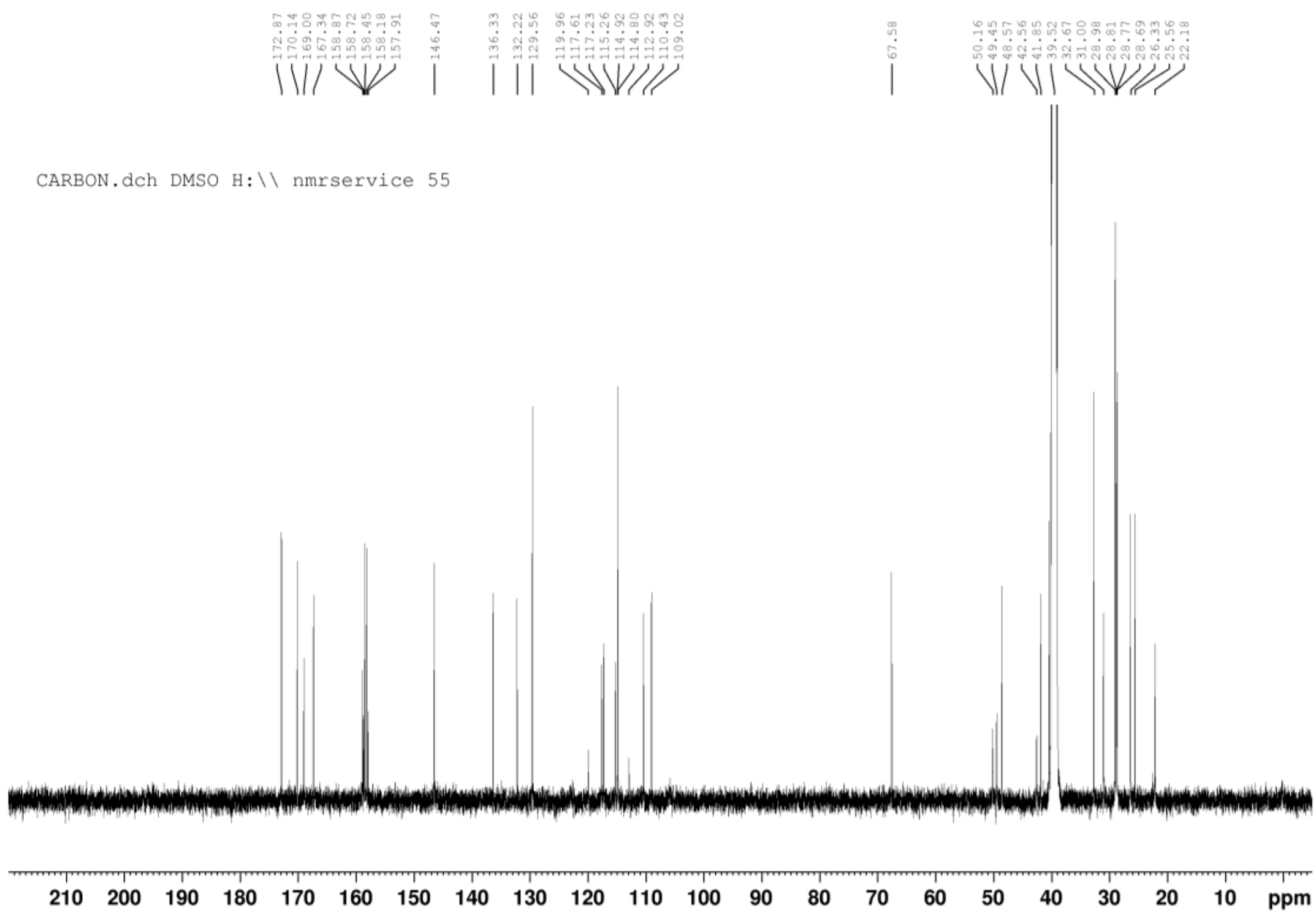
CARBON.dch DMSO H:\\ nmrservice 55
ppm
